# Male animal sterilization: history, current practices, and potential methods for replacing castration

**DOI:** 10.3389/fvets.2024.1409386

**Published:** 2024-07-03

**Authors:** Rex A. Hess, Chan Jin Park, Sandra Soto, Lindsey Reinacher, Ji-Eun Oh, Mary Bunnell, CheMyong J. Ko

**Affiliations:** ^1^Department of Comparative Biosciences, College of Veterinary Medicine, University of Illinois at Urbana-Champaign, Urbana, IL, United States; ^2^Epivara, Inc, Champaign, IL, United States

**Keywords:** castration, sterilization, chemical sterilization, immunocastration, gene manipulation, population control, aggressive behavior, sexual behavior

## Abstract

Sterilization and castration have been synonyms for thousands of years. Making an animal sterile meant to render them incapable of producing offspring. Castration or the physical removal of the testes was discovered to be the most simple but reliable method for managing reproduction and sexual behavior in the male. Today, there continues to be global utilization of castration in domestic animals. More than six hundred million pigs are castrated every year, and surgical removal of testes in dogs and cats is a routine practice in veterinary medicine. However, modern biological research has extended the meaning of sterilization to include methods that spare testis removal and involve a variety of options, from chemical castration and immunocastration to various methods of vasectomy. This review begins with the history of sterilization, showing a direct link between its practice in man and animals. Then, it traces the evolution of concepts for inducing sterility, where research has overlapped with basic studies of reproductive hormones and the discovery of testicular toxicants, some of which serve as sterilizing agents in rodent pests. Finally, the most recent efforts to use the immune system and gene editing to block hormonal stimulation of testis function are discussed. As we respond to the crisis of animal overpopulation and strive for better animal welfare, these novel methods provide optimism for replacing surgical castration in some species.

## 1 Introduction and history

The purpose of this review is to look into the background related to the use of castration in male animals and examine the search for a replacement. This includes research on gonad-sparing sterilization, contraception, and extensive studies on the effects of chemical toxicology in male reproduction. Reproductive sterilization is a procedure that used to make an individual incapable of producing offspring (infertile), but it can also render the male deficient in sex steroid hormones and, in some cases, is used for health purposes. Castration is a simple medical procedure for physical removal of the testes (gonadectomy), and for thousands of years, the two words, sterilization and castration, have been used interchangeably. However, modern science and medicine have extended the meaning of male sterilization to include methods for inhibiting the development of sperm capable of fertilizing an egg, chemical destruction of testicular parenchyma, and the blockage of sperm transport through the reproductive tract. The terms used for male sterilization are extensive and now include ‘chemical castration’, ‘immunocastration’, ‘emasculation’, ‘neutering’, and ‘vasectomy’. Some of these terms are associated with leaving the testes intact (sparing the gonad) and some provide only temporary infertility (or contraception), unless applied on a regular basis, although contraceptive methods can induce sterility if continued for a long period (1–5).

Evidence of castration reaches back in ancient times, with discoveries connecting its practice in animals and humans for thousands of years (6). Very early, it was discovered that castration provided control over sex hormone-induced behavior and breeding in domesticated herd kept for secondary animal products, such as wool and milk (7). Evidence exists that herd of castrated sheep and goats were maintained during the Uruk period approximately 4,000 BC and some claim as early as 9,000 years ago (8). Archeologists have dated castrated cattle in burial sites by examining horn cores and skeletons, in which evidence of hormonal control over growth was observed, long before the field of endocrinology was even established (7, 9). The first recorded use of castration in domestic animals was for the creation of geldings, or castrated male horses, which was documented in the 7th Century BC by the Scythians (10). This practice continues to be performed today by veterinarians and trained farm personnel (11).

Human castration is generally considered repulsive in modern society, and its use has been limited to cases of violent sexual offenders or for medical treatment (8, 12). However, historically, castration of humans dates back for thousands of years. Some believe that castration of humans was derived from the established practice in animal herds. In the earliest recorded history, human slaves were castrated to make them display *better behavior* (8). In the Byzantine Empire and earlier, castration was an accepted practice and eunuchs served in unique systems of hierarchy as trusted servants in royal families (7, 8, 13–15). This practice in China lasted for over 2000 years and did not end until the 1900s (6). Castration became woven into the ancient society and was even performed for religious and spiritual reasons, and voluntary eunuchs can be found in some countries even today (6, 16). The early forms of male sterilization were invasive and carried significant risks, a far cry from the safe and controlled medical procedures was known today. Nowadays, when castration is used for sex offenders, there is debate over whether surgical removal of the testes should be replaced with chemical treatments (17, 18). In contrast, different methods of castration, including orchiectomy and Gonadotropin-Releasing Hormone (GnRH) agonists, are positively used as a form of androgen-deprivation therapy for the management of prostate cancer (19).

Castration has become a mainstay in food production herd animals and expanded to include domesticated pet species and zoo animals (20, 21). From a purely biological perspective, this medical procedure has become a routine practice in cats and dogs to control reproduction, inhibit hormone-induced sexual behavior, and control androgen-induced cancers, such as prostate carcinoma. Castration in dogs and cats gained popularity as a method to prevent animal overpopulation and sexual behavior, long before it was studied for treating prostate hypertrophy and cancer in aging male dogs (22). Studies have even indicated that in dogs, castration increases the lifespan by several years, with neutered dogs being less likely to die of trauma and infectious, vascular, and degenerative diseases (23, 24). However, this hypothesis is being challenged at least for some breeds, such us the Rottweiler, as neutering at young ages can reduce the lifespan of a dog (25).

The age of the animal is a major factor to take into consideration when selecting a sterilization method to use in specific species and under specific conditions. For male pigs, the neonatal period is considered ideal for castration because piglets are easier to handle than older pigs (26), and gonad removal stops the production of androgens which is responsible for aggression in the growing boars and ‘boar taint’, an offensive odor or taste that occurs more frequently in pork from uncastrated male pigs (27). In domesticated dogs and cats, spays and neuters at 6 to 9 months of age are standard practice in veterinary medicine (28). In animal care and control facilities, surgical sterilization is performed as early as 7 weeks (pediatric spay/neuter) before animals are adopted out (29). Under certain conditions, such as cryptorchidism, it is recommended that castration can be delayed until the inguinal canal is closed, approximately 6 months of age (30). However, some studies suggest that early castration may increase the risk of specific diseases in certain breeds (23, 29, 31, 32). In cats, prepuberal castration (before 5 months) can cause adhesion of the prepuce to the penis at sexual maturity; however, this can be avoided by giving intramuscular testosterone treatments (33, 34). Thus, determining the appropriate age for sterilization depends on various factors for each animal species or breed and is relevant to the selection of an optimal method.

This review will provide a comprehensive background of the biological targets of sterilization in male animals and introduce purpose- and species-dependent methods of sterilization that are currently used by veterinarians and farmers. Products currently being tested and potential future methods are also discussed. Historically, the evolution of male sterilization in animals has gone through several phases, starting with surgical castration, ligation and crushing of the spermatic cord, and then moving to chemical (non-hormonal) sterilization. In the early 1960s, occurring during the early days in the establishment of toxicology as a discipline (35–37), chemicals were introduced for sterilization in mammals, primarily as rodenticides (38), but they were also tested in monkeys (39) and dogs (40). By the 1970s and 80s, chemosterilants were being tested in numerous mammalian species (39, 41). Various methods of treatment were also studied, including direct injection of chemicals into the testis (42), epididymis (43), and vas deferens (44). While the use of chemicals in male sterilization continues to be investigated today (45, 46), the pathological responses and inflammatory reactions, as well as general toxicity, have contributed to their lack of success in domesticated animals. In the case of male rodent species, the focus on basic research into the reproductive toxicity of various chemicals has led to the development of several sterilization products (47). The death of the animal and secondary health effects are not significant concerns in the control of pests, which has facilitated the approval and use of these sterilization products.

The use of hormones and hormonal agonists/antagonists formed the next phase for advancing the methods of sterilization in both male and female reproduction (48–56). In most cases, the results have not produced sterility, instead only contraception has produced (i.e., temporary inhibition of fertility). However, sterility can be produced by hormonal treatment, depending on the age at treatment (57). Congruently, immunocastration methods have been developed in the form of vaccines against specific peptide hormones and other proteins that are involved in the regulation of male reproduction. Some of these vaccines are already commercially used in farm animals while still being tested in cats and dogs. While immunocastration remains a viable alternative to surgical sterilization, the next major phase in the development of male sterilants appears to be genetics, more specifically the use of gene editing.

## 2 Male reproductive system and potential targets for sterilization

Male reproduction is complex and depends on the development and physiology of several organs, including the brain, pituitary, testes, epididymides, vas deferens, ejaculatory ducts, and accessory sex organs, all of which can vary from species to species. For male sterilization, each of these organs and their specific cell types and unique functions have been considered as potential targets, although castration has been the method of choice for thousands of years. Today, sterilization is achieved by three main approaches; (1) surgical removal of the reproductive organ(s), i.e., castration and vasectomy; (2) pharmacological suppression of the function of reproductive organs; (3) inhibition of the development or maturation of the reproductive organs. The organs of the male reproductive system and the potential cellular targets within each which have received consideration for inducing sterilization are presented here.

### 2.1 Hypothalamus and pituitary

**Figure 1:**
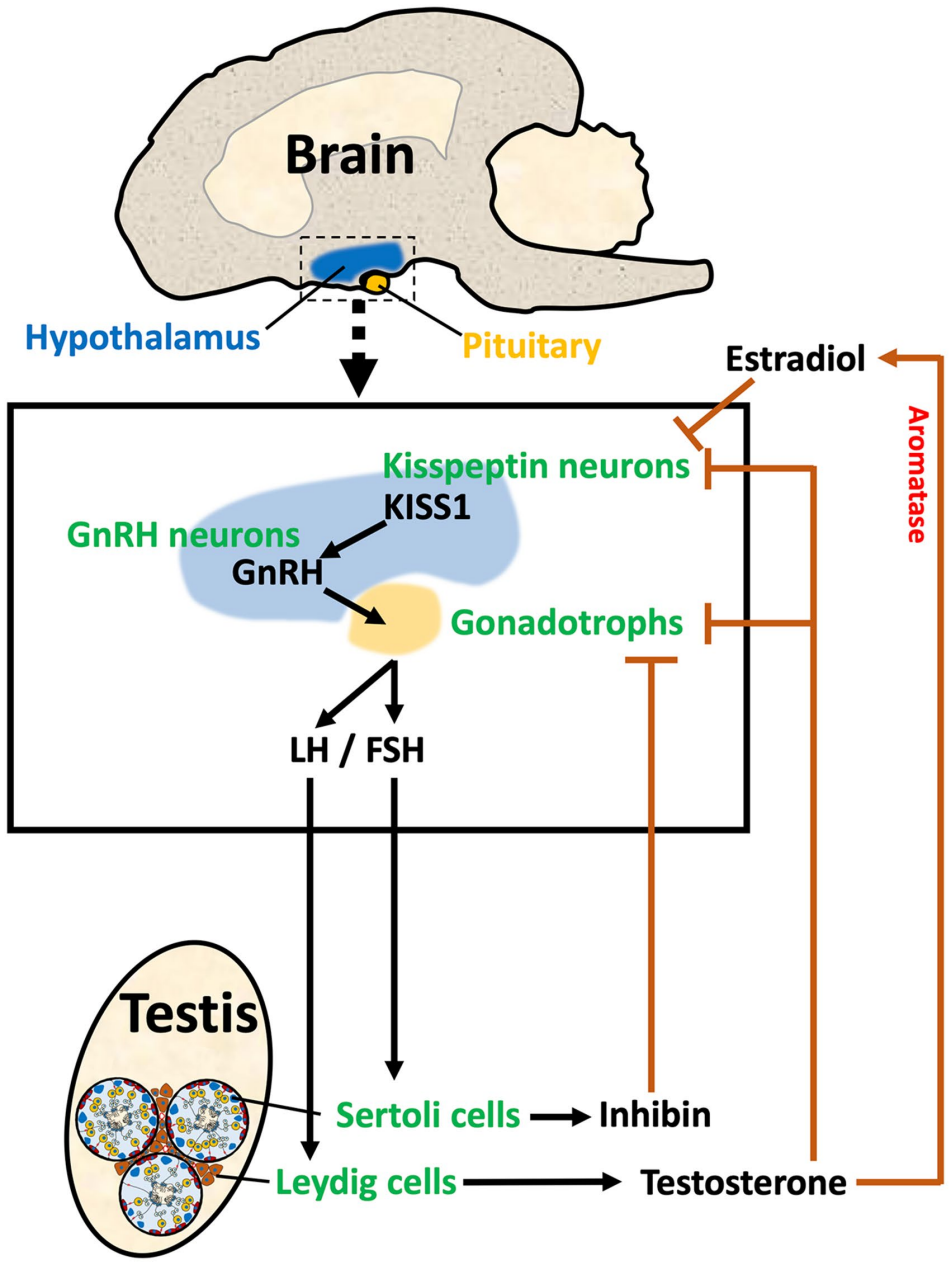
The HPG axis in male reproduction. The diagram illustrates the three components of the Hypothalamus/Pituitary/Gonadal axis in the male. In the hypothalamus, Kisspeptin (KISS1) from Kisspeptin neurons stimulates the release of GnRH from the GnRH neuron. GnRH stimulates the synthesis and release of gonadotropins (LH and FSH) in the pituitary. LH and FSH travel via the vasculature to the testis where they stimulate Leydig and Sertoli cells, respectively. Leydig cells in the testis interstitial space produce sex steroids, such as testosterone, which provides negative feedback to the hypothalamus and pituitary by binding to androgen receptors on both the Kisspeptin neurons and the gonadotrophs. Aromatase is also found in the hypothalamus, which allows for the conversion of testosterone to estradiol, providing a local negative feedback to decrease KISS1. Sertoli cells within the seminiferous tubules produce inhibin-B, which provides negative feedback to the gonadotrophs in the pituitary. Thus, the HPG axis has a regulatory system that maintains the required levels of hormones for stimulation of testicular function.

The male reproductive system begins in the brain region called hypothalamus. This region initiates the cascade of hormonal stimulations required for the development and function of peripheral reproductive organs. The hypothalamus along with the pituitary and gonads form the reproductive axis, hence called HPG axis, in which a hormone produced in one area either stimulates or inhibits the secretion of a hormone in the other organ via positive and negative feedback loops. Hypothalamic neurons produce two key hormones, Kisspeptin (KISS1) and GnRH (58–61). In a unidirectional regulatory mechanism, KISS1 binds to KISS1 receptor (KISS1R; also known as GPR54) on the GnRH neuron cell membrane, triggering the release of GnRH (62, 63). GnRH travels through a hypophyseal portal system to the anterior pituitary and stimulates the secretion of gonadotropins, luteinizing hormone (LH), and follicle-stimulating hormone (FSH), which stimulates the male gonads to produce sex steroids, peptide hormones, and spermatozoa (Figure 1). There are multiple sites within the HPG axis which are potential targets for the purpose of inducing contraception or sterilization in the male, and both GnRH and Kisspeptin neurons can be targeted through multiple approaches.

In the hypothalamus, two distinctive populations of Kisspeptin neurons regulate the secretory activity of GnRH neurons. One population is located in the arcuate nucleus (ARC), and the other is located in the anteroventral periventricular nucleus (AVPV) in rodents (64) or the preoptic area (POA) in humans (65). The pulsatile activity of ARC Kisspeptin neurons is synchronized with GnRH and LH pulse patterns in males and females (66–68). Maintaining physiological levels of circulating gonadotropin concentrations and LH secretion requires the proper interval of kisspeptin neuron activation (69, 70). There are considerable differences between males and females, especially males have a smaller population of anterior (i.e., AVPV) kisspeptin neurons and fewer fiber connections with GnRH cell bodies than females, resulting in the absence of an LH surge (71, 72).

Kisspeptin neurons are potential targets for sterilization, as a deficiency in the number of kisspeptin neurons results in infertility in both males and females. Indeed, the removal or mutation of either *Kiss1* or *Kiss1r* genes resulted in hypogonadism and sterility (73–78). Interestingly, studies revealed that exposure to estrogen during the neonatal period was shown to decrease the number of kisspeptin neurons present in the AVPV and ARC regions (57, 79, 80). The detailed mechanisms related to this phenomenon have not been fully elucidated, but some studies suggest that estrogen-induced apoptosis of kisspeptin neurons is a contributing factor (80, 81).

Rodent studies revealed that kisspeptin neurons influence male sexual behavior, including mounting and thrusting, as well as the typical male-like olfactory partner preferences (82). This highlights an essential role for KISS1 signaling in the development and expression of sexually dimorphic behavior (82). Recent studies on the regulation of sexual behaviors have uncovered a role for KISS1 expression in the amygdala (MeA) (83, 84), with MeA kisspeptin neurons playing a crucial role in the olfactory reproductive pathways (85), especially in males (86). Inhibition of this pathway could be a method for inducing male infertility by blocking sexual behaviors that are necessary for animal breeding.

The expression of neurokinin B and its receptor in the ARC is crucial for postnatal testis development. In the mouse knockout of *Tacr3*, the neurokinin B (NKB) receptor gene resulted in significantly smaller testes and lower FSH levels than normal animals (87). In the ARC, most of the cells expressing KISS1 also express NKB, but some cells do not. Approximately 67% of kisspeptin neurons express TAC3 and 84% of NKB neurons express KISS1 in cats. In dogs, nearly 100% of the kisspeptin neurons expressed NKB and 49% of the NKB neurons expressed KISS1 (88). Therefore, blocking both kisspeptin and NKB neurons could be an ideal approach for inhibiting functional development of the gonads and provides a new target of sterilization.

The GnRH neuron is also a major target for controlling male reproduction. Much of the effort to target GnRH neurons has focused on chemical inhibition of GnRH release, as well as the use of antibodies against the peptide (see sections 3.4 and 3.5.4). An indirect method has also been used to inhibit the function of GnRH neurons by targeting VAX1. This transcription factor is known to be essential for the maturation of GnRH neurons and directly activates the GnRH promoter by binding to specific sites, which is crucial for fertility regulation. In mice, the deletion of *Vax1* from GnRH neurons led to infertility in both males and females, as well as delayed puberty and hypogonadism (89).

The anterior pituitary consists of five major hormone-producing cell types: somatotrophs, thyrotrophs, lactotrophs, corticotrophs, and gonadotrophs. Each of these secretes specific hormones, growth hormone, thyrotropin, prolactin, ACTH, and gonadotropins, respectively. The pulsatile releases of the gonadotropins into the blood are crucial for normal male reproduction (90), although all of these peptide hormones have indirect effects on testis development and function. GnRH stimulates the secretion of LH and FSH from gonadotrophs, but continuous GnRH stimulation of the pituitary results in decreased LH and FSH secretion via desensitizing the GnRH receptor (69). Thus, long-term inactivation of gonad function can be achieved through continuous treatment with GnRH agonists; however, in many species, including dogs, cats, sheep, and primates, this effect is reversible (91–94).

In the testis, LH binds to receptors on Leydig cells in the interstitium, stimulating the production of testosterone, whereas FSH binds to Sertoli cells lining the seminiferous tubules and helps to regulate their proliferation prior to puberty. After puberty, once Sertoli cells begin to express androgen receptor (AR), FSH enhances the action of testosterone, which provides the support needed for germ cell progression through the final phases of spermatogenesis (95, 96). In males, a feedback loop in the HPG axis is established by the secretion of testicular hormones. In addition to responding to estrogen and testosterone, cells in the hypothalamus are also capable of converting testosterone to estrogen (specifically, 17β-estradiol, or E2) by expressing aromatase (97). ARC neurons express estrogen receptor 1 (ESR1) and AR (98), and the removal of androgen production by castration causes increased KISS1 expression in the ARC, which results in GnRH stimulation of gonadotropin secretion. Thus, androgens are negative regulators of ARC kisspeptin neurons (98, 99). Inhibin-B, a major peptide hormone produced by Sertoli cells in response to FSH, also contributes to the negative feedback loop between testes and the pituitary by suppressing the secretion of FSH (100).

### 2.2 Testes

The testis has two major compartments: seminiferous tubules and interstitial space or intertubular region. Sertoli and germ cells occupy the seminiferous tubule epithelium, both of which can be targeted for sterilization (101). Sertoli cells are the somatic cells whose cytoplasm extends as complex thin arms around the developing germ cells, guiding their proliferation and differentiation through multiple phases of spermatogenesis to produce spermatozoa that are released into the lumen (102). The intricate physical association of Sertoli cells with germ cells begins with the Sertoli–Sertoli tight junction, which is a major part of the blood–testis barrier (BTB). The BTB must be traversed by large cohorts of preleptotene spermatocytes held together by thin cytoplasmic bridges (103). Part of this tight junctional complex between Sertoli cells is a structure called the basal ectoplasmic specialization, an actin filament/endoplasmic reticulum-associated structure, which, if disrupted chemically, renders the male infertile (104–107). The ectoplasmic specialization is retained in the apical cytoplasm and serves as the anchoring device to allow Sertoli cells to hold and traffic germ cells within the epithelium until they are released by spermiation (108, 109). A review of the molecular components maintain Sertoli cell physiology, and the BTB reveals an array of potential targets for impeding spermatogenesis, several of which have been experimentally targeted by chemical exposure and proposed as potential male contraceptives (106). Due to the close relationship between the Sertoli cell membrane and developing germ cells, any disturbance in Sertoli cell physiology will always result in abnormal development or loss of germ cells within the epithelium (110).

Sertoli cells are required for the formation of the seminiferous tubules and maintenance of germ cell development. During fetal and neonatal development, Sertoli cells are partially dependent on FSH for proliferation and establishment of the adult population, but in adults, both FSH and testosterone are required for Sertoli cells to maintain quantitatively normal spermatogenesis (111, 112). Therefore, Sertoli cells are a potential target for inducing infertility; however, blocking FSH stimulation alone would not necessarily induce sterility but rather produce a significant decline in sperm production. In contrast, blocking testosterone production would prevent Sertoli cell’s maintenance of sperm production and stop sexual behavior.

Some chemicals directly affect the germ cells, as it is possible to target spermatocytes and the process of meiosis (105), spermiogenesis or formation of the spermatids (105, 113), and specific steps in sperm release (105, 114, 115). However, the best germ cell target for sterilization would be the spermatogonium. Spermatogonia may be directly inhibited or indirectly inhibited by disrupting the integrity and function of the BTB, which is essential for spermatogenesis in adulthood (105). Destruction of spermatogonial stem cells results in progressive loss of all germ cells, leaving only Sertoli cells and inducing permanent sterility (116, 117). Spermatogonial stem cells are regulated by hormones and growth factors from Sertoli, Leydig, and peritubular cells, and their self-renewal is dependent on specific genes such as GDNF, ETV5, and ID4 signaling pathways (118, 119). Although spermatogonial stem cell renewal can be inhibited (118), in light of the importance of stem cell biology in other organs, careful consideration must be given to potential serious multisystemic side-effects. Specifically, GDNF, ETV5, and ID4 signaling play crucial roles in the differentiation and survival of neurons, as well as in the quiescence of stem cells (120–122). Therefore, the disruption of these signaling pathways by intervention of exogenous chemicals could potentially lead to dysfunctions in the brain and spinal cord.

Leydig cells of the testis synthesize androgens, most importantly testosterone, and are a primary source of intratesticular estrogen. Thus, Leydig cells are targeted if the goal is to inhibit androgen production and sexual behavior in addition to inducing sterility, as they synthesize androgens, most importantly testosterone, and are also a primary source of intratesticular estrogen (123, 124). Leydig cells are located in the space identified as the interstitium, between seminiferous tubules, which also contains blood vessels, open lymphatics and sinusoids, immune-reactive cells, and peritubular cells (125). This location is important because testosterone is released into circulation for stimulation of masculine characteristics throughout the body and serves as an essential Sertoli cell stimulus for the maintenance of spermatogenesis (110, 126). A crucial factor in targeting Leydig cells is age of the male. During gestation, LH is not required for fetal Leydig cell synthesis of testosterone (127). However, after birth, there is a shift to LH dependency for Leydig cell maturation at puberty and androgen production (125, 128). Thus, after birth, the Leydig cell could be indirectly targeted by inhibiting the HPG axis (129).

### 2.3 Male reproductive tract (efferent ductules, epididymis, vas deferens)

Spermatozoa leave the testis via rete testis chambers and enter the male reproductive tract, where they travel slowly during storage and maturation before ejaculation. The testes have tracts that consist of multiple efferent ductules coming from the rete testis, forming single epididymal tubes, and ending with the vas deferens and a common ejaculatory duct (130).

Efferent ductules are thin, convoluted tubules that connect rete testis with the epididymis and thus are found adjacent to the testis (131). This proximal location provides a unique target for sterilization, as blockage of the lumens in the ductules will prevent sperm from entering the epididymis and, in some species and under specific conditions, can lead to testicular atrophy (132, 133). Efferent ductules are the only site in the male tract having motile (kinetic) cilia, which vigorously agitate the luminal fluid (134, 135), an activity required for the maintenance of fluid reabsorption (131, 132, 136). Efferent ductules have kidney-like physiology and reabsorb nearly 90% of the fluid in the lumen, thereby increasing sperm concentration 28-fold before passage into the epididymis (137). Both the ciliated and non-ciliated cells are potential targets for male sterility. Disturbing the function of either cell type can result in permanent damage to the head of the epididymis and, consequently, reduced fertility or cause sterility in some cases (131–133, 135, 138, 139). However, the basic anatomy of these tubules differs between large and small mammals. In smaller mammals, such as rodents, several ductules near the rete testis merge into a single, very thin, common duct near the head of the epididymis, thus forming a funnel. This forces the fluid and sperm to release into the epididymis through a single small tubule, creating a potential problem. In larger mammals (dogs, cats, pigs, cattle, and human) and birds, numerous efferent ductules released from the rete testis with only a few cranial ducts merging to enter the top of the epididymis, while most remain independent, providing multiple entries into the side of the epididymis. Thus, smaller mammals will be more susceptible to blockage and fluid accumulation than larger mammals due to the funnel formation leaving only one exit for sperm (133). On the other hand, fluid reabsorption is inhibited rather than creating a blockage; as observed in the *Esr1*-knockout male (138–140), it would be possible to induce sterilization in all mammals by diluting the semen and affecting sperm maturation in the lumen.

Numerous efforts have also been made to create a male contraceptive or induce sterility by targeting the epididymis to disrupt sperm maturation or block sperm transport (141, 142). The epididymis forms a single highly convoluted tubule between the efferent ductules and vas deferens. As spermatozoa pass through the epididymis, they acquire progressive motility and fertilizing ability; thus, the epididymal epithelium is uniquely established to create a nurturing environment to facilitate these physiological functions. In general, there are four major regions beginning with the head of the epididymis (initial segment and caput), the corpus (middle), and cauda (storage area). It has often been overlooked that the head of the epididymis in larger mammals consists almost entirely of coiled efferent ductules that enter the single epididymal tube (130). This important distinction must be considered when comparing chemical effects on the epididymis in different species, but older literature often used the entire epididymis for analyses and called it “epididymis” while including all the efferent ductules. Analysis of the rat epididymal transcriptome revealed at least 19 distinct segments of the epididymis, based on differential gene expression patterns (143). Each segment has distinct and overlapping functions, derived from structural and molecular differences among the basic epithelial cell types. Thus, there are numerous potential targets for contraception and sterilization within the epididymis (142). Principal cells synthesize essentially all proteins secreted into the lumen but also support long, branched, microvilli that continue the reabsorption of luminal fluids. Narrow and clear cells actively secrete protons into the lumen, thereby lowering the fluid pH, which helps to maintain the maturing sperm in a non-motile state (144). The basal cells have stem cell features and may be able to regenerate the epithelium (145). Most importantly, they have axiopodia that can reach the lumen in some regions and participate in transepithelial transport and physiological cross-talk with the clear cells (144). In addition to the basic epithelial cells, the epididymis has a highly regulated immune environment consisting of monocytes, macrophages, lymphocytes, and intraepithelial dendritic cells (144, 146).

The vas deferens is a muscular tube that transports sperm from the cauda epididymis to the ejaculatory duct. The three layers of smooth muscle across a folded epithelium with branched microvilli provide a powerful propulsion of sperm at ejaculation (147). Because the vas deferens is easily located and palpable, vasectomy has become one of the most successful, simple sterilization methods for men (148) and has also been proposed for use in dogs and cats (3, 149). Based on its success in men, there have been numerous attempts to adapt a non-surgical method of vas occlusion by injecting various types of sclerosing agents that induce fibrosis and blockage of the lumen (see Section 3.3.2). Although this method has been focused on adult males, there is no reason to believe that it could not also be performed in prepubertal animals, resulting in sterility while maintaining testicular function and testosterone production. In feral animals, epididymal blockage and vasectomy have been proposed as being advantageous in some species by providing breeding competition in the population (150, 151).

**Figure 2:**
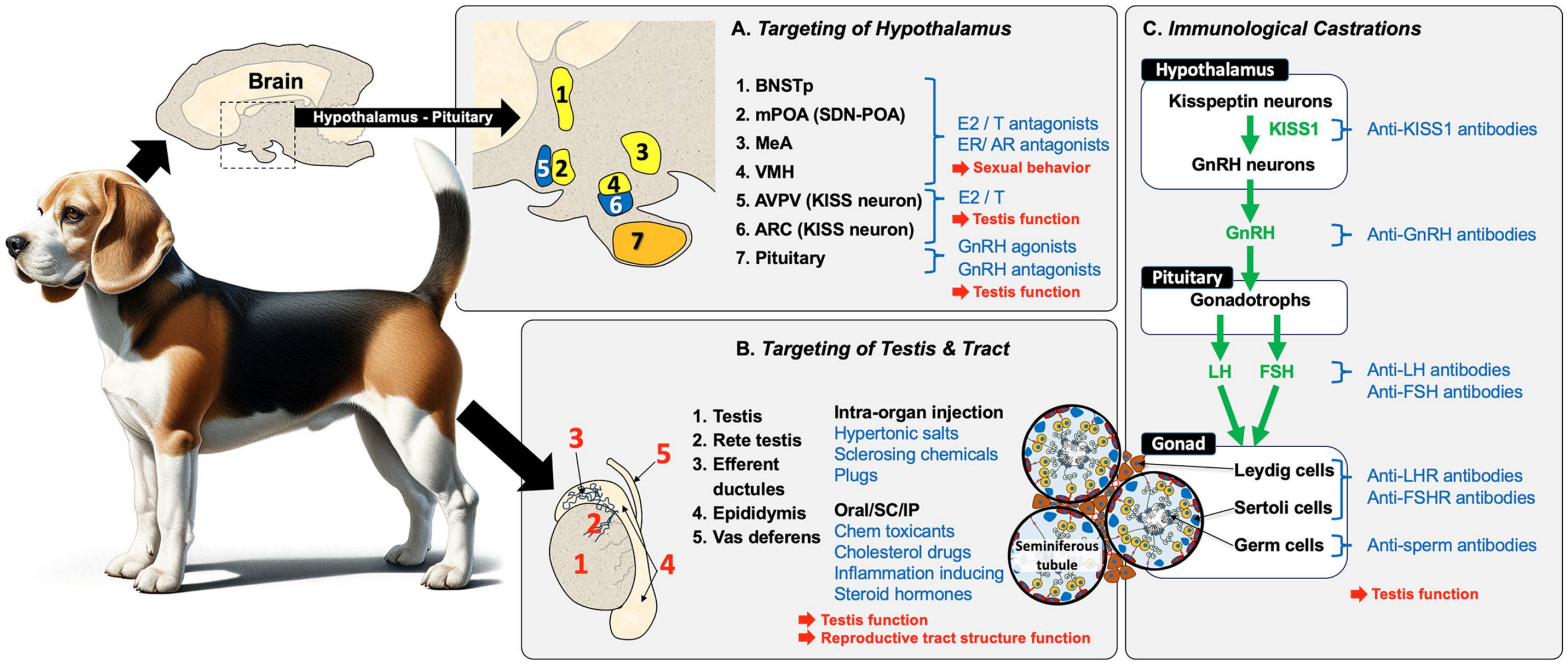
Overview of potential methods for replacing castration for inducing sterility in male animals. Sterilization methods for male mammals (i.e., dogs), can be broadly categorized into three approaches that result in the blocking of sperm production and fertility in the male: **(A)** Targeting of the hypothalamus/pituitary region is a common approach. Estrogens (E2) and androgens (T) and their antagonists inhibit sexual behavior and testicular function by indirectly inhibiting GnRH production and, if given neonatally, can induce permanent infertility. Another approach involves the use of GnRH agonists/antagonists, but these require periodic administration to achieve complete sterilization. **(B)** Direct chemical targeting of the testis and reproductive tract has been attempted using several methods, including intra-organ injections. Direct injection into the testis, epididymis, or vas deferens can induce tissue necrosis and fibrosis, which blocks the transport of sperm. Blockage can also induce sterility by injecting a polymer into the vas deferens lumen. A variety of chemicals have also been given orally, subcutaneously (SC), or intra-peritoneally (IP) to produce specific effects that involve testicular toxicity, or inhibition of specific pathways (e.g., cholesterol synthesis), as well as inflammation, which contributes to cellular necrosis. **(C)** Immunological castration uses antibodies or stimulation of antibody production to target components within the HPG axis, including KISS1, GnRH, LH, and FSH in the hypothalamus and pituitary. Immunological methods can also directly target the testes by inducing antibodies against the gonadotropin hormone receptors, LHR and FSHR, as well as target sperm proteins to inhibit motility or fertilizing ability. However, vaccine-induced infertility is only temporary and requires periodic administration.

In summary, the male reproductive system across all mammalian species begins with a peptide hormone regulatory pathway in the hypothalamus and pituitary. This system sets up a feedback loop with hormones produced in the target sexual organ, the testis. Because the goal is to produce mature germ cells (spermatozoa) that can be ejaculated and fertilize eggs in the female reproductive tract, there are numerous checkpoints to target for potential sterilization of the male (Figure 2). Some targets can provide a permanent infertility, while others have the potential for recovery and thus would be labeled as contraceptive. Some targets will inhibit the production of testosterone and thus dampen or remove sexual behavior, as well as prevent the production of sperm, while other targets will only block the release of sperm by ejaculation.

## 3 Methods of sterilization

### 3.1 Surgical sterilization

**Figure 3:**
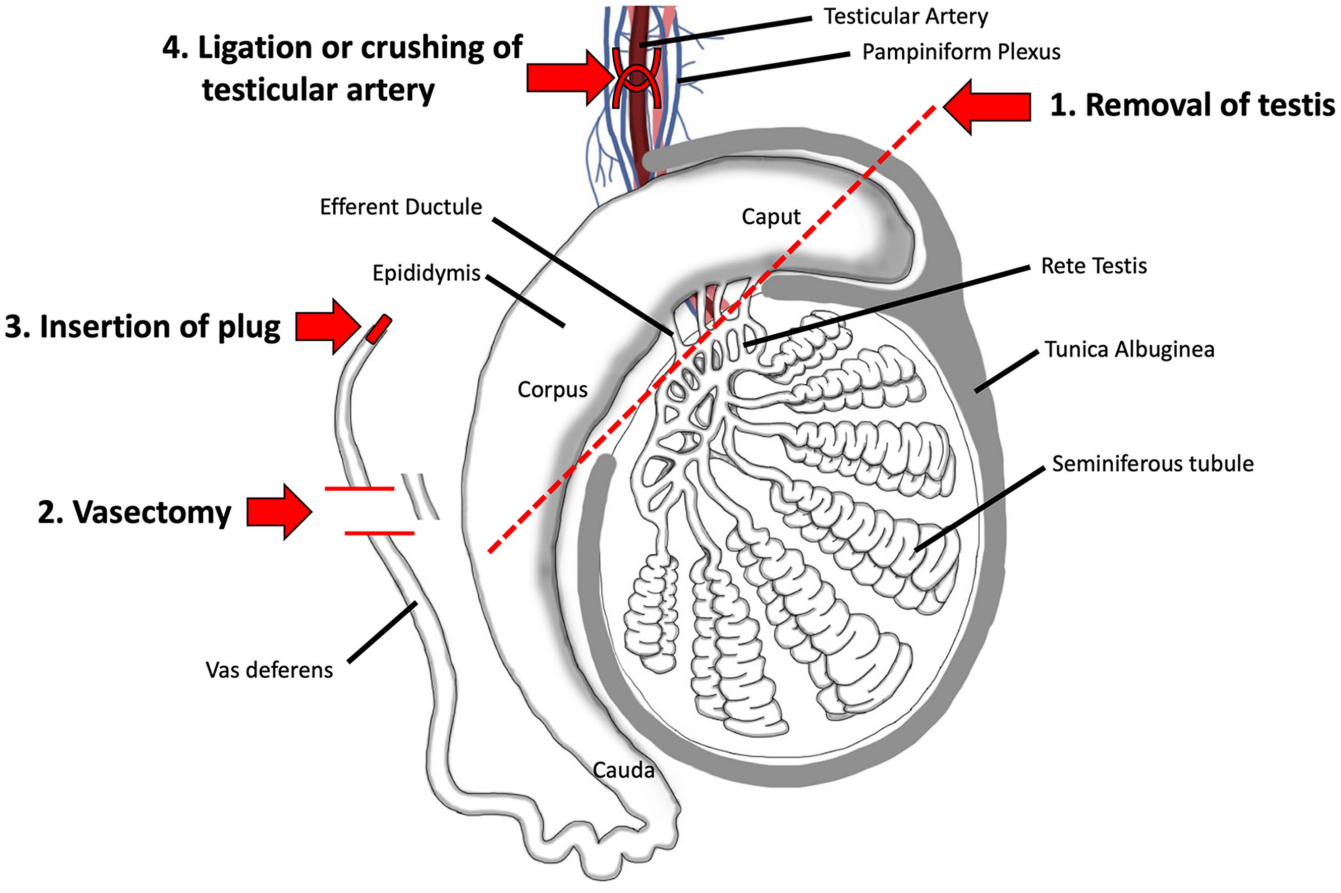
Surgical methods for sterilizing the male. 1. Surgical removal of the testis by castration is the oldest method and remains the most reliable even today. 2. Vasectomy, which involves ligation and removal of a section of the vas deferens, is a commonly used method in humans and has been proposed as a gonad-sparing method in domesticated animals when it is desirable to maintain androgen output by the testis. 3. Insertion of a plug, made of polyurethane or other types of polymers, has been tested in humans. 4. Surgical ligation of the testicular artery has been proposed as a way to induce testicular atrophy, without complete removal of the organ. Adapted and modified from Biorender.

There are four main procedures for surgical sterilization: physical removal of the gonad (castration or gonadectomy), gonad-sparing by cutting the vas deferens (vasectomy) or insertion of a plug, and ligation of the testicular artery (Figure 3).

#### 3.1.1 Removal of testis

Surgical castration, recently also called desexing (152), has been used for thousands of years as a method of sterilization in numerous animal species (7). In dogs and cats, it has become the method of choice for inhibiting male reproduction to mitigate overpopulation, especially in free-roaming animals (153–156). Castration provides the added benefit of removing the major source of testosterone production and thus helps to control sexual and aggressive behaviors and androgen-associated diseases, such as prostate enlargement (benign prostatic hyperplasia) and testicular neoplasia (30, 157, 158). Some studies have reported a 13.8% increase in life span for gonadectomy in male dogs possibly due to a shift in the causes associated with death and in animal behavior (23, 24). However, others have indicated that when accounting for the age at castration, early testis removal reduces the lifespan at least in some dog breeds (25). Some specific cancers, such as the osteosarcoma, are more common in neuter that intact male dogs (159). Moreover, neutering leads to persistent supraphysiological levels of LH, which may affect multiple organs that express LH receptors (159). Although the mechanism for these phenomena has not yet been firmly established, considerable attention is being paid to help the owners determine the optimal age for gonad removal in each dog breed (23, 30, 160, 161).

Castration is also used in large animal production with the main purpose of suppressing the production of androgens, such as in beef cattle (162, 163) and pigs (27, 164), but is also called ‘physical castration’ in bulls because it can be performed in a rather crude manner without anesthesia. For example, one method uses an elastic band to constrict the blood supply causing necrosis (165) but a method called Burdizzo castration involves crushing the spermatic cord of each testis within the scrotum (163). The benefit of surgical castration in pigs has been recognized for centuries, not only for controlling the presence of ‘boar taint’ in pork but also for reducing male aggression on large commercial farms (27, 166–168). Castration in dogs and cats is performed under strict regulations by licensed veterinarians using either general anesthesia or sedation and local anesthetics (153, 169). However, male pigs are typically castrated soon after birth without anesthesia (27), and calves are castrated with or without local anesthesia (162). Significant concern has been raised over the resulting pain experienced by the young pigs, cattle, and horses (27, 164, 170, 171). However, general anesthesia would be too costly on the farms and still not account for post-operative pain. Various methods for pain reduction have been discussed (168, 172), and alternatives to surgical removal of the testes, such as immunocastration, have been proposed and tested (173–175). In the horse, castration is reportedly best done in a standing position, with induced sedation and local analgesia. Although fairly routine in practice, the risk of complications is not trivial, with edema, infection, and hemorrhage being the most frequently reported problems (171, 176–178).

While castration helps to address animal welfare problems created by overpopulation of certain animal groups, especially among free-roaming dogs and cats (179, 180), there is now considerable push-back on the continued use of this method of sterilization, and several reviews have examined alternative methods (20, 21, 154, 181–188). Animal welfare concerns are repeatedly being raised regarding the associated risks of pain, stress, and post-surgical complications (163, 184, 189–191). Such issues have led to the proposal of numerous alternatives and potentially more humane methods, especially for raising male pigs (168, 191–195).

#### 3.1.2 Vasectomy

Vasectomy involves surgical isolation of the vas deferens, ligation in two areas, and then removal of the ligated region. This is also called gonad-sparing because it preserves intact testes and their ability to produce hormones (3). The method is performed under general anesthesia, typically involving a simple incision of the scrotum in men and inguinal incision in dogs and cats (153, 158). Vasectomy is one of the most common, convenient, and effective methods of sterilization/contraception in men, with nearly 500,000 being performed annually in the United States (196, 197). Although vasectomy is considered safe and reliable, a few complications have been shown to arise post-surgery, such as infections, chronic scrotal pain, and sperm granulomas (198). Vasectomy was first explored in dogs and then attempted in men in the late 1890s (199, 200).

In dogs, vasectomy is becoming a popular choice as a form of gonad-sparing surgery, with some evidence suggesting better health and behavior outcomes than testis removal, despite not preventing reproductive diseases, such as prostate enlargement (2). However, in one experimental study with dogs, vasectomy was shown to induce testicular damage and thus may not be reversible (201). A new technique called ‘laparoscopic castration’ and ‘vasoligation’ was introduced for use in dogs, whereby ligation or fusion of the tissues is performed on the vas deferens and/or the testicular blood vessels, without removing the testes (202–204). This method caused total testicular atrophy and epididymal fibrosis, with testosterone concentrations equivalent to that after surgical castration. Another method is simply to induce blockage of the vas deferens, without ligation, by injecting substances directly into the lumen (as discussed in section 3.3.2).

Testicular effects after vasectomy appear to be dependent on two events: increased pressure and expansion of the luminal contents into the head of the epididymis (205) and/or formation of sperm granulomas (198, 206). While some studies have reported no swelling or back-pressure in the testis (as sperm and fluid continue to be produced by the seminiferous epithelium), they did observe degenerative changes in the seminiferous epithelium leading to atrophy. An immune reaction within the testis and formation of antibodies to sperm would induce testicular lesions without back-pressure being the cause (207–209). In most cases, it is thought that the formation of sperm granulomas in the epididymis will help to prevent swelling of the testis by reabsorbing the build-up of fluids and sperm after vasectomy. The immune response and the potential for testicular orchitis depend on a leukocyte (regulatory T-cell) cell response, which can lead to a prolonged tolerance state in mice (210). However, the formation of circulating antibodies is quite variable, with approximately 7–30% of vasectomized men showing anti-sperm autoantibody production (198). Vasectomy in immature dogs caused a delay in testis maturation but had no effect on testosterone concentrations after either immature or adult vasectomies, although some dogs showed significant pathology in the testes (211).

In summary, surgical castration is the predominant method for sterilizing animals, whereas vasectomy is more commonly utilized in humans. The advantage of surgically removing the testes in animals lies in the established techniques for the swift cessation of both sperm production and the primary secretion of testosterone, resulting in permanent sterilization. Surgical excision of any component of the male reproductive tract can induce sterility; however, its application and utility will be context-driven. Traditionally, veterinarians and pet owners have favored castration as a form of inducing sterility while decreasing sexual behaviors, which necessitates either the suppression of Leydig cell activity or the complete excision of these androgen-producing cells. However, gonad-sparing surgery is gaining popularity, especially among dog breeders, as increasing evidence suggests health benefits from maintaining reproductive steroid production at least in young animals. In contrast, vasectomy is not a feasible alternative to castration in farm animals due to its time-consuming nature and the skill required. Therefore, its use is limited to specific situations, such as producing teaser bulls for estrus detection.

### 3.2 Systemic chemical sterilization

The idea of using chemicals or drugs to induce male sterility was originated from toxicology studies of chemical compounds, some of which were identified as chemosterilants (36). For example, exposure to highly toxic environmental chemicals such as cadmium resulted in the complete destruction of the seminiferous epithelium (212). However, these chemicals can induce systemic problems, such as neurotoxicity (213), limiting their specific application for inducing sterility, especially in larger mammals. However, this category of sterilization has a significant application for use in smaller animals, especially rodent pests, in which death of the animal is not of significant concern (38, 214–217). While hundreds of chemicals have demonstrated toxicity to the male reproductive system or act as inhibitors of specific pathways crucial for spermatogenesis, as observed in endocrine-disrupting compounds (218–222), only a few have been proposed for potential use as male sterilants or long-term contraception in domesticated species [see the following reviews (45, 223, 224)]. A few examples are discussed here but others are presented in Table 1.

**Table 1:** Systemic non-hormonal chemicals that induce potential sterilizing effects in male animals.^1^

Target organ	Admin^2^	Chemical	Trade name^3^	Species	Dosage	Original use	Short-term pathology	Long-term pathology	Mechanisms of action	T decreased^3^	Inflammation	Side-effects
Testis	Oral, s.c.	Ketoconazole	Nizoral	Mouse, Rat, Dog, Monkey	10–300 mg/kg	Imidazole antifungal drug	Germ cell apoptosis	Decrease testis wt; germ cell loss; testicular atrophy; azoospermia	Inhibition of cytochrome p450 enzymes, blocking steroidogenesis; reduces testosterone	Yes	Yes	Inhibits steroid synthesis; central nervous system toxicity; adrenal insufficiency
		Embelin	N/A	Dog, Rat, Mouse	20–80 mg/kg	Anti-cancer	Reversible infertility; decrease T	Spermatogenesis arrest; decrease testis wt	Inhibition of energy metabolism and sperm motility	Yes	No	None
		Dibromochloro-propane	N/A	Rat	50 mg/kg	Nematicide	Multinucleated giant cells; sperm granuloma	Azoospermia	Inhibit sperm glucose metabolism	No	No	Damage to liver, kidneys, stomach
		4-Vinylcyclo-hexene diepoxide	N/A	Rat	100–500 mg/kg	Industrial chemical	Decrease sperm count; decrease T	Testis necrosis	Induces oxidative stress	Yes	Yes	Liver, kidney, stomach, and brain toxicity
		20,25-diazacholesterol dihydrochloride	DiazaCon™	Rat, Mouse, Bird, Squirrel	8–100 mg/kg	Lower serum cholesterol	Decrease T; germ cell loss; multinucleated giant cells	Leydig cell degeneration; testis atrophy	Inhibits steroidogenesis	Yes	No	Inhibits synthesis of glucocorticoids
		Quinestrol and Levonorgestrel	EP-1	Rat, wild rodent	0.33–0.67 mg/kg	Female contraceptive; synthetic hormones	Decreased epididymal weight; decreased cauda sperm number; germ cell loss	Testis recovers	Inhibits HPG axis	No	Not determined	Increased weight adrenal gland
Testicular and epididymis	Oral, s.c.	Methyl 1-(butylcarbamoyl)-2-benzimidazole-carbamate	Benomyl (Benlate) Carbendazim	Rat	25–500 mg/kg	Fungicides	Increase testis wt; sloughing germ cells; inhibit mitosis; occlusion of efferent ducts	Testis atrophy; decrease testis wt; fibrosis of efferent ducts; azoospermia	Inhibits microtubule polymerization; occlusion of efferent ducts	No	Yes	Hepatotoxicity; genotoxicity; fetal malformations
		Lonidamine derivatives	Gamendazole Adjudin	Rat, Mouse, Monkey, Dog, Rabbit	25–100 mg/kg	Anti-cancer	Sloughing germ cells	Testis atrophy; azoospermia	Disruption of Sertoli cell junctions; occlusion of efferent ducts;	No	Yes	Reduced motor activity, palprebral ptosis, lacrimation, tremors, and dyspnea
Epididymis	Oral, s.c.	3-Chloro-1,2-propanediol	α-Chlorohydrin Epibloc®	Rat, Mouse, Hamster, Dog, Guineapig, Sheep, Boar, Monkey	10–140 mg/kg	Organic chemical food contaminant	Immobilization of sperm	Testis atrophy; necrosis; azoospermia	Occlusion of efferent ducts; formation spermatoceles; inhibits epididymal function; inhibits glycolysis	No	Yes	High doses are neurotoxic and nephrotoxic

^1^For supporting references see: Supplementary Table S1 References. ^2^Administration of the chemical/drug; oral, subcutaneous, or cutaneous (s.c.). ^3^N/A, not applicable; T, testosterone.

*Ketoconazole (Nizoral)* is a good example of a chemical that was developed for therapeutic use but later explored for its potential as a male sterilant or contraceptive. It was initially developed as an imidazole antifungal drug for humans (225, 226), as well as for use in dogs, cats, and other animals (227–229). Its mode of action involves inhibiting ergosterol biosynthesis and disrupting membrane lipids in fungi (229). Further research revealed its ability to inhibit cytochrome p450 enzymes in mammals, to impede steroid synthesis (230). Its inhibitory effect on androgen synthesis (231, 232) raised the possibility that a derivative could be used as a male contraceptive (233), as well as a therapeutic method to suppress gonadal and adrenal hormone synthesis for treatment of prostate cancer and Cushing’s syndrome (230, 234).

Ketoconazole demonstrated the ability to inhibit sperm motility in dogs, primates, and humans, following oral administration at the optimum dose (233, 235–237). Most importantly, the drug inhibited testosterone synthesis and interstitial fluid production directly in the testis, without inhibiting pituitary function (238, 239). In contrast, other imidazole compounds were shown to first decrease LH, which indirectly decreased testosterone. At higher dosages, ketoconazole induced germ cell apoptosis, producing oligospermia and azoospermia, and lead to a reduction in testicular weight with partial atrophy. Additionally, it caused decreases in epididymis and ventral prostate weights due to steroid synthesis inhibition (237). Male sterility was rapidly achieved, as treatments resulted in infertility within 3 days, using 200–400 mg/kg in rats (236). However, the adverse effects included not only central nervous system toxicity but also adrenal insufficiency, with decreased adrenal corticosteroid production and could lead to death (236, 240). Therefore, analogs of ketoconazole were synthesized hoping to reduce toxicity and be potent spermicides (236). Ultimately, none of these compounds have been marketed for human contraception, possibly due to their potential interaction with the oral contraceptive pill (241). While the drug is used in dogs as an antifungal, it has not been approved for inhibiting reproduction in domestic animals.

*4-Vinylcyclohexene diepoxide* (*VCD*), a metabolite of vinyl cyclohexene (VCH), is an industrial chemical that is used as a diluent in the production of epoxides, epoxy resins, plastics, rubber, and pesticides (242, 243). During the study of its potential toxicity to human exposure, it was discovered that VCD was a reproductive toxicant in both males and females (243). In the male, VCD caused germ cell degeneration (244) and was later proposed as a non-surgical contraceptive or sterilant in dogs and cats, especially for the female (245–248). However, its systemic toxicity, particularly its potential negative effects on the brain (249), was a major concern and limited its further development. Nevertheless, VCD is an example of careful targeting of a chemical for sterilization in a species where general toxicity or even death is of less concern, as long as the compound induces male sterility. With further development, the testicular toxicant triptolide (250) was added to VCD and marketed as ContraPest® (251–254). This commercial product is approved by the US Environmental Protection Agency (EPA) as a rodenticide, but unfortunately the doses used will not achieve sterility and the rodents must receive continual exposure for sustained infertility (217).

*20,25-diazacholesterol dihydrochloride* was first developed by G.D. Searle, as a potential drug for lowering serum cholesterol levels in humans (255, 256). Subsequent studies of this drug found secondary effects on reproduction, which made it a potential reproductive toxicant in several species (257–259). The compound inhibits side-chain cleavage of cholesterol, thus reducing the synthesis of reproductive steroid hormones by blocking the conversion of cholesterol to pregnenolone (260). In male birds and rodents, this chemical decreased testosterone levels, caused the loss of germ cells, induced seminiferous tubule atrophy, and resulted in the degeneration of Leydig cells (257–259, 261). The compound was given the trademark DiazaCon™ and was marketed first as Ornitrol to control bird populations (260, 262). However, at higher doses, there were non-reproductive health effects and, in one study, caused the death of two birds (259). It has also been used in the control of other mammalian wildlife, including the prairie dog (263, 264) and grey squirrels (265–267). However, DiazaCon is no longer registered for use in the USA.

Some chemicals show broad-spectrum effects due to their mechanistic actions that disrupt biochemical pathways in both the testes and the male reproductive tract (Table 1). Two examples are the benzimidazole-carbamate compounds and lonidamine. Because the outcome depends on dose and time of exposure, the interpretation of the mechanisms leading to infertility has proven to be difficult. At the lower dosages, these chemicals tend to have direct effects on the seminiferous epithelium, such as disruption of Sertoli cell function, sloughing of germ cells, and disruption of the BTB, while at the higher doses, the overwhelming effects can be found in the reproductive tract, such as ciliostasis and occlusion of efferent ductules, inflammation, and fibrosis, which ultimately lead to testicular atrophy.

*Benomyl (BEN) and Carbendazim (CBD)* (methyl 1-(butylcarbamoyl)-2-benzimidazole-carbamate) are systemic fungicides used for the application on plants. BEN is rapidly degraded in water to form CBD. The fungicidal effects are mediated by the binding of the compounds to β-tubulin thereby causing depolymerization of microtubules, which disrupts the formation of the mitotic spindle, thus blocking cell division (268). Because mitosis is essential in both plants and mammalian cells, the potential toxicological effects of this class of compounds was carefully tested for reproductive toxicity in rats (269).

Early studies found that these compounds induced infertility by decreasing cauda epididymal sperm counts and producing testicular atrophy (269–273). These effects were dependent on age at exposure, dosage, and duration of exposure. First, the focus was on the microtubule effects, as cell proliferation in the seminiferous epithelium was disrupted, and there was massive sloughing of germ cells into the lumen (272). However, subsequent experiments showed that these initial testicular effects were rapid, happening within minutes after exposure (274–277), while simultaneous pathological changes were occurring downstream in the efferent ductules, where the lumens became occluded. The ductal effect blocked the passage with compacted sperm, indicating that treatment had interfered with fluid reabsorption (277–280). An increased rate of fluid reabsorption would cause an over-concentration of the sperm, creating a luminal plug and thereby inhibiting sperm transport into the epididymis. This pathological response has now been shown to occur after inhibiting motile cilia in the efferent ducts (135, 281). BEN has been demonstrated to induce ciliostasis also in the trachea (282). Ultimately, the obstruction of efferent ductules results in the accumulation of sperm and fluid in the lumen, leading to back-pressure in the testis and dilation of rete testis chambers and seminiferous tubules. Over time, the testis weight nearly doubles until it peaks, followed by a gradual regression until total atrophy, resulting in sterility. This pathological mechanism is common to several animal species (133). However, its ability to induce male sterility appears to be limited to the rat species and is dependent on the induction of a rapid, strong inflammatory response leading to permanent occlusion by fibrosis (283). However, these compounds have never been developed as rodenticides.

*Lonidamine* is an indazole carboxylic acid derivative that produces male sterility through a mechanism similar to the benzimidazole-carbamate compounds. This chemical was first developed for the treatment of cancer (284) and later identified as an anti-spermatogenesis compound (285). As an anti-cancer agent, it inhibited cellular energy metabolism (286, 287) and showed no toxicity in rats, monkeys, and dogs at low doses. However, at higher dosages, the compound had severe effects on the testis (288, 289), which were similar to BEN and CBD, as it produced Sertoli cell effects in the testis that resulted in the sloughing of immature germ cells (290–295), loss of epididymal sperm, and testicular atrophy (294, 296). Consequently, the potential use of lonidamine as a rodenticide by inducing sterility in male rats and mice was studied. Instead, at lower doses, the effects were primarily on Sertoli cells, causing disruption of the blood-testis-barrier (293, 297), and the effects were reversible. Therefore, lonidamine was studied as a male contraceptive under the names Gamendazole and Adjudin (107, 236, 286, 290, 293, 294, 298, 299).

Although this compound has received extensive attention for fertility control in pests, the mechanism that produces testicular atrophy and loss of epididymal sperm has not been completely elucidated. Data reported thus far point to an occlusion of the efferent ductules, although histopathology of these ductules has never been evaluated. First, there is evidence that the seminiferous tubule may go through transient dilation (291) before atrophy (294, 296). Second, high-dose treatments resulted in the loss of epididymal sperm (288). Finally, lonidamine inhibited the cystic fibrosis transmembrane conductance regulator (CFTR) in the epididymis (300, 301) and, like the benzimidazole-carbamate chemicals, inhibited microtubule polymerization (302). CFTR is highly expressed in the efferent ductule epithelium and epididymis (303–306), and downregulation of CFTR is associated with ductal occlusions (304, 307). Thus, lonidamine-induced sterility in rodents appears to involve reproductive tract occlusions and testicular back-pressure atrophy, similar to BEN and CBD.

*α-Chlorohydrin* (*3-Chloro-1,2-propanediol*) is a chemical solvent that also targets the efferent ductules. This compound is classified as a chemosterilant (308) and registered with the EPA as a rodenticide. It is marketed under the name Epibloc® (Reg. No. 42882–2), but despite its application in rodents (309), the observed toxicity found in non-reproductive organs at higher doses (310–312) has precluded its further development as a contraceptive or a sterilant in larger mammals. Anti-fertility activity of α-chlorohydrin was discovered in the 1960s through compound screening for activity on post-testicular spermatozoa (313). At first, it appeared that the chemical might be the perfect male reversible contraceptive because it was shown to act directly on sperm within the epididymis by inhibiting sperm motility and thus fertilizing capability and was effective in a number of species (310, 314–316). Subsequent studies revealed that low doses of α-chlorohydrin inhibited glyceraldehyde-3-phosphate dehydrogenase activity, which caused the dramatic decrease in motility (311, 317–321). The effect on sperm was rapid and direct (311, 322), but other effects were also found in the epididymis such as inhibition of fluid reabsorption in the cauda region, where sodium and water transport were inhibited by 50% (323, 324). Moreover, sugar transport across the epithelium of the rat caput epididymides was disrupted (325).

While lower doses of α-chlorohydrin had low toxicity, they only produced a contraceptive effect and no sterility. However, it was discovered that a single high-dose administration in the rat could make the males permanently infertile (41, 311, 314, 326–329). The higher doses caused rapid formation of spermatoceles and sperm granulomas, which at first were thought to be located in the caput epididymis, but further study revealed the lesions to be in the efferent ductules and initial segment of the epididymis (327, 328). These lesions near the testis were found to cause complete blockage of the reproductive tract, ultimately resulting in sterility (311, 327, 329). This pathology was similar to that observed with the benzimidazole-carbamate compounds and lonidamine, as any occlusion of the efferent ductules can cause fluid accumulation in the testis and produce atrophy (133). However, it was found that the high doses also led to neurological and kidney damage (311).

In summary, systemic exposure to chemicals for the purpose of inducing male sterility is basically an offshoot of toxicology studies. Although hundreds of chemicals have shown detrimental effects on male reproduction, only a few have given promise for clinical use to inhibit male fertility in domesticated species. Most efforts were initiated as potential ways of inducing contraception in men, but testing in a wide range of animals found that sterility could also be induced at higher dosages. However, the potential toxicity displayed in other organ systems has drastically limited the application of these technologies for contraception in men and companion animals. Noticeably, because toxicity in organs outside the reproductive system is not a limiting factor for use in controlling pest animals, it has permitted those chemicals to be developed into rodenticides. Regarding farm animals, because most of the tested chemicals are classified as having systemic and reproductive toxicity in humans, their use has not been considered or approved due to food safety concerns.

### 3.3 Chemical castration

Chemical sterilization has primarily been focused on the testis, but some treatments affect both the testis and epididymis, while others target only the epididymis and/or vas deferens. Treatments have included a wide variety of chemical classes and methods of delivery, including organ injections (testis, epididymis, and vas deferens), oral dosing, and subcutaneous or intramuscular injections. Some chemicals are effective after a single dose, but others require multiple treatments. The mechanism of action for each chemical depends on the primary cellular target within the organ and physiochemical pathways involved, as well as the delivery method (Tables 2, 3). A large number of chemicals given by direct injection into the reproductive organs have been studied for inducing infertility, but most of these fall under the classification of ‘sclerosing agents’. This category is based on the typical reactions observed in tissues following treatment, which include necrosis (death of tissue), inflammation, fibrosis (an increase in connective tissue), formation of granulomas, and obstruction of the tubular lumens.

**Table 2:** Chemical castration by intratesticular administration and potential sterilizing effects in male animals.^1^

Target organ	Admin^2^	Chemical^3^	Trade name^5^	Species	Dosage	Original use	Short-term pathology	Long-term pathology	Mechanisms of action	T Decreased^5^	Inflammation	Side-effects
Testis	Intra-testicular^2^	Zinc compounds	Kastrin, Neutersol™, Zeuterin™, EsterilSol™, Testoblock®, Infertile®	Rat, Dog, Cat, Monkey, Bear, Pig	2.6–58 mg/mL	Dietary supplement; treatment of common cold	Multinucleated giant cells; acute inflammation; edema; testis swelling	Seminiferous tubule necrosis; atrophy;	Sertoli cell barrier damage; massive neutrophilic inflammation; necrosis; tubular fibrosis	Yes	Yes	Scrotal ulceration and dermatitis; necrotizing reactions
		4-allyl-2-methoxyphenol	Eugenol Clove oil	Dog	1 mL	Cosmetics; anti-anthelmintic; expectorant	Testis swelling	Seminiferous tubular fibrosis	Testicular necrosis; inflammation	Yes	Yes	Epididymal vacuolation
		CaCl_2_ ± ethanol & hypertonic NaCl	Calchlorin™, Salts	Rat, Dog, Cat, Bull, Goat, Donkey, Ram, Guinea pig, Buffalo	20–50% in water or ethanol	Inorganic salt; food additive; deicing salt; desiccant	Testis swelling; inflammation	Testicular atrophy	Testicular dehydration; necrosis; coagulative fibrosis	Yes	Yes	Scrotal necrosis if leakage
		CdCl_2_	Metallic salt	Rat, Mouse, Dog, Rabbit, Hamster	150 μ/kg	Inorganic salt pigment	Hemorrhage; edema; inflammation	Testicular atrophy	Decreased blood flow; ischemia; oxidative stress	Yes	Yes	Toxic to numerous organs, if given oral
		1,2,3-trlhydroxypropane	Glycerol	Rat, Dog, Cat, Monkey, Rabbit	10–70%	Food additive; soap; laxative	Seminiferous tubule disruption	Testicular atrophy	Hyperosmotic dehydration	Yes	Yes	None
		Lactic acid	Chem-Cast®	Rat, Dog, Bull	85–92%	Organic synthesis; food additive	Germ cell degeneration	Testicular atrophy	Sclerosing, caustic chemical; necrosis; fibrosis	Yes	Yes	Scrotal necrosis if leakage
		KMnO_4_	N/A	Pig	0.25 g/17 mL acetic acid	Inorganic oxidizing agent; water treatment	N/A	Seminiferous tubular atrophy; fibrosis efferent ducts	Sclerosing, caustic chemical	N/A	Yes	Leydig cell hypertrophy
		Metallic & rare earth salts	N/A	Rat, Mouse, Monkey	0.02–0.08 mM/kg	Salts	Focal necrosis	Testicular total necrosis for some salts	Cytolysis, ischemia, necrosis	N/A	Yes	Some Leydig cell loss; early effects on vas deferens sperm; some salts showed no effect
		Ethanol	Alcohol	Bull	100%; 10 mL	Organic solvent	Testis swelling; necrosis	Testicular atrophy	Necrosis; inflammation; edema; fibrosis	Yes	Yes	Only 50% became infertile
	Intra- Testicular; s.c or i.m	Inflammatory induction agents^4^	N/A	Guinea pig, Monkey, Rat, Rabbit; Dog; Ram	N/A	Antibody stimulating	Inflammation; seminiferous tubular dilation; testis swelling	Granuloma; necrosis; testicular atrophy	Inflammation;	No	Yes	Injection outside testis required for azoospermia for some agents

^1^For supporting references see: Supplementary Table S2 References. ^2^Administration of the chemical/drug directly into the testis; subcutaneous or cutaneous (s.c.), intramuscular (i.m.). ^3^Some of these are considered to be sclerosing agents and have also been tested as intra-epididymal sterilants. ^4^Testicular antigens ± killed bacteria; killed mycobacteria; Freund’s complete adjuvant injected into foot after intratesticular injection of turpentine; Freund’s complete adjuvant plus testis homogenate. ^5^N/A, not applicable; T, testosterone.

**Table 3:** Chemical castration by injection of non-hormonal chemicals into the reproductive tract and potential sterilizing effects in male animals.^1^

Target organ	Admin^2^	Chemical	Trade name^4^	Species	Dosage	Original use	Short-term pathology	Long-term pathology	Mechanisms of action	T decreased^4^	Inflammation	Side-effects
Epididymis Vas deferens	Intra-lumen	Sclerosing agents^3^	N/A	Ram, Goat, Dog, Cat, Rat, Mouse, Monkey	Wide range	Numerous chemicals	Inflammation; epididymis swollen	Granulomas and cysts; tissue scarring; azoospermia	Spermatic granuloma; fibrosis; blockage	No	Yes	Adhesions between epididymis and tunica vaginalis; sometimes testicular effects
	Intra-lumen plugs	Silicone, polyurethane elastomers, hydrogels, co-polymers	RISUG® ADAM™ Vasalgel®	Rat, Rabbit, Monkey, Man	N/A	Elastomers; gels	Occlusion of vas deferens	Azoospermia; testis and epididymal pathology	Prevention of sperm transport; blockage	No	Yes	Testis focal degeneration; hematoma

^1^For supporting references see: Supplementary Table S3 References. ^2^Administration of the chemical/drug directly into the epididymis or vas deferens. ^3^Sclerosing agents consist of a broad range of caustic chemicals that induce tissue damage and elicit fibrosis: Quinacrine dihydrochloride, zinc arginate, sodium tetradecyl sulfate, potassium permanganate, ethanol ±ascorbic acid, silver nitrate, formaldehyde, styrene maleic anhydride, chlorhexidine digluconate, lactic acid, methylcyanoacrylate, urea and ethanol, formaldehyde and ethanol, CaCl_2_ in ethanol. ^4^N/A, not applicable; T, testosterone.

#### 3.3.1 Testicular injection

Direct injection of chemicals into the testis to induce sterility was begun as a method for potentially by-passing adverse systemic reactions that would typically be observed in other organs if a chemical was taken orally or by subcutaneous or intramuscular injection (Table 2). Treatment by direct injection into the testis could theoretically reduce the effective dosage of a compound. For example, testicular damage with subcutaneous injections of CaCl_2_ required approximately 2.5 mg/kg dosage, whereas direct injection of 0.15 mg/kg into the testis was sufficient to induce testicular atrophy (330). Intra-testicular injections have also been used in toxicology studies because systemic exposures may inhibit pathways that indirectly damage the testis or induce death before specific effects on testicular cells can be analyzed (331).

A point to consider is the potential effects of the injection itself. Russell et al. (331) found that the injection of any volume greater than 50 μL caused testicular swelling and an increase in turgidity of the testis in rats and volumes of greater than 75 μL could cause fluid back-flow within the testis. There was no investigation of the volume required to induce damage due to increased pressure alone; however, it is well recognized that back-pressure swelling of the testis can induce rapid damage to the seminiferous epithelium (132, 133). Because many of the studies listed involved the use of high volumes (as high as 500 μL in the rat), caution is warranted when trying to interpret the dosages given in each case. An additional consideration is that while the injected substances were observed to disseminate rapidly throughout the testis within a few hours, the effects observed were less severe in regions further from the site of injection. Some of the chemicals presented in Table 2 are capable of inducing total testicular atrophy, but the mechanisms of action are not fully understood. Some responses are due to a direct action on cellular pathways within the organ, while indirect effects can be linked to several causes, including the following: (a) general alterations in blood flow (by coagulative necrosis), (b) dehydration of tissues by hypertonic solutions, (c) induction of tissue damage and fibrosis due to the caustic nature of the chemicals, and (d) severe inflammation (linked to several responses).

Although some compounds have shown remarkable success for inducing sterility in the male, they reached clinical trials rarely and only one received approval for use in animals by the Food and Drug Administration (FDA) in the US (332). Included in this section are some of the chemicals that have been tested with this mode of delivery: zinc compounds and hypertonic salt solutions.

##### 3.3.1.1 Zinc compounds

Of the chemicals studied for testicular injection, only zinc compounds have received FDA approval for use in animals, specifically in the male dog (332). Although these compounds were initially developed for other purposes, such as dietary supplements and the treatment of zinc deficiency, researchers began experimenting using direct injection into the testis and the reproductive tract, as a substitute for castration. The first compound tested was zinc tannate and was called Kastrin (42, 333), which produced considerable variation in testosterone levels and testicular pathology (334). This led to the development and use of the FDA approved, pH-neutralized zinc gluconate (335), which was marketed by Pet Healthcare International, Inc. (Columbia, MO, USA) as Neutersol® (336). Ark Sciences, Inc. rebranded the compound as Zeuterin™ in 2014. Although it was marketed heavily in the US, with specific training of veterinarians on the correct procedure for injection, and even sold outside the US under the trade name EsterilSol™ (337, 338), in 2015, the trademarks for these products were abandoned. Similar zinc gluconate sterilants have been launched in Brazil under the names Infertile® (RhobiPharma Ind. Farm., Hortolândia, SP, BR) (339, 340) and Testoblock® (BioRelease Technologies LCC, Birmingham, AL, USA) (341–344). Overall, this approach has not received a broad market acceptance due to inconsistencies in results and concerns regarding animal welfare.

Testicular injection of zinc compounds produced sterility primarily in pubertal and adult dogs but was also found to work in prepubertal puppies (345). However, the observed pathology showed inconsistent results, which included testicular swelling, scrotal ulceration, and necrotizing orchitis (332, 334, 345–348). Effects on testosterone concentrations were also inconsistent (337, 344, 345, 348–350). The primary purpose of using the zinc compounds was to induce complete testicular atrophy in the adult dog, with a corresponding reduction in testosterone, as would occur following surgical castration. The inconsistent results that were obtained led others to test several types of modifications, such as adding DMSO (dimethyl sulfoxide) to the zinc gluconate solution, as a way to increase dispersion of the solution throughout the testis. This along with using two injections instead of one resulted in a more severe histopathological response and a highly significant reduction in testosterone levels (351–353). This method has also been tested in cats (151, 341, 342), bulls (338), monkeys (348), and bears (354) but with limited success in these species.

In some cases, a transient swelling of the testis with dilation of seminiferous tubules was observed. This suggested that in some animals, the injections may have been displaced, causing either a disturbance in blood flow, acute inflammation, and/or failure of fluid reabsorption in the efferent ductules (132, 133). Although an investigation of the efferent ductules has never been reported, in 2009, another zinc gluconate patent was published in which more specific instructions were given for administration. These included direct injection into the rete testis/efferent ductule region, specifically to cause blockage of the reproductive tract, which was claimed to induce sterility without affecting testosterone production. The stated goal of this *refined* approach was for use in free-roaming dogs, to allow the introduction of treated dogs back into the feral population for breeding competition, which would not occur if the males lost androgen stimulation (355).

An important pathological response common to zinc gluconate and other compounds has been an acute, massive inflammatory reaction, leading to local edema, hemorrhage, vascular degeneration, swelling of the testis, and necrosis, with evidence of direct effects on Sertoli cells and degeneration of the blood–testis barrier (334, 338, 342, 343, 345, 346, 349, 350). These reactions have led to considerable debate on whether such intense pathology is required for zinc gluconate to induce sterility. To test this hypothesis, zinc gluconate injections were co-treated with anti-inflammatory drugs, primarily COX-2 inhibitors and the glucocorticoid dexamethasone (343, 350). Surprisingly, the anti-inflammatory drugs did not interfere with the sterilizing action of the zinc compounds (343, 350). However, the inflammatory responses were similar to those observed with autoimmune orchitis in dogs, which included lymphocytic infiltration associated with disruption of the blood–testis barrier (356). The major health concern appears to be testicular swelling, followed by scrotal ulceration and dermatitis and, in some cases, necrotizing orchitis (223, 338, 347, 357, 358). The occurrence of necrotizing reactions ranged from 3 to 38% (223, 359), leaving some to speculate that the severity of treatment was dependent on the injection technique (346). Another hypothesis was that the injections had induced vascular injury, along with scrotal ulcers, edema, hemorrhage, and tissue granulation (338). The failure to uncover precise mechanisms involved in testicular atrophy and the inability to control those pathways for consistency, together with concerns regarding animal welfare, led to the ultimate removal of zinc gluconate as a dog sterilant from the USA market.

##### 3.3.1.2 Hypertonic salt solutions

Salt solutions can be hypertonic to body tissues and have been explored in various species as male sterilants using direct testicular injection. The idea came from the injection of hypertonic solutions into tumors and cysts (360). CaCl_2_ is one of the salts that has been around the longest (361) and happens to be non-toxic inexpensive and simple to formulate. Treatments have varied from a 5% solution to 75%, the maximum solubility in water (75 g/100 mL), as well as addition to 95% ethanol. Injections have successfully induced testicular atrophy in rats (362–364), dogs (365–374), cats (46, 375, 376), bulls (162, 361, 377–379), goats (380), water buffaloes (381), and guinea pigs (382). Only in the donkey, the treatment was not recommended for sterilization (383); however, this could have been due to the use of a lower concentration (20%). The decrease in testosterone in the dog was shown to be dose-dependent, with 30% CaCl_2_ providing azoospermia (367). In the cat, 20% CaCl_2_ with 0.5% DMSO or 95% ethanol resulted in azoospermia (46, 368). Although a 20% solution was effective in dogs, it appears that in larger mammals, up to 30–50% was required for necrosis and total atrophy (162, 361, 377–379). Hypertonic NaCl solution has also been tested in numerous species (376, 384–390) but was less consistent than CaCl_2_. Although higher concentrations were required for effectiveness in some species, in the bull, even the higher concentration was less effective after 5 months of age (390).

Hypertonic salt solutions appear to have some advantages over zinc solutions. Contrary to zinc solutions, the salt solutions seem to be more consistent in causing the loss of Leydig cells, consequently decreasing serum testosterone and sexual behavior, while also destroying the seminiferous tubules. The pathological responses to intra-testicular injection of hypertonic salts are similar to those with zinc solutions, including tubular necrosis and inflammatory cell infiltration, which results in testicular atrophy and tubular calcification (389, 391). However, adverse effects are generally minimal, except at very high concentrations and also if there is leakage from the testicular capsule. The Parsemus Foundation (San Francisco, CA, US), which is dedicated to animal health and welfare, recommends the use of CaCl_2_ intra-testicular injections for sterilization in dogs and has obtained a trademark for Calchlorin™, but this inexpensive and non-proprietary sterilant has yet to receive regulatory approval (369).

#### 3.3.2 Injections into the epididymis and vas deferens

In addition to testicular injections, administering chemicals directly into the reproductive tract (Table 3) has also been tested as a replacement for surgical vasectomies (44, 392). Although vasectomy is minimally invasive and highly effective in dogs and cats, the injection of a chemical directly into the epididymis or vas deferens could be in principle more efficient and with less risk. Blockage of the reproductive tract has been achieved by two types of injections. The first involves injecting sclerosing chemicals that induce tissue scarring or hardening by non-specific, inflammatory fibrosis in the tubular wall, which causes luminal stenosis and obliteration (393). A wide range of sclerosing agents have been tested in several different species (Table 3). The second type of injection is the use of sealing agents that plug the vas deferens lumen.

Sclerosing agents have been clinically employed to induce fibrosis and eradication of varicose veins (394) and even epididymal cysts (395). Therefore, it is not surprising that their inflammatory properties could be used for chemical vasectomy and epididymal obstruction. Several studies have shown this method which is capable of inducing permanent blockage of the epididymal lumen, resulting in azoospermia (44, 396, 397). Many of the sclerosing chemicals are caustic or hypertonic to tissues and capable of producing permanent damage. However, the results have shown considerable variation between studies, depending on the species, dosage injected, and the technique used. Overall, the rodent species appear to be more susceptible to permanent blockage than others. In one study, ethanol induced only approximately 70% infertility in the rat (392), while two other studies resulted in 100% infertility (398, 399). Technical challenges, such as ensuring accurate injection into the vas deferens, have been reported (400), suggesting that administration may require a more experienced technician using echography guidance (373). Recanalization of the duct is another potential problem, as well as other unwarranted side effects including abscess formation, scrotal hematomas and sperm granulomas (43, 151, 223, 401). These problems and the inconsistency in larger mammals have reduced enthusiasm for its use, along with its failure to reduce testosterone levels and sexual behavior (151).

Non-sclerosing methods for occluding the vas deferens have also been developed using silicone, polyurethane elastomers, hydrogels, and various co-polymers. Several have been developed for human contraception, with trade names including, RISUG®, ADAM™, and Vasalgel® (4, 402). Some of these methods produce plugs that are reversible to some extent, but others produce sterility.

In summary, chemical castration is a term generally applied to the injection of a substance directly into the testis or reproductive tract. While chemical injections into the testis have never been proposed for use in men, this method has received huge support for inducing sterility in a variety of domesticated animals. The particular use of zinc compounds for injections received the greatest effort more recently, resulting in a product that was commercialized for use in dogs. Another promising method is the injection of hypertonic salt solutions into the testis, which appears to be as efficacious, if not better than the zinc compounds. However, these methods work by inducing an inflammatory reaction in the tissues, producing sclerosis, and possibly inhibiting blood flow, which results in tissue necrosis. The associated pain has discouraged their use in companion animals. An alternative method has been developed for humans, whereby the vas deferens is injected with a substance that results in plugging the lumen. Whether this could be developed for rapid use in pet animals would require considerable investigation. Regarding farm animals, in addition to concerns about animal welfare, the potential for chemical diffusion reaching muscle tissues and affecting food safety reduces the likelihood of these approaches being utilized in livestock.

### 3.4 Hormonal sterilization

Hormones, including steroids and synthetic peptides, have been explored for controlling male reproduction. However, the focus has been on reversible contraception for both men and domestic or wildlife animals (21, 156, 182, 197, 403–406) rather than inducing sterility. For instance, anabolic steroids such as testosterone undecanoate can be used alone or with a progestin to temporarily suppress gonadotropins, leading to azoospermia, or the absence of sperm. In general, this approach has not worked consistently and can result in undesirable side effects, including hypokalemia, hypokalemic periodic paralysis, depression, and reduced libido (5, 197). However, a synthetic progesterone with a testosterone derivative is being tested for contraception in men under the name DMAU (dimethandrolone undecanoate) with promising results (407).

GnRH agonists and antagonists, which suppress gonadotropin synthesis and secretion, have been widely tested in domestic and wildlife animals. GnRH agonists, such as deslorelin, have been used successfully for temporary inhibition of reproduction in both males and females (20, 92, 152, 156, 186, 408–415). Suprelorin® (Virbac) is commercially available in Australia, New Zealand, and Europe for contraception in male dogs. However, GnRH agonists may not work in all species (53), and so far, there has been no indication of producing sterility. In men, the results using this method have been too inconsistent to be relied on as a contraceptive. GnRH analogs have also been tested in neonatal and juvenile male animals (rodents, cats, dogs, and monkeys), with variable long-term results, but in each case, there was only a delay in the onset of puberty (51, 416–421). In one study following postnatal treatment with Deslorelin, 2 of 6 dogs experienced cryptorchidism, and at 108 weeks of age, testes histology had not returned to normal (419). These preliminary results raise the possibility that neonatal treatment with GnRH agonists might be adapted for induction of sterility in some species.

Sterility can occur with androgen treatments, as evidenced by cases of permanent infertility in adult males following unsupervised use. The best example is the use of anabolic-androgenic steroids for enhancing athletic performance and building muscle mass, especially among professional athletes and bodybuilders (422). Anabolic steroids are also used to generally improve male appearance and emotional mood. This practice has been labeled ‘substance abuse’ because many of these compounds are synthetic testosterone derivatives, which can increase its potency by several folds (423). Consequently, their use can lead to male infertility by their negative feedback on the HPG-axis, resulting in reduced gonadotrophin stimulation of the testis and subsequent hypogonadotropic hypogonadism (422). While testicular function typically recovers within 2 years after discontinuation (424), gonadotropin replacement injections may be necessary to expedite recovery in some cases (425). In other instances, hypogonadism can be permanent, even after discontinuing the use of synthetic androgens.

As related to food animal production, androgens are given together with estrogens for the purpose of promoting growth, as in the pig and bull (426–428). While there is negative feedback on the HPG and reproductive behavior and fertility can be inhibited, the decrease in testosterone and the inhibition of gonad function are transient (426, 428). On the other hand, there is debate regarding the safety of steroid hormone treatment in food-producing animals, especially when administered close to the time of slaughter. Thus, some countries, such as EU, have banned their use, and others, such as the USA, allowed them with the condition of removing and discarding the site of administration (the ear) at slaughter.

Estrogen treatment in the adult male also suppresses testosterone production by negative feedback on the HPG and is used in gender-affirming hormone therapy (429). While the negative feedback on the HPG did suppress LH, FSH, and testosterone and, in general, resulted in hypoplasia of germ cells, it has been surprising to find that estrogen treatment did not arrest spermatogenesis (430). The effects appear to be reversible after treatment has stopped (431). However, observed changes in the rete testis and epididymis, which included epithelial hyperplasia and interstitial fibrosis (430), suggest that infertility could be permanent by blockage of the reproductive tract.

Overall, estrogen or androgen treatments in the adult male do not induce sterility consistently and could be problematic due to the adverse effects on other organs (429, 432). However, a transient neonatal administration of sex steroids, prior to the onset of puberty, has shown potential for inducing sterilization. Androgens, progestins, and estrogenic compounds have been tested, but it appears that estrogen or estrogen plus an androgen give the strongest response for permanent inhibition of spermatogenesis and decreases in testosterone (79, 433–439). On the other hand, long-term effects have not been consistent across studies, with some showing full recovery, while others showed persistent hypogonadism. Thus, the possibility of using neonatal steroid treatments to induce sterility is a complex matter, depending on several factors including species, type of steroid, age at exposure, and total dosage.

Experimental studies have shown that neonatal estrogen treatment (soon after birth) resulted in a more targeted effect on the male reproductive system, with fewer side-effects, but considerable variation between species. For example, in a recent study, newborn male rats injected daily with estradiol benzoate for 10 days, showed profound suppression of *Kiss1* expression in the ARC region of the hypothalamus, and resulted in permanent inhibition of testicular growth and spermatogenesis (79). Similar research on females concluded that production of sterility was due to the precise targeting of the kisspeptin neurons during a critical period of neonatal development, which permanently suppressed pituitary gonadotrophins LH and FSH (57). Regardless of testicular effects, estrogen also has direct effects on the reproductive tract, particularly targeting the efferent ductules, where ESR has the highest expression and is co-expressed with AR (440, 441). Disrupting the ESR1 or AR pathway in the male reproductive tract can permanently disrupt luminal physiology, leading to back-pressure atrophy of the testis, independent of the HPG axis (132, 133, 139, 440). Accordingly, two distinct hormonal target mechanisms can cause testicular atrophy and sterility: (a) permanent suppression of the hypothalamic Kisspeptin pathway leading to the reduction in pituitary gonadotropin secretion, testosterone production, and sexual behavior and (b) direct inhibition of steroid hormone receptor function in the male reproductive tract, which may or may not affect testosterone levels but can result in sterility due to blockage of sperm transport (133).

It is well established that exposure to estrogenic compounds during fetal development can induce male infertility, and a variety of detrimental health effects now associated with what is called ‘testicular dysgenesis syndrome’ (TDS) in most mammalian species (442–447). Thus, fetal or neonatal over-exposure to steroid hormones in the male is of greatest concern. Symptoms of TDS include cryptorchidism (undescended testes), hypospadias (abnormal urethral opening), poor semen quality, and testicular cancer (448–454), as well as stimulated growth of the prostate gland (455, 456). Indeed, loss of testosterone action during the neonatal period not only blocked the required stimulus for germ cell development but it also prevented normal descent of the testis into the scrotum, producing cryptorchidism (79, 457, 458). Because cryptorchidism alone induces testicular degeneration (459–461), similar to that observed after neonatal treatment with estrogens, further development of this sterilization method would require careful study of testis descent in each species, considering that in some species, the testes are already descended through the inguinal canal at birth (462). Overall, further study is required to determine if an optimum dose and treatment duration can be achieved to induce male sterility without incurring the side effects associated with the use of steroid hormones in developing male animals, such as cryptorchidism (79, 437, 463–466).

In summary, the use of hormone treatment to induce sterility does not appear to be an efficient method, as it typically induced only temporary infertility or contraception. The most successful for companion animal use has been the GnRH agonists/antagonists, but the need of repeated injections raises issues of convenience and costs. The use of androgenic compounds in men has resulted in numerous side effects, and in some cases, reversal of gonad inhibition has been challenging. Estrogen treatments also inhibit male reproduction simply by providing negative feedback to the HPG axis, but this method has shown inconsistency and could potentially produce serious side effects on other organs. On the other hand, inducing permanent infertility through the administration of steroid hormones during the neonatal period would be a viable method with great potential for application in pets and livestock. Nevertheless, such treatments will require careful attention to dose and timing of exposure. It is also important to notice that the use of hormones in food-producing animals is highly regulated due to food safety considerations.

### 3.5 Immunological sterilization

The induction of sterilization using a vaccine, also called immunocastration, has historically been one of the most explored alternatives to castration of males. However, the term ‘immunocontraception’ is often used interchangeably with ‘immunocastration’ because of the potential reversibility of the infertility effects. In principle, this is a quite versatile method, as it allows targeting any component of the HPG axis by stimulating the immune system to produce specific neutralizing antibodies against the host proteins. As described below, numerous studies have successfully tested vaccines in companion, farm, and wildlife animals (254). In the US, the technology for antibody-mediated immunocontraception received a patent in 2014 (467). Although most immune methods that only provide contraception and reimmunizations are necessary, studies have shown that sterility can be achieved by using specific methods and immune targets (195, 468, 469).

Immunocastration has shown great success in livestock, including ram, buck, boar, and bull (190, 195, 470). Immunization against GnRH or KISS1 not only inhibits spermatogenesis, rendering the animal infertile, but also decreases androgen-related male behaviors and odors. This approach is safe for the food chain and relatively easy to integrate with other routine vaccinations. Moreover, it can potentially minimize the negative effect that a decrease in androgens has on animal growth by waiting until pubertal age to administer the vaccine. However, some management-related issues have limited their widespread application in commercial settings. In specific, GnRH and KISS1 vaccines developed for livestock need two doses to achieve the desired testis-blocking effect (190, 471), leading to increased costs and labor. This is not the case for GonaCon™, a GnRH vaccine developed by the National Wildlife Research Center (Fort Collins, CO) to control wildlife populations (472, 473); but, it is only registered for use in female wild equids, white-tailed deer, and prairie dogs, and booster vaccinations are necessary to prolong its effect.

The use of immunocastration as an alternative to neuter surgery of male cats and dogs is also of great interest because it has the potential to prevent male sexual behavior, as well as inducing infertility. In cats, immunocastration has been proposed as a potential cost-effective tool for controlling populations of free-roaming animals (474). Finally, immunocastration is a useful method for wildlife management as an alternative to massive culling (473). Some models estimate that a method involving contraception can be as effective as culling (475). In addition, it has the advantage of allowing for remote delivery (e.g., using preloaded darts), therefore reducing the stress of capture and restraining the animals (473). However, except for species that have a harem mating system, almost every male would need to be treated to affect the population size, whereas a more efficient outcome can be achieved by targeting females (476).

#### 3.5.1 Anti-sperm antibodies

Impaired fertility or complete infertility has been achieved by antigens targeting sperm, as reviewed by others (477–480). First attempts involved the use of whole sperm extracts to immunize females. A 1932 study reported infertility in women during a year-long trial after being injected with their partner’s semen (481). However, this is no longer regarded as a feasible approach, as the shared antigens between sperm and somatic cells led to pathological side effects (477). Thus, the search for a specific sperm membrane antigen was initiated, as such specificity would likely be safer and treatment could be targeted to the male rather than the female.

The current list of sperm antigens which has been targeted for their immunocontraceptive potential keeps increasing as the functions of more sperm membrane proteins are elucidated. Table 4 highlights some successful examples of active immunization against sperm proteins. Moreover, passive immunization with anti-sperm antibodies (ASA) has also been explored. For example, antibodies against 80 kDa HAS induced infertility in both male and female rats due to agglutination of epididymal sperm and impairment of sperm motility (482). However, variable levels of contraception were achieved, partly due to the overlap of sperm protein functions with other cell types.

**Table 4:** Examples of anti-sperm antibodies (ASA) used for active immunization.

ASA	Species	Protein type	Protein function	Main results
Sperm adhesion molecule 1 (SPAM1) or PH-20	Guinea Pig	Hyaluronidase and Receptor	Penetration through the cumulus cells surrounding the egg. Also binds to the zona pellucida after acrosome reaction.	Temporal infertility (1 year or longer) and absence of normal sperm in the epididymis
Epididymal Protease Inhibitor (Eppin)	Monkey	WFDC (whey-acidic protein four-disulfide core) protease inhibitor family	Binds to the semen coagulum protein semenogelin-1 (SEMG1), which transiently inhibits sperm motility after ejaculation.	Temporal infertility (approx. 2 years)
Human fertilization antigen-1 (FA-1)	Mouse	Glycoprotein	Binds to the zona pellucida (ZP3)	Temporal reduction in fertility (for up to 10 months) and blocked binding to zona pellucida *in vitro* fertilization
ADAM metallopeptidase domain 2 (ADAM2) or Fertilin or PH-30	Guinea pig	ADAM family member (membrane-anchored protein)	Sperm-egg adhesion and fusion with the egg plasma membrane	Infertility (recovery not determined)
80 KDa Human sperm antigen (80 kDa HSA)	Marmoset	Glycoprotein	Progressive motility	Temporal infertility (8–10 weeks after booster dose). Loss of sperm progressive motility

Overall, sperm-immunization is mainly interesting from the male human contraception perspective, in which the aim is to impair sperm function without affecting hormone synthesis. Instead, targeting components outside the HPG axis appear to have little value for companion and farm animals, as the prevention of male reproductive and aggressive behaviors is often more important than inducing infertility.

#### 3.5.2 Anti-LH or LHR antibodies

Two N-terminal fragments of the porcine LH receptor were used for active immunization of prepubertal male mice and achieved a reduction in testosterone, azoospermia, and infertility in 75% of the males (483). In the rabbit, this approach did not work because the receptor antibodies showed both LH agonistic and antagonistic activities (484). Attempts were also made using vaccines based on ovine LH and LH receptors (isolated from sheep testis), which were tested in rabbits and monkeys (485). Overall, these studies showed that ovine LH vaccines produced desirable antibody titers and resulted in a drastic decrease in serum testosterone (~90%), a reduction in seminiferous tubule spermatids (>90%), and, consequently, azoospermia and infertility (485). However, due to side effects related to the lack of androgen production, such as muscle loss or alopecia, the pursuit of an anti-LH contraceptive vaccine for men was soon abandoned.

#### 3.5.3 Anti-FSH or FSHR antibodies

Early trials of ovine FSH itself or FSHβ subunit vaccines led to encouraging results in non-human primates (485). In these, spermatogenesis was impaired without affecting testosterone levels, and a low proportion of non-responders (i.e., individuals with low antibody titers) was achieved (485). Although sperm production was only qualitatively and not quantitatively impaired, monkeys were rendered infertile. Similarly, immunization against FSH receptor (FSHR) induced a decrease in sperm concentration and sperm motility in monkeys, as well as infertility in subsequent mating trials (486). Moreover, passive immunization of male bonnet monkeys with ovine FSH also resulted in testicular dysfunction, oligozoospermia, and infertility (487). However, long-term contraceptive efficacy with FSH immunization was not achieved, as the antibody titer did not last more than 90–100 days (488). Consequently, further testing was discouraged due to the nature of transient effects, which would necessitate continuous immunization to maintain contraception.

The improvement of methods for synthesis of recombinant proteins has allowed for the optimization of these vaccines by targeting specific peptides that can induce a higher immune response. For instance, four FSHR extracellular domain (ECD) peptides involved in FSH binding that are potent B-cell epitope peptides were identified as specific inducers of a B-cell immune response (489). Vaccines were then engineered by conjugating an ECD peptide in tandem with a T-cell epitope. Three of the four peptides tested induced infertility in male mice, which showed decreased serum testosterone and sperm concentration (489). They later refined this method by performing simultaneous immunization with B-cell epitopes of both human FSHR and Eppin, a sperm antigen (490). This dual FSHR-Eppin target enhanced the contraceptive effect, causing up to 95% infertility in male mice.

In conclusion, FSH or FSHR vaccines received attention as a method for immunocontraception in men, which would induce reversible infertility without side effects related to a reduction in androgen synthesis. Unfortunately, they failed to reach an acceptable level of contraception. As related to companion and farm animals, the LH/LHRH vaccines showed more potential for preventing androgen-related behaviors and odors, but the greatest success thus far has been from targeting GnRH for immunocastration (depending on the species), as it has the potential to disrupt the entire HPG axis and work in both males and females.

#### 3.5.4 Anti-GnRH antibodies

GnRH has been the main target for immunocastration of domestic male animals. In fact, commercial vaccines are available for bovine (bulls) and swine (boars). One of the major appeals is that identical GnRH vaccines can potentially be utilized for both females and males of the same species. By preventing the binding of native GnRH to its receptor on pituitary gonadotrophs, the release of both LH and FSH is inhibited. Consequently, both spermatogenesis and ovulation are disrupted, and the production of steroid hormones is inhibited. Therefore, GnRH vaccines can prevent male aggressive and mounting behaviors, in addition to inducing infertility.

There are 30 structurally different forms of GnRH that have been identified in animals (491). GnRH-I is the one mainly utilized for immunocastration, as it has four residues in the N-terminus and C-terminus that are involved in receptor binding and are conserved across species (491). Because GnRH is a small decapeptide, it needs to be conjugated to a carrier protein to become immunogenic (174). Thus, effective vaccines have been developed in male rats by conjugating GnRH with tetanus toxoid (TT) (492), diphtheria toxoid (DT) (493), and ovalbumin (OA) (494), among others. Other approaches for constructing non-conjugated GnRH vaccines with enough immunogenic capacity involve the polymerization of the GnRH peptide, such as the D-Lys6-GnRH-tandem-dimer peptide (TDK) (495).

Studies in male rats have validated that GnRH immunization reduces GnRH content in the median eminence of the hypothalamus, leading to a decrease in serum LH, FSH, and testosterone and, consequently, suppression of spermatogenesis and testicular atrophy (496). Moreover, GnRH vaccination decreases the hypothalamic mRNA expressions of GnRH, GnRH receptor, AR, Kiss1, and Kisspeptin receptor (GPR54) (496, 497). Therefore, it appears that anti-GnRH immunization further decreases GnRH release and disrupts the HPG axis by decreasing the entire androgen-AR-Kisspeptin-GPR54 signaling pathway. Interestingly, the basic structural organization of the testicular components is maintained after GnRH immunization (492), which indicates that an eventual recovery of function is possible. In fact, total recovery of spermatogenesis was observed 300 days after GnRH vaccination of male rats (493). However, atrophic changes observed in the epididymis suggested that prolonged GnRH immunization may lead to irreversible damage and sterility (492). In addition, the decrease in androgen synthesis also affects the size of accessory organs, including vesicular gland and prostate (494), which is why GnRH vaccines have gained attention for the management of prostate cancer in men.

**Figure 4:**
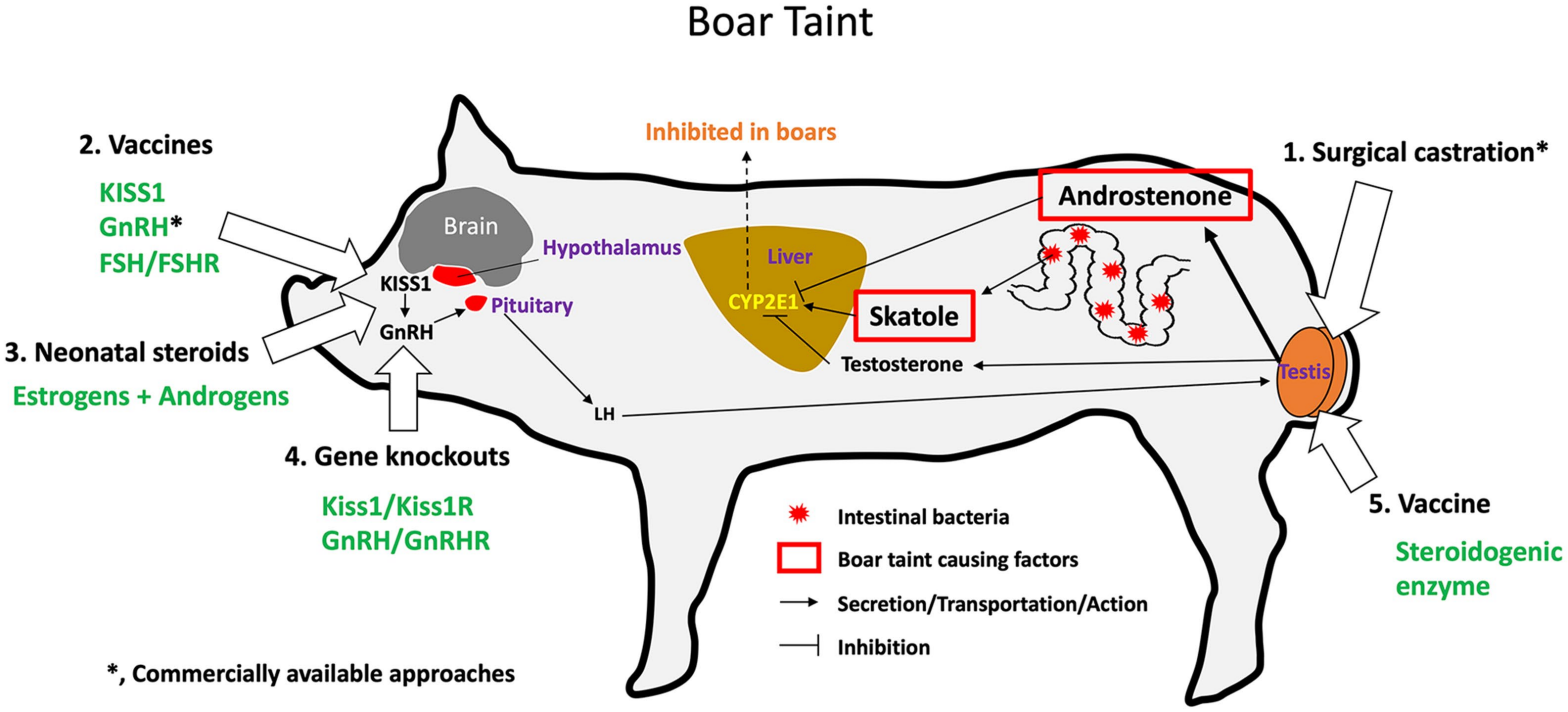
Causes of boar taint, current solutions, and potential targets for the inhibition of androstenone and skatole production in the male pig. Boar taint is the pungent odor produced by cooked pork, especially present in the meat from boars with intact gonads. The odor is due to the buildup of skatole and androstenone in the meat. Skatole comes from bacteria in the gastrointestinal tract and androstenone is an androgen produced by Leydig cells of the testis. Skatole is normally metabolized in the liver by CYP2E1, but androstenone, the dominant androgen in pigs, inhibits this enzyme, thereby allowing for the buildup of skatole in the blood and tissues. The three components of the HPG axis are indirectly involved in the cause of boar taint, as the cascade of hormonal stimulation of the testis involves KISS1, GnRH, and LH. Thus, there are numerous targets for potential inhibition of boar taint production. 1. Simple removal of the source of androstenone and testosterone by castration has been used for thousands of years. 2. The hypothalamus has been targeted by developing vaccines against KISS1 and GnRH. Commercial availability is indicated by an asterisk (*). 3. The hypothalamus and pituitary organs can also be inhibited by treating newborn animals with a combination of estrogen and androgens, an experimental approach that is currently under development. 4. Gene knockout technology is also being tested experimentally to remove Kiss1 gene or its receptor, Kiss1R, as well as to eliminate the GnRH gene or its receptor, GnRHR, which would thereby inhibit the production of LH. 5. Direct inhibition of testicular Leydig cells could also be targeted by the development of specific vaccines to block steroidogenic enzymes in the testis, which would decrease the production of androgenic steroids that help to maintain skatole and the buildup boar taint molecules in the meat.

A summary of recent advances in GnRH immunocastration of farm and companion animals is presented in Table 5. Although some of these studies used experimental vaccines, most were commercial products. GonaCon™ (National Wildlife Research Center, Fort Collins, CO), which was developed for wildlife population control (498) and contained GnRH-I conjugated to keyhole limpet hemocyanin (KLH), had been used mostly in females (499–501). Improvest® or Improvac® (Zoetis, NJ), which is commercially available for male pigs in the USA and EU, respectively, contains an incomplete GnRH analog conjugated to DT; Bopriva™ (Zoetis). GnRH analog conjugated to DT was developed for beef cattle. Equity® (Zoetis) is a GnRH-DT conjugate which is indicated for estrous control of horses. Vaxstrate® (Arthur Webster Pty Ltd, NSW), currently a discontinued GnRH-OA conjugate, was developed for pregnancy prevention in cows. Canine GnRH Immunotherapeutic® (Pfizer Animal Health, PA), also a GnRH-DT conjugate, was developed for treating benign prostatic hyperplasia in dogs but is currently discontinued. Note that the goals of immunocastration in farm animals include reduction of male aggressive/reproductive behavior, improvement of meat and carcass characteristics, improvement of feed efficiency relative to physical castrates, and prevention of male-associated odors (502). Male pigs, specifically, are mainly castrated to prevent boar taint (Figure 4), and the unpleasant odor in pork meat from intact boars is indirectly caused by androgen synthesis and the buildup of androstenone and skatole (503, 504). Therefore, in Table 5, studies targeting these specific production characteristics are included. On the other hand, in companion animals, immunocastration is regarded as an alternative to neuter surgery, and thus, the end-goal in cats and dogs is to induce permanent or temporary infertility while reducing male behaviors (505, 506).

**Table 5:** Effects of GnRH-immunization in domestic animals in terms of reproduction, behavior, and production performance.^1,2^

Effect	Age^3^	Vaccine^4^ (Doses/Interval)	Main Results^5^
Bull (*Bos taurus* & *Bos indicus*)			
Reproduction	Prepubertal <6 months	Bopriva® (2 doses/3 weeks)	Decreased serum T up to 22 weeks old, and scrotum size up to 44 weeks old.
	Peripubertal 6–14 months	GnRH-OA conjugate (3 Doses/4–5 months)	Decreased scrotum size for more than a year.
		Bopriva® (2 Doses/6 weeks)	Decreased serum T and testis weight up to 15 weeks after booster.
		Improvac® (3 doses/3–15 weeks)	Decreased scrotum and testis size. Arrested spermatogenesis up to 20 weeks after booster.
	Adult >22 months at booster	GnRH-OA & GnRH-Trx conjugates (3 doses/20 weeks)	Decreased testosterone and testis weight up to 8 months after first booster.
		Bopriva® (2–3 doses/1–3 months)	Decreased scrotum size, sperm motility, and testosterone up to 4 months after first booster.
Behavior	Prepubertal <6 months	GnRH-KLH conjugate (2 doses/1–6 months)	Reduced aggressive behavior (frequency of butts and sparring) compared to intact bull.
	Postpubertal 8–22 months	GnRH-KLH conjugate (2 doses/8 months)	Reduced aggressive behavior (frequency of butts and sparring) compared to intact bull.
Performance	Postpubertal 8–22 months	Bopriva® (2 doses/4–6 weeks)	*Compared to bulls*: Lower ADG, HCW, and dressing percentage. Higher fat thickness and marbling. *Compared to steers*: Higher ADG. Similar HCW, dressing percentage, fat thickness and meat quality.
	Adult >22 months	GnRH-OA conjugate (3 doses/2 weeks)	*Compared to bulls*: Similar carcass weight, and dressing percentage. Higher fat depth and marbling. *Compared to steers*: Similar carcass weight, and dressing percentage. Higher marbling.
		Bopriva® (2 doses/3 months)	*Compared to steers:* Higher ADG and HCW. Lower dressing percentage. Similar meat quality.
Boar			
Reproduction	Early-treated ~10 weeks	GnRH-OA conjugate (2 doses/8 weeks)	Decreased testis size, serum LH, FSH, and T. Absence of mature sperm in seminiferous tubules up to 8 weeks after booster.
		GnRH-MBP conjugate (2 doses/8 weeks)	Decreased scrotum size and plasma T. Absence of mature sperm up to 7 weeks after booster.
		Improvest®/ Improvac® (2 doses/5–10 weeks)	Decreased testis weight and serum T. Absence of mature sperm in seminiferous tubules up to 10 weeks after booster.
	Late-treated 15–18 weeks	Improvac® (2 doses/4 weeks)	Decreased testes weight, bulbo-urethral gland weight, and serum LH, T, and androsterone up to 10 weeks after booster.
Behavior	Early-treated ~10 weeks	GnRH-MBP conjugate (2 doses/8 weeks)	Delayed onset of mounting behavior (4 months later than intact boars).
		Improvac® (2 doses/10 weeks)	Decreased activity (time standing), sexual (mounting), and aggressive behavior (biting and fighting), and skin lesions compared to intact boars.
	Late-treated 15–16 weeks	Improvest®/ Improvac® (2 doses/4–6 weeks)	Decreased aggressive and mounting behaviors, and skin lesions compared to intact boars.
	Heavy pork 37–41 weeks	Improvac® (2–4 doses/4–10 weeks)	Decreased social, aggressive, and mounting behavior compared to intact boars up to 22 weeks after booster.
Performance	Early-treated ~10 weeks	Improvac® (2 doses/9–10 weeks)	*Compared to barrows*: Similar-higher ADG. Lower FCR. Similar-higher lean meat percentage. *Compared to intact boars*: Similar-higher ADG, and FCR. Lower lean meat percentage.
	Late-treated 15–18 weeks	Improvest®/ Improvac® (2 doses/4–6 weeks)	*Compared to barrows*: Higher ADG and feed intake. Lower FCR. Lower dressing percentage, marbling and bacon slicing yield. Higher lean meat percentage. *Compared to intact boars*: Higher ADG, feed intake, and FCR. Lower dressing percentage and lean meat percentage. Similar bacon characteristics.
	Heavy pork 31–41 weeks	Improvac® (2–4 doses/4–10 weeks)	*Compared to barrows*: Higher ADG and HCW. Lower FCR, dressing percentage, and backfat thickness.
Boar Taint	Early-treated ~10 weeks	Improvac® (2 doses/9 weeks)	*Compared to barrows*: Similar androstenone and skatole concentration in fat. *Compared to intact boars*: Lower androstenone and skatole concentration in fat.
	Late-treated 15–18 weeks	Improvac® (2 doses/4–6 weeks)	*Compared to barrows*: Similar androstenone and skatole concentration in fat. *Compared to intact boars*: Lower androstenone and skatole concentration in fat.
	Heavy pork 37–41 weeks	Improvac® (2 doses/4–6 weeks)	*Compared to intact boars*: Lower androstenone and skatole in fat up to 22 weeks after booster.
Small Ruminants (Ram & Buck)			
Reproduction	Prepubertal Lamb < 4 months	GnRH-OA & GnRH-Trx conjugates (3 doses/4–8 weeks)	Decreased scrotum size and absence of mature sperm in seminiferous tubules up to 23 weeks after first booster.
	Peripubertal Lamb 4–8 months	GnRH-OA conjugate (2 doses/4 weeks)	Decreased serum LH, FSH, and T. Decreased scrotum and accessory glands size. Absence of mature sperm in seminiferous tubules up to 22 weeks after booster
		Improvac® (2 doses/2–4 weeks)	Decreased scrotum size up to 4 weeks after booster.
		Bopriva® (2 doses/4 weeks)	Decreased plasma T, and sperm concentration in ejaculates (azoospermia in 70–80% of males) up to 1 month after booster.
	Adult Buck >1 year	Vaxstrate® (2 doses/2–4 weeks)	Decreased plasma LH, FSH, and T, sperm concentration in ejaculates, and scrotum size for more than a year in 90% of animals.
Behavior	Prepubertal Lamb < 4 months	GnRH-KHL conjugate (Regime not specified)	Decreased sexual behavior (frequency of mounts and ejaculations).
		GnRH-OA & GnRH-Trx conjugates (3 doses/4–8 weeks)	Delayed onset of mounting activity (5 weeks later compared to intact ram).
	Adult Buck >1 year	Vaxstrate® (2 doses/2–4 weeks)	Decreased agonistic behavior and male odor associated with reproductive season.
Performance	Prepubertal Lamb < 4 months	GnRH-KHL conjugate (Regime not specified)	*Compared to physically castrate:* Similar ADG, FCR and dressing percentage. Lower marbling and back fat thickness. *Compared to intact ram:* Lower ADG. Higher FCR, and dressing percentage. Similar marbling and backfat.
	Peripubertal Lamb 4–8 months	GnRH-OA & GnRH-Trx conjugates (2 doses/8 weeks)	*Compared to physically castrate:* Similar ADG, HCW, dressing percentage and other carcass measurements. *Compared to intact ram*: Similar ADG, HCW, and dressing percentage. Higher subcutaneous fat.
		Improvac® (2 doses/2–4 weeks)	*Compared to intact ram:* Similar ADG, HCW, and dressing percentage.
Dog			
Reproduction & Behavior	Prepubertal <6 months	GnRH-CDV Th cell epitope p35 conjugate (2 doses/4 weeks)	Decreased testes size and absence of mature sperm in seminiferous tubules up to 14 weeks after booster (3/8 dogs).
	Adult >1 year	GnRH-TT conjugate (3 doses/2–6 weeks)	Decreased serum T and scrotum size up to 28 weeks after booster (5/12 dogs), and absence of mature sperm in seminiferous tubules.
		Canine GnRH Immuno- therapeutic® (2 doses/4 weeks)	Decreased serum LH, serum T, and testicular volume up to 8 weeks after booster (4/4 dogs).
		GnRXG/Q antigen recombinant protein (2 doses/4 weeks)	Decreased serum T and sperm concentration in ejaculates up to 8 months after booster (5/7 dogs). Decreased sexual, agonistic, and marking behavior.
Cat			
Reproduction	Prepubertal <4 months	GnRH-LKTA conjugate (2 doses/4 weeks +2-year dose)	Decreased serum T and absence of mature sperm in seminiferous tubules (3/4 cats).
	Peripubertal 4–9 months	GnRH-MBP conjugate (2 doses/6 weeks)	Decreased serum T, testis size and absence of mature sperm in seminiferous tubules up to 6 weeks after booster.
		GnRH-STF2 conjugate (2 doses/4 weeks)	Decreased serum T, testis size and number of mature sperm in seminiferous tubules up to 5 months after booster (6/14 cats).
	Adult >9 months	GnRH-KHL conjugate (2 doses/not specified)	Decreased serum T and scrotal size. Absence of sperm in ejaculates at least up to 6 months after booster (6/9 cats).
		Improvac® (2 doses/4 weeks)	Decreased serum T, scrotal size, and sperm concentration in ejaculates up to 20 weeks after booster.
Stallion			
Reproduction & Behavior	Young Horse (<4 years)	Improvac® (2 doses/4 weeks)	Decreased serum T and testis size up to 10 weeks after booster
	Adult Horse (>4 years)	Equity™ (3 doses/4–8 weeks)	Decreased plasma T, scrotal circumference, libido, and sperm concentration in ejaculates up to 6–8 months after booster.
	Adult Pony	GnRH- OA conjugate (2 doses/2 weeks)	Decreased serum T, scrotal size, and libido up to 6–14 months after booster. Low or absence of mature sperm in seminiferous tubules.

^1^Others have reviewed studies before 2000; therefore, this table mainly includes studies thereafter. ^2^For supporting references see: Supplementary Table S5 References. ^3^Age refers to age at booster or second immunization, except for boars. In the boar section, *early-treated* and *late-treated* refers to age at first immunization, and *heavy pork* refers to age of slaughter (37–41 weeks, instead of the conventional 24–26 weeks old). ^4^OA, Ovoalbumin; Trx, Thioredoxin; MBP, Matose binding protein; KLH, keyhole limpet hemocyanin; TT, Tetanus toxoid; CDV, canine distemper virus; Th, T helper; LKTA, leukotoxin A; STF2, *Salmonella typhimurium* flagellin fljB. FSH, Follicle Stimulating Hormone; LH, Luteinizing Hormone; T, Testosterone. ^5^BW, Body Weight; HCW, Hot Carcass Weight (Kg; weight of the carcass after slaughter and the removal of the head, hide, intestinal tract, and internal organs); ADG, Average daily gain (Kg/day; increase in BW per day); FCR, Feed conversation ratio (Kg/Kg; weight of feed intake divided by BW gained); Dressing percentage or carcass yield (Kg/Kg; HCW expressed as the percentage of the BW at slaughter); Lean meat percentage (Kg/Kg; sum of denuded shoulder, back and ham weights as percentage of cold carcass weight); Marbling (intermingling of fat with lean in a muscle); Bacon slicing yield (Kg/Kg), percentage of green weight (weight of fresh pork bellies after being skinned and trimmed).

GnRH immunocastration has received greater attention for hog production, as denoted by the vast number of published studies in male pigs. The immunization requires a second injection before puberty to be effective (4 weeks after the first dose is recommended by the vaccine manufacturer). Clearance of androstenone from fat was achieved 3 weeks after the second GnRH immunization (507). The need for two doses decreases the attractiveness of immunocastration for some producers, simply due to the higher costs and repeated handling of larger animals. In addition, the timing of the two doses relative to slaughter is particularly important, as reproductive function tends to recover over time. While disrupted testis morphometry and spermatogenesis are reported in GnRH-immunized pigs at slaughter age (~6 months old) (497, 508, 509), boars treated with two doses of Improvest® at 10 and 15 weeks of age recovered testosterone levels by 40 weeks of age and sperm concentration and motility by 60 weeks (509). Because hogs are usually sent to market by 24–26 weeks of age, before the testis can recover, the immunization procedure is considered equivalent to castration. The exception is heavy pork production systems, in which hogs are fattened up to higher weights and age (>37 weeks old) to produce cured ham meat. In these systems, one or two additional booster vaccines (total of 3–4 doses) can prevent boar taint up to the late slaughter ages (510, 511).

On the other hand, others have argued that the cost of the vaccine, including the increased workload, may be compensated by better growth performance and higher carcass leanness compared with surgical castrates (512). A meta-analysis concluded that GnRH-immunocastrated pigs had higher average daily gain (ADG; g BW/day) compared with barrows and intact boars (513). Thus, boar taint is reduced to levels of castrates without significantly affecting growth. Moreover, they stated that immunocastrates had a lower feed conversation ratio (kg of feed needed for kg of BW) than barrows, although higher than intact boars (513). In terms of hot carcass weight, dressing percentage, fat deposition, and meat quality parameters, immunocastrated boars fell in between barrows and intact boars (513). The maintenance of testicular activity in immunized males until second dose or booster is believed to account for the higher ADG and lower fat deposition compared with barrows (514). However, variability in the timing from booster to the biological response has been noted in pigs, which, in turns, affects their production performance (515). The age at vaccination and time before slaughter can also affect fat deposition and meat quality attributes (470).

Despite the relative success of GnRH immunocastration in bulls, preventing both spermatogenesis (516) and bull aggressive behavior (517), the inability to maintain these effects until slaughter age has likely hampered its application in beef cattle. For instance, bulls immunized against GnRH at 3 and 6 weeks of age recovered spermatogenic capacity by approximately 68 weeks of age (518). Because spermatogenesis and testosterone production recover over time, accurate timing of vaccines is needed for the effects to last until slaughter age. Regarding small ruminants (rams and bucks), GnRH immunization also disrupted spermatogenesis, as denoted by smaller seminiferous tubules that are depleted of mature sperm (519, 520). Moreover, GnRH immunization was able to reduce aggression and mounting behavior in both ram (521) and bucks (522). However, no commercial vaccine is available for these species. Although most of the trials in ruminants have been performed in peripubertal or mature males, some studies indicate that GnRH vaccines could be used to delay puberty: e.g., bull calves immunized at 3–6 weeks of age had a 3-week delay in reaching a 28-cm scrotal circumference (518) and GnRH-immunized prepubertal lambs showed a 4-month delay in the onset of mounting behavior (519).

Regarding companion animals, GnRH immunization leads to variable outcomes. Despite overall success at disrupting testicular function, considerable variation in the antibody titer response was reported in GnRH-immunized toms (523–525). Although achieving a relatively high percentage of infertility is enough to reduce the size of a population of free-roaming cats (474), the need for repeated dosing to maintain infertility limits its application for population management purposes. In male dogs, the results have been even less promising. Although immunization decreased testis size, with spermatogenesis arrest and even lowered testosterone levels, the effects were inconsistent due to high individual variability (526–529). Further difficulty with assessing the utility of immunocastration in dogs is that studies thus far have used very small sample sizes and lack breeding trials. Notably, no side effects were reported, apart from mild inflammation at the injection site (528, 530). Moreover, these vaccines can potentially be conjugated with viral antigens for animals to be immunized at the same time against common diseases, such as rabies or distemper (527). Although the reversibility of the effects would limit their use to that of a contraceptive [e.g., testicular size and serum testosterone concentrations recovered 16 weeks after GnRH immunization booster in male dogs (529)], this method still holds great promise.

GnRH vaccines have also been examined as a method for managing wildlife populations, including deer, horses, squirrels, prairie dogs, elk, elephants, brushtail possums, and wild boars, as reviewed by others (174, 476, 531). GonaCon™, in particular, shows long-term effectiveness at reducing fertility in white-tailed deer and wild horses by targeting females (473). However, the use of GnRH vaccines in males has been discouraged in some wildlife species due to the disruptive effect of androgen dysfunction on their social hierarchy (476). For instance, immunocastration impaired the ability of wild stallions to hold breeding bands (476) and the antler development in male white-tailed deer (174). However, there are some examples of successful application in males. In the capybara, GnRH immunization induced infertility without affecting their social structure (498). In male elephants, a GnRH vaccine prevented aggressive behavior which was occurred during rutting season (i.e., musth) (532) and thus could be a helpful tool for preventing severe conflict in human communities living close to wild elephants. In the common eland antelope, immunization with Improvac® reduced aggressive behavior of juvenile males without negatively affecting their social behavior (533). Finally, in fallow deer farming conditions, Improvac® vaccination was combined with amino acid supplementation and the treatment led to atrophied seminiferous tubules and decreased sperm viability. However, only a mild effect on antler development (lighter antlers and lower mineralization) was observed, presumably due to a lack of total suppression of testosterone, although testosterone was not measured (534, 535). However, repeated dosing was required.

#### 3.5.5 Anti-Kisspeptin antibodies

The relatively recent discovery of Kisspeptin’s critical role in the HPG axis has turned the focus of immunocastration toward the development of an anti-KISS1 vaccine. Thus far, this approach has been tested only in small ruminants, but the promising results in terms of efficacy and safety will likely encourage trials in other species, especially in those for which the Kisspeptin pathway is better defined.

The first KISS1 vaccine developed, pKS-asd, was a recombinant plasmid constructed with the Human KISS1 fused with the hepatitis B surface antigen S (HBsAg-S) gene and was first tested in rams (536). This vaccine inhibited testicular function and resulted in a decrease in circulating testosterone, testis size, and spermatogenesis (536). In a follow-up study, the safety of the pKS-asd vaccine was assessed, and the KS fusion fragment of the vaccine was not detected in the host genome after vaccination (537). Although the vaccine showed promise for inhibiting male fertility in the ram, the effect was not permanent, as testosterone production and spermatogenesis recovered by weeks 22 to 30 post-immunization (537). Another study in rams immunized with pKS-asd reported a significant alteration in the expressions of genes associated with cellular immunity specific to the testis (469). Specific effects were noted on the cytotoxic pathway of Leydig cells mediated by natural killer cells, and it was suggested that these could lead to permanent infertility.

A second KISS1 vaccine, PVAX-B2L-Kisspeptin-54-asd (PBK-asd), was developed using an antigen of the Parapoxvirus (B2L), which caused a highly contagious disease in small ruminants; therefore, it acts as an immunomodulator maximizing the inhibitory effect on gonadal function (538). This vaccine achieved greater testicular atrophy in male rats than the pKS-asd and resulted in complete disruption of spermatogenesis (i.e., seminiferous tubules devoid of spermatids and mature sperm) (538). The PBK-asd vaccine was later successfully tested in both rams and bucks, causing a decrease in serum Kisspeptin, LH, and testosterone (538, 539). Consequently, gonads of treated bucks showed inhibited Leydig cell proliferation (540). Both pKS-asd and PBK-asd vaccines decreased the expression of not only KISS1 but also of AR, GPR54, and GnRH in the hypothalamus (538, 540, 541). Furthermore, there was a downregulation of LH, FSH, and GnRH receptors in the pituitary and of LH and FSH receptors in the testis (538, 540, 541). Altogether, these data indicate a complete disruption of the HPG axis and pathways needed for maintaining adequate testosterone synthesis and spermatogenesis.

Other outcomes in small ruminants indicate that KISS1-mediated immunocastration would be an interesting alternative for castration of farm animals. First, both PBK-asd and pKS-asd immunization decreased aggression and sexual behaviors in rams (i.e., frequency of mounting, sniffing, and butting) (469, 536). Second, bucks immunized with PBK-asd showed similar growth and carcass and meat characteristics compared with intact bucks, whereas surgical castration reduced daily gain and carcass weight (539). Third, an oral form of the pKS-asd vaccine has also been successfully tested in rams (471), which would represent an advantage over intramuscular administration in terms of management. In this study, prepubertal lambs treated with three doses of oral pKS-asd at 4 weeks of interval presented disrupted spermatogenesis, lower serum testosterone levels, and lower frequency of sexual behaviors, including butting, sniffing, and mounting (471). The one drawback, as described above for GnRH, is that anti-KISS1 vaccines produce only temporary infertility, and repeated vaccinations would be needed to extend reproductive inhibition until slaughter age. However, further study of the inflammatory response and a focus on the Leydig cell specifically could bring this method closer to that of castration.

#### 3.5.6 Other antibodies

Active immunization against testosterone synthesis was briefly tested. As reviewed by the authors (542), the results were inconsistent: either a low efficacy was achieved, probably due to the presence of other androgenic steroids that compensate testosterone’s role; or a paradoxical enlargement of the gonads was observed, as the lack of negative feedback on GnRH-LH release promoted testosterone synthesis. A more recent study targeted pregnenolone, the precursor of sex steroid hormones (543). Prepubertal rabbits actively immunized with pregnenolone-hemisuccinate covalently linked to Bovine serum albumin presented decreased serum testosterone and disrupted spermatogenesis with a very low number of mature sperm (543). However, fertility was not tested. Development of specific vaccines to block steroidogenic enzymes in the testis could also provide direct inhibition of the Leydig cells, which would decrease the production of androgenic steroids.

Alternative methods for active immunization have been explored. As described above, passive immunization has been tested as a contraceptive method, for example, by injecting FSH antibodies (487) or Human Sperm Antigen (482). This approach can represent an advantage over the use of vaccines, considering the difficulty of achieving high antibody titers (544). However, the sophisticated tools currently available for vaccine development, including antigen recombination to potent immunogens, largely overcome this inconvenience. Finally, an unspecific immune response can be triggered directly within the testis using intratesticular injections of immunogens, ultimately damaging the testis tissue. The most notable example is the combination of rabies vaccine and a chemical sterilant (zinc gluconate) which received a US patent in 2014 after trials in community dogs (545).

In summary, immunocastration is regarded as a feasible alternative to castration of males and appears to address some animal welfare concerns associated with castration. What is interesting about this method is the wide variety of targets to which antibodies can be developed, which can result in specific inhibitions ranging from hypothalamus/pituitary, upstream inhibition of the entire reproductive system, to specific cell types within the testis and even sperm within the epididymal lumen. Some vaccines are already commercially available for use in livestock. Despite improvement in productive parameters such as feed efficiency, the number of immunizations required to induce an effective response has hampered its widespread acceptance in some markets, such as the USA. Regarding companion animals, so far, research has shown great individual variability in response. Moreover, in-depth research on the appropriate frequency and number of doses must be conducted in pets.

### 3.6 Genetic sterilization

Genome-editing technology has the potential to revolutionize not only human health but also veterinary medicine and food animal production (546, 547). This method has been proposed as an alternative to surgical castration (548, 549), as it is now possible to inactivate any of the multiple genes responsible for regulating development and maintenance of the male reproductive system (Figure 1). Choosing the ideal gene to block testis function is important because pathways can have unexpected ways of compensating for the loss of a specific gene. Therefore, the first effort in genomic castration targeted the Kisspeptin/GnRH pathway, as these are situated upstream in the HPG axis. Inactivation of this pathway resulted in failure of the gonads to reach maturity, rendering the males sterile. The first experiment was the inactivation of KISS1R, Kisspeptin receptor, a protein on the GnRH neuron that is essential for the release of pituitary gonadotropins. This gene was edited in the White Composite male pig, which was then born sterile, as the adult testes of the homozygote GPR54 knockout (*GPR54*^−/−^) remained in a pre-pubertal state (550). Loss of pituitary stimulation resulted in low concentrations of serum androgens, which prevented the development of androgen-induced boar taint. From this original effort, Hendrix Genetics announced an alliance with Recombinetics/Acceligen to further the development of this genetic approach to replace surgical castration (551). Using CRISPR/Cas9 technology, the alliance has also targeted *KISS1* gene rather than the receptor and found a similar inhibition of puberty in the pig (552). However, this technology inevitably carries a problem, as homozygous sows and boars are sterile and unable to produce litters. A solution to this inborn problem is being sought, but finding an economically viable way to reproduce the *GPR54*^−/−^ pigs will be challenging. It is expected that CRISPR/Cas9 technology will be employed in coming years to target many other genes that are essential for reproductive organ development or functions.

In summary, the newer technology of gene editing holds great promise for replacing castration in animals, but choosing the best target cell/gene and resolving all the looming problems will be a challenge, particularly the ethical concerns over gene manipulation in the reproductive system. Moreover, the likely high cost of this technology would greatly reduce the application in livestock, while it may be acceptable for use in unique dog and cat breeds.

### 3.7 Evolution of male sterilization

This review discusses the past and current efforts to target various aspects of the male reproductive system using a variety of methods, ranging from surgical sterilization to treatments involving chemical toxicants, hormonal manipulations, and finally gene disruption (Figure 2). One important variable is the age at which sterilization is induced. From the literature, both advantages and disadvantages can be found for castration at the young or pubertal ages. If the goal is not only to induce sterility but also to inhibit male sexual behavior, as is typical in non-human males, a reduction in serum testosterone levels will be required, regardless of the method used or age at treatment. However, there are cases when it would be desirable to allow for normal androgen production while rendering the males sterile. Recent use of vaccines for ‘immunocastration’ appears to be successful to a certain degree, especially when targeting GnRH in the hypothalamus. However, a drawback of this approach is that it often requires additional injections, and failing to adhere to the schedule for these boosters can result in an inability to achieve the desired effects. Finally, with simpler methods for the disruption of the genetic code becoming available, gene targeting will become a promising method for developing novel approaches for male sterility.

The various alternatives presented so far have been developed with distinct purposes and can broadly be categorized into three types. The first type is designed to reduce the population of animal pests such as rats, the second is to replace neutering surgeries in pets, and the third is for efficient and humane castration of livestock. Consequently, not all methods can be uniformly applied to the sterilization of all mammalian species. It is crucial to understand the advantages and disadvantages of each method before selecting, refining, and optimizing a sterilization technique for a specific species. Factors to consider include animal welfare, animal safety, user safety, human food safety, environmental safety, side effects, permanency of treatment effect, and cost. Table 6 summarizes the feasibility of applying different sterilization methods to male livestock and pets.

**Table 6:** Comparison of sterilization methods for their application in farm and companion animals.

Method	Efficacy	Animal welfare concerns	Feasibility	Other concerns
Farm animals				
Surgical testis removal	Reproductive behavior Androgen production Fertility Permanent	Pain and stress due to absence of anesthesia or analgesia	Feasible: heavily used in current practice	Risk to workers (injury with scalpels); Adverse effects on production yield; Increase in mortality
Vasectomy	Sperm in ejaculate Fertility Permanent	No concerns if pain control is applied	Not feasible: time-consuming and relatively high cost; Used at small-scale to produce teaser males for estrus detection	Need trained surgeons; Retained androgen production and aggressive behavior
Systemic chemical sterilization	Unknown	Non-reproductive organ effects (usually high dose)	Not feasible: human food safety concerns (toxic residues)	May have only temporal effects at safe doses; May require repeated treatments
Chemical sterilization by injection into organ	Androgen production Sperm in ejaculate Fertility	Inflammation and pain	Feasible, if the best chemical solution is selected	Inconsistencies in treatment outcomes; May require training for optimal injection
Hormonal sterilization	Androgen production Fertility Transient	None or minor	Feasible, but requires repeated administration that increase costs	Human food safety and environmental safety; Inconsistencies in treatment outcomes
Pre-pubertal hormonal sterilization	Unknown	None or minor	Feasible: would require a single treatment	Human food safety and environmental safety; Effective treatment windows have to be defined for each species
Immunological sterilization	Reproductive behavior Androgen production Sperm in ejaculate Fertility Transient	None or minor	Feasible: popular alternative in hog production; Requires booster doses that increase costs	Risk to workers (self-injection); Handling of large animals as they age
Genetic sterilization	Reproductive behavior Androgen production Sperm in ejaculate Fertility Permanent	None or minor	Not feasible: high cost of development and implementation	Low consumer acceptance of genetically modified organisms
Companion animals				
Surgical testis removal	Reproductive behavior Androgen production Fertility Permanent	Risk of complications Stress before and after surgery	Feasible: heavily used in current practice; Logistic and economical limitations for use in free-roaming dog/cat communities	Time-consuming; Variable cost by region, breed, age, etc.; Debate over effects on lifespan; Selection of neutering age to minimize health impact; Retained reproductive behavior when performed at older ages
Vasectomy	Sperm in ejaculate Fertility Permanent	Risk of complications Stress before and after surgery	Feasible: popular alternative to surgical castration; Allows for positive effects of reproductive steroids on animal growth	Debate over effects of reproductive steroids on lifespan; Retained reproductive behaviors
Systemic chemical sterilization	Androgen production Sperm in ejaculate Fertility Transient	Non-reproductive organ effects (usually high dose)	Not feasible: animal welfare concerns	Temporal effects at safe doses; Requires repeated treatment;
Chemical sterilization by injection into organ	Androgen production Sperm in ejaculate Fertility Permanent	Inflammation and pain in the testis	Feasible, if the best chemical solution is selected	Inconsistencies in treatment outcomes; May require training for optimal injection
Hormonal sterilization	Reproductive behavior Androgen production Sperm in ejaculate Fertility Transient	Potential side effects on reproductive organs	Feasible: GnRH agonists are used in male dogs as contraceptives; Requires repeated administration	Temporal effects; Economical limitations
Pre-pubertal hormonal sterilization	Unknown	Potential side effects on reproductive organs development and growth	Feasible: would require a single treatment	Effective treatment windows have to be defined for each species
Immunological sterilization	Reproductive behavior Androgen production Sperm in ejaculate Fertility Transient	None or minor	Feasible, but would require booster doses; Potential conjugation with antigens for common diseases	Inconsistent results with high individual variability; Temporal effects
Genetic sterilization	Reproductive behavior Androgen production Sperm in ejaculate Fertility Permanent	None or minor	Feasible, but could be limited to unique breeds	Relatively high cost of development and implementation; Concerns regarding genetically modified organisms

## 4 Concluding remarks

For thousands of years, sterilization by castration has been a reliable method for managing reproduction in domestic and feral animals. In companion animals, this method has been necessary for controlling animal behavior and addressing the significant issue of animal abandonment and overpopulation. In livestock, male castration is widely used for improving animal management and the quality of the meat. However, in modern society, the practice has also heightened global awareness and concern for animal welfare. The ideal method for inducing animal sterility should not cause animal suffering or welfare concerns, such as side effects, its application should be cost- and time-efficient, and its effectiveness should be consistent regardless of the user. Additionally, neutering livestock must ensure safety for humans who may consume the meat. While some methods have shown potential to suppress reproduction in domesticated pets and food production animals over the long term, unfortunately, no alternative to surgical sterilization has yet fully met all these requirements. Nevertheless, research has led to successful chemical sterilization of rodent species. Hopefully, this discussion will inspire future breakthroughs in male sterilization, as we respond to the crisis of animal overpopulation and strive for better animal welfare.

## Author contributions

RH: Conceptualization, Funding acquisition, Investigation, Supervision, Visualization, Writing – original draft, Writing – review & editing. CP: Conceptualization, Funding acquisition, Investigation, Resources, Visualization, Writing – original draft, Writing – review & editing. SS: Conceptualization, Investigation, Writing – original draft, Writing – review & editing. LR: Investigation, Writing – review & editing. J-EO: Investigation, Writing – review & editing. MB: Investigation, Writing – review & editing. CK: Conceptualization, Funding acquisition, Resources, Writing – original draft, Writing – review & editing.

## References

[1] Adams VJ. Reproduction in dogs part 1: surgical and non-surgical de-sexing options. Companion Animal. (2020) 25:1–9. doi: 10.12968/coan.2020.0022

[2] Zink C Delgado MM Stella JL. Vasectomy and ovary-sparing spay in dogs: comparison of health and behavior outcomes with gonadectomized and sexually intact dogs. J Am Vet Med Assoc. (2023) 261:366–74. doi: 10.2460/javma.22.08.0382, PMID: 36656681

[3] Kutzler MA. Gonad-sparing surgical sterilization in dogs. Front Vet Sci. (2020) 7:342. doi: 10.3389/fvets.2020.00342 32596276 PMC7303261

[4] Waller D Bolick D Lissner E Premanandan C Gamerman G. Reversibility of Vasalgel™ male contraceptive in a rabbit model. Basic Clin Androl. (2017) 27:8. doi: 10.1186/s12610-017-0051-1, PMID: 28417005 PMC5381074

[5] Thirumalai A Amory JK. Emerging approaches to male contraception. Fertil Steril. (2021) 115:1369–76. doi: 10.1016/j.fertnstert.2021.03.047, PMID: 33931201 PMC8169637

[6] Morgentaler A Hanafy HM. The testis, eunuchs, and testosterone: a historical review over the ages and around the world. Sex Med Rev. (2023) 12:199–209. doi: 10.1093/sxmrev/qead051 38146670

[7] Reusch K. That which was missing: The archaeology of castration. Oxford: University of Oxford (2013).

[8] Cheney VT. A Brief History Of Castration. 2nd ed. Bloomington, IN: AuthorHouse (2006).

[9] Marshall FH Hammond J. On the effects of complete and incomplete castration upon horn growth in herdwick sheep. J Physiol. (1914) 48:171–6. doi: 10.1113/jphysiol.1914.sp001654 16993274 PMC1420515

[10] Levine MA Bailey G Whitwell KE Jeffcott LB. Palaeopathology and horse domestication: the case of some Iron age horses horn the Altai Mountains, Siberia In: Bailey G Charles R Winder N, editors. Human Ecodynamics. Symposia of the association for environmental archaeology. Oxford: Oxbow Books (2000). 123–33.

[11] Price J Eager RA Welsh EM Waran NK. Current practice relating to equine castration in the UK. Res Vet Sci. (2005) 78:277–80. doi: 10.1016/j.rvsc.2004.09.009, PMID: 15766949

[12] Meyer WJIC Collier M. Physical and chemical castration of sex offenders: a review section a: links between biology and crime. J Offender Rehab. (1997) 25:1–18. doi: 10.1300/J076v25n03_01

[13] Ringrose K. Eunuchs in historical perspective. History. Compass. (2007) 5:495–506. doi: 10.1111/j.1478-0542.2006.00379.x

[14] Dale MS. Inside the world of the eunuch: a social history of the Emperor’s servants in Qing China. Hong Kong China: Paramount Printing Co., Ltd (2019). 223 p.

[15] Nacchia A Lombardo R Tubaro A De Nunzio C. From terror to treatment: a history of Human castration. Int J Urologic History. (2023) 2:67–75. doi: 10.53101/IJUH.2.2.01052306

[16] Bibi R Kazmi SF Pervaiz T Bashir R. The mental health issues of eunuchs in the context of social exclusion. J Pak Med Assoc. (2021) 71:578–84. doi: 10.47391/JPMA.887, PMID: 33941939

[17] Lee JY Cho KS. Chemical castration for sexual offenders: physicians' views. J Korean Med Sci. (2013) 28:171–2. doi: 10.3346/jkms.2013.28.2.171, PMID: 23401647 PMC3565125

[18] Berlin FS. Sex offender treatment and legislation. J Am Acad Psychiatry Law. (2003) 31:510–3. PMID: 14974807

[19] Choi E Buie J Camacho J Sharma P de Riese WTW. Evolution of androgen deprivation therapy (ADT) and its new emerging modalities in prostate Cancer: an update for practicing urologists, clinicians and medical providers. Res Rep Urol. (2022) 14:87–108. doi: 10.2147/RRU.S303215, PMID: 35386270 PMC8977476

[20] Asa CS. Contraception in dogs and cats. Vet Clin North Am Small Anim Pract. (2018) 48:733–42. doi: 10.1016/j.cvsm.2018.02.014, PMID: 29685519

[21] Asa C Moresco A. Fertility Control in wildlife: review of current status, including novel and future technologies. Adv Exp Med Biol. (2019) 1200:507–43. doi: 10.1007/978-3-030-23633-5_17, PMID: 31471808

[22] Huggins C. Effect of orchiectomy and irradiation on CANCER of the prostate. Ann Surg. (1942) 115:1192–200. doi: 10.1097/00000658-194206000-00030, PMID: 17858048 PMC1543858

[23] Howe LM. Current perspectives on the optimal age to spay/castrate dogs and cats. Vet Med. (2015) 6:171–80. doi: 10.2147/VMRR.S53264, PMID: 30101104 PMC6070019

[24] Hoffman JM Creevy KE Promislow DE. Reproductive capability is associated with lifespan and cause of death in companion dogs. PLoS One. (2013) 8:e61082. doi: 10.1371/journal.pone.0061082, PMID: 23613790 PMC3629191

[25] Joonè CJ Konovalov DA. The effect of neuter status on longevity in the Rottweiler dog. Sci Rep. (2023) 13:17845. doi: 10.1038/s41598-023-45128-w, PMID: 37857755 PMC10587138

[26] Pedersen LJ. Chapter 1 – overview of commercial pig production systems and their main welfare challenges In: Špinka M, editor. Advances in pig welfare. Sawston: Woodhead Publishing (2018). 3–25.

[27] Bonneau M Weiler U. Pros and cons of alternatives to piglet castration: welfare, boar taint, and other meat quality traits. Animals (Basel). (2019) 9:884. doi: 10.3390/ani9110884, PMID: 31671665 PMC6912452

[28] Kustritz MV. Determining the optimal age for gonadectomy of dogs and cats. J Am Vet Med Assoc. (2007) 231:1665–75. doi: 10.2460/javma.231.11.1665, PMID: 18052800

[29] Olson PN Kustritz MV Johnston SD. Early-age neutering of dogs and cats in the United States (a review). J Reprod Fertil Suppl. (2001) 57:223–32. PMID: 11787153

[30] Yates D Leedham R. Prepubertal neutering of dogs — some risks and benefits. Companion Anim. (2019) 24:38–42. doi: 10.12968/coan.2019.24.1.38

[31] Cooley DM Beranek BC Schlittler DL Glickman NW Glickman LT Waters DJ. Endogenous gonadal hormone exposure and bone sarcoma risk. Cancer Epidemiol Biomarkers Prev. (2002) 11:1434–40. 12433723

[32] Salmeri KR Bloomberg MS Scruggs SL Shille V. Gonadectomy in immature dogs: effects on skeletal, physical, and behavioral development. J Am Vet Med Assoc. (1991) 198:1193–203. doi: 10.2460/javma.1991.198.07.1193, PMID: 2045340

[33] Herron MA. A potential consequence of prepubercal feline castration. Feline Pract. (1971) 1:17–9.

[34] Herron MA. The effect of prepubertal castration on the penile urethra of the cat. J Am Vet Med Assoc. (1972) 160:208–11. PMID: 5017757

[35] Hendry JA Homer RF Rose FL Walpole AL. Cytotoxic agents: III, derivatives of ethyleneimine. Br J Pharmacol Chemother. (1951) 6:357–410. doi: 10.1111/j.1476-5381.1951.tb00650.x, PMID: 14878975 PMC1509131

[36] Hayes WJ Jr. The toxicology of chemosterilants. Bull World Health Organ. (1964) 31:721–36. PMID: 14278008 PMC2555129

[37] Kimbrough R Gaines TB. Toxicity of hexamethylphosphoramide in rats. Nature. (1966) 211:146–7. doi: 10.1038/211146a0 6007470

[38] Marsh RE Howard WE. Prospects of chemosterilant and genetic control of rodents. Bull World Health Organ. (1973) 48:309–16. PMID: 4583051 PMC2481060

[39] Kar AB. Chemical sterilization of male Rhesus monkeys. Endocrinology. (1961) 69:1116–9. doi: 10.1210/endo-69-6-1116 14453827

[40] Chatterjee SN Kar AB. Chemical sterilization of stray dogs. Indian Vet J. (1968) 45:649–54. PMID: 5751654

[41] Ericsson RJ. Male antifertility compounds: U-5897 as a rat chemosterilant. J Reprod Fert. (1970) 22:213–22. doi: 10.1530/jrf.0.0220213, PMID: 5428943

[42] Fahim MS Fahim Z Harman JM. Chemical sterilization in the male part I: rats. Arch Androl. (1982) 9:261–5. doi: 10.3109/01485018208990248, PMID: 7181552

[43] Fahim MS Wang M Sutcu MF Fahim Z Youngquist RS. Sterilization of dogs with intra-epididymal injection of zinc arginine. Contraception. (1993) 47:107–22. doi: 10.1016/0010-7824(93)90113-L, PMID: 8435998

[44] Freeman C Coffey DS. Sterility in male animals induced by injection of chemical agents into the vas deferens. Fertil Steril. (1973) 24:884–90. doi: 10.1016/S0015-0282(16)40036-1, PMID: 4742009

[45] Cavalieri J. Chemical sterilisation of animals: a review of the use of zinc- and CaCl(2) based solutions in male and female animals and factors likely to improve responses to treatment. Anim Reprod Sci. (2017) 181:1–8. doi: 10.1016/j.anireprosci.2017.03.010, PMID: 28366279

[46] Paranzini CS Sousa AK Cardoso GS Perencin FM Trautwein LGC Bracarense A. Effects of chemical castration using 20% CaCl(2) with 0.5% DMSO in tomcats: evaluation of inflammatory reaction by infrared thermography and effectiveness of treatment. Theriogenology. (2018) 106:253–8. doi: 10.1016/j.theriogenology.2017.10.013, PMID: 29096273

[47] SenesTech. (2023). Contrapest: The pest control difference. Available at: https://senestech.com/contrapest/

[48] Lacoste D Dubé D Trudel C Bélanger A Labrie F. Normal gonadal functions and fertility after 23 months of treatment of prepubertal male and female dogs with the GnRh agonist [D-Trp6, des-Gly-NH2(10)]GnRH ethylamide. J Androl. (1989) 10:456–65. doi: 10.1002/j.1939-4640.1989.tb00140.x, PMID: 2695507

[49] England GC. Effect of progestogens and androgens upon spermatogenesis and steroidogenesis in dogs. J Reprod Fertil Suppl. (1997) 51:123–38. PMID: 9404279

[50] Garcia Romero G Valiente C Aquilano D Corrada Y Gobello C. Endocrine effects of the GnRH antagonist, acyline, in domestic dogs. Theriogenology. (2009) 71:1234–7. doi: 10.1016/j.theriogenology.2008.12.017, PMID: 19193431

[51] Carranza A Faya M Merlo ML Batista P Gobello C. Effect of GnRH analogs in postnatal domestic cats. Theriogenology. (2014) 82:138–43. doi: 10.1016/j.theriogenology.2014.03.012, PMID: 24725419

[52] Lucas X. Clinical use of Deslorelin (GnRH agonist) in companion Animals: a review. Reprod Domest Anim. (2014) 49:64–71. doi: 10.1111/rda.12388, PMID: 25277434

[53] Glocova K Cizek P Novotny R Hauptman K Tichy F. Effect of GnRH agonist deslorelin implant on spermatogenesis and testosterone concentration in Guinea pigs (*Cavia aperea* porcellus). Theriogenology. (2020) 154:232–6. doi: 10.1016/j.theriogenology.2020.05.038, PMID: 32679355

[54] Knol BW Egberink-Alink ST. Treatment of problem behaviour in dogs and cats by castration and progestagen administration: a review. Vet Q. (1989) 11:102–7. doi: 10.1080/01652176.1989.9694206 2662568

[55] Amory JK. Testosterone/progestin regimens: a realistic option for male contraception? Curr Opin Investig Drugs. (2004) 5:1025–30. PMID: 15535423

[56] Ilani N Roth MY Amory JK Swerdloff RS Dart C Page ST. A new combination of testosterone and nestorone transdermal gels for male hormonal contraception. J Clin Endocrinol Metab. (2012) 97:3476–86. doi: 10.1210/jc.2012-1384, PMID: 22791756 PMC3462927

[57] Park CJ Minabe S Hess RA Lin P-CP Zhou S Bashir ST. Single neonatal estrogen implant sterilizes female animals by decreasing hypothalamic KISS1 expression. Sci Rep. (2023) 13:9627. doi: 10.1038/s41598-023-36727-8, PMID: 37316510 PMC10267159

[58] Kaprara A Huhtaniemi IT. The hypothalamus-pituitary-gonad axis: Tales of mice and men. Metabolism. (2018) 86:3–17. doi: 10.1016/j.metabol.2017.11.018, PMID: 29223677

[59] Lopez-Rodriguez D Franssen D Bakker J Lomniczi A Parent AS. Cellular and molecular features of EDC exposure: consequences for the GnRH network. Nat Rev Endocrinol. (2021) 17:83–96. doi: 10.1038/s41574-020-00436-3, PMID: 33288917

[60] Pinilla L Aguilar E Dieguez C Millar RP Tena-Sempere M. Kisspeptins and reproduction: physiological roles and regulatory mechanisms. Physiol Rev. (2012) 92:1235–316. doi: 10.1152/physrev.00037.2010 22811428

[61] Dhillo WS. Kisspeptin: a novel regulator of reproductive function. J Neuroendocrinol. (2008) 20:963–70. doi: 10.1111/j.1365-2826.2008.01753.x 18510709

[62] Novaira HJ Sonko ML Radovick S. Kisspeptin induces dynamic chromatin modifications to Control GnRH gene expression. Mol Neurobiol. (2016) 53:3315–25. doi: 10.1007/s12035-015-9269-0, PMID: 26081144

[63] Novaira HJ Fadoju D Diaczok D Radovick S. Genetic mechanisms mediating kisspeptin regulation of GnRH gene expression. J Neurosci. (2012) 32:17391–400. doi: 10.1523/JNEUROSCI.2438-12.2012, PMID: 23197730 PMC3534770

[64] Smith JT Cunningham MJ Rissman EF Clifton DK Steiner RA. Regulation of Kiss1 gene expression in the brain of the female mouse. Endocrinology. (2005) 146:3686–92. doi: 10.1210/en.2005-0488 15919741

[65] Rometo AM Krajewski SJ Voytko ML Rance NE. Hypertrophy and increased kisspeptin gene expression in the hypothalamic infundibular nucleus of postmenopausal women and ovariectomized monkeys. J Clin Endocrinol Metab. (2007) 92:2744–50. doi: 10.1210/jc.2007-0553, PMID: 17488799

[66] Han SY Kane G Cheong I Herbison AE. Characterization of GnRH pulse generator activity in male mice using GCaMP Fiber photometry. Endocrinology. (2019) 160:557–67. doi: 10.1210/en.2018-01047, PMID: 30649269

[67] McQuillan HJ Han SY Cheong I Herbison AE. GnRH pulse generator activity across the estrous cycle of female mice. Endocrinology. (2019) 160:1480–91. doi: 10.1210/en.2019-00193, PMID: 31083714

[68] Clarkson J Han SY Piet R McLennan T Kane GM Ng J. Definition of the hypothalamic GnRH pulse generator in mice. Proc Natl Acad Sci USA. (2017) 114:E10216–23. doi: 10.1073/pnas.1713897114 29109258 PMC5703322

[69] Belchetz PE Plant TM Nakai Y Keogh EJ Knobil E. Hypophysial responses to continuous and intermittent delivery of hypopthalamic gonadotropin-releasing hormone. Science. (1978) 202:631–3. doi: 10.1126/science.100883 100883

[70] Han SY Cheong I McLennan T Herbison AE. Neural determinants of pulsatile luteinizing hormone secretion in male mice. Endocrinology. (2020) 161:bqz045. doi: 10.1210/endocr/bqz045 31907531

[71] Clarkson J Herbison AE. Postnatal development of kisspeptin neurons in mouse hypothalamus; sexual dimorphism and projections to gonadotropin-releasing hormone neurons. Endocrinology. (2006) 147:5817–25. doi: 10.1210/en.2006-0787, PMID: 16959837 PMC6098691

[72] Marraudino M Miceli D Farinetti A Ponti G Panzica G Gotti S. Kisspeptin innervation of the hypothalamic paraventricular nucleus: sexual dimorphism and effect of estrous cycle in female mice. J Anat. (2017) 230:775–86. doi: 10.1111/joa.12603, PMID: 28295274 PMC5442148

[73] Lapatto R Pallais JC Zhang D Chan YM Mahan A Cerrato F. Kiss1−/− mice exhibit more variable hypogonadism than Gpr54−/− mice. Endocrinology. (2007) 148:4927–36. doi: 10.1210/en.2007-0078, PMID: 17595229

[74] Funes S Hedrick JA Vassileva G Markowitz L Abbondanzo S Golovko A. The KiSS-1 receptor GPR54 is essential for the development of the murine reproductive system. Biochem Biophys Res Commun. (2003) 312:1357–63. doi: 10.1016/j.bbrc.2003.11.066, PMID: 14652023

[75] Messager S Chatzidaki EE Ma D Hendrick AG Zahn D Dixon J. Kisspeptin directly stimulates gonadotropin-releasing hormone release via G protein-coupled receptor 54. Proc Natl Acad Sci USA. (2005) 102:1761–6. doi: 10.1073/pnas.0409330102, PMID: 15665093 PMC545088

[76] d'Anglemont de Tassigny X Fagg LA Dixon JP Day K Leitch HG Hendrick AG. Hypogonadotropic hypogonadism in mice lacking a functional Kiss1 gene. Proc Natl Acad Sci USA. (2007) 104:10714–9. doi: 10.1073/pnas.0704114104, PMID: 17563351 PMC1965578

[77] Novaira HJ Sonko ML Hoffman G Koo Y Ko C Wolfe A. Disrupted kisspeptin signaling in GnRH neurons leads to hypogonadotrophic hypogonadism. Mol Endocrinol. (2014) 28:225–38. doi: 10.1210/me.2013-1319, PMID: 24422632 PMC3896637

[78] Seminara SB Messager S Chatzidaki EE Thresher RR Acierno JS Jr Shagoury JK. The GPR54 gene as a regulator of puberty. N Engl J Med. (2003) 349:1614–27. doi: 10.1056/NEJMoa035322 14573733

[79] Minabe S Sato M Inoue N Watanabe Y Magata F Matsuda F. Neonatal estrogen causes irreversible male infertility via specific suppressive action on hypothalamic Kiss1 neurons. Endocrinology. (2019) 160:1223–33. doi: 10.1210/en.2018-00732, PMID: 30920587

[80] Minabe S Ieda N Watanabe Y Inoue N Uenoyama Y Maeda KI. Long-term neonatal estrogen exposure causes irreversible inhibition of LH pulses by suppressing arcuate Kisspeptin expression via estrogen receptors alpha and beta in female rodents. Endocrinology. (2017) 158:2918–29. doi: 10.1210/en.2016-1144, PMID: 28368450

[81] Semaan SJ Murray EK Poling MC Dhamija S Forger NG Kauffman AS. BAX-dependent and BAX-independent regulation of Kiss1 neuron development in mice. Endocrinology. (2010) 151:5807–17. doi: 10.1210/en.2010-0783, PMID: 20926580 PMC2999490

[82] Kauffman AS Park JH McPhie-Lalmansingh AA Gottsch ML Bodo C Hohmann JG. The kisspeptin receptor GPR54 is required for sexual differentiation of the brain and behavior. J Neurosci. (2007) 27:8826–35. doi: 10.1523/JNEUROSCI.2099-07.2007, PMID: 17699664 PMC6672184

[83] Nakamura S Watanabe Y Goto T Ikegami K Inoue N Uenoyama Y. Kisspeptin neurons as a key player bridging the endocrine system and sexual behavior in mammals. Front Neuroendocrinol. (2022) 64:100952. doi: 10.1016/j.yfrne.2021.100952, PMID: 34755641

[84] Adekunbi DA Li XF Lass G Shetty K Adegoke OA Yeo SH. Kisspeptin neurones in the posterodorsal medial amygdala modulate sexual partner preference and anxiety in male mice. J Neuroendocrinol. (2018) 30:e12572. doi: 10.1111/jne.12572, PMID: 29356147 PMC5873280

[85] Mills EGA O'Byrne KT Comninos AN. The roles of the amygdala Kisspeptin system. Semin Reprod Med. (2019) 37:64–70. doi: 10.1055/s-0039-3400462, PMID: 31847026

[86] Kim J Semaan SJ Clifton DK Steiner RA Dhamija S Kauffman AS. Regulation of Kiss1 expression by sex steroids in the amygdala of the rat and mouse. Endocrinology. (2011) 152:2020–30. doi: 10.1210/en.2010-1498, PMID: 21363930 PMC3075940

[87] Yang JJ Caligioni CS Chan YM Seminara SB. Uncovering novel reproductive defects in neurokinin B receptor null mice: closing the gap between mice and men. Endocrinology. (2012) 153:1498–508. doi: 10.1210/en.2011-1949, PMID: 22253416 PMC3281542

[88] Rumpler E Takacs S Gocz B Baska F Szenci O Horvath A. Kisspeptin neurons in the infundibular nucleus of Ovariectomized cats and dogs exhibit unique anatomical and neurochemical characteristics. Front Neurosci. (2020) 14:598707. doi: 10.3389/fnins.2020.598707, PMID: 33343288 PMC7738562

[89] Hoffmann HM Trang C Gong P Kimura I Pandolfi EC Mellon PL. Deletion of Vax1 from gonadotropin-releasing hormone (GnRH) neurons abolishes GnRH expression and leads to hypogonadism and infertility. J Neurosci. (2016) 36:3506–18. doi: 10.1523/JNEUROSCI.2723-15.2016, PMID: 27013679 PMC4804008

[90] Perrett RM McArdle CA. Molecular mechanisms of gonadotropin-releasing hormone signaling: integrating cyclic nucleotides into the network. Front Endocrinol (Lausanne). (2013) 4:180. doi: 10.3389/fendo.2013.00180 24312080 PMC3834291

[91] Goericke-Pesch S. Long-term effects of GnRH agonists on fertility and behaviour. Reprod Domest Anim. (2017) 52:336–47. doi: 10.1111/rda.12898, PMID: 28025851

[92] Driancourt MA Briggs JR. Gonadotropin-releasing hormone (GnRH) agonist implants for male dog fertility suppression: a review of mode of action, efficacy, safety, and uses. Front Vet Sci. (2020) 7:483. doi: 10.3389/fvets.2020.00483, PMID: 32923467 PMC7456901

[93] Mann DR Smith MM Gould KG Collins DC. Effect of a gonadotropin-releasing hormone agonist on luteinizing hormone and testosterone secretion and testicular histology in male rhesus monkeys. Fertil Steril. (1985) 43:115–21. doi: 10.1016/S0015-0282(16)48328-7, PMID: 3880709

[94] Gonzalez-Bulnes A Souza CJ Scaramuzzi RJ Campbell BK Baird DT. Long-term suppression of reproductive function by a single dose of gonadotropin-releasing hormone antagonists in a sheep model. Fertil Steril. (2006) 86:1121–8. doi: 10.1016/j.fertnstert.2006.02.104 16952359

[95] Huhtaniemi I. Mechanisms in endocrinology: hormonal regulation of spermatogenesis: mutant mice challenging old paradigms. Eur J Endocrinol. (2018) 179:R143–r150. doi: 10.1530/EJE-18-0396, PMID: 29959220

[96] Oduwole OO Huhtaniemi IT Misrahi M. The roles of luteinizing hormone, follicle-stimulating hormone and testosterone in spermatogenesis and Folliculogenesis revisited. Int J Mol Sci. (2021) 22:12735. doi: 10.3390/ijms222312735, PMID: 34884539 PMC8658012

[97] Stanić D Dubois S Chua HK Tonge B Rinehart N Horne MK. Characterization of aromatase expression in the adult male and female mouse brain. I. Coexistence with Oestrogen receptors α and β, and androgen receptors. PLoS One. (2014) 9:e90451. doi: 10.1371/journal.pone.0090451 24646567 PMC3960106

[98] Smith JT Dungan HM Stoll EA Gottsch ML Braun RE Eacker SM. Differential regulation of KiSS-1 mRNA expression by sex steroids in the brain of the male mouse. Endocrinology. (2005) 146:2976–84. doi: 10.1210/en.2005-0323, PMID: 15831567

[99] Oakley AE Clifton DK Steiner RA. Kisspeptin signaling in the brain. Endocr Rev. (2009) 30:713–43. doi: 10.1210/er.2009-0005, PMID: 19770291 PMC2761114

[100] Anawalt BD Bebb RA Matsumoto AM Groome NP Illingworth PJ McNeilly AS. Serum inhibin B levels reflect Sertoli cell function in normal men and men with testicular dysfunction. J Clin Endocrinol Metab. (1996) 81:3341–5. PMID: 8784094 10.1210/jcem.81.9.8784094

[101] Hess RA de Franca LR. Spermatogenesis and cycle of the seminiferous epithelium. Adv Exp Med Biol. (2008) 636:1–15. doi: 10.1007/978-0-387-09597-4_1, PMID: 19856159

[102] Hess RA Vogl AW. 1 – Sertoli cell anatomy and cytoskeleton In: Griswold M, editor. Sertoli Cell Biology. Oxford: Elsevier Academic Press (2015). 1–55.

[103] Smith BE Braun RE. Germ cell migration across Sertoli cell tight junctions. Science. (2012) 338:798–802. doi: 10.1126/science.1219969, PMID: 22997133 PMC3694388

[104] Mruk DD Cheng CY. Tight junctions in the testis: new perspectives. Philos Trans R Soc Lond Ser B Biol Sci. (2010) 365:1621–35. doi: 10.1098/rstb.2010.0010, PMID: 20403874 PMC2871926

[105] Cheng CY Mruk DD. The blood-testis barrier and its implications for male contraception. Pharmacol Rev. (2012) 64:16–64. doi: 10.1124/pr.110.002790, PMID: 22039149 PMC3250082

[106] Mruk DD Cheng CY. The mammalian blood-testis barrier: its biology and regulation. Endocr Rev. (2015) 36:564–91. doi: 10.1210/er.2014-1101, PMID: 26357922 PMC4591527

[107] Wong EWP Yan HHN Li MWM Lie PPY Mruk DD Cheng CY. Cell junctions in the testis as targets for toxicants In: McQueen CA, editor. Comprehensive toxicology. Amsterdam: Elsevier Science (2018). 167–88.

[108] Vogl AW Young JS Du M. New insights into roles of tubulobulbar complexes in sperm release and turnover of blood-testis barrier. Int Rev Cell Mol Biol. (2013) 303:319–55. doi: 10.1016/B978-0-12-407697-6.00008-8, PMID: 23445814

[109] O’Donnell L Stanton PG. Spermiation In: Skinner MK, editor. Encyclopedia of reproduction. Oxford: Academic Press (2018). 145–51.

[110] O'Donnell L Smith LB Rebourcet D. Sertoli cells as key drivers of testis function. Semin Cell Dev Biol. (2021) 121:2–9. doi: 10.1016/j.semcdb.2021.06.016 34229950

[111] Oduwole OO Peltoketo H Huhtaniemi IT. Role of follicle-stimulating hormone in spermatogenesis. Front Endocrinol. (2018) 9:763. doi: 10.3389/fendo.2018.00763, PMID: 30619093 PMC6302021

[112] O'Shaughnessy PJ. Hormonal control of germ cell development and spermatogenesis. Semin Cell Dev Biol. (2014) 29:55–65. doi: 10.1016/j.semcdb.2014.02.010 24598767

[113] Chang Z Qin W Zheng H Schegg K Han L Liu X. Triptonide is a reversible non-hormonal male contraceptive agent in mice and non-human primates. Nat Commun. (2021) 12:1253. doi: 10.1038/s41467-021-21517-5, PMID: 33623031 PMC7902613

[114] Saito K O'Donnell L McLachlan RI Robertson DM. Spermiation failure is a major contributor to early spermatogenic suppression caused by hormone withdrawal in adult rats. Endocrinology. (2000) 141:2779–85. doi: 10.1210/endo.141.8.7628, PMID: 10919263

[115] Li L Tang EI Chen H Lian Q Ge R Silvestrini B. Sperm release at spermiation is regulated by changes in the organization of actin- and microtubule-based cytoskeletons at the apical ectoplasmic specialization – a study using the adjudin model. Endocrinology. (2017) 158:4300–16. doi: 10.1210/en.2017-00660, PMID: 29040437 PMC5711386

[116] Buaas FW Kirsh AL Sharma M McLean DJ Morris JL Griswold MD. Plzf is required in adult male germ cells for stem cell self-renewal. Nat Genet. (2004) 36:647–52. doi: 10.1038/ng1366 15156142

[117] Chen C Ouyang W Grigura V Zhou Q Carnes K Lim H. ERM is required for transcriptional control of the spermatogonial stem cell niche. Nature. (2005) 436:1030–4. doi: 10.1038/nature03894, PMID: 16107850 PMC2909764

[118] Hofmann MC McBeath E. Sertoli cell-germ cell interactions within the niche: paracrine and Juxtacrine molecular communications. Front Endocrinol (Lausanne). (2022) 13:897062. doi: 10.3389/fendo.2022.897062, PMID: 35757413 PMC9226676

[119] Law NC Oatley JM. Spermatogonial stem cell and niche In: Skinner MK, editor. Encyclopedia of reproduction. Oxford: Academic Press (2018). 117–23.

[120] Liu Y Zhang Y. ETV5 is essential for neuronal differentiation of Human neural progenitor cells by repressing NEUROG2 expression. Stem Cell Rev Rep. (2019) 15:703–16. doi: 10.1007/s12015-019-09904-4, PMID: 31273540

[121] Zhang R Boareto M Engler A Louvi A Giachino C Iber D. Id4 downstream of Notch2 maintains neural stem cell quiescence in the adult Hippocampus. Cell Rep. (2019) 28:1485–1498.e6. doi: 10.1016/j.celrep.2019.07.014, PMID: 31390563

[122] Baloh RH Johnson JP Avalos P Allred P Svendsen S Gowing G. Transplantation of human neural progenitor cells secreting GDNF into the spinal cord of patients with ALS: a phase 1/2a trial. Nat Med. (2022) 28:1813–22. doi: 10.1038/s41591-022-01956-3, PMID: 36064599 PMC9499868

[123] Zirkin BR Papadopoulos V. Leydig cells: formation, function, and regulation. Biol Reprod. (2018) 99:101–11. doi: 10.1093/biolre/ioy059, PMID: 29566165 PMC6044347

[124] Cooke PS Nanjappa MK Ko C Prins GS Hess RA. Estrogens in male physiology. Physiol Rev. (2017) 97:995–1043. doi: 10.1152/physrev.00018.2016, PMID: 28539434 PMC6151497

[125] Huhtaniemi I Teerds K. Leydig Cells In: Skinner MK, editor. Encyclopedia of reproduction. Oxford: Academic Press (2018). 30–8.

[126] Cao C Ma Q Mo S Shu G Liu Q Ye J. Single-cell RNA sequencing defines the regulation of spermatogenesis by Sertoli-cell androgen signaling. Front Cell Dev Biol. (2021) 9:763267. doi: 10.3389/fcell.2021.763267, PMID: 34869354 PMC8634442

[127] Culty M Papadopoulos V Zirkin B. Leydig cells: fetal to aged testes In: Skinner MK, editor. Encyclopedia of reproduction. Oxford: Academic Press (2018). 39–41.

[128] Li L Zirkin BR Papadopoulos V. Leydig cell androgen synthesis In: Skinner MK, editor. Encyclopedia of reproduction. Oxford: Academic Press (2018). 215–21.

[129] de Kretser DM. Hypothalamic pituitary testis Axis In: Skinner MK, editor. Encyclopedia of reproduction. Oxford: Academic Press (2018). 180–3.

[130] Sullivan R Legare C Lamontagne-Proulx J Breton S Soulet D. Revisiting structure/functions of the human epididymis. Andrology. (2019) 7:748–57. doi: 10.1111/andr.12633, PMID: 31033221

[131] Hess RA. Efferent ductules: structure and function In: Skinner MK, editor. Encyclopedia of reproduction. Oxford: Academic Press (2018). 270–8.

[132] Hess RA. Endocrinology and pathology of rete testis and efferent ductules In: Skinner MK, editor. Encyclopedia of reproduction. Oxford: Academic Press (2018). 279–85.

[133] Hess RA. Disruption of estrogen receptor signaling and similar pathways in the efferent ductules and initial segments of the epididymis. Spermatogenesis. (2014) 4:e979103. doi: 10.4161/21565562.2014.979103, PMID: 26413389 PMC4581051

[134] Ilio KY Hess RA. Structure and function of the ductuli efferentes: a review. Microsc Res Tech. (1994) 29:432–67. doi: 10.1002/jemt.1070290604, PMID: 7873793

[135] Yuan S Liu Y Peng H Tang C Hennig GW Wang Z. Motile cilia of the male reproductive system require miR-34/miR-449 for development and function to generate luminal turbulence. Proc Natl Acad Sci USA. (2019) 116:3584–93. doi: 10.1073/pnas.1817018116, PMID: 30659149 PMC6397547

[136] Hess RA. The efferent ductules: structure and functions In: Robaire B Hinton B, editors. The epididymis: From molecules to clinical practice. New York: Springer (2002). 49–80.

[137] Clulow J Jones RC Hansen LA Man SY. Fluid and electrolyte reabsorption in the ductuli efferentes testis. J Reprod Fertil Suppl. (1998) 53:1–14. PMID: 10645261

[138] Joseph A Shur BD Ko C Chambon P Hess RA. Epididymal hypo-osmolality induces abnormal sperm morphology and function in the estrogen receptor alpha knockout mouse. Biol Reprod. (2010) 82:958–67. doi: 10.1095/biolreprod.109.080366, PMID: 20130266 PMC2857636

[139] Hess RA Bunick D Lee KH Bahr J Taylor JA Korach KS. A role for oestrogens in the male reproductive system. Nature. (1997) 390:509–12. doi: 10.1038/37352 9393999 PMC5719867

[140] Joseph A Hess RA Schaeffer DJ Ko C Hudgin-Spivey S Chambon P. Absence of estrogen receptor alpha leads to physiological alterations in the mouse epididymis and consequent defects in sperm function. Biol Reprod. (2010) 82:948–57. doi: 10.1095/biolreprod.109.079889, PMID: 20130267 PMC2857635

[141] Reynolds-Wright JJ Anderson RA. Male contraception: where are we going and where have we been? BMJ Sex Reproductive Health. (2019) 45:236–42. doi: 10.1136/bmjsrh-2019-200395, PMID: 31537614 PMC6892591

[142] Drevet JR. Epididymal approaches to male contraception. Basic Clin Androl. (2018) 28:12. doi: 10.1186/s12610-018-0078-y, PMID: 30410765 PMC6219163

[143] Jelinsky SA Turner TT Bang HJ Finger JN Solarz MK Wilson E. The rat epididymal transcriptome: comparison of segmental gene expression in the rat and mouse epididymides. Biol Reprod. (2007) 76:561–70. doi: 10.1095/biolreprod.106.057323, PMID: 17167166

[144] Breton S Nair AV Battistone MA. Epithelial dynamics in the epididymis: role in the maturation, protection, and storage of spermatozoa. Andrology. (2019) 7:631–43. doi: 10.1111/andr.12632, PMID: 31044554 PMC6688936

[145] Girardet L Cyr DG Belleannée C. Arl13b controls basal cell stemness properties and hedgehog signaling in the mouse epididymis. Cell Mol Life Sci. (2022) 79:556. doi: 10.1007/s00018-022-04570-1, PMID: 36261680 PMC11803030

[146] Da Silva N Barton CR. Macrophages and dendritic cells in the post-testicular environment. Cell Tissue Res. (2016) 363:97–104. doi: 10.1007/s00441-015-2270-0, PMID: 26337514 PMC4703462

[147] Sullivan R Belleannée C. Cell biology of the epididymis In: Skinner MK, editor. Encyclopedia of reproduction. Oxford: Academic Press (2018). 286–91.

[148] Silber SJ. Vasectomy In: Knobil E Neill JD, editors. Encyclopedia of reproduction. San Diego: Academic Press (1998). 977–85.

[149] Libermann S Martin KW Fransson BA. Laparoscopic castration and sterilization techniques of the male dog In: Fransson BA, Mayhew PD, editors. Small animal laparoscopy and Thoracoscopy. Hoboken, NJ: Wiley (2022). 267–75. doi: 10.1002/9781119666912.ch23

[150] McCarthy RJ Levine SH Reed JM. Estimation of effectiveness of three methods of feral cat population control by use of a simulation model. J Am Vet Med Assoc. (2013) 243:502–11. doi: 10.2460/javma.243.4.502, PMID: 23902443

[151] Kutzler MA. Intratesticular and intraepididymal injections to sterilize male cats: from calcium chloride to zinc gluconate and beyond. J Feline Med Surg. (2015) 17:772–6. doi: 10.1177/1098612X15594991, PMID: 26323801 PMC11148979

[152] Urfer SR Kaeberlein M. Desexing dogs: a review of the current literature. Animals (Basel). (2019) 9:1086. doi: 10.3390/ani9121086 31817504 PMC6940997

[153] Howe LM. Surgical methods of contraception and sterilization. Theriogenology. (2006) 66:500–9. doi: 10.1016/j.theriogenology.2006.04.005 16716381

[154] Root Kustritz MV. Population Control in small Animals. Vet Clin North Am Small Anim Pract. (2018) 48:721–32. doi: 10.1016/j.cvsm.2018.02.013 29656769

[155] Howe LM Slater MR Boothe HW Hobson HP Holcom JL Spann AC. Long-term outcome of gonadectomy performed at an early age or traditional age in dogs. J Am Vet Med Assoc. (2001) 218:217–21. doi: 10.2460/javma.2001.218.217 11195826

[156] Ferré-Dolcet L Romagnoli S. Reversible control of reproduction in tom cats: medical options for manipulating libido and fertility. J Feline Med Surg. (2023) 25:1098612X2311714. doi: 10.1177/1098612X231171406 PMC10811986 37158289

[157] Kutzler M. Benign prostatic hyperplasia in small Animals In: Line S, editor. The Merck veterinary manual. Whitehouse Station, NJ: Merck & Co., Inc. (2019)

[158] Marvel SJ. Concepts in sterilization. Vet Clin North Am Small Anim Pract. (2022) 52:419–36. doi: 10.1016/j.cvsm.2021.11.003 35082093

[159] Kutzler MA. Possible relationship between long-term adverse health effects of gonad-removing surgical sterilization and luteinizing hormone in dogs. Animals (Basel). (2020) 10:599. doi: 10.3390/ani10040599 32244716 PMC7222805

[160] Picut CA Remick AK. Impact of age on the male reproductive system from the Pathologist’s perspective. Toxicol Pathol. (2017) 45:195–205. doi: 10.1177/0192623316672744, PMID: 27856969

[161] Gob CS. Age of neutering in large and Giant breed dogs. Clinicians Brief. (2016):19–23.

[162] Yamada PH Codognoto VM Rydygier de Ruediger F Mayara da Silva K Aristizábal VV Kastelic JP. A comparison of immunological, chemical and surgical castration of Nelore bulls. Theriogenology. (2021) 169:9–13. doi: 10.1016/j.theriogenology.2021.03.021, PMID: 33887521

[163] Marquette GA Ronan S Earley B. Review: castration – animal welfare considerations. J Appl Anim Res. (2023) 51:703–18. doi: 10.1080/09712119.2023.2273270

[164] Morales J Dereu A Manso A de Frutos L Pineiro C Manzanilla EG. Surgical castration with pain relief affects the health and productive performance of pigs in the suckling period. Porcine Health Manag. (2017) 3:18. doi: 10.1186/s40813-017-0066-1, PMID: 28879020 PMC5585944

[165] AVMA Literature review on the welfare implications of castration of cattle. (2014). 1–10.

[166] AVMA Welfare implications of swine castration. (2013). 1–5. Available at: https://www.avma.org/KB/Resources/LiteratureReviews/Documents/swine_castration_bgnd.pdf

[167] Thun R Gajewski Z Janett F. Castration in male pigs: techniques and animal welfare issues. J Physiol Pharmacol. (2006) 57:189–94. 17242482

[168] Weiler U Bonneau M. Why it is so difficult to end surgical castration of boars in Europe: pros and cons of alternatives to piglet castration. IOP Conf Series. (2019) 333:012001. doi: 10.1088/1755-1315/333/1/012001

[169] Silva E Schumacher J Passler T. Castration of dogs using local anesthesia after sedating with Xylazine and subanesthetic doses of ketamine. Front Vet Sci. (2020) 6:478. doi: 10.3389/fvets.2019.00478 32039245 PMC6989469

[170] Ting STL Earley B Veissier I Gupta S Crowe MA. Effects of Burdizzo castration on CO2 laser induced thermal nociception of Holstein–Friesian calves of different ages. Appl Anim Behav Sci. (2010) 126:12–8. doi: 10.1016/j.applanim.2010.05.005

[171] Baldwin CM. A review of prevention and management of castration complications. Equine Vet Educ. (2024) 36:97–106. doi: 10.1111/eve.13880

[172] Garcia A. Towards an improved method of piglet castration to reduce pain: the use of one incision in combination with the use of a Vapocoolant and Metacam™ (C-17-037) In: Pork checkoff. Des Moines, IA: National Port Board (2019). 1–17. doi: 10.5772/intechopen.68650

[173] Čandek-Potokar M Škrlep M Zamaratskaia G. Immunocastration as alternative to surgical Castrationin pigs. Theriogenology. (2017) 6:109–26.

[174] Naz RK Saver AE. Immunocontraception for Animals: current status and future perspective. Am J Reprod Immunol. (2016) 75:426–39. doi: 10.1111/aji.12431, PMID: 26412331

[175] Čandek-Potokar M Batorek-Lukac N. Alternatives to surgical castration of pigs. Životnov Dni Nauki. (2015) 52:41–51.

[176] Green P. Castration techniques in the horse. In Pract. (2001) 23:250–61. doi: 10.1136/inpract.23.5.250

[177] Kilcoyne I Spier SJ. Castration complications: a review of castration techniques and how to manage complications. Vet Clin North Am Equine Pract. (2021) 37:259–73. doi: 10.1016/j.cveq.2021.04.002 34116922

[178] Moll HD Pelzer KD Pleasant RS Modransky PD May KA. A survey of equine castration complications. J Equine Vet. (1995) 15:522–6. doi: 10.1016/S0737-0806(07)80421-7

[179] Hassan A Fromsa A. Review on chemical sterilization of male dogs. Int J Adv Res. (2017) 5:758–70. doi: 10.21474/IJAR01/5828

[180] Massei G Miller LA. Nonsurgical fertility control for managing free-roaming dog populations: a review of products and criteria for field applications. Theriogenology. (2013) 80:829–38. doi: 10.1016/j.theriogenology.2013.07.016 23998740

[181] Başa A. Canpolat I., Chemical sterilization in domestic animals. (2019): p. 5–9.

[182] Fesseha H. Non-surgical sterilization methods in male animals: a review. Vet Med Open J. (2019) 4:49–56. doi: 10.17140/VMOJ-4-136

[183] Seid AM Terefe DA. A review on chemical castration methods to control stray dog population. Online J Anim Feed Res. (2019) 9:233–40. doi: 10.36380/scil.2019.ojafr32

[184] Vasetska A. Non-surgical methods of regulation reproductive function and contraception males of domestic animals. Ukrainian J Vet Agric Sci. (2020) 3:44–50. doi: 10.32718/ujvas3-3.09

[185] Gobello C. Options for contraception in female and male felids In: Johnson A Kutzler M, editors. Feline reproduction. Oxfordshire: CABI (2022). 251–6.

[186] Padodara RJ Singh VK Odedara AB Vasava AA Sharma AK Mehta VM. Modern approaches to contraception in domestic and wild animals: a review. J Global Ecol Environ. (2022) 16:14–25. doi: 10.56557/jogee/2022/v16i17622

[187] Rhodes L. New approaches to non-surgical sterilization for dogs and cats: opportunities and challenges. Reprod Domest Anim. (2017) 52:327–31. doi: 10.1111/rda.12862, PMID: 27892642

[188] Kumar R Soni N Kumar S Pandey AK. Chemical Control of fertility in male dogs: a review. Int J Curr Microbiol App Sci. (2018) 7:1760–73. doi: 10.20546/ijcmas.2018.707.209

[189] Froldi F Ferronato G Prandini A. Animal welfare in swine production In: Fellegara AM Torelli R Caccialanza A, editors. Sustainable transition of meat and cured meat supply chain: A transdisciplinary approach. Cham: Springer Nature Switzerland (2023). 85–102.

[190] Wang C Yang C Zeng Y Zhang M. GnRH-immunocastration: an alternative method for male animal surgical castration. Front Vet Sci. (2023) 10:1248879. doi: 10.3389/fvets.2023.1248879, PMID: 38026623 PMC10644813

[191] Linden J. Literature Review on the Welfare Implications of Swine Castration. AVMA Policies: Swine castration. (2013) 1–5. Available at: https://www.avma.org/resources-tools/avma-policies/swine-castration

[192] Borell EV Bonneau M Holinger M Prunier A Stefanski V Zöls S. Welfare aspects of raising entire male pigs and Immunocastrates. Animals (Basel). (2020) 10:2140. doi: 10.3390/ani10112140 33213105 PMC7698590

[193] Fàbrega E. Alternatives to castration of pigs In: Edwards S, editor. Understanding the behaviour and improving the welfare of pigs. London: Burleigh Dodds Science Publishing (2020)

[194] Weiler U Font-I-Furnols M Tomasevic I Bonneau M. Alternatives to piglet castration: from issues to solutions. Animals. (2021) 11:1041. doi: 10.3390/ani11041041, PMID: 33917235 PMC8067991

[195] Ahmed S Jiang X Liu G Sadiq A Farooq U Wassie T. New trends in immunocastration and its potential to improve animal welfare: a mini review. Trop Anim Health Prod. (2022) 54:369. doi: 10.1007/s11250-022-03348-8, PMID: 36323906

[196] Kim J So B Heo Y So H Jo JK. Advances in male contraception: when will the novel male contraception be available? World J Mens Health. (2024) 42:118. doi: 10.5534/wjmh.230118 PMC11216971 38164023

[197] Service CA Puri D Hsieh T-C Patel DP. Emerging concepts in male contraception: a narrative review of novel, hormonal and non-hormonal options. Therap Adv Reproduct Health. (2023) 17:1–24. doi: 10.1177/26334941221138323 PMC9996746 36909934

[198] Yang F Li J Dong L Tan K Huang X Zhang P. Review of vasectomy complications and safety concerns. World J Men's Health. (2021) 39:406–18. doi: 10.5534/wjmh.200073, PMID: 32777870 PMC8255399

[199] Leavesley JH. Brief history of vasectomy. Fam Plann Inf Serv. (1980) 1:2–3. PMID: 12336890

[200] Harrison R. On some structural varieties of the enlarged prostate relative to its treatment. Lancet. (1899) 154:331–2. doi: 10.1016/S0140-6736(01)40338-2

[201] Sarrat R Whyte J Torres A Lostalé F Díaz MP. Experimental vasectomy and testicular structure. Histol Histopathol. (1996) 11:1–6. PMID: 8720442

[202] Libermann S Etchepareborde S Gautherot A Etienne-Raffestin CL Coste M Moreau S. Laparoscopic castration in dogs: complications, outcomes and long-term follow-up. Revue Vétérinaire Clinique. (2020) 55:1–10. doi: 10.1016/j.anicom.2020.02.001

[203] Madbouly H Korany R El-Shahat K Eissa H Fathi M. Efficacy of Intratesticular glycerol injection as male cat contraception in comparison with two surgical approaches. Top Companion Anim Med. (2021) 42:100493. doi: 10.1016/j.tcam.2020.100493, PMID: 33152526

[204] Mahalingam A Kumar N Maiti SK Sharma A Dimri U Kataria M. Laparoscopic vasectomy vs laparoscopic sterilization in dogs: a comparison of two techniques. World J Laparoscopic Surg DVD. (2014) 7:7–15. doi: 10.5005/jp-journals-10007-1210

[205] Seppan P Krishnaswamy K. Long-term study of vasectomy in *Macaca radiata* – histological and ultrasonographic analysis of testis and duct system. Syst Biol Reprod Med. (2014) 60:151–60. doi: 10.3109/19396368.2014.896957, PMID: 24593799

[206] McDonald SW. Cellular responses to vasectomy. Int Rev Cytol. (2000) 199:295–339. doi: 10.1016/S0074-7696(00)99006-5 10874581

[207] Flickinger CJ Herr JC Howards SS Caloras D Yarbro ES Spell DR. The influence of vasovasostomy on testicular alterations after vasectomy in Lewis rats. Anat Rec. (1987) 217:137–45. doi: 10.1002/ar.1092170205 3495205

[208] Alexander NJ. Vasectomy: long-term effects in the rhesus monkey. J Reprod Fert. (1972) 31:399–406. doi: 10.1530/jrf.0.0310399 4630651

[209] Ansbacher R. Sperm-agglutinating and sperm-immobilizing antibodies in vasectomized men. Fertil Steril. (1971) 22:629–32. doi: 10.1016/S0015-0282(16)38520-X, PMID: 5115099

[210] Rival C Wheeler K Jeffrey S Qiao H Luu B Tewalt EF. Regulatory T cells and vasectomy. J Reprod Immunol. (2013) 100:66–75. doi: 10.1016/j.jri.2013.08.004, PMID: 24080233 PMC3965253

[211] Urry RL Dougherty KA Cockett AT. Vasectomy and Vasovasostomy. I. Timing of histologic changes in immature and mature dog testis after vasectomy. Fertil Steril. (1976) 27:937–44. doi: 10.1016/S0015-0282(16)42022-4, PMID: 955136

[212] Parizek J Zahor Z. Effect of cadmium salts on testicular tissue. Nature. (1956) 177:1036–7. doi: 10.1038/1771036b0, PMID: 13322022

[213] Rios C Méndez-Armenta M. Cadmium Neurotoxicity In: Nriagu J, editor. Encyclopedia of environmental health. Oxford: Elsevier (2019). 485–91.

[214] Saini MS Parshad VR. Field evaluation of alpha-chlorohydrin against the Indian mole rat: studies on toxic and antifertility effects. Ann Appl Biol. (1993) 122:153–60. doi: 10.1111/j.1744-7348.1993.tb04023.x

[215] Jenkins JR. Surgical sterilization in small mammals. Spay and castration. Vet Clin North Am Exot Anim Pract. (2000) 3:617–28. doi: 10.1016/S1094-9194(17)30065-8, PMID: 11228921

[216] Roosa KA Mukai M Place NJ. 4-Vinylcyclohexene diepoxide reduces fertility in female Siberian hamsters when treated during their reproductively active and quiescent states. Reprod Toxicol. (2015) 51:40–6. doi: 10.1016/j.reprotox.2014.12.003, PMID: 25511107

[217] Witmer GW Raymond-Whish S. Reduced fecundity in free-ranging Norway rats after baiting with a liquid fertility control bait. Human-Wildlife Interact. (2021) 15:111–23. doi: 10.26077/10a0-13c9

[218] Kortenkamp A. Which chemicals should be grouped together for mixture risk assessments of male reproductive disorders? Mol Cell Endocrinol. (2020) 499:110581. doi: 10.1016/j.mce.2019.110581, PMID: 31525431

[219] Yan M Mao B Li L Li SYT Wong CKC Silvestrini B. Testis toxicants In: Skinner MK, editor. Encyclopedia of reproduction. Oxford: Academic Press (2018). 559–66.

[220] Boekelheide K. Mechanisms of toxic damage to spermatogenesis. J Natl Cancer Inst Monogr. (2005) 2005:6–8. doi: 10.1093/jncimonographs/lgi006 15784812

[221] Nolte T Harleman JH Jahn W. Histopathology of chemically induced testicular atrophy in rats. Exp Toxicol Pathol. (1995) 47:267–86. doi: 10.1016/S0940-2993(11)80260-5 8855122

[222] Balaguer P Bourguet W. Toxicant actions: mode of action of endocrine disruptors In: Skinner MK, editor. Encyclopedia of reproduction. Oxford: Academic Press (2018). 567–72.

[223] Kutzler M Wood A. Non-surgical methods of contraception and sterilization. Theriogenology. (2006) 66:514–25. doi: 10.1016/j.theriogenology.2006.04.014 16757019

[224] Humphrys S Lapidge S. Delivering and registering species-tailored oral antifertility products: a review. Wildl Res. (2008) 35:578–85. doi: 10.1071/WR07145

[225] Heeres J Backx LJ Mostmans JH Van Cutsem J. Antimycotic imidazoles. Part 4. Synthesis and antifungal activity of ketoconazole, a new potent orally active broad-spectrum antifungal agent. J Med Chem. (1979) 22:1003–5. doi: 10.1021/jm00194a023, PMID: 490531

[226] Dismukes WE Stamm AM Graybill JR Craven PC Stevens DA Stiller RL. Treatment of systemic mycoses with ketoconazole: emphasis on toxicity and clinical response in 52 patients. National Institute of Allergy and Infectious Diseases collaborative antifungal study. Ann Intern Med. (1983) 98:13–20. doi: 10.7326/0003-4819-98-1-13, PMID: 6293361

[227] Katiraee F Kouchak Kosari Y Soltani M Shokri H Hassan Minooieanhaghighi M. Molecular identification and antifungal susceptibility patterns of dermatophytes isolated from companion Animals with clinical symptoms of Dermatophytosis. J Vet Res. (2021) 65:175–82. doi: 10.2478/jvetres-2021-0020, PMID: 34250302 PMC8256462

[228] Willard MD Nachreiner RF Howard VC Fooshee SK. Effect of long-term administration of ketoconazole in cats. Am J Vet Res. (1986) 47:2510–3. PMID: 3800111

[229] Borgers M Van den Bossche H De Brabander M. The mechanism of action of the new antimycotic ketoconazole. Am J Med. (1983) 74:2–8. doi: 10.1016/0002-9343(83)90507-7 6295147

[230] Tritos NA. Adrenally directed medical therapies for Cushing syndrome. J Clin Endocrinol Metab. (2021) 106:16–25. doi: 10.1210/clinem/dgaa778, PMID: 33118025

[231] Schürmeyer T Nieschlag E. Effect of ketoconazole and other imidazole fungicides on testosterone biosynthesis. Acta Endocrinol. (1984) 105:275–80. doi: 10.1530/acta.0.1050275, PMID: 6320571

[232] Pont A Williams PL Azhar S Reitz RE Bochra C Smith ER. Ketoconazole blocks testosterone synthesis. Arch Intern Med. (1982) 142:2137–40. doi: 10.1001/archinte.1982.00340250097015 6291475

[233] Waller DP Martin A Vickery BH Zaneveld LJD. The effect of ketoconazole on fertility of male rats. Contraception. (1990) 41:411–7. doi: 10.1016/0010-7824(90)90040-3, PMID: 2335105

[234] Sonino N. The use of ketoconazole as an inhibitor of steroid production. N Engl J Med. (1987) 317:812–8. doi: 10.1056/NEJM198709243171307 3306384

[235] Vickery BH Burns J Zaneveld LJ Goodpasture JC Bergstrom K. Orally administered ketoconazole rapidly appears in seminal plasma and suppresses sperm motility. Adv Contracept. (1985) 1:341–53. doi: 10.1007/BF01849310 3842225

[236] Zaneveld LJ Waller DP. Nonhormonal mediation of male reproductive tract damage: data from contraceptive drug research. Prog Clin Biol Res. (1989) 302:129–49. discussion 150–6 2666986

[237] Joshi SC Jain GC Lata M. Effects of ketoconazole (an imidazole antifugal agent) on the fertility and reproductive function of male mice. Acta Eur Fertil. (1994) 25:55–8. PMID: 7887081

[238] Adams ML Meyer ER Cicero TJ. Imidazoles suppress rat testosterone secretion and testicular interstitial fluid formation *in vivo*. Biol Reprod. (1998) 59:248–54. doi: 10.1095/biolreprod59.2.248, PMID: 9687292

[239] Amin A. Ketoconazole-induced testicular damage in rats reduced by Gentiana extract. Exp Toxicol Pathol. (2008) 59:377–84. doi: 10.1016/j.etp.2007.10.008, PMID: 18222659

[240] Janssen P., Ketoconazole. (2021).

[241] Tsabai C. Potential drug interactions in patients taking Oral contraceptive pills. Am Fam Physician. (2019) 100:599–600. PMID: 31730323

[242] Chhabra RS Elwell MR Peters A. Toxicity of 4-vinyl-1-cyclohexene diepoxide after 13 weeks of dermal or oral exposure in rats and mice. Fundam Appl Toxicol. (1990) 14:745–51. doi: 10.1016/0272-0590(90)90299-Y 2361574

[243] Dhillon S Von Burg R. Vinylcyclohexene dioxide. J Appl Toxicol. (1996) 16:465–8. 8889800 10.1002/jat.2550160503

[244] Hooser SB DeMerell DG Douds DA Hoyer P Sipes IG. Testicular germ cell toxicity caused by vinylcyclohexene diepoxide in mice. Reprod Toxicol. (1995) 9:359–67. doi: 10.1016/0890-6238(95)00022-3, PMID: 7496092

[245] Kappeler CJ Hoyer PB. 4-vinylcyclohexene diepoxide: a model chemical for ovotoxicity. Syst Biol Reprod Med. (2012) 58:57–62. doi: 10.3109/19396368.2011.648820, PMID: 22239082 PMC3307534

[246] Max A Jurka P Dobrzyn A Rijsselaere T. Non-surgical contraception in female dogs and cats. Acta Sci Pol Zootechnica. (2014) 13:3–18.

[247] Paksoy Z Kandemir F Gökhan N Ozkaraca M. The effects of 4-vinylcyclohexene diepoxide on the testes of dogs. Veterinarski Arhiv. (2018) 88:807–22. doi: 10.24099/vet.arhiv.0101

[248] Cathey M Memon MA. Nonsurgical methods of contraception in dogs and cats: where are we now? Vet Med. (2010) dvm360:12–7.

[249] Abolaji AO Omozokpia MU Oluwamuyide OJ Akintola TE Farombi EO. Rescue role of hesperidin in 4-vinylcyclohexene diepoxide-induced toxicity in the brain, ovary and uterus of wistar rats. J Basic Clin Physiol Pharmacol. (2020) 31:20180115. doi: 10.1515/jbcpp-2018-0115, PMID: 32160159

[250] Huynh PN Hikim AP Wang C Stefonovic K Lue YH Leung A. Long-term effects of triptolide on spermatogenesis, epididymal sperm function, and fertility in male rats. J Androl. (2000) 21:689–99. doi: 10.1002/j.1939-4640.2000.tb02137.x, PMID: 10975416

[251] Elmore H. The Rodent Birth Control Landscape (2022). 30 p.

[252] Jacoblinnert K Jacob J Zhang Z Hinds LA. The status of fertility control for rodents—recent achievements and future directions. Integrative Zool. (2022) 17:964–80. doi: 10.1111/1749-4877.12588, PMID: 34549512

[253] Massei G. Fertility Control for wildlife: a European perspective. Animals. (2023) 13:428. doi: 10.3390/ani13030428, PMID: 36766317 PMC9913817

[254] Asa C Griffin S Eckery D Hinds L Massei G. Foreword to the special issue on ‘fertility control for wildlife in the 21st century’. Wildl Res. (2024) 51:1–5. doi: 10.1071/WR23142

[255] Counsell RE Lu MC El-Masry S Weinhold PA. Inhibition of cholesterol side-chain cleavage by azacholesterols. Biochem Pharmacol. (1971) 20:2912–5. doi: 10.1016/0006-2952(71)90205-X, PMID: 5165491

[256] Ranney RE Cook DL. The hypocholesterolemic action of 20,25-diazacholesterol. Arch Int Pharmacodyn Ther. (1965) 154:51–62. PMID: 14346416

[257] Sinha Hikim AP Chakraborty J. Effects of diazacholesterol dihydrochloride (SC-12937), an avian antifertility agent, on rat testis. J Androl. (1986) 7:277–84. doi: 10.1002/j.1939-4640.1986.tb00930.x, PMID: 3771367

[258] Singh SK Chakravarty S. Antispermatogenic and antifertility effects of 20,25-diazacholesterol dihydrochloride in mice. Reprod Toxicol. (2003) 17:37–44. doi: 10.1016/S0890-6238(02)00075-8, PMID: 12507656

[259] Lofts B Murton RK Thearle RJ. The effects of 22,25-diazacholesterol dihydrochloride on the pigeon testis and on reproductive behaviour. J Reprod Fertil. (1968) 15:145–8. doi: 10.1530/jrf.0.0150145, PMID: 5643478

[260] Yoder C.A. Bynum K. Miller L. Development of diazacon™ as an avian contraceptive. In 11th wildlife damage management conference. (2005). Traverse City, MI.

[261] Yoder C Andelt W Miller LA Johnston J Goodall M. Effectiveness of twenty, twenty-five Diazacholesterol, avian gonadotropin-releasing hormone, and chicken riboflavin carrier protein for inhibiting reproduction in Coturnix quail. Poult Sci. (2004) 83:234–44. doi: 10.1093/ps/83.2.234, PMID: 14979575

[262] Avery ML Yoder CA Tillman EA. Diazacon inhibits reproduction in invasive monk parakeet populations. J Wildl Manag. (2008) 72:1449–52. doi: 10.2193/2007-391

[263] Nash P Furcolow CA Bynum KS Yoder CA Miller LA Johnston JJ. 20,25-Diazacholesterol as an oral contraceptive for black-tailed prairie dog population management. Human–Wildlife Conflicts. (2007) 1:60–7.

[264] Yoder CA Mauldin RE Gionfriddo JP Crane KA Goldade DA Engeman RM. DiazaCon reduces black-tailed prairie dog reproduction in Colorado. Wildl Res. (2016) 43:655–61. doi: 10.1071/WR15210

[265] Mayle B Feryman M Peace A Yoder CA Miler LA Cowan D. The use of DiazaCon(™) to limit fertility in grey squirels In: 8th European vertebrate Pest management conference. Berlin, Germany: Julius-Kühn-Archiv (2011). 120–1. doi: 10.5073/jka.2011.432.065

[266] Yoder CA Mayle BA Furcolow CA Cowan DP Fagerstone KA. Feeding of grey squirrels (*Sciurus carolinensis*) with the contraceptive agent DiazaCon™: effect on cholesterol, hematology, and blood chemistry. Integr Zool. (2011) 6:409–19. doi: 10.1111/j.1749-4877.2011.00247.x, PMID: 22182332

[267] Mayle BA Ferryman M Peace A Yoder CA Miller L Cowan D. The use of DiazaCon™ to limit fertility by reducing serum cholesterol in female grey squirrels, *Sciurus carolinensis*. Pest Manag Sci. (2013) 69:414–24. doi: 10.1002/ps.3347, PMID: 22791583

[268] Davidse LC Flach W. Differential binding of methyl benzimidazol-2-yl carbamate to fungal tubulin as amechanism of resistance to this antimitotic agent in mutant strains of aspergillus nidulans. J Cell Biol. (1977) 72:174–93. doi: 10.1083/jcb.72.1.174, PMID: 12184 PMC2110979

[269] Carter SD Laskey JW. Effect of benomyl on reproduction in the male rat. Toxicol Lett. (1982) 11:87–94. doi: 10.1016/0378-4274(82)90111-4 7090021

[270] Barnes T Verlangieri A Wilson M. Reproductive toxicity of methyl-1-butylcarbamoyl-2-benzimidazole carbamate benomyl in male Wistar rats. Toxicology. (1983) 28:103–15. doi: 10.1016/0300-483X(83)90110-5, PMID: 6636195

[271] Carter SD Hein JF Rehnberg GL Laskey JW. Effect of benomyl on the reproductive development of male rats. J Toxicol Environ Health. (1984) 13:53–68. doi: 10.1080/15287398409530481, PMID: 6425509

[272] Carter SD Hess RA Laskey JW. The fungicide methyl 2-benzimidazole carbamate causes infertility in male Sprague-Dawley rats. Biol Reprod. (1987) 37:709–17. doi: 10.1095/biolreprod37.3.709, PMID: 3676414

[273] Lim J Miller MG. The role of the benomyl metabolite carbendazim in benomyl-induced testicular toxicity. Toxicol Appl Pharmacol. (1997) 142:401–10. doi: 10.1006/taap.1996.8042, PMID: 9070363

[274] Nakai M Hess RA. Effects of carbendazim (methyl 2-benzimidazole carbamate; MBC) on meiotic spermatocytes and subsequent spermiogenesis in the rat testis. Anat Rec. (1997) 247:379–87. doi: 10.1002/(SICI)1097-0185(199703)247:3<379::AID-AR9>3.0.CO;2-P 9066915

[275] Nakai M Hess RA Netsu J Nasu T. Deformation of the rat Sertoli cell by oral administration of carbendazim (methyl 2-benzimidazole carbamate). J Androl. (1995) 16:410–6. doi: 10.1002/j.1939-4640.1995.tb00554.x, PMID: 8575980

[276] Nakai M Hess RA. Morphological changes in the rat Sertoli cell induced by the microtubule poison carbendazim. Tissue Cell. (1994) 26:917–27. doi: 10.1016/0040-8166(94)90041-8 7886678

[277] Nakai M Hess RA Moore BJ Guttroff RF Strader LF Linder RE. Acute and long-term effects of a single dose of the fungicide carbendazim (methyl 2-benzimidazole carbamate) on the male reproductive system in the rat. J Androl. (1992) 13:507–18. doi: 10.1002/j.1939-4640.1992.tb00345.x 1293130

[278] Hess RA Moore BJ Forrer J Linder RE Abuel-Atta AA. The fungicide benomyl (methyl 1-(butylcarbamoyl)-2- benzimidazolecarbamate) causes testicular dysfunction by inducing the sloughing of germ cells and occlusion of efferent ductules. Fund Appl Toxicol. (1991) 17:733–45. doi: 10.1016/0272-0590(91)90181-3, PMID: 1778360

[279] Nakai M Moore BJ Hess RA. Epithelial reorganization and irregular growth following carbendazim-induced injury of the efferent ductules of the rat testis. Anat Rec. (1993) 235:51–60. doi: 10.1002/ar.1092350106, PMID: 8417628

[280] Hess RA. Effects of environmental toxicants on the efferent ducts, epididymis and fertility. J Reprod Fertil Suppl. (1998) 53:247–59. PMID: 10645284

[281] Hoque M Chen D Hess RA Li FQ Takemaru KI. CEP164 is essential for efferent duct multiciliogenesis and male fertility. Reproduction. (2021) 162:129–39. doi: 10.1530/REP-21-0042, PMID: 34085951 PMC8269963

[282] Kucera SP Swann JM Kennedy JR Schultz TW. The effects of benomyl and its breakdown products carbendazim and butyl isocyanate on the structure and function of tracheal ciliated cells. J Environ Sci Health B. (1995) 30:779–99. doi: 10.1080/03601239509372965, PMID: 7594216

[283] Hess RA Nakai M. Histopathology of the male reproductive system induced by the fungicide benomyl. Histol Histopathol. (2000) 15:207–24. doi: 10.14670/HH-15.207, PMID: 10668211

[284] Carter SK. Current protocol approaches in large bowel cancer. Semin Oncol. (1976) 3:433–43. 793018

[285] Cioli V Bellocci B Putzolu S Malorni W Demartino C. Anti-spermogenic activity of lonidamine (AF-1890) in rabbit. Ultramicroscopy. (1980) 5:418–23.

[286] Xie QR Liu Y Shao J Yang J Liu T Zhang T. Male contraceptive Adjudin is a potential anti-cancer drug. Biochem Pharmacol. (2013) 85:345–55. doi: 10.1016/j.bcp.2012.11.008 23178657 PMC4108200

[287] Nath K Guo L Nancolas B Nelson DS Shestov AA Lee S-C. Mechanism of antineoplastic activity of lonidamine. Biochim Biophys Acta. (2016) 1866:151–62. doi: 10.1016/j.bbcan.2016.08.001, PMID: 27497601 PMC5138080

[288] Heywood R James RW Barcellona PS Campana A Cioli V. Toxicological studies on 1-substituted-indazole-3-carboxylic acids. Chemotherapy. (1981) 27:91–7. doi: 10.1159/000238049 7285641

[289] James RW. A toxicological study of some chemical actions on the testes of laboratory rats and beagle dogs [dissertation] In: Department of Pharmacy & pharmacology. Bath, UK: University of Bath (1980).

[290] Cheng CY Mo M Grima J Saso L Tita B Mruk D. Indazole carboxylic acids in male contraception. Contraception. (2002) 65:265–8. doi: 10.1016/S0010-7824(01)00318-3, PMID: 12020774

[291] Maranghi F Mantovani A Macri C Romeo A Eleuteri P Leter G. Long-term effects of lonidamine on mouse testes. Contraception. (2005) 72:268–72. doi: 10.1016/j.contraception.2005.05.019, PMID: 16181970

[292] Traina ME Guarino M Urbani E Saso L Eleuteri P Cordelli E. Lonidamine transiently affects spermatogenesis in pubertal CD1 mice. Contraception. (2005) 72:262–7. doi: 10.1016/j.contraception.2005.04.008, PMID: 16181969

[293] Mruk DD Silvestrini B Yan Cheng C. Anchoring junctions as drug targets: role in contraceptive development. Pharmacol Rev. (2008) 60:146–80. doi: 10.1124/pr.107.07105, PMID: 18483144 PMC3023124

[294] Tash JS Attardi B Hild SA Chakrasali R Jakkaraj SR Georg GI. A novel potent indazole carboxylic acid derivative blocks spermatogenesis and is contraceptive in rats after a single oral dose. Biol Reprod. (2008) 78:1127–38. doi: 10.1095/biolreprod.106.057810, PMID: 18218612

[295] De Martino C Malcorni W Bellocci M Floridi A Marcante ML. Effects of AF 1312 TS and lonidamine on mammalian testis. A morphological study. Chemotherapy. (1981) 27:27–42. doi: 10.1159/000238043 7285636

[296] Patton DS Heywood R Barcellona PS. Carcinogenicity assessment of lonidamine by dietary administration to Sprague-Dawley rats. Toxicol Lett. (1992) 62:209–14. doi: 10.1016/0378-4274(92)90023-D, PMID: 1412505

[297] Mruk DD Bonanomi M Silvestrini B. Lonidamine-ethyl ester-mediated remodelling of the Sertoli cell cytoskeleton induces phosphorylation of plakoglobin and promotes its interaction with alpha-catenin at the blood-testis barrier. Reprod Fertil Dev. (2017) 29:998–1011. doi: 10.1071/RD15378 28442050

[298] Georg I.G. Tash J.S. Chakrasali R. Jakkaraj S.R. Roby K., Lonidamine Analogues for Fertility Management, States. (2013). University of Kansas, Lawrence, KS: p. 1–44.

[299] Su L Cheng CY Mruk DD. Adjudin-mediated Sertoli-germ cell junction disassembly affects, Sertoli cell barrier function in vitro and in vivo. Int J Biochem Cell Biol. (2010) 42:1864–75. doi: 10.1016/j.biocel.2010.08.004, PMID: 20713173 PMC2950250

[300] Cheung KH Leung CT Leung GP Wong PY. Synergistic effects of cystic fibrosis transmembrane conductance regulator and aquaporin-9 in the rat epididymis. Biol Reprod. (2003) 68:1505–10. doi: 10.1095/biolreprod.102.010017, PMID: 12606488

[301] Gong XD Linsdell P Cheung KH Leung GP Wong PY. Indazole inhibition of cystic fibrosis transmembrane conductance regulator cl(−) channels in rat epididymal epithelial cells. Biol Reprod. (2002) 67:1888–96. doi: 10.1095/biolreprod.102.007450 12444067

[302] Malorni W Meschini S Matarrese P Arancia G. The cytoskeleton as a subcellular target of the antineoplastic drug lonidamine. Anticancer Res. (1992) 12:2037–45. PMID: 1295447

[303] Leung PH. Transport mechanism underlying the formation of microenvironment in rat efferent duct and epididymis [dissertation]. Hong Kong: The Chinese University of Hong Kong (Hong Kong) (2001). 194 p.

[304] Wang YY Lin YH Wu YN Chen YL Lin YC Cheng CY. Loss of SLC9A3 decrease CFTR protein and causes obstructed azoospermia in mice. PLoS Genet. (2017) 13:e1006715. doi: 10.1371/journal.pgen.1006715, PMID: 28384194 PMC5398719

[305] Hihnala S Kujala M Toppari J Kere J Holmberg C Hoglund P. Expression of SLC26A3, CFTR and NHE3 in the human male reproductive tract: role in male subfertility caused by congenital chloride diarrhoea. Mol Hum Reprod. (2006) 12:107–11. doi: 10.1093/molehr/gal009 16421216

[306] Kujala M Hihnala S Tienari J Kaunisto K Hastbacka J Holmberg C. Expression of ion transport-associated proteins in human efferent and epididymal ducts. Reproduction. (2007) 133:775–84. doi: 10.1530/rep.1.00964, PMID: 17504921

[307] El Khouri E Whitfield M Stouvenel L Kini A Riederer B Lores P. Slc26a3 deficiency is associated with epididymis dysplasia and impaired sperm fertilization potential in the mouse. Mol Reprod Dev. (2018) 85:682–95. doi: 10.1002/mrd.23055, PMID: 30118583

[308] PubChem. 3-Chloro-1,2-propanediol. Bethesda, MD: National Library of Medicine (US) (2021).

[309] Ericsson R.J. Epibloc. (2021).

[310] Brown-Woodman PDC White IG. Effect of α-chlorohydrin on cauda epididymis and spermatozoa of the rat and general physiological status. Contraception. (1975) 11:69–78. doi: 10.1016/0010-7824(75)90052-9 1116362

[311] Jones AR. Antifertility actions of alpha-chlorohydrin in the male. Aust J Biol Sci. (1983) 36:333–50. doi: 10.1071/BI9830333, PMID: 6362633

[312] Haratake J Furuta A Hashimoto H. Immunohistochemical and ultrastructural study of hepatic sinusoidal linings during dichloropropanol-induced acute hepatic necrosis. Liver. (1994) 14:90–7. doi: 10.1111/j.1600-0676.1994.tb00054.x, PMID: 8196516

[313] Ericsson RJ Youngdale GA. Male antifertility compounds: structure and activity relationships of U-5897, U-l5,646 and related substances. J Reprod Fertil. (1970) 21:263–6. doi: 10.1530/jrf.0.0210263 5443209

[314] Ericsson RJ Baker VF. Male antifertility compounds: biological properties of U-5897 and U-15646. J Reprod Fert. (1970) 21:267–73. doi: 10.1530/jrf.0.0210267 5443210

[315] Back DJ Glover TD Shenton JC Boyd GP. The effects of a-chlorohydrin on the composition of rat and rabbit epididymal plasma: a possible explanation of species difference. J Reprod Fert. (1975) 45:117–28. doi: 10.1530/jrf.0.0450117, PMID: 1195243

[316] Crabo BG Zimmerman KJ Gustafsson B Holtman M Koh TJP Graham EF. Effect of low doses of a-chlorohydrin on fertility and semen characteristics and binding of the drug to spermatozoa in swine. Int J Fertil. (1975) 20:87–94. PMID: 3483

[317] Mohri HD Sutter DAI Brown-Woodman PDC White G Ridley D. Identification of the biochemical lesion produced by a-chlorohydrin in spermatozoa. Nature. (1975) 255:75–7. doi: 10.1038/255075a0, PMID: 1128672

[318] Brown-Woodman PDC White JG. Disruption of the metabolism motility and morphology of spermatozoa by injection of a-chlorohydrin into rams. Aust J Biol Sci. (1976) 29:545–55. doi: 10.1071/BI9760545 1023867

[319] Paz GF Homonnai TZ. A direct effect of alpha-chlorohydrin on rat epididymal spermatozoa. Int J Androl. (1982) 5:308–16. doi: 10.1111/j.1365-2605.1982.tb00259.x, PMID: 7118269

[320] Jones AR Porter LM. Inhibition of glycolysis in boar spermatozoa by alpha-chlorohydrin phosphate appears to be mediated by phosphatase activity. Reprod Fertil Dev. (1995) 7:1089–94. doi: 10.1071/RD9951089, PMID: 8848575

[321] Jelks KB Miller MG. Alpha-chlorohydrin inhibits glyceraldehyde-3-phosphate dehydrogenase in multiple organs as well as in sperm. Toxicol Sci. (2001) 62:115–23. doi: 10.1093/toxsci/62.1.115 11399799

[322] Yamada T Inoue T Sato A Yamagishi K Sato M. Effects of short-term administration of alpha-chlorohydrin on reproductive toxicity parameters in male Sprague-Dawley rats. J Toxicol Sci. (1995) 20:195–205. doi: 10.2131/jts.20.195 8667446

[323] Wong PY Yeung CH. Inhibition by alpha-chlorohydrin of fluid reabsorption in the rat cauda epididymidis. J Reprod Fertil. (1977) 51:469–71. doi: 10.1530/jrf.0.0510469, PMID: 592300

[324] Wong PYD. Effect of alpha-chlorohydrin on transport processes in perfused rat cauda epididymides. Contraception. (1977) 16:637–44. doi: 10.1016/0010-7824(77)90064-6

[325] Hinton BT Hernandez H Howards SS. The male antifertility agents alpha chlorohydrin, 5-thio-D-glucose, and 6-chloro-6-deoxy-D-glucose interfere with sugar transport across the epithelium of the rat caput epididymidis. J Androl. (1983) 4:216–21. doi: 10.1002/j.1939-4640.1983.tb00758.x 6874562

[326] Samojlik E Chang MC. Antifertility activity of 3-chloro-1, 2-propanediol (U-5897) on male rats. Biol Reprod. (1970) 2:299–304. doi: 10.1095/biolreprod2.2.299 5520328

[327] Cooper ER Jones AR Jackson H. Effects of alpha-chlorohydrin and related compounds on the reproductive organs and fertility of the male rat. J Reprod Fertil. (1974) 38:379–86. doi: 10.1530/jrf.0.0380379, PMID: 4833816

[328] Reijonen K Kormano M Ericsson RJ. Studies on the rat epididymal blood vessels following alpha-chlorohydrin administration. Biol Reprod. (1975) 12:483–90. doi: 10.1095/biolreprod12.4.483, PMID: 56954

[329] Ericsson R.J. Alpha-chlorohydrin (EPIBLOC): a toxicant-sterilant as an alternative in rodent control. 10th Vertebrate Pest Conference p. 6–9. (1982).

[330] Kar AB Das RP. Sterilization of males by intratesticular administration of cadmium chloride. Acta Endocrinol. (1962) 40:321–31. doi: 10.1530/acta.0.0400321 14453824

[331] Russell LD Saxena NK Weber JE. Intratesticular injection as a method to assess the potential toxicity of various agents and to study mechanisms of normal spermatogenesis. Gamete Res. (1987) 17:43–56. doi: 10.1002/mrd.1120170106, PMID: 2906900

[332] Fahim M.S. Neutersol® injectable solution for Dogs (Zinc Gluconate Neutralized by Arginine). 2003, FDA. Available at: https://animaldrugsatfda.fda.gov/adafda/app/search/public/document/downloadFoi/748

[333] Migally NB Fahim MS. Pharmacokinetics of zinc tannate after intratesticular injection. Arch Androl. (1984) 13:129–36. doi: 10.3109/01485018408987511, PMID: 6537741

[334] Tepsumethanon V Wilde H Hemachudha T. Intratesticular injection of a balanced zinc solution for permanent sterilization of dogs. J Med Assoc Thail. (2005) 88:686–9. PMID: 16149690

[335] Fahim MS. Chemical castration. USA patent US 5372822 (1994). 14 p.

[336] Moldave K Briggs JR. Contraception and fertility Control in dogs and cats, dogs. A report for the Alliance for Contraception in Cats & Dogs. (2013). 1–154.

[337] Vanderstichel R Forzan M Pérez GE Serpell J Garde E. Changes in blood testosterone concentrations after surgical and chemical sterilization of male free-roaming dogs in southern Chile. Theriogenology. (2015) 83:1021–7. doi: 10.1016/j.theriogenology.2014.12.001 25557187

[338] Forzan MJ Garde E Perez GE Vanderstichel RV. Necrosuppurative orchitis and scrotal necrotizing dermatitis following intratesticular administration of zinc gluconate neutralized with arginine (EsterilSol) in 2 mixed-breed dogs. Vet Pathol. (2014) 51:820–3. doi: 10.1177/0300985813505875, PMID: 24078007

[339] Benka VAW. Nonsurgical Fertility Control In: Polak JM Kommedal AT, editors. Field manual for small animal medicine. Hoboken, NJ: John Wiley & Sons (2018). 179–200.

[340] Araujo-Lima CF Nunes RJ Carpes RM Aiub CA Felzenszwalb I. Pharmacokinetic and toxicological evaluation of a zinc gluconate-based chemical Sterilant using in vitro and in Silico approaches. Biomed Res Int. (2017) 2017:5746768. doi: 10.1155/2017/5746768 28197414 PMC5288521

[341] Fagundes AK Oliveira EC Tenorio BM Melo CC Nery LT Santos FA. Injection of a chemical castration agent, zinc gluconate, into the testes of cats results in the impairment of spermatogenesis: a potentially irreversible contraceptive approach for this species? Theriogenology. (2014) 81:230–6. doi: 10.1016/j.theriogenology.2013.09.013, PMID: 24238399

[342] Oliveira EC Fagundes AK Melo CC Nery LT Revoredo RG Andrade TF. Intratesticular injection of a zinc-based solution for contraception of domestic cats: a randomized clinical trial of efficacy and safety. Vet J. (2013) 197:307–10. doi: 10.1016/j.tvjl.2013.01.011, PMID: 23465750

[343] Oliveira EC Muller PM Silva FL Nery LT de Sa MJ Guerra MM. Oral administration of an anti-inflammatory does not compromise the efficacy of intra-testicular injection of zinc gluconate as a contraceptive for dogs. Anim Reprod Sci. (2012) 132:207–12. doi: 10.1016/j.anireprosci.2012.05.003 22682769

[344] Oliveira EC Moura MR de Sa MJ Silva VA Jr Kastelic JP Douglas RH. Permanent contraception of dogs induced with intratesticular injection of a zinc gluconate-based solution. Theriogenology. (2012) 77:1056–63. doi: 10.1016/j.theriogenology.2011.10.008, PMID: 22192397

[345] Oliveira EC Moura MR Silva VA Jr Peixoto CA Saraiva KL de Sa MJ. Intratesticular injection of a zinc-based solution as a contraceptive for dogs. Theriogenology. (2007) 68:137–45. doi: 10.1016/j.theriogenology.2007.03.026, PMID: 17559925

[346] Levy JK Crawford PC Appel LD Clifford EL. Comparison of intratesticular injection of zinc gluconate versus surgical castration to sterilize male dogs. Am J Vet Res. (2008) 69:140–3. doi: 10.2460/ajvr.69.1.140, PMID: 18167099

[347] DiGangi BA Grijalva J Jaramillo EPP Dueñas I Glenn C Cruz MEC. Post-operative outcomes of surgical and chemical castration with zinc gluconate in dogs presenting to veterinary field clinics. Vet J. (2017) 229:26–30. doi: 10.1016/j.tvjl.2017.10.016, PMID: 29183570

[348] Woodward KN Keesler RI Reader JR Christe KL. Evaluation of a zinc gluconate neutralized with arginine product as a nonsurgical method for sterilization of Rhesus macaques (*Macaca mulatta*). J Am Assoc Lab Anim Sci. (2017) 56:520–6. PMID: 28903822 PMC5605175

[349] Rafatmah D Mogheiseh A Eshghi D. Chemical sterilization with intratesticular administration of zinc gluconate in adult dogs: a preliminary report. Basic Clin Androl. (2019) 29:12. doi: 10.1186/s12610-019-0092-8, PMID: 31388429 PMC6670226

[350] de Macêdo SRB de Lima LAR de Torres SM de Oliveira VVG de Morais RN Peixoto CA. Effects of intratesticular injection of zinc-based solution in rats in combination with anti-inflammatory and analgesic drugs during chemical sterilization. Vet World. (2018) 11:649–56. doi: 10.14202/vetworld.2018.649-656, PMID: 29915504 PMC5993773

[351] Soto FR Viana WG Mucciolo GC Hosomi FY Vannucchi CI Mazzei CP. Evaluation of efficacy and safety of zinc gluconate associated with dimethyl sulphoxide for sexually mature canine males chemical neutering. Reprod Domest Anim. (2009) 44:927–31. doi: 10.1111/j.1439-0531.2008.01119.x, PMID: 18992122

[352] Soto F Viana W Sousa A Pinheiro S Mucciolo G Hosomi F. Evaluation of zinc gluconate, either associated or not to dimethyl sulfoxide, as contraceptive method for male dogs. J Anim Reprod. (2007) 4:119–24.

[353] Vannucchi CI Angrimani DS Eyherabide AR Mazzei CP Lucio CF Maiorka PC. Effects of intratesticular administration of zinc gluconate and dimethyl sulfoxide on clinical, endocrinological, and reproductive parameters in dogs. Theriogenology. (2015) 84:1103–10. doi: 10.1016/j.theriogenology.2015.06.005, PMID: 26174036

[354] Brito LF Sertich PL Rives W Knobbe M Del Piero F Stull GB. Effects of intratesticular zinc gluconate treatment on testicular dimensions, echodensity, histology, sperm production, and testosterone secretion in American black bears (*Ursus americanus*). Theriogenology. (2011) 75:1444–52. doi: 10.1016/j.theriogenology.2010.10.036, PMID: 21356548

[355] Wang M., Chemical Sterilant for adult male dog population control with concomtant reduction in Human dog-bit acquired rabies, in US Patent office. (2009).

[356] Matschurat C Rode K Hollenbach J Wolf K Urhausen C Beineke A. Impaired spermatogenesis, tubular wall disruption, altered blood-testis barrier composition and intratubular lymphocytes in an infertile beagle dog – a putative case of autoimmune orchitis. Histol Histopathol. (2019) 34:525–35. doi: 10.14670/hh-18-058 30403279

[357] ACC&D Zeuterin/Esterilsol product profile and position paper. (2015).

[358] Uddin J Hossain M Shamsuddin M Hossain S. Effects of Esterilsol® on sterilization of dogs. Commonwealth Vet J. (2015) 31:9–14.

[359] FDA, Freedom of Information Summary. Neutersol® Injectable Solution for Dogs (zinc gluconate neutralized by arginine). Intratesticular injection for chemical sterilization in 3 to 10 month old male dogs. (2003). US Food and Drug Administration: Washington, DC. 141–217.

[360] Akhan O Dincer A Gököz A Sayek I Havlioglu S Abbasoglu O. Percutaneous treatment of abdominal hydatid cysts with hypertonic saline and alcohol. An experimental study in sheep. Investig Radiol. (1993) 28:121–7. doi: 10.1097/00004424-199302000-00008, PMID: 8444568

[361] Koger LM. Calcium chloride castration Modern. Vet Prac. (1978) 59:119–21. 564449

[362] Jana K Samanta PK. Evaluation of single intratesticular injection of calcium chloride for nonsurgical sterilization in adult albino rats. Contraception. (2006) 73:289–300. doi: 10.1016/j.contraception.2005.07.011, PMID: 16472573

[363] Karmakar SN Das SK. Chemosterilization induced by intratesticular injection of calcium chloride (CaCl2) – a tool for population control. Int J Pharmaceut Chem Biol Sci. (2017) 7:25–35.

[364] Hami PMM Jahandideh AV Alireza V Rafiee SM. Ultrasonographic and histopathologic study of chemical castration with calcium chloride solution in rat testis. J Crit Rev. (2020) 7:931–5.

[365] Samanta PK. Chemosterilization of stray dogs. Indian J Anim Health. (1998) 37:61–2.

[366] Jana K Samanta PK. Sterilization of male stray dogs with a single intratesticular injection of calcium chloride: a dose-dependent study. Contraception. (2007) 75:390–400. doi: 10.1016/j.contraception.2007.01.022, PMID: 17434022

[367] Leoci R Aiudi G Silvestre F Lissner EA Marino F Lacalandra GM. A dose-finding, long-term study on the use of calcium chloride in saline solution as a method of nonsurgical sterilization in dogs: evaluation of the most effective concentration with the lowest risk. Acta Vet Scand. (2014) 56:63. doi: 10.1186/s13028-014-0063-1, PMID: 25317740 PMC4196017

[368] Leoci R Aiudi G Silvestre F Lissner EA Lacalandra GM. Alcohol diluent provides the optimal formulation for calcium chloride non-surgical sterilization in dogs. Acta Vet Scand. (2014) 56:62. doi: 10.1186/s13028-014-0062-2, PMID: 25317658 PMC4195956

[369] Anonymous calcium chloride (“Calchlorin”) male animal sterilization: Ingredients and procedure. Paramus, NJ: Parsemus Foundation (2014).

[370] Abu-Ahmed H. Chemical sterilization of dogs using single bilateral intra-testicular injection of calcium chloride or clove oil. Alexandria J Vet Sci. (2015) 45:26. doi: 10.5455/ajvs.179468

[371] Puri B Shah MK Thakur B Regmi B Dhakal I. Intratesticular injection of calcium chloride is a useful alternative for neutering the male dog. Int J Appl Sci Biotechnol. (2018) 6:158–63. doi: 10.3126/ijasbt.v6i2.20431

[372] Silva RCA Paranzini CS Franco LG Miguel MP Honsho CS Souza FF. Calcium chloride combined with dimethyl sulphoxide for the chemical sterilization of dogs. Reprod Domest Anim. (2018) 53:1330–8. doi: 10.1111/rda.13252, PMID: 30133007

[373] Leoci R Aiudi G Cicirelli V Brent L Iaria C Lacalandra GM. Effects of intratesticular vs intraepididymal calcium chloride sterilant on testicular morphology and fertility in dogs. Theriogenology. (2019) 127:153–60. doi: 10.1016/j.theriogenology.2019.01.006, PMID: 30708272

[374] Thakre T Shukla SN Mishra A Gupta N Kumar P. Sterilization of male dogs by intra-testicular Administration of Calcium Chloride Solution. Indian J Anim Res. (2023) 57:1091–5. doi: 10.18805/IJAR.B-4356

[375] Jana K Samanta PK. Clinical evaluation of non-surgical sterilization of male cats with single intra-testicular injection of calcium chloride. BMC Vet Res. (2011) 7:39–9. doi: 10.1186/1746-6148-7-39, PMID: 21774835 PMC3152893

[376] Ijaz M Aleem RA Manzoor A Qureshi AS Hassan F Ud Din MTM. Comparative evaluation of single, bilateral intra-testicular injection of hypertonic saline solution and calcium chloride as chemical sterilizing agents in male cats. Biomed J Sci Tech Res. (2019) 21:15858–64. doi: 10.26717/BJSTR.2019.21.003598

[377] Mitra B Samanta PK. Testicular degeneration of scrub bulls by calcium chloride. Indian J Vet Surg. (2000) 21:37–8.

[378] Canpolat I Gur S Gunay C Bulut S Eroksuz H. An evaluation of the outcome of bull castration by intra-testicular injection of ethanol and calcium chloride. Rev Med Vet. (2006) 157:8–9.

[379] Pereira LF Dias FCF Miguel MP Honsho CS Tavares DC Hellú JAA. Testicular histological evaluation and serum testosterone concentrations of bulls after chemical castration with calcium chloride. Pesquisa Veterinária Brasileira. (2018) 38:1554–63. doi: 10.1590/1678-5150-pvb-4945

[380] Jana K Samanta PK Ghosh D. Evaluation of single intratesticular injection of calcium chloride for nonsurgical sterilization of male black Bengal goats (*Capra hircus*): a dose-dependent study. Anim Reprod Sci. (2005) 86:89–108. doi: 10.1016/j.anireprosci.2004.05.021, PMID: 15721661

[381] Martins LT Gonçalves MC Tavares K Gaudêncio S Dos Santos Neto P Dias ALG. Castration methods do not affect weight gain and have diverse impacts on the welfare of water buffalo males. Livestock Sci. (2011) 140:171–6. doi: 10.1016/j.livsci.2011.03.026

[382] Sen CC Yumusak N Faundez R Temamogullari F Taskin A. Evaluation of intra-testicular injections of calcium chloride and 4-vinylcyclohexene 1,2 monoepoxide for chemical sterilization in guinea pigs. Pol J Vet Sci. (2017) 20:251–60. doi: 10.1515/pjvs-2017-0030, PMID: 28865222

[383] Ibrahim A Magdam MA Nasser SA-K Marwa FA. Evaluation of chemical castration with calcium chloride versus surgical castration in donkeys: testosterone as an endpoint marker. BMC Vet Res. (2016) 12:46. doi: 10.1186/s12917-016-0670-3, PMID: 26956100 PMC4784444

[384] Emir L Dadali M Sunay M Erol D Caydere M Ustün H. Chemical castration with intratesticular injection of 20% hypertonic saline: a minimally invasive method. Urol Oncol. (2008) 26:392–6. doi: 10.1016/j.urolonc.2007.05.013, PMID: 18367099

[385] Kwak BK Lee SH. Intratesticular injection of hypertonic saline: non-invasive alternative method for animal castration model. Dev Reprod. (2013) 17:435–40. doi: 10.12717/DR.2013.17.4.435, PMID: 25949160 PMC4382956

[386] Byung Kuk K Sung-Ho L. Evaluation of newly developed chemical castration method: changes in hormone gene expression of hypothalamic-pituitary Axis. Dev Reproduct. (2017) 21:307–15. doi: 10.12717/DR.2017.21.3.307 PMC5651697 29082346

[387] Maadi M-A Behfar M Rasaei A Shalizar-Jalali A Najafi G Mohammadi V. Chemical castration using an intratesticular injection of mannitol: a preliminary study in a rat model. Turkish J Vet Anim Sci. (2021) 45:519–30. doi: 10.3906/vet-2010-111

[388] Canpolat I Karabulut E Eroksuz Y. Chemical castration of adult and non-adult male dogs with sodium chloride solution. IOSR J Agric Vet Sci. (2016) 9:09–11.

[389] Neto OA Gasperin BG Rovani MT Ilha GF Nóbrega JE Jr Mondadori RG. Intratesticular hypertonic sodium chloride solution treatment as a method of chemical castration in cattle. Theriogenology. (2014) 82:1007–1011.e1. doi: 10.1016/j.theriogenology.2014.07.020, PMID: 25149022

[390] Oliveira FC Ferreira CER Haas CS Oliveira LG Mondadori RG Schneider A. Chemical castration in cattle with intratesticular injection of sodium chloride: effects on stress and inflammatory markers. Theriogenology. (2017) 90:114–9. doi: 10.1016/j.theriogenology.2016.12.001, PMID: 28166957

[391] Baqerkhani M Soleimanzadeh A Mohammadi R. Effects of intratesticular injection of hypertonic mannitol and saline on the quality of donkey sperm, indicators of oxidative stress and testicular tissue pathology. BMC Vet Res. (2024) 20:99. doi: 10.1186/s12917-024-03915-1, PMID: 38468237 PMC10926677

[392] Freeman C Coffey DS. Male sterility induced by ethanol injection into the vas deferens. Int J Fertil. (1973) 18:129–32. 4357276

[393] Nakanuma Y Harada K Katayanagi K Tsuneyama K Sasaki M. Definition and pathology of primary sclerosing cholangitis. J Hepato-Biliary-Pancreat Surg. (1999) 6:333–42. doi: 10.1007/s005340050127 10664278

[394] Goldman MP. Sodium tetradecyl sulfate for sclerotherapy treatment of veins: is compounding pharmacy solution safe? Dermatologic Surg. (2004) 30:1454–6; discussion 1456. doi: 10.1111/j.1524-4725.2004.30502.x, PMID: 15606731

[395] Braslis KG Moss DI. Long-term experience with sclerotherapy for treatment of epididymal cyst and hydrocele. Aust N Z J Surg. (1996) 66:222–4. doi: 10.1111/j.1445-2197.1996.tb01169.x, PMID: 8611129

[396] Pineda MH Reimers TJ Faulkner LC Hopwood ML Seidel GE Jr. Azoospermia in dogs induced by injection of sclerosing agents into the caudae of the epididymides. Am J Vet Res. (1977) 38:831–8. PMID: 560154

[397] Pineda MH Hepler DI. Chemical vasectomy in dogs. Long-term study. Theriogenology. (1981) 16:1–11. doi: 10.1016/0093-691X(81)90108-4, PMID: 16725614

[398] Sharma JD Chinoy NJ Dixit VP. Fertility control in vas occluded rats and the biochemical effects of ascorbic acid feeding. Exp Clin Endocrinol. (1983) 82:337–41. doi: 10.1055/s-0029-1210295, PMID: 6686149

[399] Raman G Purandare TV Munshi SR. Sterility induced in male rats by injection of chemical agents into the vas deferens. Andrologia. (1976) 8:321–5. doi: 10.1111/j.1439-0272.1976.tb01663.x, PMID: 1008263

[400] Plant JW Seaman JT Jakovljevic D. Non-surgical sterilisation of rams using a sclerosing agent. Aust Vet J. (1979) 55:263–4. doi: 10.1111/j.1751-0813.1979.tb14706.x 574761

[401] Lewis RW Garcia RR. The results of epididymal ablation by sclerosing agents in the nonhuman primate. Fertil Steril. (1984) 41:465–9. doi: 10.1016/S0015-0282(16)47729-0 6698239

[402] Ansari AS Hussain M Khan SR Lohiya NK. Relative suitability of DMSO and NaHCO3 for reversal of RISUG® induced long-term contraception. Andrology. (2016) 4:306–13. doi: 10.1111/andr.12155 26748683

[403] Kent K Johnston M Strump N Garcia TX. Toward development of the male pill: a decade of potential non-hormonal contraceptive targets. Front Cell Dev Biol. (2020) 8:61. doi: 10.3389/fcell.2020.00061, PMID: 32161754 PMC7054227

[404] Segalla E. Contraception methods in wild animal species: a literature review [thesis]. Padua: Università Degli Studi di Padova (2022). 27 p.

[405] Schwartz E Tornaben JA Boxill GC. Effects of chronic oral administration of a long-acting estrogen, quinestrol, to dogs. Toxicol Appl Pharmacol. (1969) 14:487–94. doi: 10.1016/0041-008X(69)90010-6, PMID: 5787517

[406] Kroopnick JM Lee MS Blithe DL. Development of new hormonal male contraception for the couple. Andrology. (2024). doi: 10.1111/andr.13654, PMID: 38745531

[407] Louwagie EJ Quinn GFL Pond KL Hansen KA. Male contraception: narrative review of ongoing research. Basic Clin Androl. (2023) 33:30. doi: 10.1186/s12610-023-00204-z, PMID: 37940863 PMC10634021

[408] Eşki F Çetin N Uslu S Uslu BA Şendağ S Yörük M. Effects of long-term release GnRH agonist "deslorelin" on testicular HSP expression, accessory sex glands and testicular functions in adult male rats. Theriogenology. (2019) 134:104–11. doi: 10.1016/j.theriogenology.2019.05.016, PMID: 31158733

[409] Romagnoli S Baldan A Ferro S Righetti C Scenna L Gabai G. Length of efficacy and effect of implant location in adult tom cats treated with a 9.4 mg deslorelin subcutaneous implant. J Feline Med Surg. (2019) 21:507–19. doi: 10.1177/1098612X18788157, PMID: 30056772 PMC6537142

[410] Giriboni J Lacuesta L Santiago-Moreno J Ungerfeld R. Chronic use of a GnRH agonist (deslorelin) or immunization against GnRH: effects on testicular function and sperm quality of bucks. Domest Anim Endocrinol. (2020) 71:106395. doi: 10.1016/j.domaniend.2019.106395, PMID: 31731252

[411] Singh G Kumar A Dutt R Arjun V Jain VK. Chemical castration in Animals: an update. Int J Curr Microbiol App Sci. (2020) 9:2787–807. doi: 10.20546/ijcmas.2020.904.329

[412] Grisolia-Romero M Faya M Marchetti C Körber H Goericke-Pesch S Gobello C. Testicular effects of a postnatal GnRH antagonist in domestic cats. Acta Vet Hung. (2022) 70:44–50. doi: 10.1556/004.2021.00052 34908532

[413] Stempel S Körber H Reifarth L Schuler G Goericke-Pesch S. What happens in male dogs after treatment with a 4.7 mg Deslorelin implant? II. Recovery of testicular function after implant removal. Animals (Basel). (2022) 12:12(19). doi: 10.3390/ani12192545 PMC9559295 36230286

[414] Stempel S Körber H Reifarth L Schuler G Goericke-Pesch S. What happens in male dogs after treatment with a 4.7 mg Deslorelin implant? I. Flare up. Downregulat Anim. (2022) 12:379. doi: 10.3390/ani12182379 PMC9495213 36139239

[415] Ferré-Dolcet L Bordogna M Contiero B Fontaine C Bedin S Romagnoli S. Anti-Müllerian hormone concentrations for determining resumption of Sertoli cell function following removal of a 4.7 mg Deslorelin implant in tomcats. Animals (Basel). (2023) 13:552. doi: 10.3390/ani13162552 37627341 PMC10451382

[416] Kolho K-L Nikula H Huhtaniemi I. Sexual maturation of male rats treated postnatally with a gonadotrophin-releasing hormone antagonist. J Endocrinol. (1988) 116:241–6. doi: 10.1677/joe.0.1160241 3127516

[417] Kolho K-L Huhtaniemi I. Suppression of pituitary-testis function in rats treated neonatally with a gonadotrophin-releasing hormone agonist and antagonist: acute and long-term effects. J Endocrinol. (1989) 123:83–91. doi: 10.1677/joe.0.1230083, PMID: 2509621

[418] Kolho KL Huhtaniemi I. Neonatal treatment of male rats with a gonadotropin-releasing hormone antagonist results in altered function of the pituitary-testicular axis in adult age. Biol Reprod. (1989) 41:1084–90. doi: 10.1095/biolreprod41.6.1084, PMID: 2696558

[419] Faya M Marchetti C Priotto M Grisolia M D'Francisco F Gobello C. Postponement of canine puberty by neonatal administration of a long term release GnRH superagonist. Theriogenology. (2018) 118:190–5. doi: 10.1016/j.theriogenology.2018.05.043, PMID: 29913424

[420] Kaya D Gram A Kowalewski MP Schäfer-Somi S Kuru M Boos A. Expression of GnRH receptor in the canine corpus luteum, and luteal function following deslorelin acetate-induced puberty delay. Reprod Domest Anim. (2017) 52:1104–12. doi: 10.1111/rda.13038, PMID: 28963736

[421] Sirivaidyapong S Mehl NS Trigg TE. Delay of puberty and reproductive performance in male dogs following the implantation of 4.7 and 9.4 mg GnRH-agonist deslorelin at an early pre-pubertal age. Reprod Domest Anim. (2012) 47:400–2. doi: 10.1111/rda.12066, PMID: 23279549

[422] de Souza GL Hallak J. Anabolic steroids and male infertility: a comprehensive review. BJU Int. (2011) 108:1860–5. doi: 10.1111/j.1464-410X.2011.10131.x 21682835

[423] Kicman AT. Pharmacology of anabolic steroids. Br J Pharmacol. (2008) 154:502–21. doi: 10.1038/bjp.2008.165 18500378 PMC2439524

[424] Desai A Yassin M Cayetano A Tharakan T Jayasena CN Minhas S. Understanding and managing the suppression of spermatogenesis caused by testosterone replacement therapy (TRT) and anabolic-androgenic steroids (AAS). Ther Adv Urol. (2022) 14:17562872221105017. doi: 10.1177/17562872221105017 35783920 PMC9243576

[425] Turek PJ Williams RH Gilbaugh JH 3rd Lipshultz LI. The reversibility of anabolic steroid-induced azoospermia. J Urol. (1995) 153:1628–30. doi: 10.1016/S0022-5347(01)67482-2, PMID: 7714991

[426] Renaville R Burny A Sneyers M Rochart S Portetelle D Théwis A. Effects of an anabolic treatment before puberty with trenbolone acetate-oestradiol or oestradiol alone on growth rate, testicular development and luteinizing hormone and testosterone plasma concentrations. Theriogenology. (1988) 29:461–76. doi: 10.1016/0093-691X(88)90248-8, PMID: 16726369

[427] Ventanas J Sancho G Garcia-Regueiro JA Antequera T Martinez M López-Bote C. Testicular development, androstenone levels and androstenone odour of untreated and trenbolone implanted boars. J Sci Food Agric. (1991) 57:127–33. doi: 10.1002/jsfa.2740570114

[428] Godfrey RW Lunstra DD Schanbacher BD. Effect of implanting bull calves with testosterone propionate, dihydrotestosterone propionate or oestradiol-17β prepubertally on the pituitary–testicular axis and on postpubertal social and sexual behaviour. Reproduction. (1992) 94:57–69. doi: 10.1530/jrf.0.0940057, PMID: 1552493

[429] Pfau DR Schwartz AR Dela Cruz C Padmanabhan V Moravek MB Shikanov A. A mouse model to investigate the impact of gender affirming hormone therapy with estradiol on reproduction. Adv Biol. (2023):e2300126. doi: 10.1002/adbi.202300126 PMC10920391 37688350

[430] Matoso A Khandakar B Yuan S Wu T Wang LJ Lombardo KA. Spectrum of findings in orchiectomy specimens of persons undergoing gender confirmation surgery. Hum Pathol. (2018) 76:91–9. doi: 10.1016/j.humpath.2018.03.007, PMID: 29555572

[431] Schneider F Neuhaus N Wistuba J Zitzmann M Heß J Mahler D. Testicular functions and clinical characterization of patients with gender dysphoria (GD) undergoing sex reassignment surgery (SRS). J Sex Med. (2015) 12:2190–200. doi: 10.1111/jsm.13022, PMID: 26559385

[432] Tassinari R Maranghi F. Rodent model of gender-affirming hormone therapies as specific tool for identifying susceptibility and vulnerability of transgender people and future applications for risk assessment. Int J Environ Res Public Health. (2021) 18:12640. doi: 10.3390/ijerph182312640, PMID: 34886364 PMC8656759

[433] Swanson HE Werfftenbosch JJ. The "early-androgen" syndrome; differences in response to pre-natal and post-natal administration of various doses of testosterone propionate in female and male rats. Acta Endocrinol. (1964) 47:37–50. doi: 10.1530/acta.0.0470037, PMID: 14208150

[434] Swanson HE van Werff ten Bosch JJ. Modification of male rat reproductive tract development by a single injection of testosterone propionate shortly after birth. Acta Endocrinol. (1965) 50:310–6. doi: 10.1530/acta.0.0500310, PMID: 5897729

[435] Kincl FA Pi AF Maqueo M Lasso LH Dorfman RI Oriol A. Inhibition of sexual development in male and female rats treated with various steroids at the age of five days. Acta Endocrinol. (1965) 49:193–206. PMID: 14303249 10.1530/acta.0.0490193

[436] Harris GW Levine S. Sexual differentiation of the brain and its experimental control. J Physiol. (1965) 181:379–400. doi: 10.1113/jphysiol.1965.sp007768 5893725 PMC1357646

[437] Feder HH. Specificity of testosterone and estradiol in the differentiating neonatal rat. Anat Rec. (1967) 157:79–86. doi: 10.1002/ar.1091570112 6030761

[438] Feigelson M. Suppression of testicular maturation and fertility following androgen administration to neonatal male Rats1. Biol Reprod. (1986) 35:1321–32. doi: 10.1095/biolreprod35.5.1321, PMID: 3828440

[439] Vanderstichele H Eechaute W Lacroix E Leusen I. The effects of neonatal androgenization of male rats on testosterone metabolism by the hypothalamus-pituitary-gonadal axis. J Steroid Biochem. (1987) 26:493–7. doi: 10.1016/0022-4731(87)90062-8, PMID: 3108585

[440] Hess RA Sharpe RM Hinton BT. Estrogens and development of the rete testis, efferent ductules, epididymis and vas deferens. Differentiation. (2021) 118:41–71. doi: 10.1016/j.diff.2020.11.004, PMID: 33441255 PMC8026493

[441] Hess RA Gist DH Bunick D Lubahn DB Farrell A Bahr J. Estrogen receptor (alpha and beta) expression in the excurrent ducts of the adult male rat reproductive tract. J Androl. (1997) 18:602–11. doi: 10.1002/j.1939-4640.1997.tb02437.x, PMID: 9432133

[442] Rajpert-De Meyts E Almstrup K Skakkebæk NE. Testicular dysgenesis syndrome and carcinoma in situ testis In: Ježek D, editor. Atlas on the Human testis. London: Springer (2013). 159–78.

[443] Juul A Almstrup K Andersson AM Jensen TK Jorgensen N Main KM. Possible fetal determinants of male infertility. Nat Rev Endocrinol. (2014) 10:553–62. doi: 10.1038/nrendo.2014.97 24935122

[444] Nistal M González-Peramato P Serrano Á. Testicular dysgenesis syndrome (TDS) In: Clues in the diagnosis of non-tumoral testicular pathology. Cham: Springer International Publishing (2017). 101–9.

[445] Sharpe RM. Fetal life shapes adult male reproductive function. Lancet Child Adolesc Health. (2018) 2:695–6. doi: 10.1016/s2352-4642(18)30276-1 30236372

[446] Ivell R Mamsen LS Andersen CY Anand-Ivell R. Expression and role of INSL3 in the fetal testis. Front Endocrinol. (2022) 13:868313. doi: 10.3389/fendo.2022.868313, PMID: 35464060 PMC9019166

[447] Li D Ping H Li K Lin J Jiang X Zhang X. Environmental oestrogens disrupt testicular descent and damage male reproductive health: mechanistic insight. J Cell Mol Med. (2023) 27:2095–102. doi: 10.1111/jcmm.17837, PMID: 37409668 PMC10399541

[448] Toppari J Virtanen H Skakkebaek NE Main KM. Environmental effects on hormonal regulation of testicular descent. J Steroid Biochem Mol Biol. (2006) 102:184–6. doi: 10.1016/j.jsbmb.2006.09.020 17049842

[449] Sharpe RM Skakkebaek NE. Testicular dysgenesis syndrome: mechanistic insights and potential new downstream effects. Fertil Steril. (2008) 89:e33–8. doi: 10.1016/j.fertnstert.2007.12.026, PMID: 18308057

[450] Sonne SB Kristensen DM Novotny GW Olesen IA Nielsen JE Skakkebaek NE. Testicular dysgenesis syndrome and the origin of carcinoma in situ testis. Int J Androl. (2008) 31:275–87. doi: 10.1111/j.1365-2605.2007.00855.x, PMID: 18205797

[451] Main KM Skakkebaek NE Toppari J. Cryptorchidism as part of the testicular dysgenesis syndrome: the environmental connection. Endocr Dev. (2009) 14:167–73. doi: 10.1159/000207485, PMID: 19293583

[452] Skakkebaek NE Rajpert-De Meyts E Buck Louis GM Toppari J Andersson AM Eisenberg ML. Male reproductive disorders and fertility trends: influences of environment and genetic susceptibility. Physiol Rev. (2016) 96:55–97. doi: 10.1152/physrev.00017.2015, PMID: 26582516 PMC4698396

[453] van den Driesche S Kilcoyne KR Wagner I Rebourcet D Boyle A Mitchell R. Experimentally induced testicular dysgenesis syndrome originates in the masculinization programming window. JCI Insight. (2017) 2:e91204. doi: 10.1172/jci.insight.91204 28352662 PMC5358493

[454] Soto-Heras S Reinacher L Wang B Oh JE Bunnell M Park CJ. Cryptorchidism and testicular cancer in the dog: unresolved questions and challenges in translating insights from human studies. Biol Reprod. (2024). doi: 10.1093/biolre/ioae075, PMID: 38738783

[455] Dobbs RW Malhotra NR Greenwald DT Wang AY Prins GS Abern MR. Estrogens and prostate cancer. Prostate Cancer Prostatic Dis. (2019) 22:185–94. doi: 10.1038/s41391-018-0081-6 30131606

[456] Prins GS. Developmental estrogenization: prostate gland reprogramming leads to increased disease risk with aging. Differentiation. (2021) 118:72–81. doi: 10.1016/j.diff.2020.12.001 33478774 PMC8026668

[457] Makela JA Koskenniemi JJ Virtanen HE Toppari J. Testis Development. Endocr Rev. (2019) 40:857–905. doi: 10.1210/er.2018-00140 30590466

[458] Rodprasert W Virtanen HE Makela JA Toppari J. Hypogonadism and cryptorchidism. Front Endocrinol. (2020) 10:906. doi: 10.3389/fendo.2019.00906, PMID: 32010061 PMC6974459

[459] Yin Y DeWolf WC Morgentaler A. Experimental cryptorchidism induces testicular germ cell apoptosis by p53-dependent and -independent pathways in mice. Biol Reprod. (1998) 58:492–6. doi: 10.1095/biolreprod58.2.492 9475406

[460] Jhun H Lee WY Park JK Hwang SG Park HJ. Transcriptomic analysis of testicular gene expression in a dog model of experimentally induced cryptorchidism. Cells. (2022) 11:2476. doi: 10.3390/cells11162476, PMID: 36010553 PMC9406621

[461] Zheng Z-H Tian Q He J-P Yuan J-L Yang S-H Liu J-L. Comparative transcriptome analysis of experimental cryptorchidism: of mice and cynomolgus monkeys. Physiol Genomics. (2022) 54:187–95. doi: 10.1152/physiolgenomics.00010.2022, PMID: 35468005

[462] Amann RP Veeramachaneni DN. Cryptorchidism in common eutherian mammals. Reproduction. (2007) 133:541–61. doi: 10.1530/REP-06-0272 17379650

[463] Rajfer J Walsh PC. Hormonal regulation of testicular descent: experimental and clinical observations. J Urol. (1977) 118:985–90. doi: 10.1016/S0022-5347(17)58273-7, PMID: 21972

[464] Kogan BA Gupta R Juenemann KP. Fertility in cryptorchidism: further development of an experimental model. J Urol. (1987) 137:128–31. doi: 10.1016/S0022-5347(17)43903-6, PMID: 2879046

[465] Griffiths AL Hutson JM. Testicular descent: the role of oestrogen in gubernacular migration and inguinoscrotal testicular descent. Pediatr Surg Int. (1993) 8:322–8. doi: 10.1007/BF00173356

[466] Aceitero J Llanero M Parrado R Pena E Lopez-Beltran A. Neonatal exposure of male rats to estradiol benzoate causes rete testis dilation and backflow impairment of spermatogenesis. Anat Rec. (1998) 252:17–33. doi: 10.1002/(SICI)1097-0185(199809)252:1<17::AID-AR3>3.0.CO;2-B, PMID: 9737741

[467] Hay B.A. Li J. Antibody-Mediated Immunocontraception, in US Patent Office. (2014).

[468] Junco JA Fuentes F Millar RP. A dual kisspeptin-GnRH immunogen for reproductive immunosterilization. Vaccine. (2021) 39:6437–48. doi: 10.1016/j.vaccine.2021.07.080, PMID: 34489132

[469] Ahmed S Bo D Zhao J Liu G Ding Y Jiang X. Immunocastration with gene vaccine (KISS1) induces a cell-mediated immune response in ram testis: a transcriptome evaluation. Reprod Domest Anim. (2022) 57:653–64. doi: 10.1111/rda.14106 35247007

[470] Needham T Lambrechts H Hoffman LC. Castration of male livestock and the potential of immunocastration to improve animal welfare and production traits: invited review. South African J Anim Sci. (2017) 47:731–42. doi: 10.4314/sajas.v47i6.1

[471] Tesema B Zhao JY Jiang XP Liu GQ Han YG Wassie T. Kisspeptin recombinant oral vaccine: a master gene vaccine inhibiting the reproductive physiology and behavior of ram lambs. Vaccine. (2019) 37:4630–6. doi: 10.1016/j.vaccine.2017.09.001 29097006

[472] Miller LA Gionfriddo JP Fagerstone KA Rhyan JC Killian GJ. The single-shot GnRH immunocontraceptive vaccine (GonaCon) in white-tailed deer: comparison of several GnRH preparations. Am J Reprod Immunol. (2008) 60:214–23. doi: 10.1111/j.1600-0897.2008.00616.x 18782282

[473] Wang-Cahill FWJ Hall T. Use of GonaCon in wildlife damage management risk assessment In: Ali N O'Hare J Ruell EW, editors. Human health and ecological risk assessment for the use of wildlife damage management methods. Washington, DC: APHIS Wildlife Services (2022)

[474] Levy JK. Contraceptive vaccines for the humane control of community cat populations. Am J Reprod Immunol. (2011) 66:63–70. doi: 10.1111/j.1600-0897.2011.01005.x, PMID: 21501281 PMC5567843

[475] Bradford JB Hobbs NT. Regulating overabundant ungulate populations: an example for elk in Rocky Mountain National Park, Colorado. J Environ Manag. (2008) 86:520–8. doi: 10.1016/j.jenvman.2006.12.005, PMID: 17276577

[476] Kirkpatrick JF Lyda RO Frank KM. Contraceptive vaccines for wildlife: a review. Am J Reprod Immunol. (2011) 66:40–50. doi: 10.1111/j.1600-0897.2011.01003.x, PMID: 21501279

[477] Kaur K Prabha V. Immunocontraceptives: new approaches to fertility control. Biomed Res Int. (2014) 2014:868196. doi: 10.1155/2014/868196 25110702 PMC4119744

[478] Bronson R Cooper G Rosenfeld D. Sperm antibodies: their role in infertility. Fertil Steril. (1984) 42:171–83. doi: 10.1016/S0015-0282(16)48009-X, PMID: 6378672

[479] Frayne J Hall L. The potential use of sperm antigens as targets for immunocontraception; past, present and future. J Reprod Immunol. (1999) 43:1–33. doi: 10.1016/S0165-0378(99)00005-4 10392779

[480] Ohl DA Naz RK. Infertility due to antisperm antibodies. Urology. (1995) 46:591–602. doi: 10.1016/S0090-4295(99)80282-9 7571238

[481] Baskin MJ. Temporary sterilization by the injection of human spermatozoa. A preliminary report. Am J Obstet Gynecol. (1932) 24:892–7. doi: 10.1016/S0002-9378(32)91129-6

[482] Bandivdekar AH Vernekar VJ Kamada M Raghavan VP. Antifertility effect of passive administration of antibodies to 80 kDa human sperm antigen and its synthetic peptides in male and female rats. Am J Reprod Immunol. (2005) 54:332–41. doi: 10.1111/j.1600-0897.2005.00309.x, PMID: 16305658

[483] Remy JJ Bozon V Couture L Goxe B Salesse R Garnier J. Suppression of fertility in male mice by immunization against LH receptor. J Reprod Immunol. (1993) 25:63–79. doi: 10.1016/0165-0378(93)90042-G, PMID: 8271240

[484] Jeyakumar M Moudgal NR. Immunization of male rabbits with sheep luteal receptor to LH results in production of antibodies exhibiting hormone-agonistic and -antagonistic activities. J Endocrinol. (1996) 150:431–43. doi: 10.1677/joe.0.1500431, PMID: 8882162

[485] Moudgal N Jeyakumar M Krishnamurthy H Sridhar S Krihsnamurthy H Martin F. Development of male contraceptive vaccine – a perspective. Hum Reprod Update. (1997) 3:335–46. doi: 10.1093/humupd/3.4.335, PMID: 9459279

[486] Moudgal NR Sairam MR Krishnamurthy HN Sridhar S Krishnamurthy H Khan H. Immunization of male bonnet monkeys (*M. radiata*) with a recombinant FSH receptor preparation affects testicular function and fertility. Endocrinology. (1997) 138:3065–8. doi: 10.1210/endo.138.7.5381 9202254

[487] Murty GS Rani CS Moudgal NR Prasad MR. Effect of passive immunization with specific antiserum to FSH on the spermatogenic process and fertility of adult male bonnet monkeys (*Macaca radiata*). J Reprod Fertil Suppl. (1979) 26:147–63. 118251

[488] Moudgal NR Ravindranath N Murthy GS Dighe RR Aravindan GR Martin F. Long-term contraceptive efficacy of vaccine of ovine follicle-stimulating hormone in male bonnet monkeys (*Macaca radiata*). J Reprod Fertil. (1992) 96:91–102. doi: 10.1530/jrf.0.0960091, PMID: 1432977

[489] Yan P He W Liang Z Chen Z Shang X He H. A novel dominant B-cell epitope of FSHR identified by molecular docking induced specific immune response and suppressed fertility. Gynecol Endocrinol. (2009) 25:828–38. doi: 10.3109/09513590903015536, PMID: 19906003

[490] Yan P He W Wu Y Chen Z He H Ni B. Enhanced suppression of fertility can be achieved by priming with FSHR and Eppin and further boosting with their B-cell epitope peptides. Am J Reprod Immunol. (2015) 74:156–68. doi: 10.1111/aji.12381, PMID: 25864521

[491] Flanagan CA Manilall A. Gonadotropin-releasing hormone (GnRH) receptor structure and GnRH binding. Front Endocrinol. (2017) 8:274. doi: 10.3389/fendo.2017.00274, PMID: 29123501 PMC5662886

[492] Upadhyay SN Alam A Talwar GP. Functional morphology of testis and its excurrent ducts in rats immunized with synthetic luteinizing hormone releasing hormone conjugated to tetanus toxoid. J Reprod Immunol. (1989) 16:151–63. doi: 10.1016/0165-0378(89)90024-7, PMID: 2689646

[493] Rovan E Fiebiger E Kalla NR Talwar GP Aulitzky W Frick J. Effect of active immunization to luteinizing-hormone-releasing hormone on the fertility and histoarchitecture of the reproductive organs of male rat. Urol Res. (1992) 20:323–34. doi: 10.1007/BF00922744, PMID: 1455565

[494] Quesnell MM Zhang Y de Avila DM Bertrand KP Reeves JJ. Immunization of male mice with luteinizing hormone-releasing hormone fusion proteins reduces testicular and accessory sex gland function. Biol Reprod. (2000) 63:347–53. doi: 10.1095/biolreprod63.1.347, PMID: 10859278

[495] Han XF Li JL Zhou YQ Ren XH Liu GC Cao XH. Active immunization with GnRH-tandem-dimer peptide in young male rats reduces serum reproductive hormone concentrations, testicular development and spermatogenesis. Asian J Androl. (2016) 18:485–91. doi: 10.4103/1008-682X.156856, PMID: 26208395 PMC4854110

[496] Han XF Cao XH Tang J Du XG Zeng XY. Active immunization against GnRH reduces the synthesis of GnRH in male rats. Theriogenology. (2013) 80:1109–16. doi: 10.1016/j.theriogenology.2013.08.014 24084232

[497] Han X Zhou Y Zeng Y Sui F Liu Y Tan Y. Effects of active immunization against GnRH versus surgical castration on hypothalamic-pituitary function in boars. Theriogenology. (2017) 97:89–97. doi: 10.1016/j.theriogenology.2017.04.038, PMID: 28583614

[498] Rosenfield DA Nichi M Losano JDA Kawai G Leite RF Acosta AJ. Field-testing a single-dose immunocontraceptive in free-ranging male capybara (*Hydrochoerus hydrochaeris*): evaluation of effects on reproductive physiology, secondary sexual characteristics, and agonistic behavior. Anim Reprod Sci. (2019) 209:106148. doi: 10.1016/j.anireprosci.2019.106148, PMID: 31514916

[499] Pinkham R Koon KK To, J Chan J Vial F Gomm M. Long-term effect of a GnRH-based immunocontraceptive on feral cattle in Hong Kong. PLoS One. (2022) 17:e0272604. doi: 10.1371/journal.pone.0272604, PMID: 35976896 PMC9385044

[500] Frey RK Wehtje ME Nol P Clarke PR Rhyan JC McCollum MP. Effects of the Immunocontraceptive Gonacon on pregnancy in Brucella-seropositive Bison (*Bison bison*). J Wildl Dis. (2024) 60:339–45. doi: 10.7589/JWD-D-21-00168, PMID: 38373061

[501] Shiels AB Runte J Ruell EW Eckery DC Witmer GW Salkeld DJ. Treatment with the immunocontraceptive vaccine, GonaCon, induces temporary fertility control in free-ranging prairie dog populations in Colorado, USA. Wildl Res. (2024) 51:1–10. doi: 10.1071/WR22135

[502] Thompson DL. Immunization against GnRH in male species (comparative aspects). Anim Reprod Sci. (2000) 60-61:459–69. doi: 10.1016/S0378-4320(00)00116-0 10844216

[503] Patterson RLS. 5α-androst-16-ene-3-one:—compound responsible for taint in boar fat. J Sci Food Agric. (1968) 19:31–8. doi: 10.1002/jsfa.2740190107

[504] Babol J Squires EJ Lundstrom K. Relationship between metabolism of androstenone and skatole in intact male pigs. J Anim Sci. (1999) 77:84–92. doi: 10.2527/1999.77184x, PMID: 10064031

[505] Purswell BJ Kolster KA. Immunocontraception in companion animals. Theriogenology. (2006) 66:510–3. doi: 10.1016/j.theriogenology.2006.04.018, PMID: 16837035

[506] Munks MW. Progress in development of immunocontraceptive vaccines for permanent non-surgical sterilization of cats and dogs. Reprod Domest Anim. (2012) 47:223–7. doi: 10.1111/j.1439-0531.2012.02079.x, PMID: 22827374

[507] Claus R Lacorn M Danowski K Pearce MC Bauer A. Short-term endocrine and metabolic reactions before and after second immunization against GnRH in boars. Vaccine. (2007) 25:4689–96. doi: 10.1016/j.vaccine.2007.04.009, PMID: 17485149

[508] Stojanovic S Uscebrka G Zikic D Stukelj M. Histological and morphometric examination of the testes of boars and male pigs Immunocastrated with Improvac®. Acta Sci Vet. (2017) 45:7. doi: 10.22456/1679-9216.80582

[509] Lugar DW Rhoads ML Clark-Deener SG Callahan SR Revercomb AK Prusa KJ. Immunological castration temporarily reduces testis size and function without long-term effects on libido and sperm quality in boars. Animal. (2017) 11:643–9. doi: 10.1017/S1751731116002081, PMID: 27786141

[510] Pinna A Schivazappa C Virgili R Parolari G. Effect of vaccination against gonadotropin-releasing hormone (GnRH) in heavy male pigs for Italian typical dry-cured ham production. Meat Sci. (2015) 110:153–9. doi: 10.1016/j.meatsci.2015.07.002, PMID: 26225931

[511] Pesenti Rossi G Dalla Costa E Filipe JFS Mazzola SM Motta A Borciani M. Does Immunocastration affect behaviour and body lesions in heavy pigs? Vet Sci. (2022) 9:410. doi: 10.3390/vetsci9080410 36006325 PMC9414783

[512] de Roest K Montanari C Fowler T Baltussen W. Resource efficiency and economic implications of alternatives to surgical castration without anaesthesia. Animal. (2009) 3:1522–31. doi: 10.1017/S1751731109990516, PMID: 22444985

[513] Nautrup BP Van Vlaenderen I Aldaz A Mah CK. The effect of immunization against gonadotropin-releasing factor on growth performance, carcass characteristics and boar taint relevant to pig producers and the pork packing industry: a meta-analysis. Res Vet Sci. (2018) 119:182–95. doi: 10.1016/j.rvsc.2018.06.002, PMID: 29958153

[514] Palma-Granados P Lara L Seiquer I Lachica M Fernández-Fígares I Haro A. Protein retention, growth performance and carcass traits of individually housed immunocastrated male- and female- and surgically castrated male Iberian pigs fed diets of increasing amino acid concentration. Animal. (2021) 15:100187. doi: 10.1016/j.animal.2021.100187, PMID: 33637438

[515] Turkstra JA Zengt XY van Diepent JT Jongbloed AW Oonk HB van de Wielt DF. Performance of male pigs immunized against GnRH is related to the time of onset of biological response. J Anim Sci. (2002) 80:2953–9. doi: 10.2527/2002.80112953x 12462264

[516] Monleon E Noya A Carmen Garza M Ripoll G Sanz A. Effects of an anti-gonadotrophin releasing hormone vaccine on the morphology, structure and function of bull testes. Theriogenology. (2020) 141:211–8. doi: 10.1016/j.theriogenology.2019.07.019, PMID: 31387698

[517] Price EO Adams TE Huxsoll CC Borgwardt RE. Aggressive behavior is reduced in bulls actively immunized against gonadotropin-releasing hormone. J Anim Sci. (2003) 81:411–5. doi: 10.2527/2003.812411x, PMID: 12643484

[518] Janett F Gerig T Tschuor AC Amatayakul-Chantler S Walker J Howard R. Effect of vaccination against gonadotropin-releasing factor (GnRF) with Bopriva(R) in the prepubertal bull calf. Anim Reprod Sci. (2012) 131:72–80. doi: 10.1016/j.anireprosci.2012.02.012, PMID: 22440457

[519] Ulker H Kanter M Gokdal O Aygun T Karakus F Sakarya ME. Testicular development, ultrasonographic and histological appearance of the testis in ram lambs immunized against recombinant LHRH fusion proteins. Anim Reprod Sci. (2005) 86:205–19. doi: 10.1016/j.anireprosci.2004.07.011, PMID: 15766801

[520] Ulker H Yilmaz A Karakus F Yoruk M Budag C deAvila DM. LHRH fusion protein immunization alters testicular development, ultrasonographic and histological appearance of ram testis. Reprod Domest Anim. (2009) 44:593–9. doi: 10.1111/j.1439-0531.2007.01024.x, PMID: 19019064

[521] Kiyma Z Adams TE Hess BW Riley ML Murdoch WJ Moss GE. Gonadal function, sexual behavior, feedlot performance, and carcass traits of ram lambs actively immunized against GnRH. J Anim Sci. (2000) 78:2237–43. doi: 10.2527/2000.7892237x, PMID: 10985393

[522] Godfrey SI Walkden-Brown SW Martin GB Speijers EJ. Immunisation of goat bucks against GnRH to prevent seasonal reproductive and agonistic behaviour. Anim Reprod Sci. (1996) 44:41–54. doi: 10.1016/0378-4320(96)01479-0

[523] Levy JK Miller LA Cynda Crawford P Ritchey JW Ross MK Fagerstone KA. GnRH immunocontraception of male cats. Theriogenology. (2004) 62:1116–30. doi: 10.1016/j.theriogenology.2003.12.025, PMID: 15289051

[524] Robbins SC Jelinski MD Stotish RL. Assessment of the immunological and biological efficacy of two different doses of a recombinant GnRH vaccine in domestic male and female cats (*Felis catus*). J Reprod Immunol. (2004) 64:107–19. doi: 10.1016/j.jri.2004.08.004, PMID: 15596230

[525] Lee YJ Jo EJ Lee HW Hwang BR Kim YH Park BJ. Evaluation of infertility efficacy of the *E. coli* expressed STF2-GnRH vaccine in male cats. J Vet Sci. (2019) 20:e30. doi: 10.4142/jvs.2019.20.e30 31161748 PMC6538513

[526] Ladd A Tsong YY Walfield AM Thau R. Development of an antifertility vaccine for pets based on active immunization against luteinizing hormone-releasing hormone. Biol Reprod. (1994) 51:1076–83. doi: 10.1095/biolreprod51.6.1076, PMID: 7888486

[527] Jung MJ Moon YC Cho IH Yeh JY Kim SE Chang WS. Induction of castration by immunization of male dogs with recombinant gonadotropin-releasing hormone (GnRH)-canine distemper virus (CDV) T helper cell epitope p35. J Vet Sci. (2005) 6:21–4. doi: 10.4142/jvs.2005.6.1.21, PMID: 15785119

[528] Siel D Ubilla M Vidal S Loaiza A Quiroga J Cifuentes F. Reproductive and behavioral evaluation of a new Immunocastration dog vaccine. Animals. (2020) 10:226. doi: 10.3390/ani10020226, PMID: 32023851 PMC7070807

[529] Donovan CE Greer M Kutzler MA. Physiologic responses following gonadotropin-releasing hormone immunization in intact male dogs. Reprod Domest Anim. (2012) 47:403–5. doi: 10.1111/rda.12017, PMID: 23279550

[530] Ochoa JS Favre RN García MF Stornelli MC Sangache WC Rearte R. Immunocontraception of male domestic cats using GnRH vaccine Improvac. Theriogenology. (2023) 198:211–6. doi: 10.1016/j.theriogenology.2022.12.020 36610370

[531] Yang J Zhou Z Li G Dong Z Li Q Fu K. Oral immunocontraceptive vaccines: a novel approach for fertility control in wildlife. Am J Reprod Immunol. (2023) 89:e13653. doi: 10.1111/aji.13653, PMID: 36373212

[532] De Nys HM Bertschinger HJ Turkstra JA Colenbrander B Palme R Human AM. Vaccination against GnRH may suppress aggressive behaviour and musth in African elephant (*Loxodonta africana*) bulls – a pilot study. J S Afr Vet Assoc. (2010) 81:8–15. doi: 10.4102/jsava.v81i1.88, PMID: 20649148

[533] Musa AS Needham T Kotrba R Ceacero F. Activity and social behaviour of farmed common eland (*Taurotragus oryx*), and the effect of immunocastration thereon. Appl Anim Behav Sci. (2024) 272:106189. doi: 10.1016/j.applanim.2024.106189

[534] Chitambala T Ny V Ceacero F Barton L Bures D Kotrba R. Effects of Immunocastration and amino acid supplementation on yearling fallow deer (*Dama dama*) testes development. Animals (Basel). (2023) 14:115. doi: 10.3390/ani14010115 38200846 PMC10778327

[535] Ceacero F Ny V Kotrba R Barton L Cupic S Bureš D. Combined effects of supplementation of amino acids and immunocastration in first antler growth of farmed fallow deer (*Dama dama*). Anim Prod Sci. (2023) 63:1583–93. doi: 10.1071/AN22258

[536] Han Y Liu G Jiang X Ijaz N Tesema B Xie G. KISS1 can be used as a novel target for developing a DNA immunocastration vaccine in ram lambs. Vaccine. (2015) 33:777–82. doi: 10.1016/j.vaccine.2014.12.054, PMID: 25562792

[537] Han YG Liu GQ Jiang XP Xiang XL Huang YF Nie B. Reversibility and safety of KISS1 metastasis suppressor gene vaccine in immunocastration of ram lambs. Asian Australas J Anim Sci. (2018) 31:835–41. doi: 10.5713/ajas.17.0629, PMID: 29268573 PMC5933981

[538] Wassie T Fanmei Z Jiang X Liu G Girmay S Min Z. Recombinant B2L and Kisspeptin-54 DNA vaccine induces immunity against Orf virus and inhibits spermatogenesis in rats. Sci Rep. (2019) 9:16262. doi: 10.1038/s41598-019-52744-y, PMID: 31700161 PMC6838309

[539] Wassie T Zeng F Jiang X Liu G Kasimu H Ling S. Effect of Kisspeptin-54 immunization on performance, carcass characteristics, meat quality and safety of Yiling goats. Meat Sci. (2020) 166:108139. doi: 10.1016/j.meatsci.2020.108139 32289558

[540] Han Y Si W Han Y Na R Zeng Y Yang EG. Immunization with oral KISS1 DNA vaccine inhibits testicular Leydig cell proliferation mainly via the hypothalamic-pituitary-testicular axis and apoptosis-related genes in goats. Anim Biotechnol. (2021) 32:395–9. doi: 10.1080/10495398.2019.1697701 31805804

[541] Tesema B Liu GQ Jiang XP. Active kisspeptin DNA vaccines oral immunization disrupt mRNA hormone receptors expression in ram lambs. Anim Biotechnol. (2022) 34:2285–94. doi: 10.1080/10495398.2022.2087665 35714982

[542] D'Occhio MJ. Immunological suppression of reproductive functions in male and female mammals. Anim Reprod Sci. (1993) 33:345–72. doi: 10.1016/0378-4320(93)90123-9

[543] Han X Cheng W Chen Z Du X Cao X Zeng X. Active immunisation against pregnenolone reduces testicular steroidogenesis and GnRH synthesis in rabbits. Anim Reprod Sci. (2014) 145:161–9. doi: 10.1016/j.anireprosci.2014.01.016, PMID: 24576399

[544] Naz RK Rajesh C. Passive immunization for immunocontraception: lessons learned from infectious diseases. Front Biosci. (2004) 9:2457–65. doi: 10.2741/1407 15353298

[545] Wang M. Intra-testicular injection of Immunogens, in US Patent office. Islandia, NY: Ark Sciences, Inc. (2014).

[546] Fischer K Schnieke A. How genome editing changed the world of large animal research. Front Genome Edit. (2023) 5:1272687. doi: 10.3389/fgeed.2023.1272687, PMID: 37886655 PMC10598601

[547] Happi Mbakam C Lamothe G Tremblay G Tremblay JP. CRISPR-Cas9 gene therapy for Duchenne muscular dystrophy. Neurotherapeutics. (2022) 19:931–41. doi: 10.1007/s13311-022-01197-9, PMID: 35165856 PMC9294086

[548] Yunes MC Teixeira DL von Keyserlingk MAG Hötzel MJ. Is gene editing an acceptable alternative to castration in pigs? PLoS One. (2019) 14:e0218176. doi: 10.1371/journal.pone.0218176, PMID: 31233520 PMC6590801

[549] Fahrenkrug SC Carlson DF. Genetically Sterile Animals, Patent, U.S. St. Paul, MN: Recombinetics, Inc. (2019).

[550] Sonstegard T Fahrenkrug S Carlson D. 307 precision animal breeding to make genetically castrated animals for improved animal welfare and alternative breeding applications. J Anim Sci. (2017) 95:149–50. doi: 10.2527/asasmw.2017.307

[551] Huijsmans M. (2019). The Alliance to end surgical castration of swine announces precision breeding successes. News; Available at: https://www.hendrix-genetics.com/en/news/alliance-end-surgical-castration-swine-announces-precision-breeding-successes/

[552] Flórez JM Martins K Solin S Bostrom JR Rodríguez-Villamil P Ongaratto F. CRISPR/Cas9-editing of KISS1 to generate pigs with hypogonadotropic hypogonadism as a castration free trait. Front Genet. (2023) 13:1078991. doi: 10.3389/fgene.2022.1078991, PMID: 36685939 PMC9854396

