# Supplementary material for: Male animal sterilization: history, current practices, and potential methods for replacing castration
Source: Front Vet Sci. 2024 Jul 3;11:1409386. doi: 10.3389/fvets.2024.1409386 (PMC11255590; doi:10.3389/fvets.2024.1409386)
Supplement: Supplementary file 1 [file Table_1.pdf]

Supplemental Table 1 References. Non-hormonal Chemicals that Induce Potential Sterilizing Effects in Male Animals

Target Organ	Admin[1]	Chemical	Trade Name[3]	Species	Dosage	Original Use	Short-term Pathology	Long-term Pathology	Mechanisms of action	T Decreased[1]	Inflammation	Side-Effects	Refs[6]
Testis	Oral, s.c.	Ketoconazole	Nizoral	Mouse, Rat, Dog, Monkey	10-300 mg/kg	Imidazole antifungal drug	Germ cell apoptosis	Decrease testis wt; germ cell loss; testicular atrophy; azoospermia	Inhibition of cytochrome p450 enzymes, blocking steroidogenesis; reduces testosterone	Yes	Yes	Inhibits steroid synthesis; central nervous system toxicity; adrenal insufficiency	1
		Embelin	N/A	Dog, Rat, Mouse	20-80 mg/kg	Anti-cancer	Reversible infertility; decrease T	Spermatogenesis arrest; decrease testis wt	Inhibition of energy metabolism and sperm motility	Yes	No	None	2
		Dibromochloro-propane	N/A	Rat	50 mg/kg	Nematicide	Multinucleated giant cells; sperm granuloma	Azoospermia	Inhibit sperm glucose metabolism	No	No	Damage to liver, kidneys, stomach	3
		4-Vinylcyclo-hexene diepoxide	N/A	Rat	100-500 mg/kg	Industrial chemical	Decrease sperm count; decrease T	Testis necrosis	Induces oxidative stress	Yes	Yes	Liver, kidney, stomach and brain toxicity	4
		20,25-diazacholesterol dihydrochloride	DiazaCon™	Rat, Mouse, Bird, Squirrel	8-100 mg/kg	Lower serum cholesterol	Decrease T; germ cell loss; multinucleated giant cells	Leydig cell degeneration; testis atrophy	Inhibits steroidogenesis	Yes	No	Inhibits synthesis of glucocorticoids	5
		Quinestrol and Levonorgestrel	EP-1	Rat, wild rodent	0.33-0.67 mg/kg	Female contraceptive; synthetic hormones	Decreased epididymal weight; decreased cauda sperm number; germ cell loss	Testis recovers	Inhibits HPG axis	No	Not determined	Increased weight adrenal gland	6
Testicular and epididymis	Oral, s.c.	Methyl 1-(butylcarbamoyl)-2-benzimidazole-carbamate	Benomyl (Benlate) Carbendazim	Rat	25-500 mg/kg	Fungicides	Increase testis wt; sloughing germ cells; inhibit mitosis; occlusion of efferent ducts	Testis atrophy; decrease testis wt; fibrosis of efferent ducts; azoospermia	Inhibits microtubule polymerization; occlusion of efferent ducts	No	Yes	Hepatotoxicity; genotoxicity; fetal malformations	7
		Lonidamine derivatives	Gamendazole Adjudin	Rat, Mouse, Monkey, Dog, Rabbit	25-100 mg/kg	Anti-cancer	Sloughing germ cells	Testis atrophy; azoospermia	Disruption of Sertoli cell junctions; occlusion of efferent ducts;	No	Yes	Reduced motor activity, palprebral ptosis, lacrimation, tremors and dyspnea	8
Epididymis	Oral, s.c.	3-Chloro-1,2-propanediol	α-Chlorohydrin Epibloc®	Rat, Mouse, Hamster, Dog, Guinea-pig, Sheep, Boar, Monkey	10-140 mg/kg	Organic chemical food contaminant	Immobilization of sperm	Testis atrophy; necrosis; azoospermia	Occlusion of efferent ducts; formation spermatoceles; inhibits epididymal function; inhibits glycolysis	No	Yes	High doses are neurotoxic and nephrotoxic	9
Testis	Intra-testicular[4]	Zinc compounds	Kastrin, Neutersol™, Zeuterin™, EsterilSol™, Testoblock®, Infertile®	Rat, Dog, Cat, Monkey, Bear, Pig	2.6-58 mg/ml	Dietary supplement; treatment of common cold	Multinucleated giant cells; acute inflammation; edema; testis swelling	Seminiferous tubule necrosis; atrophy;	Sertoli cell barrier damage; massive neutrophilic inflammation; necrosis; tubular fibrosis	Yes	Yes	Scrotal ulceration and dermatitis; necrotizing reactions	10
		4-allyl-2-methoxyphenol	Eugenol Clove oil	Dog	1 ml	Cosmetics; anti-anthelmintic; expectorant	Testis swelling	Seminiferous tubular fibrosis	Testicular necrosis; inflammation	Yes	Yes	Epididymal vacuolation	11

		$CaCl_2$ +/- ethanol & hypertonic NaCl	Calchlorin™, Salts	Rat, Dog, Cat, Bull, Goat, Donkey, Ram, Guinea pig, Buffalo	20-50% in water or ethanol	Inorganic salt; food additive; deicing salt; desiccant	Testis swelling; inflammation	Testicular atrophy	Testicular dehydration; necrosis; coagulative fibrosis	Yes	Yes	Scrotal necrosis if leakage	12
		$CdCl_2$	Metallic salt	Rat, Mouse, Dog, Rabbit, Hamster	150 μ/kg	Inorganic salt pigment	Hemorrhage; edema; inflammation	Testicular atrophy	Decreased blood flow; ischemia; oxidative stress	Yes	Yes	Toxic to numerous organs, if given oral	13
		1,2,3-trlhydroxypropane	Glycerol	Rat, Dog, Cat, Monkey, Rabbit	10-70%	Food additive; soap; laxative	Seminiferous tubule disruption	Testicular atrophy	Hyperosmotic dehydration	Yes	Yes	None	14
		Lactic acid	Chem-Cast®	Rat, Dog, Bull	85-92%	Organic synthesis; food additive	Germ cell degeneration	Testicular atrophy	Sclerosing, caustic chemical; necrosis; fibrosis	Yes	Yes	Scrotal necrosis if leakage	15
		$KMnO_4$	N/A	Pig	0.25 g/17 ml acetic acid	Inorganic oxidizing agent; water treatment	N/A	Seminiferous tubular atrophy; fibrosis efferent ducts	Sclerosing, caustic chemical	N/A	Yes	Leydig cell hypertrophy	16
		Metallic & rare earth salts	N/A	Rat, Mouse, Monkey	0.02-0.08 mM/kg	Salts	Focal necrosis	Testicular total necrosis for some salts	Cytolysis, ischemia, necrosis	N/A	Yes	Some Leydig cell loss; early effects on vas deferens sperm; some salts showed no effect	17
		Ethanol	Alcohol	Bull	100%; 10 ml	Organic solvent	Testis swelling; necrosis	Testicular atrophy	Necrosis; inflammation; edema; fibrosis	Yes	Yes	Only 50% became infertile	18
	Intra-Testicular; s.c or i.m	Inflammatory induction agents[5]	N/A	Guinea pig, Monkey, Rat, Rabbit; Dog; Ram	N/A	Antibody stimulating	Inflammation; seminiferous tubular dilation; testis swelling	Granuloma; necrosis; testicular atrophy	Inflammation;	No	Yes	Injection outside testis required for azoospermia for some agents	19
Epididymis Vas deferens	Intra-lumen	Sclerosing agents[2]	N/A	Ram, Goat, Dog, Cat, Rat, Mouse, Monkey	Wide range	Numerous chemicals	Inflammation; epididymis swollen	Granulomas and cysts; tissue scarring; azoospermia	Spermatic granuloma; fibrosis; blockage	No	Yes	Adhesions between epididymis and tunica vaginalis; sometimes testicular effects	20
	Intra-lumen plugs	Silicone, polyurethane elastomers, hydrogels, co-polymers	RISUG® ADAM™ Vasalgel®	Rat, Rabbit, Monkey, Man	N/A	Elastomers; gels	Occlusion of vas deferens	Azoospermia; testis and epididymal pathology	Prevention of sperm transport; blockage	No	Yes	Testis focal degeneration; hematoma	21

[1] Administration of the chemical/drug; oral, subcutaneous or cutaneous (s.c.), intramuscular (i.m.), intra-peritoneal or intra-organ (intra-testicular, intra-epididymal, intra-vas deferens); T, testosterone

[2] Sclerosing agents consist of a broad range of caustic chemicals that induce tissue damage and elicit fibrosis: Quinacrine dihydrochloride, zinc arginate, sodium tetradecyl sulfate, potassium permanganate, ethanol +/-ascorbic acid, silver nitrate, formaldehyde, styrene maleic anhydride, chlorhexidine digluconate, lactic acid, methylcyanoacrylate, urea and ethanol, formaldehyde and ethanol, $CaCl_2$ in ethanol

[3] N/A, not applicable

[4] Some of these are considered to be sclerosing agents and have been tested as intra-epididymal sterilants.

[5] Testicular antigens +/- killed bacteria; killed mycobacteria; Freund's complete adjuvant injected into foot after intratesticular injection of turpentine; Freund's complete adjuvant plus testis homogenate

[6] Supplemental References

1: Ketoconazole [1-18]

2: Embelin [19-25]

3: Dibromochloropropane [26-33]

4: 4-Vinylcyclohexene diepoxide [34-46]

5: 20,25-diazacholesterol dihydrochloride [47-62]
6: Quinestrol and Levonorgestrel [63, 64]
7: Methyl 1-(butylcarbamoyl)-2-benzimidazole-carbamate [65-88]
8: Lonidamine [11, 89-113]
9: 3-Chloro-1,2-propanediol [81, 114-173]
10: Zinc compounds [174-215]
11: 4-allyl-2-methoxyphenol [216-221]
12: $CaCl_2$ +/- ethanol and hypertonic $NaCl_2$ [42, 220, 222-257]fib
13: $CdCl_2$ [258-283]
14: 1,2,3-trlhydroxypropane [284-292]
15: Lactic Acid [293-296]
16: Potassium permanganate [297, 298]
17: Metallic and rare earth salts [299, 300]
18: Ethanol [238]
19: Inflammatory induction agents [209, 301-307]
20: Sclerosing Agents [233, 298, 308-316, 317l, 318-321]
21: Intra-lumen plugs [322-328]

1. Heeres, J., Backx, L.J., Mostmans, J.H., and Van Cutsem, J., Antimycotic imidazoles. part 4. Synthesis and antifungal activity of ketoconazole, a new potent orally active broad-spectrum antifungal agent. J Med Chem, 1979. 22(8): p. 1003-5.
2. Pont, A., Williams, P.L., Azhar, S., Reitz, R.E., Bochra, C., Smith, E.R., and Stevens, D.A., Ketoconazole blocks testosterone synthesis. Arch Intern Med, 1982. 142(12): p. 2137-40.
3. Borgers, M., Van den Bossche, H., and De Brabander, M., The mechanism of action of the new antimycotic ketoconazole. The American Journal of Medicine, 1983. 74(1, Part 2): p. 2-8.
4. Dismukes, W.E., Stamm, A.M., Graybill, J.R., Craven, P.C., Stevens, D.A., Stiller, R.L., Sarosi, G.A., Medoff, G., Gregg, C.R., Gallis, H.A., Fields, B.T., Jr., Marier, R.L., Kerkering, T.A., Kaplowitz, L.G., Cloud, G., Bowles, C., and Shadomy, S., Treatment of systemic mycoses with ketoconazole: emphasis on toxicity and clinical response in 52 patients. National Institute of Allergy and Infectious Diseases collaborative antifungal study. Ann Intern Med, 1983. 98(1): p. 13-20.
5. Pont, A., Graybill, J.R., Craven, P.C., Galgiani, J.N., Dismukes, W.E., Reitz, R.E., and Stevens, D.A., High-dose ketoconazole therapy and adrenal and testicular function in humans. Arch Intern Med, 1984. 144(11): p. 2150-3.
6. Schürmeyer, T. and Nieschlag, E., Effect of ketoconazole and other imidazole fungicides on testosterone biosynthesis. Acta Endocrinol (Copenh), 1984. 105(2): p. 275-80.
7. Vickery, B.H., Burns, J., Zaneveld, L.J., Goodpasture, J.C., and Bergstrom, K., Orally administered ketoconazole rapidly appears in seminal plasma and suppresses sperm motility. Adv Contracept, 1985. 1(4): p. 341-53.
8. Willard, M.D., Nachreiner, R., McDonald, R., and Roudebush, P., Ketoconazole-induced changes in selected canine hormone concentrations. Am J Vet Res, 1986. 47(12): p. 2504-9.
9. Willard, M.D., Nachreiner, R.F., Howard, V.C., and Fooshee, S.K., Effect of long-term administration of ketoconazole in cats. Am J Vet Res, 1986. 47(12): p. 2510-3.
10. Sonino, N., The use of ketoconazole as an inhibitor of steroid production. N Engl J Med, 1987. 317(13): p. 812-8.
11. Zaneveld, L.J. and Waller, D.P., Nonhormonal mediation of male reproductive tract damage: data from contraceptive drug research. Prog Clin Biol Res, 1989. 302: p. 129-49; discussion 150-6.
12. Waller, D.P., Martin, A., Vickery, B.H., and Zaneveld, L.J.D., The effect of ketoconazole on fertility of male rats. Contraception, 1990. 41(4): p. 411-417.
13. Joshi, S.C., Jain, G.C., and Lata, M., Effects of ketoconazole (an imidazole antifungal agent) on the fertility and reproductive function of male mice. Acta Eur Fertil, 1994. 25(1): p. 55-8.
14. Adams, M.L., Meyer, E.R., and Cicero, T.J., Imidazoles suppress rat testosterone secretion and testicular interstitial fluid formation In vivo. Biol Reprod, 1998. 59(2): p. 248-54.
15. Amin, A., Ketoconazole-induced testicular damage in rats reduced by Gentiana extract. Experimental and Toxicologic Pathology, 2008. 59(6): p. 377-384.

16. Blode, H., Zeun, S., Parke, S., Zimmermann, T., Rohde, B., Mellinger, U., and Kunz, M., Evaluation of the effects of rifampicin, ketoconazole and erythromycin on the steady-state pharmacokinetics of the components of a novel oral contraceptive containing estradiol valerate and dienogest in healthy postmenopausal women. Contraception, 2012. 86(4): p. 337-44.
17. Katiraee, F., Kouchak Kosari, Y., Soltani, M., Shokri, H., and Hassan Minooieanhaghighi, M., Molecular Identification and Antifungal Susceptibility Patterns of Dermatophytes Isolated from Companion Animals with Clinical Symptoms of Dermatophytosis. J Vet Res, 2021. 65(2): p. 175-182.
18. Tritos, N.A., Adrenally Directed Medical Therapies for Cushing Syndrome. J Clin Endocrinol Metab, 2021. 106(1): p. 16-25.
19. Chitra, M., Sukumar, E., Suja, V., and Devi, C.S., Antitumor, anti-inflammatory and analgesic property of embelin, a plant product. Chemotherapy, 1994. 40(2): p. 109-13.
20. Kalyan Kumar, G., Dhamotharan, R., Kulkarni, N.M., Mahat, M.Y., Gunasekaran, J., and Ashfaque, M., Embelin reduces cutaneous TNF-alpha level and ameliorates skin edema in acute and chronic model of skin inflammation in mice. Eur J Pharmacol, 2011. 662(1-3): p. 63-9.
21. Ahn, K.S., Sethi, G., and Aggarwal, B.B., Embelin, an inhibitor of X chromosome-linked inhibitor-of-apoptosis protein, blocks nuclear factor-kappaB (NF-kappaB) signaling pathway leading to suppression of NF-kappaB-regulated antiapoptotic and metastatic gene products. Mol Pharmacol, 2007. 71(1): p. 209-19.
22. Agrawal, S., Chauhan, S., and Mathur, R., Antifertility Effects of Embelin in Male Rats. Andrologia, 1986. 18(2): p. 125-131.
23. Dixit, V.P. and Bhargava, S.K., Reversible Contraception like Activity of Embelin in Male Dogs (Canis indicus Linn). Andrologia, 1983. 15(5): p. 486-494.
24. Gupta, S., Sanyal, S.N., and Kanwar, U., Antispermatogenic effect of embelin, a plant benzoquinone, on male albino rats in vivo and in vitro. Contraception, 1989. 39(3): p. 307-20.
25. Githui, E.K., Makawiti, D.W., and Midiwo, J.O., Changes in the concentrations of testosterone, luteinising hormone and progesterone associated with administration of embelin. Contraception, 1991. 44(3): p. 311-7.
26. Whorton, M.D. and Foliart, D.E., Mutagenicity, carcinogenicity and reproductive effects of dibromochloropropane (DBCP). Mutat Res, 1983. 123(1): p. 13-30.
27. Greenwell, A., Tomaszewski, K.E., and Melnick, R.L., A biochemical basis for 1,2-dibromo-3-chloropropane-induced male infertility: inhibition of sperm mitochondrial electron transport activity. Toxicol Appl Pharmacol, 1987. 91(2): p. 274-80.
28. Kluwe, W.M., Lamb, J.C.t., Greenwell, A.E., and Harrington, F.W., 1,2-dibromo-3-chloropropane (DBCP)-induced infertility in male rats mediated by a post-testicular effect. Toxicol Appl Pharmacol, 1983. 71(2): p. 294-8.
29. Sod-Moriah, U.A., Sror, U., Shemi, D., Potashnik, G., Chayoth, R., Shaked, I., and Kaplanski, J., Long term effects of dibromochloropropane (DBCP) on male rats' reproductive system. Andrologia, 1988. 20(1): p. 60-6.
30. Glass, R.I., Lyness, R.N., Mengle, D.C., Powell, K.E., and Kahn, E., Sperm count depression in pesticide applicators exposed to dibromochloropropane. Am J Epidemiol, 1979. 109(3): p. 346-51.
31. Whorton, M.D., Male occupational reproductive hazards. West J Med, 1982. 137(6): p. 521-4.
32. Biava, C.G., Smuckler, E.A., and Whorton, D., The testicular morphology of individuals exposed to dibromochloropropane. Exp Mol Pathol, 1978. 29(3): p. 448-58.
33. Yoshida, S., Yamada, H., Sugawara, I., and Takeda, K., Effect of Dibromochloropropane (DBCP) on the Hormone Receptors of the Male Rat Reproductive System. Bioscience, Biotechnology, and Biochemistry, 1998. 62(3): p. 479-483.
34. Chhabra, R.S., Elwell, M.R., and Peters, A., Toxicity of 4-vinyl-1-cyclohexene diepoxide after 13 weeks of dermal or oral exposure in rats and mice. Fundam Appl Toxicol, 1990. 14(4): p. 745-51.
35. Dhillon, S. and Von Burg, R., Vinylcyclohexene dioxide. J Appl Toxicol, 1996. 16(5): p. 465-8.
36. Adedara, I.A., Abolaji, A.O., Ladipo, E.O., Fatunmibi, O.J., Abajingin, A.O., and Farombi, E.O., 4-Vinylcyclohexene diepoxide disrupts sperm characteristics, endocrine balance and redox status in testes and epididymis of rats. Redox Rep, 2017. 22(6): p. 388-398.
37. Hooser, S.B., DeMerell, D.G., Douds, D.A., Hoyer, P., and Sipes, I.G., Testicular germ cell toxicity caused by vinylcyclohexene diepoxide in mice. Reprod Toxicol, 1995. 9(4): p. 359-67.
38. Hoyer, P.B., Devine, P.J., Hu, X., Thompson, K.E., and Sipes, I.G., Ovarian toxicity of 4-vinylcyclohexene diepoxide: a mechanistic model. Toxicol Pathol, 2001. 29(1): p. 91-9.
39. Bhattacharya, P. and Keating, A.F., Ovarian metabolism of xenobiotics. Exp Biol Med (Maywood), 2011. 236(7): p. 765-71.
40. Kappeler, C.J. and Hoyer, P.B., 4-vinylcyclohexene diepoxide: a model chemical for ovotoxicity. Syst Biol Reprod Med, 2012. 58(1): p. 57-62.
41. Hinds, L.A., Henry, S., Sharma, S., Leung, L., Dyer, C., and Mayer, L., Effects of Oral Uptake of the Chemosterilant 4-Vinylcyclohexene Diepoxide in Wild House Mice, Mus domesticus. Proceedings of the Vertebrate Pest Conference, 2014. 26: p. 380-385.
42. Sen, C.C., Yumusak, N., Faundez, R., Temamogullari, F., and Taskin, A., Evaluation of intra-testicular injections of calcium chloride and 4-vinylcyclohexene 1,2 monoepoxide for chemical sterilization in guinea pigs. Pol J Vet Sci, 2017. 20(2): p. 251-260.

43. Paksoy, Z., Kandemir, F., Gökhan, N., and Ozkaraca, M., The effects of 4-vinylcyclohexene diepoxide on the testes of dogs. Veterinarski Arhiv, 2018. 88: p. 807-822.
44. Abolaji, A.O., Omozokpia, M.U., Oluwamuyide, O.J., Akintola, T.E., and Farombi, E.O., Rescue role of hesperidin in 4-vinylcyclohexene diepoxide-induced toxicity in the brain, ovary and uterus of wistar rats. J Basic Clin Physiol Pharmacol, 2020. 31(2).
45. Witmer, G.W., Raymond-Whish, S., Moulton, R.S., Pyzyna, B.R., Calloway, E.M., Dyer, C.A., Mayer, L.P., and Hoyer, P.B., Compromised Fertility in Free Feeding of Wildcaught Norway Rats (Rattus norvegicus) with a Liquid Bait Containing 4-Vinylcyclohexene Diepoxide and Triptolide. J Zoo Wildl Med, 2017. 48(1): p. 80-90.
46. Witmer, G.W. and Raymond-Whish, S., Reduced fecundity in free-ranging Norway rats after baiting with a liquid fertility control bait. Human-Wildlife Interactions, 2021. 15(1): p. 111-123-123.
47. Fagerstone, K.A., Miller, L.A., Killian, G., and Yoder, C.A., Review of issues concerning the use of reproductive inhibitors, with particular emphasis on resolving human-wildlife conflicts in North America. Integrative Zoology, 2010. 5(1): p. 15-30.
48. Yoder, C., Andelt, W., Miller, L.A., Johnston, J., and Goodall, M., Effectiveness of Twenty, Twenty-Five Diazacholesterol, Avian Gonadotropin-Releasing Hormone, and Chicken Riboflavin Carrier Protein for Inhibiting Reproduction in Coturnix Quail. Poultry science, 2004. 83: p. 234-44.
49. Counsell, R.E., Lu, M.C., el-Masry, S., and Weinhold, P.A., Inhibition of cholesterol side-chain cleavage by azacholesterols. Biochem Pharmacol, 1971. 20(10): p. 2912-5.
50. Ranney, R.E. and Cook, D.L., THE HYPOCHOLESTEROLEMIC ACTION OF 20,25-DIAZACHOLESTEROL. Arch Int Pharmacodyn Ther, 1965. 154: p. 51-62.
51. Lofts, B., Murton, R.K., and Thearle, R.J., The effects of 22,25-diazacholesterol dihydrochloride on the pigeon testis and on reproductive behaviour. J Reprod Fertil, 1968. 15(1): p. 145-8.
52. Yoder, C.A., Bynum, K., and Miller, L. Development of diazacon™ as an avian contraceptive. in 11th Wildlife Damage Management Conference. 2005. Traverse City, Michigan. USA.
53. Sinha Hikim, A.P. and Chakraborty, J., Effects of diazacholesterol dihydrochloride (SC-12937), an avian antifertility agent, on rat testis. J Androl, 1986. 7(5): p. 277-84.
54. Singh, S.K. and Chakravarty, S., Antispermatogenic and antifertility effects of 20,25-diazacholesterol dihydrochloride in mice. Reprod Toxicol, 2003. 17(1): p. 37-44.
55. Nash, P., Furcolow, C.A., Bynum, K.S., Yoder, C.A., Miller, L.A., and Johnston, J.J., 20,25-Diazacholesterol as an oral contraceptive for black-tailed prairie dog population management. Human–Wildlife Conflicts, 2007. 1(1): p. 60-67.
56. Avery, M.L., Yoder, C.A., and Tillman, E.A., Diazacon inhibits reproduction in invasive monk parakeet populations. The Journal of Wildlife Management, 2008. 72(6): p. 1449-1452.
57. Mayle, B., Feryman, M., Peace, A., Yoder, C.A., Miler, L.A., and Cowan, D., The use of DiazaCon(™) to limit fertility in grey squirels, in 8th European Vertebrate Pest Management Conference. 2011, Julius-Kühn-Archiv: Berlin, Germany,. p. 120-121.
58. Yoder, C.A., Mayle, B.A., Furcolow, C.A., Cowan, D.P., and Fagerstone, K.A., Feeding of grey squirrels (Sciurus carolinensis) with the contraceptive agent DiazaCon™: effect on cholesterol, hematology, and blood chemistry. Integr Zool, 2011. 6(4): p. 409-19.
59. Mayle, B.A., Ferryman, M., Peace, A., Yoder, C.A., Miller, L., and Cowan, D., The use of DiazaCon™ to limit fertility by reducing serum cholesterol in female grey squirrels, Sciurus carolinensis. Pest Manag Sci, 2013. 69(3): p. 414-24.
60. Yoder, C.A., Mauldin, R.E., Gionfriddo, J.P., Crane, K.A., Goldade, D.A., and Engeman, R.M., DiazaCon reduces black-tailed prairie dog reproduction in Colorado. Wildlife Research, 2016. 43(8): p. 655-661.
61. Asa, C. and Moresco, A., Fertility Control in Wildlife: Review of Current Status, Including Novel and Future Technologies. Advances in Experimental Medicine and Biology, 2019. 1200: p. 507-543.
62. Humphrys, S. and Lapidge, S., Delivering and registering species-tailored oral antifertility products: a review. Wildlife Research - WILDLIFE RES, 2008. 35.
63. Liu, M., Qu, J., Yang, M., Wang, Z., Wang, Y., Zhang, Y., and Zhang, Z., Effects of quinestrol and levonorgestrel on populations of plateau pikas, Ochotona curzoniae, in the Qinghai-Tibetan Plateau. Pest Manag Sci, 2012. 68(4): p. 592-601.
64. Liu, M., Wan, X., Yin, Y., Li, Y.X., Sun, F., Zhang, Z., and Wang, Y.L., Subfertile effects of quinestrol and levonorgestrel in male rats. Reprod Fertil Dev, 2012. 24(2): p. 297-308.
65. Davidse, L.C. and Flach, W., Differential binding of methyl benzimidazol-2-yl carbamate to fungal tubulin as amechanism of resistance to this antimitotic agent in mutant strains of Aspergillus nidulans. J Cell Biol, 1977. 72: p. 174-193.
66. Carter, S.D. and Laskey, J.W., Effect of benomyl on reproduction in the male rat. Toxicol Lett, 1982. 11(1-2): p. 87-94.
67. Barnes, T., Verlangieri, A., and Wilson, M., Reproductive toxicity of methyl-1-butylcarbamoyl-2-benzimidazole carbamate benomyl in male Wistar rats. Toxicology, 1983. 28: p. 103-115.
68. Carter, S.D., Hein, J.F., Rehnberg, G.L., and Laskey, J.W., Effect of benomyl on the reproductive development of male rats. J Toxicol Environ Health, 1984. 13(1): p. 53-68.
69. Carter, S.D., Hess, R.A., and Laskey, J.W., The fungicide methyl 2-benzimidazole carbamate causes infertility in male Sprague-Dawley rats. Biol Reprod, 1987. 37(3): p. 709-17.
70. Lim, J. and Miller, M.G., The role of the benomyl metabolite carbendazim in benomyl-induced testicular toxicity. Toxicol Appl Pharmacol, 1997. 142(2): p. 401-10.

71. Hess, R.A., Moore, B.J., Forrer, J., Linder, R.E., and Abuel-Atta, A.A., The fungicide benomyl (methyl 1-(butylcarbamoyl)-2- benzimidazolecarbamate) causes testicular dysfunction by inducing the sloughing of germ cells and occlusion of efferent ductules. Fund Appl Toxicol, 1991. 17(4): p. 733-45.
72. Nakai, M. and Hess, R.A., Morphological changes in the rat Sertoli cell induced by the microtubule poison carbendazim. Tissue Cell, 1994. 26(6): p. 917-27.
73. Nakai, M., Hess, R.A., Matsuo, F., Gotoh, Y., and Nasu, T., Further observations on carbendazim-induced abnormalities of spermatid morphology in rats. Tissue Cell, 1997. 29(4): p. 477-85.
74. Correa, L.M., Nakai, M., Strandgaard, C.S., Hess, R.A., and Miller, M.G., Microtubules of the mouse testis exhibit differential sensitivity to the microtubule disruptors Carbendazim and colchicine. Toxicol Sci, 2002. 69(1): p. 175-82.
75. Nakai, M., Miller, M.G., Carnes, K., and Hess, R.A., Stage-specific effects of the fungicide carbendazim on Sertoli cell microtubules in rat testis. Tissue Cell, 2002. 34(2): p. 73-80.
76. Nakai, M. and Hess, R.A., Effects of carbendazim (methyl 2-benzimidazole carbamate; MBC) on meiotic spermatocytes and subsequent spermiogenesis in the rat testis. Anat Rec, 1997. 247(3): p. 379-87.
77. Nakai, M., Hess, R.A., Netsu, J., and Nasu, T., Deformation of the rat Sertoli cell by oral administration of carbendazim (methyl 2-benzimidazole carbamate). J Androl, 1995. 16(5): p. 410-6.
78. Nakai, M., Hess, R.A., Moore, B.J., Guttroff, R.F., Strader, L.F., and Linder, R.E., Acute and long-term effects of a single dose of the fungicide carbendazim (methyl 2-benzimidazole carbamate) on the male reproductive system in the rat. J Androl, 1992. 13(6): p. 507-18.
79. Nakai, M., Moore, B.J., and Hess, R.A., Epithelial reorganization and irregular growth following carbendazim-induced injury of the efferent ductules of the rat testis. Anat Rec, 1993. 235(1): p. 51-60.
80. Hess, R.A., Effects of environmental toxicants on the efferent ducts, epididymis and fertility. J Reprod Fertil Suppl, 1998. 53: p. 247-59.
81. Hess, R.A., Efferent ductules: Structure and function, in Encyclopedia of Reproduction (Second Edition), Skinner, M.K., Editor. 2018, Academic Press: Oxford. p. 270-278.
82. Hess, R.A., Sharpe, R.M., and Hinton, B.T., Estrogens and development of the rete testis, efferent ductules, epididymis and vas deferens. Differentiation, 2021: p. (in press).
83. Hess, R.A. and Nakai, M., Histopathology of the male reproductive system induced by the fungicide benomyl. Histol Histopathol, 2000. 15(1): p. 207-224.
84. Carlisle, J.C. and Cochran, R.C., Benomyl: Risk Characterization Document, Regulation, D.o.P., Editor. 1999, California Environmental Protection Agency. p. 252.
85. Kavlock, R., Chernoff, N., Gray Jr, L., Gray, J., and Whitehouse, D., Teratogenic effects of benomyl in the Wistar rat and CD-1 mouse, with emphasis on the route of administration. Toxicol Appl Pharmacol, 1982. 62: p. 44-54.
86. Cummings, A.M., Harris, S.T., and Rehnberg, G.L., Effects of methyl benzimidazolecarbamate during early pregnancy in the rat. Fundamental and Applied Toxicology, 1990. 15(3): p. 528-535.
87. Ellis, W., Roos, D., , F., Kavlock, R., and Zeman, F., Relationship of periventricular overgrowth to hydrocephalus in brains of fetal rats exposed to benomyl. Teratogen Carcinogen Mutagen, 1988. 8: p. 377-391.
88. Rogers, J.M., Setzer, R.W., Branch, S., and Chernoff, N., Chemically induced supernumerary lumbar ribs in CD-1 mice: size distribution and dose response. Birth Defects Res Part B Dev Reprod Toxicol, 2004. 71(1): p. 17-25.
89. Carter, S.K., Current protocol approaches in large bowel cancer. Semin Oncol, 1976. 3(4): p. 433-43.
90. Cioli, V., Bellocci, B., Putzolu, S., Malorni, W., and Demartino, C., Anti-spermogenic activity of lonidamine (AF-1890) in rabbit. Ultramicroscopy, 1980. 5: p. 418-423.
91. Nath, K., Guo, L., Nancolas, B., Nelson, D.S., Shestov, A.A., Lee, S.-C., Roman, J., Zhou, R., Leeper, D.B., Halestrap, A.P., Blair, I.A., and Glickson, J.D., Mechanism of antineoplastic activity of lonidamine. Biochimica et biophysica acta, 2016. 1866(2): p. 151-162.
92. Heywood, R., James, R.W., Barcellona, P.S., Campana, A., and Cioli, V., Toxicological studies on 1-substituted-indazole-3-carboxylic acids. Chemotherapy, 1981. 27 Suppl 2: p. 91-7.
93. James, R.W., A toxicological study of some chemical actions on the testes of laboratory rats and beagle dogs, in Department of Pharmacy & Pharmacology. 1980, University of Bath: Bath, UK.
94. Cheng, C.Y., Mo, M., Grima, J., Saso, L., Tita, B., Mruk, D., and Silvestrini, B., Indazole carboxylic acids in male contraception. Contraception, 2002. 65(4): p. 265-8.
95. Maranghi, F., Mantovani, A., Macri, C., Romeo, A., Eleuteri, P., Leter, G., Rescia, M., Spano, M., and Saso, L., Long-term effects of lonidamine on mouse testes. Contraception, 2005. 72(4): p. 268-72.
96. Traina, M.E., Guarino, M., Urbani, E., Saso, L., Eleuteri, P., Cordelli, E., Rescia, M., Leter, G., and Spano, M., Lonidamine transiently affects spermatogenesis in pubertal CD1 mice. Contraception, 2005. 72(4): p. 262-7.
97. Mruk, D.D., Silvestrini, B., and Yan Cheng, C., Anchoring Junctions As Drug Targets: Role in Contraceptive Development. Pharmacol Rev, 2008.
98. Tash, J.S., Attardi, B., Hild, S.A., Chakrasali, R., Jakkaraj, S.R., and Georg, G.I., A novel potent indazole carboxylic acid derivative blocks spermatogenesis and is contraceptive in rats after a single oral dose. Biol Reprod, 2008. 78(6): p. 1127-38.

99. De Martino, C., Malcorni, W., Bellocci, M., Floridi, A., and Marcante, M.L., Effects of AF 1312 TS and lonidamine on mammalian testis. A morphological study. Chemotherapy, 1981. 27 Suppl 2: p. 27-42.
100. Georg, I.G., Tash, J.S., Chakrasali, R., Jakkaraj, S.R., and Roby, K., Lonidamine Analogues for Fertility Management, States, U., Editor. 2013, University of Kansas, Lawrence, KS: U.S. p. 1-44.
101. Xie, Q.R., Liu, Y., Shao, J., Yang, J., Liu, T., Zhang, T., Wang, B., Mruk, D.D., Silvestrini, B., Cheng, C.Y., and Xia, W., Male contraceptive Adjudin is a potential anti-cancer drug. Biochem Pharmacol, 2013. 85(3): p. 345-55.
102. Wong, E.W.P., Yan, H.H.N., Li, M.W.M., Lie, P.P.Y., Mruk, D.D., and Cheng, C.Y., Cell Junctions in the Testis as Targets for Toxicants, in Comprehensive Toxicology, McQueen, C.A., Editor. 2018. p. 167–188.
103. Marcante, M.L., Natali, P.G., Floridi, A., and De Martino, C., Effects of AF 1312/TS and lonidamine on cultured sertoli cells. Chemotherapy, 1981. 27 Suppl 2: p. 43-9.
104. Galdieri, M., Monaco, L., and De Martino, C., Morphological and biochemical effects of lonidamine on cultured Sertoli cells. Exp Mol Pathol, 1984. 41(2): p. 202-6.
105. Grima, J., Zhu, L., and Cheng, C.Y., Testin is tightly associated with testicular cell membrane upon its secretion by sertoli cells whose steady-state mRNA level in the testis correlates with the turnover and integrity of inter-testicular cell junctions. J Biol Chem, 1997. 272(10): p. 6499-509.
106. Gatto, M.T., Tita, B., Artico, M., and Saso, L., Recent studies on lonidamine, the lead compound of the antispermatogenic indazol-carboxylic acids. Contraception, 2002. 65(4): p. 277-8.
107. Su, L., Cheng, C.Y., and Mruk, D.D., Adjudin-mediated Sertoli-germ cell junction disassembly affects, Sertoli cell barrier function in vitro and in vivo. Int J Biochem Cell Biol, 2010.
108. Mruk, D.D., Bonanomi, M., and Silvestrini, B., Lonidamine-ethyl ester-mediated remodelling of the Sertoli cell cytoskeleton induces phosphorylation of plakoglobin and promotes its interaction with alpha-catenin at the blood-testis barrier. Reprod Fertil Dev, 2017. 29(5): p. 998-1011.
109. Cheung, K.H., Leung, C.T., Leung, G.P., and Wong, P.Y., Synergistic effects of cystic fibrosis transmembrane conductance regulator and aquaporin-9 in the rat epididymis. Biol Reprod, 2003. 68(5): p. 1505-10.
110. Gong, X.D., Linsdell, P., Cheung, K.H., Leung, G.P., and Wong, P.Y., Indazole inhibition of cystic fibrosis transmembrane conductance regulator Cl(-) channels in rat epididymal epithelial cells. Biol Reprod, 2002. 67(6): p. 1888-96.
111. Malorni, W., Meschini, S., Matarrese, P., and Arancia, G., The cytoskeleton as a subcellular target of the antineoplastic drug lonidamine. Anticancer Res, 1992. 12(6b): p. 2037-45.
112. El Khouri, E., Whitfield, M., Stouvenel, L., Kini, A., Riederer, B., Lores, P., Roemermann, D., di Stefano, G., Drevet, J.R., Saez, F., Seidler, U., and Toure, A., Slc26a3 deficiency is associated with epididymis dysplasia and impaired sperm fertilization potential in the mouse. Mol Reprod Dev, 2018. 85(8-9): p. 682-695.
113. Wang, Y.Y., Lin, Y.H., Wu, Y.N., Chen, Y.L., Lin, Y.C., Cheng, C.Y., and Chiang, H.S., Loss of SLC9A3 decrease CFTR protein and causes obstructed azoospermia in mice. PLoS Genet, 2017. 13(4): p. e1006715.
114. PubChem, 3-Chloro-1,2-propanediol, Information, N.C.f.B., Editor. 2021, National Library of Medicine (US): Bethesda (MD).
115. Ericsson, R.J. and Youngdale, G.A., Male antifertility compounds: structure and activity relationships of U-5897, U-l5,646 and related substances. J Reprod Fertil, 1970. 21(2): p. 263-6.
116. Ericsson, R.J. and Baker, V.F., Male antifertility compounds: biological properties of U-5897 and U-15646. J Reprod Fert, 1970. 21: p. 267-273.
117. Brown-Woodman, P.D.C. and White, I.G., Effect of α-chlorohydrin on cauda epididymis and spermatozoa of the rat and general physiological status. Contraception, 1975. 11(1): p. 69-78.
118. Back, D.J., Glover, T.D., Shenton, J.C., and Boyd, G.P., The effects of a-chlorohydrin on the composition of rat and rabbit epididymal plasma: a possible explanation of species difference. J Reprod Fert, 1975. 45: p. 117-128.
119. Crabo, B.G., Zimmerman, K.J., Gustafsson, B., Holtman, M., Koh, T.J.P., and Graham, E.F., Effect of low doses of a-chlorohydrin on fertility and semen characteristics and binding of the drug to spermatozoa in swine. Int J Fertil, 1975. 20: p. 87-94.
120. Mohri, H.D., Sutter, D.A.I., Brown-Woodman, P.D.C., White, G., and Ridley, D., Identification of the biochemical lesion produced by a-chlorohydrin in spermatozoa. Nature, 1975. 255: p. 75-76.
121. Brown-Woodman, P.D.C. and White, J.G., Disruption of the metabolism motility and morphology of spermatozoa by injection of a-chlorohydrin into rams. Aust J Biol Sci, 1976. 29: p. 545-555.
122. Paz, G.F. and Homonnai, T.Z., A direct effect of alpha-chlorohydrin on rat epididymal spermatozoa. Int J Androl, 1982. 5(3): p. 308-16.
123. Jones, A.R. and Porter, L.M., Inhibition of glycolysis in boar spermatozoa by alpha-chlorohydrin phosphate appears to be mediated by phosphatase activity. Reprod Fertil Dev, 1995. 7(5): p. 1089-94.
124. Jelks, K.B. and Miller, M.G., alpha-Chlorohydrin inhibits glyceraldehyde-3-phosphate dehydrogenase in multiple organs as well as in sperm. Toxicol Sci, 2001. 62(1): p. 115-23.
125. Wong, P.Y. and Yeung, C.H., Inhibition by alpha-chlorohydrin of fluid reabsorption in the rat cauda epididymidis. J Reprod Fertil, 1977. 51(2): p. 469-71.
126. Wong, P.Y.D., Effect of alpha-chlorohydrin on transport processes in perfused rat cauda epididymides. Contraception, 1977. 16(6): p. 637-644.

127. Hinton, B.T., Hernandez, H., and Howards, S.S., The male antifertility agents alpha chlorohydrin, 5-thio-D-glucose, and 6-chloro-6-deoxy-D-glucose interfere with sugar transport across the epithelium of the rat caput epididymidis. J Androl, 1983. 4(3): p. 216-21.
128. Ericsson, R.J., Male antifertility compounds: U-5897 as a rat chemosterilant. J Reprod Fert, 1970. 22: p. 213-222.
129. Samojlik, E. and Chang, M.C., Antifertility activity of 3-chloro-1, 2-propanediol (U-5897) on male rats. Biol Reprod, 1970. 2(2): p. 299-304.
130. Cooper, E.R., Jones, A.R., and Jackson, H., Effects of alpha-chlorohydrin and related compounds on the reproductive organs and fertility of the male rat. J Reprod Fertil, 1974. 38(2): p. 379-86.
131. Reijonen, K., Kormano, M., and Ericsson, R.J., Studies on the rat epididymal blood vessels following alpha-chlorohydrin administration. Biol Reprod, 1975. 12(4): p. 483-90.
132. Ericsson, R.J. Alpha-chlorohydrin (EPIBLOC): a toxicant-sterilant as an alternative in rodent control. in Vertebrate Pest Conference. 1982.
133. Sullivan, R. and Belleannée, C., Cell Biology of the Epididymis, in Encyclopedia of Reproduction, Skinner, M.K., Editor. 2018, Academic Press: Elsevier: Oxford. p. 286-291.
134. Sullivan, R., Legare, C., Lamontagne-Proulx, J., Breton, S., and Soulet, D., Revisiting structure/functions of the human epididymis. Andrology, 2019. 7(5): p. 748-757.
135. Ericsson, R.J. *Epibloc*. 2021 [cited 2021; Available from: https://www.epibloc.com/index.htm.
136. Haratake, J., Furuta, A., and Hashimoto, H., Immunohistochemical and ultrastructural study of hepatic sinusoidal linings during dichloropropanol-induced acute hepatic necrosis. Liver, 1994. 14(2): p. 90-7.
137. Andrews, R.V. and Belknap, R.W., Efficacy of alpha-chlorhydrin in sewer rat control. J Hyg (Lond), 1983. 91(2): p. 359-66.
138. Andrews, R.V., Belknap, R.W., and Keenan, E.J., Demographic and Endocrine Responses of Norway Rats to Antifertility Control. The Journal of Wildlife Management, 1974. 38(4): p. 868-874.
139. Bone, W. and Cooper, T.G., In vitro inhibition of rat cauda epididymal sperm glycolytic enzymes by ornidazole, alpha-chlorohydrin and 1-chloro-3-hydroxypropanone. Int J Androl, 2000. 23(5): p. 284-93.
140. Brown-Woodman, P.D. and White, I.G., Effect of alpha-chlorohydrin and vasoligation on epididymal and testicular blood flow in the rat and on sperm parameters. Acta Eur Fertil, 1978. 9(4): p. 189-99.
141. Caflisch, C.R. and DuBose, T.D., Jr., Effect of alpha-chlorohydrin on in situ pH in rat testis and epididymis. Contraception, 1990. 41(2): p. 207-12.
142. Chulavatnatol, M., Hasibuan, I., Yindepit, S., and Eksittikul, T., Lack of effect of alpha-chlorohydrin on the ATP content of rat, mouse and human spermatozoa. J Reprod Fertil, 1977. 50(1): p. 137-9.
143. Dixit, V.P. and Agrawal, M., Inhibition of spermatogenesis in house rat (Rattus rattus Rufescens) following the administration of alpha- chlorohydrin. Andrologia, 1980. 12(6): p. 513-20.
144. Ford, W.C., Harrison, A., and Waites, G.M., Effects of 6-chloro-6-deoxysugars on glucose oxidation in rat spermatozoa. J Reprod Fertil, 1981. 63(1): p. 67-73.
145. Ford, W.C. and Waites, G.M., Activities of various 6-chloro-6-deoxysugars and (S) alpha-chlorohydrin in producing spermatocoeles in rats and paralysis in mice and in inhibiting glucose metabolism in bull spermatozoa in vitro. J Reprod Fertil, 1982. 65(1): p. 177-83.
146. Gill, S.K. and Guraya, S.S., Effects of low doses of alpha-chlorohydrin on phosphatases, beta- glucosidase, beta-glucuronidase & hyaluronidase of rat testis & epididymis. Indian J Exp Biol, 1980. 18(11): p. 1351-2.
147. Gingell, R., Beatty, P.W., Mitschke, H.R., Mueller, R.L., Sawin, V.L., and Page, A.C., Evidence that epichlorohydrin is not a toxic metabolite of 1,2-dibromo- 3-chloropropane. Xenobiotica, 1987. 17(2): p. 229-40.
148. Helal, T.Y., Chemosterilant and rodenticidal effects of 3-chloro, 1,2 propanediol (EPIBLOC) against the albino laboratory rat and the Nile field rat. Int. Pest Control, 1982. 24: p. 20.
149. Hunt, D.M., Lubicz-Nawrocki, C.M., and Chang, M.C., The effects of 17 beta-estradiol and medroxyprogesterone acetate alone and in combination with alpha-chlorohydrin on reproductive function in the male rat. Biol Reprod, 1976. 14(5): p. 544-8.
150. Jones, A.R., Antifertility actions of alpha-chlorohydrin in the male. Aust J Biol Sci, 1983. 36(4): p. 333-50.
151. Jones, A.R. and Cooper, T.G., Metabolism of 36Cl-ornidazole after oral application to the male rat in relation to its antifertility activity. Xenobiotica, 1997. 27(7): p. 711-21.
152. Jones, A.R. and Dobbie, M.S., The male antifertility activity of 6-chloro-6-deoxyglucose. Contraception, 1991. 44(6): p. 649-55.
153. Jones, A.R., Fakhouri, G., and Gadiel, P., The metabolism of the soil fumigant 1,2-dibromo-3-chloropropane in the rat. Experientia, 1979. 35(11): p. 1432-4.
154. Jones, A.R., Gadiel, P., and Murcott, C., The renal toxicity of the rodenticide alpha-chlorohydrin in the rat. Naturwissenschaften, 1979. 66(8): p. 425.
155. Jones, A.R., Milton, D.H., and Murcott, C., The oxidative metabolism of alpha-chlorohydrin in the male rat and the formation of spermatocoeles. Xenobiotica, 1978. 8(9): p. 573-82.
156. Jones, A.R. and O'Brien, R.W., Metabolism of three active analogues of the male antifertility agent alpha-chlorohydrin in the rat. Xenobiotica, 1980. 10(5): p. 365-70.
157. Jones, A.R., Stevenson, D., Hutton, P., and Dawson, A.G., The antifertility action of alpha-chlorohydrin: metabolism by rat and boar sperm. Experientia, 1981. 37(4): p. 340-1.

158. Kalla, N.R., Bansal, M.P., and Werma, K., Response of scrotal and non-scrotal vertebrate testis to the male antifertility agent alpha-chlorohydrin. Acta Eur Fertil, 1979. 10(4): p. 169-76.
159. Kalla, N.R., Kaur, S., Ujwal, N., Mehta, U., Joos, H., and Frick, J., alpha-Glucosidase activity in the rat epididymis under different physiological conditions. Int J Androl, 1997. 20(2): p. 92-5.
160. Kalla, N.R. and Singh, B., Phospholipid analysis in testis-epididymis complex after alpha- chlorohydrin administration. Contraception, 1978. 17(6): p. 523-30.
161. Kalla, N.R. and Singh, B., Effect of sodium nitrite on the alpha-chlorohydrin-induced lesion of the testis--epididymis complexin the rat. J Reprod Fertil, 1979. 56(1): p. 149-51.
162. Kaur, S. and Guraya, S.S., Effects of low doses of alpha chlorohydrin on the dehydrogenases and oxidases of rat epididymal epithelium and sperms: a correlative histochemical and biochemical study. Andrologia, 1981. 13(3): p. 225-31.
163. Kaur, S. and Guraya, S.S., Effect of low doses of alpha chlorohydrin on the enzymes of glycolytic and phosphogluconate pathways in the rat testis and epididymis. Int J Androl, 1981. 4(2): p. 196-207.
164. Kaur, S. and Guraya, S.S., Biochemical observations on the protein and nucleic acid metabolism of the rat testis and epididymis after treatment with low doses of alpha- chlorohydrin. Int J Fertil, 1981. 26(1): p. 8-13.
165. Madhu, N.R., Sarkar, B., Biswas, S.J., Behera, B.K., and Patra, A., Evaluating the Anti-Fertility Potential of alpha-Chlorohydrin on Testis and Spermatozoa in the Adult Male Wild Indian House Rat (Rattus rattus). J Environ Pathol Toxicol Oncol, 2011. 30(2): p. 93-102.
166. Mineshima, H., Fujioka, M., Furukawa, M., Ikeda, T., Kinoshita, K., Koida, M., Kondoh, K., Ozawa, S., Oi, A., Ohyama, N., Takahashi, H., and Tanaka, R., Comparison of sperm motility test methods (except computer-assisted sperm analysis) in rats under the condition of alfa-chlorohydrin treatment--collaborative investigation. J Toxicol Sci, 2000. 25(5): p. 443-54.
167. Morris, I.D., Effect on gonadotrophin secretion of blockage of the ductuli efferentes in the normal and androgen-deprived rat. J Reprod Fertil, 1979. 57(2): p. 469-75.
168. Romero, I.A., Rist, R.J., Chan, M.W., and Abbott, N.J., Acute energy deprivation syndromes: investigation of m-dinitrobenzene and alpha-chlorohydrin toxicity on immortalized rat brain microvessel endothelial cells. Neurotoxicology, 1997. 18(3): p. 781-91.
169. Saini, M.S. and Parshad, V.R., Field evaluation of alpha-chlorohydrin against the Indian mole rat: studies on toxic and antifertility effects. Annals of Applied Biology, 1993. 122(1): p. 153-160.
170. Stutler, S.A., Johnson, E.W., Still, K.R., Schaeffer, D.J., Hess, R.A., and Arfsten, A.D., Effect of method of euthanasia on sperm motility of mature Sprague-Dawley rats. J Am Assoc Lab Anim Sci, 2007. 46(2): p. 13-20.
171. Toth, G.P., Wang, S.R., McCarthy, H., Tocco, D.R., and Smith, M.K., Effects of three male reproductive toxicants on rat cauda epididymal sperm motion. Reprod Toxicol, 1992. 6(6): p. 507-15.
172. Wagenfeld, A., Yeung, C.H., Strupat, K., and Cooper, T.G., Shedding of a rat epididymal sperm protein associated with infertility induced by ornidazole and alpha-chlorohydrin. Biol Reprod, 1998. 58(5): p. 1257-65.
173. Xie, S., Zhu, Y., Ma, L., Lu, Y., Zhou, J., Gui, Y., and Cao, L., Genome-wide profiling of gene expression in the epididymis of alpha-chlorohydrin-induced infertile rats using an oligonucleotide microarray. Reprod Biol Endocrinol, 2010. 8: p. 37.
174. Gordon, E.F., Gordon, R.C., and Passal, D.B., Zinc metabolism: Basic, clinical, and behavioral aspects. The Journal of Pediatrics, 1981. 99(3): p. 341-349.
175. Eby, G.A., Davis, D.R., and Halcomb, W.W., Reduction in duration of common colds by zinc gluconate lozenges in a double-blind study. Antimicrob Agents Chemother, 1984. 25(1): p. 20-4.
176. Hemila, H., Zinc lozenges may shorten the duration of colds: a systematic review. Open Respir Med J, 2011. 5: p. 51-8.
177. Fahim, M.S., Fahim, Z., and Harman, J.M., Chemical sterilization in the male part I: rats. Arch Androl, 1982. 9(3): p. 261-5.
178. Migally, N.B. and Fahim, M.S., Pharmacokinetics of zinc tannate after intratesticular injection. Arch Androl, 1984. 13(2-3): p. 129-36.
179. Tepsumethanon, V., Wilde, H., and Hemachudha, T., Intratesticular injection of a balanced zinc solution for permanent sterilization of dogs. J Med Assoc Thai, 2005. 88(5): p. 686-9.
180. Fahim, M.S., Chemical castration. 1994: USA. p. 14.
181. Fahim, M.S., NEUTERSOL® INJECTABLE SOLUTION for Dogs (Zinc Gluconate Neutralized by Arginine), Administration, F.a.D., Editor. 2003, FDA. p. 141-217.
182. Fahim, M.S., Wang, M., Sutcu, M.F., Fahim, Z., and Youngquist, R.S., Sterilization of dogs with intra-epididymal injection of zinc arginine. Contraception, 1993. 47(1): p. 107-22.
183. Moldave, K. and Briggs, J.R., Contraception and Fertility Control in Dogs and Cats, Dogs, A.f.C.i.C., Editor. 2013, Alliance for Contraception in Cats & Dogs. p. 1-154.
184. Wikipedia, c., Zeuterin. 2019, Wikipedia, The Free Encyclopedia.
185. ACC&D Zeuterin/ Esterilsol product profile and position paper. [Cited 16 January 2016]. 2015.
186. Vanderstichel, R., Forzan, M., Pérez, G.E., Serpell, J., and Garde, E., Changes in blood testosterone concentrations after surgical and chemical sterilization of male free-roaming dogs in southern Chile. Theriogenology, 2014. 83.

187. Benka, V.A.W., Nonsurgical Fertility Control, in Field Manual for Small Animal Medicine. 2018. p. 179-200.
188. Araujo-Lima, C.F., Nunes, R.J., Carpes, R.M., Aiub, C.A., and Felzenszwalb, I., Pharmacokinetic and Toxicological Evaluation of a Zinc Gluconate-Based Chemical Sterilant Using In Vitro and In Silico Approaches. Biomed Res Int, 2017. 2017: p. 5746768.
189. Fagundes, A.K., Oliveira, E.C., Tenorio, B.M., Melo, C.C., Nery, L.T., Santos, F.A., Alves, L.C., Douglas, R.H., and Silva, V.A., Jr., Injection of a chemical castration agent, zinc gluconate, into the testes of cats results in the impairment of spermatogenesis: a potentially irreversible contraceptive approach for this species? Theriogenology, 2014. 81(2): p. 230-6.
190. Oliveira, E.C., Fagundes, A.K., Melo, C.C., Nery, L.T., Revoredo, R.G., Andrade, T.F., Oliveira-Esquerre, K., Kastelic, J.P., and Silva, V.A., Jr., Intratesticular injection of a zinc-based solution for contraception of domestic cats: a randomized clinical trial of efficacy and safety. Vet J, 2013. 197(2): p. 307-10.
191. Oliveira, E.C., Muller, P.M., Silva, F.L., Nery, L.T., de Sa, M.J., Guerra, M.M., Oliveira-Esquerre, K.P., Kastelic, J.P., and Douglas, R.H., Oral administration of an anti-inflammatory does not compromise the efficacy of intra-testicular injection of zinc gluconate as a contraceptive for dogs. Anim Reprod Sci, 2012. 132(3-4): p. 207-12.
192. Oliveira, E.C., Moura, M.R., de Sa, M.J., Silva, V.A., Jr., Kastelic, J.P., Douglas, R.H., and Marques, A.P., Jr., Permanent contraception of dogs induced with intratesticular injection of a Zinc Gluconate-based solution. Theriogenology, 2012. 77(6): p. 1056-63.
193. Oliveira, E.C., Moura, M.R., Silva, V.A., Jr., Peixoto, C.A., Saraiva, K.L., de Sa, M.J., Douglas, R.H., and de Pinho Marques, A., Jr., Intratesticular injection of a zinc-based solution as a contraceptive for dogs. Theriogenology, 2007. 68(2): p. 137-45.
194. Levy, J.K., Crawford, P.C., Appel, L.D., and Clifford, E.L., Comparison of intratesticular injection of zinc gluconate versus surgical castration to sterilize male dogs. Am J Vet Res, 2008. 69(1): p. 140-3.
195. DiGangi, B.A., Grijalva, J., Jaramillo, E.P.P., Dueñas, I., Glenn, C., Cruz, M.E.C., and Pérez, R.P.M., Post-operative outcomes of surgical and chemical castration with zinc gluconate in dogs presenting to veterinary field clinics. Vet J, 2017. 229: p. 26-30.
196. Woodward, K.N., Keesler, R.I., Reader, J.R., and Christe, K.L., Evaluation of a Zinc Gluconate Neutralized with Arginine Product as a Nonsurgical Method for Sterilization of Rhesus Macaques (Macaca mulatta). J Am Assoc Lab Anim Sci, 2017. 56(5): p. 520-526.
197. Rafatmah, D., Mogheiseh, A., and Eshghi, D., Chemical sterilization with intratesticular administration of zinc gluconate in adult dogs: a preliminary report. Basic Clin Androl, 2019. 29: p. 12.
198. Vanderstichel, R., Forzán, M.J., Pérez, G.E., Serpell, J.A., and Garde, E., Changes in blood testosterone concentrations after surgical and chemical sterilization of male free-roaming dogs in southern Chile. Theriogenology, 2015. 83(6): p. 1021-1027.
199. de Macêdo, S.R.B., de Lima, L.A.R., de Torres, S.M., de Oliveira, V.V.G., de Morais, R.N., Peixoto, C.A., Tenorio, B.M., and Junior, V.A.d.S., Effects of intratesticular injection of zinc-based solution in rats in combination with anti-inflammatory and analgesic drugs during chemical sterilization. Veterinary world, 2018. 11(5): p. 649-656.
200. Soto, F.R., Viana, W.G., Mucciolo, G.C., Hosomi, F.Y., Vannucchi, C.I., Mazzei, C.P., Eyherabide, A.R., de Fatima Lucio, C., Dias, R.A., and de Azevedo, S.S., Evaluation of efficacy and safety of zinc gluconate associated with dimethyl sulphoxide for sexually mature canine males chemical neutering. Reprod Domest Anim, 2009. 44(6): p. 927-31.
201. Soto, F., Viana, W., Sousa, A., Pinheiro, S., Mucciolo, G., Hosomi, F., and Azevedo, S., Evaluation of zinc gluconate, either associated or not to dimethyl sulfoxide, as contraceptive method for male dogs. J Anim Reprod, 2007. 4.
202. Vannucchi, C.I., Angrimani, D.S., Eyherabide, A.R., Mazzei, C.P., Lucio, C.F., Maiorka, P.C., Silva, L.C., and Nichi, M., Effects of intratesticular administration of zinc gluconate and dimethyl sulfoxide on clinical, endocrinological, and reproductive parameters in dogs. Theriogenology, 2015. 84(7): p. 1103-10.
203. Brito, L.F., Sertich, P.L., Rives, W., Knobbe, M., Del Piero, F., and Stull, G.B., Effects of intratesticular zinc gluconate treatment on testicular dimensions, echodensity, histology, sperm production, and testosterone secretion in American black bears (Ursus americanus). Theriogenology, 2011. 75(8): p. 1444-52.
204. Forzan, M.J., Garde, E., Perez, G.E., and Vanderstichel, R.V., Necrosuppurative orchitis and scrotal necrotizing dermatitis following intratesticular administration of zinc gluconate neutralized with arginine (EsterilSol) in 2 mixed-breed dogs. Vet Pathol, 2014. 51(4): p. 820-3.
205. Mann, T. and Lutwak-Mann, C., Male reproductive function and semen: themes and trends in physiology, biochemistry and investigative andrology. 1981, New York: Springer-Verlag.
206. Giles, A.J., Hutchinson, M.-K.N.D., Sonnemann, H.M., Jung, J., Fecci, P.E., Ratnam, N.M., Zhang, W., Song, H., Bailey, R., Davis, D., Reid, C.M., Park, D.M., and Gilbert, M.R., Dexamethasone-induced immunosuppression: mechanisms and implications for immunotherapy. Journal for ImmunoTherapy of Cancer, 2018. 6(1): p. 51.
207. Kaur, G., Thompson, L.A., and Dufour, J.M., Sertoli cells – Immunological sentinels of spermatogenesis,. Seminars in Cell & Developmental Biology, 2014. 30: p. 36-44.

208. Matschurat, C., Rode, K., Hollenbach, J., Wolf, K., Urhausen, C., Beineke, A., Günzel-Apel, A.R., and Brehm, R., Impaired spermatogenesis, tubular wall disruption, altered blood-testis barrier composition and intratubular lymphocytes in an infertile Beagle dog - a putative case of autoimmune orchitis. Histol Histopathol, 2019. 34(5): p. 525-535.
209. Kutzler, M. and Wood, A., Non-surgical methods of contraception and sterilization. Theriogenology, 2006. 66(3): p. 514-25.
210. Uddin, J., Hossain, M., Shamsuddin, M., and Hossain, S., Effects of Esterilsol® on Sterilization of Dogs. commonwealth veterinary journal, 2015. 31: p. 9-14.
211. FDA, Freedom of Information Summary. Neutersol® Injectable Solution for Dogs (zinc gluconate neutralized by arginine). Intratesticular injection for chemical sterilization in 3 to 10 month old male dogs. 2003, US Food and Drug Administration: Washington, DC. p. 141-217.
212. Ball, J.J., Kegley, E.B., Lawrence, T.E., Roberts, S.L., Powell, J.G., and Richeson, J.T., Zinc injection as a novel castration method in beef bulls: effects on performance, behavior, and testosterone and haptoglobin concentration. Journal of animal science, 2018. 96(3): p. 890-901.
213. Dixit, V.P. and Lohiya, N.K., Chemical sterilization: effects of a single high dose of 3-chloro-1,2-propanediol on the testes and epididymides of dog. Acta Eur Fertil, 1975. 6(1): p. 57-62.
214. Dixit, V.P., Lohiya, N.K., and Agawal, M., Effects of alpha-chlorohydrin on the testes and epididymides of dog: a preliminary study. Fertil Steril, 1975. 26(8): p. 781-5.
215. Dixit, V.P., Lohiya, N.K., Arya, M., and Agrawal, M., Reversible changes in the testes and epididymides of dog treated with alpha-chlorohydrin. Acta Biol Med Ger, 1975. 34(11-12): p. 1851-6.
216. Abshenas, J., Molaei, M.M., Derakhshnfar, A., and Ghalekhani, N., Chemical Sterilization by Intratesticular Injection of Eugenia Caryophyllata Essential Oil in Dog: A Histopathological Study. Iranian Journal of Veterinary Surgery, 2013. 08(2): p. 9-16.
217. Asha, M.K., Prashanth, D., Murali, B., Padmaja, R., and Amit, A., Anthelmintic activity of essential oil of Ocimum sanctum and eugenol. Fitoterapia, 2001. 72(6): p. 669-670.
218. Jaganathan, S.K. and Supriyanto, E., Antiproliferative and molecular mechanism of eugenol-induced apoptosis in cancer cells. Molecules, 2012. 17(6): p. 6290-304.
219. Ho, Y.C., Huang, F.M., and Chang, Y.C., Mechanisms of cytotoxicity of eugenol in human osteoblastic cells in vitro. Int Endod J, 2006. 39(5): p. 389-93.
220. Abu-Ahmed, H., Chemical Sterilization of Dogs using Single Bilateral Intra-testicular Injection of Calcium Chloride or Clove Oil. Alexandria Journal of Veterinary Sciences, 2015. 45.
221. Elkomy, A., Aboubakr, M., Saber Ibrahim, S., and Abdelhamid, Y., Protective effects of Syzygium aromaticum oil (Clove) against acrylamide induced hepatic, renal, and testicular toxicity in rats. International Journal of Pharmacology and Toxicology, 2018. 6: p. 12.
222. Koger, L.M., Calcium chloride castration. Modern Vet Prac, 1978. 59: p. 119-121.
223. Jana, K. and Samanta, P.K., Evaluation of single intratesticular injection of calcium chloride for nonsurgical sterilization in adult albino rats. Contraception, 2006. 73(3): p. 289-300.
224. Karmakar, S.N. and Das, S.K., CHEMOSTERILIZATION INDUCED BY INTRATESTICULAR INJECTION OF CALCIUM CHLORIDE (CaCl2) - A TOOL FOR POPULATION CONTROL. International Journal of Pharmaceutical, Chemical & Biological Sciences, 2017. 7(1): p. 25-35.
225. Hami, P.M.M., Jahandideh, A.V., Alireza, and Rafiee, S.M., Ultrasonographic and histopathologic study of chemical castration with calcium chloride solution in rat testis. Journal of Critical Reviews, 2020. 7(1): p. 931-935.
226. Samanta, P.K., Chemosterilization of stray dogs. Indian Journal of Animal Health, 1998. 37(1): p. 61-62.
227. Jana, K. and Samanta, P.K., Sterilization of male stray dogs with a single intratesticular injection of calcium chloride: a dose-dependent study. Contraception, 2007. 75(5): p. 390-400.
228. Leoci, R., Aiudi, G., Silvestre, F., Lissner, E.A., Marino, F., and Lacalandra, G.M., A dose-finding, long-term study on the use of calcium chloride in saline solution as a method of nonsurgical sterilization in dogs: evaluation of the most effective concentration with the lowest risk. Acta Vet Scand, 2014. 56: p. 63.
229. Leoci, R., Aiudi, G., Silvestre, F., Lissner, E.A., and Lacalandra, G.M., Alcohol diluent provides the optimal formulation for calcium chloride non-surgical sterilization in dogs. Acta Veterinaria Scandinavica, 2014. 56(1): p. 62.
230. Parsemus, F. Calcium chloride ("Calchlorin") male animal sterilization: Ingredients and procedure. 2014.
231. Puri, B., Shah, M.K., Thakur, B., Regmi, B., and Dhakal, I., Intratesticular injection of calcium chloride Is a useful alternative for neutering the male dog. Int. J. Appl. Sci. Biotechnol., 2018. 6(2): p. 158-163.
232. Silva, R.C.A., Paranzini, C.S., Franco, L.G., Miguel, M.P., Honsho, C.S., and Souza, F.F., Calcium chloride combined with dimethyl sulphoxide for the chemical sterilization of dogs. Reproduction in Domestic Animals, 2018. 53(6): p. 1330-1338.
233. Leoci, R., Aiudi, G., Cicirelli, V., Brent, L., Iaria, C., and Lacalandra, G.M., Effects of intratesticular vs intraepididymal calcium chloride sterilant on testicular morphology and fertility in dogs. Theriogenology, 2019. 127: p. 153-160.
234. Thakre, T., Shukla, S.N., Mishra, A., Gupta, N., and Kumar, P., Sterilization of Male Dogs by Intra-testicular Administration of Calcium Chloride Solution. Indian Journal of Animal Research, 2021.

235. Jana, K. and Samanta, P.K., Clinical evaluation of non-surgical sterilization of male cats with single intra-testicular injection of calcium chloride. BMC veterinary research, 2011. 7: p. 39-39.
236. Paranzini, C.S., Sousa, A.K., Cardoso, G.S., Perencin, F.M., Trautwein, L.G.C., Bracarense, A., and Martins, M.I.M., Effects of chemical castration using 20% CaCl(2) with 0.5% DMSO in tomcats: Evaluation of inflammatory reaction by infrared thermography and effectiveness of treatment. Theriogenology, 2018. 106: p. 253-258.
237. Ijaz, M., Aleem, R.A., Manzoor, A., Qureshi, A.S., Hassan, F., ud Din, M.T.M., ud Din, Z.M., Hayyat, K., and Tabassum, R., Comparative Evaluation of Single, Bilateral Intra-Testicular Injection of Hypertonic Saline Solution and Calcium Chloride as Chemical Sterilizing Agents in Male Cats. Biomed J Sci & Tech Res, 2019. 21(3): p. 15858-15864.
238. Canpolat, I., Gur, S., Gunay, C., Bulut, S., and Eroksuz, H., An evaluation of the outcome of bull castration by intra-testicular injection of ethanol and calcium chloride. Revue de Médecine Vétérinaire, 2006. 157: p. 8-9.
239. Jana, K., Samanta, P.K., and Ghosh, D., Evaluation of single intratesticular injection of calcium chloride for nonsurgical sterilization of male Black Bengal goats (Capra hircus): a dose-dependent study. Anim Reprod Sci, 2005. 86(1-2): p. 89-108.
240. Martins, L.T., Gonçalves, M.C., Tavares, K., Gaudêncio, S., Dos Santos Neto, P., Dias, A.L.G., Gava, A., Saito, M., Oliveira, C., and Vieira, A., Castration methods do not affect weight gain and have diverse impacts on the welfare of water buffalo males. Livestock Science - LIVEST SCI, 2011. 140: p. 171-176.
241. Ibrahim, A., Ali, M.M., Abou-Khalil, N.S., and Ali, M.F., Evaluation of chemical castration with calcium chloride versus surgical castration in donkeys: testosterone as an endpoint marker. BMC Vet Res, 2016. 12: p. 46.
242. Yamada, P.H., Codognoto, V.M., Rydygier de Ruediger, F., Mayara da Silva, K., Aristizábal, V.V., Kastelic, J.P., Rizzoto, G., Ferreira de Souza, F., Gomes de Soutello, R.V., Mungai Chacur, M.G., and Oba, E., A comparison of immunological, chemical and surgical castration of Nelore bulls. Theriogenology, 2021. 169: p. 9-13.
243. Pereira, L.F., Dias, F.C.F., Miguel, M.P., Honsho, C.S., Tavares, D.C., Hellú, J.A.A., and Souza, F.F., Testicular histological evaluation and serum testosterone concentrations of bulls after chemical castration with calcium chloride. Pesquisa Veterinária Brasileira [online], 2018. 38(8): p. 1554-1563.
244. Mitra, B. and Samanta, P.K., Testicular degeneration of scrub bulls by calcium chloride. Indian journal of veterinary surgery, 2000. 21: p. 37-38.
245. Emir, L., Dadali, M., Sunay, M., Erol, D., Caydere, M., and Ustün, H., Chemical castration with intratesticular injection of 20% hypertonic saline: a minimally invasive method. Urol Oncol, 2008. 26(4): p. 392-6.
246. Kwak, B.K. and Lee, S.H., Intratesticular injection of hypertonic saline : non-invasive alternative method for animal castration model. Dev Reprod, 2013. 17(4): p. 435-40.
247. Byung Kuk, K. and Sung-Ho, L., Evaluation of Newly Developed Chemical Castration Method: Changes in Hormone Gene Expression of Hypothalamic-Pituitary Axis. Development & Reproduction, 2017. 21(3): p. 307-315.
248. Maadi, M.-A., Behfar, M., Rasaei, A., Shalizar-Jalali, A., Najafi, G., and Mohammadi, V., Chemical castration using an intratesticular injection of mannitol: a preliminary study in a rat model. Turkish Journal of Veterinary & Animal Sciences, 2021. 45(3): p. 519-530.
249. Canpolat, I., Karabulut, E., and Eroksuz, Y., Chemical Castration of Adult and non-Adult Male Dogs with Sodium Chloride Solution. IOSR Journal of Agriculture and Veterinary Science, 2016. 9: p. 09-11.
250. Neto, O.A., Gasperin, B.G., Rovani, M.T., Ilha, G.F., Nóbrega, J.E., Jr., Mondadori, R.G., Gonçalves, P.B., and Antoniazzi, A.Q., Intratesticular hypertonic sodium chloride solution treatment as a method of chemical castration in cattle. Theriogenology, 2014. 82(7): p. 1007-1011.e1.
251. Oliveira, F.C., Ferreira, C.E.R., Haas, C.S., Oliveira, L.G., Mondadori, R.G., Schneider, A., Rovani, M.T., Gonçalves, P.B.D., Vieira, A.D., Gasperin, B.G., and Lucia, T., Jr., Chemical castration in cattle with intratesticular injection of sodium chloride: Effects on stress and inflammatory markers. Theriogenology, 2017. 90: p. 114-119.
252. Shen, B., Jensen, R.G., and Bohnert, H.J., Mannitol Protects against Oxidation by Hydroxyl Radicals. Plant physiology, 1997. 115(2): p. 527-532.
253. Leoci, R., CHEMICAL CASTRATION BY INTRATESTICULAR INJECTION OF A CALCIUM CHLORIDE IN ALCOHOL SOLUTION IN DOGS. 2012.
254. Russell, L.D., Saxena, N.K., and Weber, J.E., Intratesticular injection as a method to assess the potential toxicity of various agents and to study mechanisms of normal spermatogenesis. Gamete Res, 1987. 17(1): p. 43-56.
255. Bergh, A., Collin, O., and Lissbrant, E., Effects of acute graded reductions in testicular blood flow on testicular morphology in the adult rat. Biol Reprod, 2001. 64(1): p. 13-20.
256. Kaya, M., Sertoli cells and various types of multinucleates in the rat seminiferous tubules following temporary ligation of the testicular artery. J Anat, 1986. 144: p. 15-29.
257. Abou-Khalil, N.S., Ali, M.F., Ali, M.M., and Ibrahim, A., Surgical castration versus chemical castration in donkeys: response of stress, lipid profile and redox potential biomarkers. BMC Vet Res, 2020. 16(1): p. 310.

258. Cheng, C.Y., Wong, E.W., Lie, P.P., Li, M.W., Su, L., Siu, E.R., Yan, H.H., Mannu, J., Mathur, P.P., Bonanomi, M., Silvestrini, B., and Mruk, D.D., Environmental toxicants and male reproductive function. Spermatogenesis, 2011. 1(1): p. 2-13.
259. Parizek, J. and Zahor, Z., Effect of cadmium salts on testicular tissue. Nature, 1956. 177(4518): p. 1036.
260. Parizek, J., The destructive effect of cadmium ion on testicular tissue and its prevention by zinc. J Endocrinol, 1957. 15(1): p. 56-63.
261. Mason, K.E., Brown, J.A., Young, J.O., and Nesbit, R.R., CADMIUM-INDUCED INJURY OF THE RAT TESTIS. Anat Rec, 1964. 149: p. 135-48.
262. Laskey, J.W., Rehnberg, G.L., Laws, S.C., and Hein, J.F., Reproductive effects of low acute doses of cadmium chloride in adult male rats. Toxicol Appl Pharmacol, 1984. 73(2): p. 250-5.
263. Shojaeepour, S., Dabiri, S., Dabiri, B., Imani, M., Fekri Soofi Abadi, M., and Hashemi, F., Histopathological Findings of Testicular Tissue Following Cadmium Toxicity in Rats. Iran J Pathol, 2021. 16(4): p. 348-353.
264. Mason, K.E. and Young, J.O., Effects of cadmium upon the excurrent duct system of the rat testis. Anat Rec, 1967. 159: p. 311-324.
265. Ali, I., Damdimopoulou, P., Stenius, U., Adamsson, A., Makela, S.I., Akesson, A., Berglund, M., Hakansson, H., and Halldin, K., Cadmium-induced effects on cellular signaling pathways in the liver of transgenic estrogen reporter mice. Toxicol Sci, 2012. 127(1): p. 66-75.
266. Kar, A.B., Chemical sterilization of male Rhesus monkeys. Endocrinology, 1961. 69: p. 1116-9.
267. Setty, B.S. and Kar, A.B., CHEMICAL STERILIZATION OF MALE FROGS (RANA TIGRINA DAUD.). Gen Comp Endocrinol, 1964. 4: p. 353-9.
268. Chatterjee, S.N. and Kar, A.B., Chemical sterilization of stray dogs. Indian Vet J, 1968. 45(8): p. 649-54.
269. Singh, S.K. and Dominic, C.J., Sterilization of a non-scrotal mammal (Suncus murinus L.) by intratesticular injection of cadmium chloride. Acta Eur Fertil, 1978. 9(1): p. 65-70.
270. Kar, A.B. and Das, R.P., Sterilization of males by intratesticular administration of cadmium chloride. Acta Endocrinologica, 1962. 40(3): p. 321-331.
271. Monsefi, M., Alaee, S., Moradshahi, A., and Rohani, L., Cadmium-induced infertility in male mice. Environ Toxicol, 2009.
272. Ren, Y., Shao, W., Zuo, L., Zhao, W., Qin, H., Hua, Y., Lu, D., Mi, C., Zeng, S., and Zu, L., Mechanism of cadmium poisoning on testicular injury in mice. Oncology Letters, 2019. 18(2): p. 1035-1042.
273. Zhu, Q., Li, X., and Ge, R.S., Toxicological Effects of Cadmium on Mammalian Testis. Front Genet, 2020. 11: p. 527.
274. Prozialeck, W.C., Edwards, J.R., Nebert, D.W., Woods, J.M., Barchowsky, A., and Atchison, W.D., The vascular system as a target of metal toxicity. Toxicol Sci, 2008. 102(2): p. 207-18.
275. Angeli, J.K., Cruz Pereira, C.A., de Oliveira Faria, T., Stefanon, I., Padilha, A.S., and Vassallo, D.V., Cadmium exposure induces vascular injury due to endothelial oxidative stress: the role of local angiotensin II and COX-2. Free Radic Biol Med, 2013. 65: p. 838-848.
276. Setchell, B.P. and Waites, G.M., Changes in the permeability of the testicular capillaries and of the 'blood-testis barrier' after injection of cadmium chloride in the rat. J Endocrinol, 1970. 47(1): p. 81-6.
277. Wan, H.T., Mruk, D.D., Wong, C.K., and Cheng, C.Y., The apical ES-BTB-BM functional axis is an emerging target for toxicant-induced infertility. Trends Mol Med, 2013. 19(7): p. 396-405.
278. Wong, C.H. and Cheng, C.Y., The blood-testis barrier: its biology, regulation, and physiological role in spermatogenesis. Curr Top Dev Biol, 2005. 71: p. 263-96.
279. Li, N., Wang, T., and Han, D., Structural, cellular and molecular aspects of immune privilege in the testis. Front Immunol, 2012. 3: p. 152.
280. Franca, L.R., Auharek, S.A., Hess, R.A., Dufour, J.M., and Hinton, B.T., Blood-tissue barriers: morphofunctional and immunological aspects of the blood-testis and blood-epididymal barriers. Adv Exp Med Biol, 2012. 763: p. 237-59.
281. Wang, L., Yan, M., Li, H., Wu, S., Ge, R., Wong, C.K.C., Silvestrini, B., Sun, F., and Cheng, C.Y., The Non-hormonal Male Contraceptive Adjudin Exerts its Effects via MAPs and Signaling Proteins mTORC1/rpS6 and FAK-Y407. Endocrinology, 2021. 162(1).
282. Hew, K.W., Heath, G.L., Jiwa, A.H., and Welsh, M.J., Cadmium in vivo causes disruption of tight junction-associated microfilaments in rat Sertoli cells. Biol Reprod, 1993. 49(4): p. 840-9.
283. Rios, C. and Méndez-Armenta, M., Cadmium Neurotoxicity, in Encyclopedia of Environmental Health (Second Edition), Nriagu, J., Editor. 2019, Elsevier: Oxford. p. 485-491.
284. Heath, E. and Arowolo, R., The early histopathologic effects of intratesticular injection with hyperosmolar glycerol, glucose or NaCl solutions. Andrologia, 1987. 19(6): p. 654-61.
285. Igdoura, S.A. and Wiebe, J.P., Suppression of spermatogenesis by low-level glycerol treatment. J Androl, 1994. 15(3): p. 234-43.
286. Wiebe, J.P., Biological Contraceptive for Males, Patent, U.S., Editor. 1988, The University of Western Ontario London, Canada: USA.
287. Wiebe, J.P., Barr, K.J., Buckingham, K.D., and Gedded, P.D., Prospects of a male contraceptive based on the selective antispermatogenic action of 1,2,3-trihydroxypropane (THP, glycerol), in Male contraception: advances and future prospects, Zatuchni, G.I., Goldsmith, A., Spieler, J.M., Sciearra, J., and Zatuchni, G.I., Editors. 1986, Harper and Row: Philadelphia. p. 252–270.

288. Wiebe, J.P. and Barr, K.J., The control of male fertility by 1,2,3-trihydroxypropane (THP;glycerol): rapid arrest of spermatogenesis without altering libido, accessory organs, gonadal steroidogenesis, and serum testosterone, LH and FSH. Contraception, 1984. 29(3): p. 291-302.
289. Wiebe, J.P. and Barr, K.J., Suppression of spermatogenesis without inhibition of steroidogenesis by a 1,2,3-trihydroxypropane solution. Life Sci, 1984. 34(18): p. 1747-54.
290. Weinbauer, G.F., Galhotra, M.M., and Nieschlag, E., Focal testicular destruction following intratesticular injection of glycerol in rats. Int J Androl, 1985. 8(5): p. 365-75.
291. Madbouly, H., Korany, R., El-Shahat, K., Eissa, H., and Fathi, M., Efficacy of Intratesticular Glycerol Injection as Male Cat Contraception in Comparison with Two Surgical Approaches. Top Companion Anim Med, 2021. 42: p. 100493.
292. Immegart, H.M. and Threlfall, W.R., Evaluation of intratesticular injection of glycerol for nonsurgical sterilization of dogs. Am J Vet Res, 2000. 61: p. 544–549.
293. Stagg, C.M. and Tribble, R.L., Chemical Canstration, Patent, U.S., Editor. 1982, Philips, Roxane, Inc., St. Joseph, MO: USA.
294. Nishimura, N., Kawate, N., Sawada, T., and Mori, J., Chemical castration by a single intratesticular injection of lactic acid in rats and dogs. Journal of Reproduction and Development, 1992. 38: p. 263-266.
295. Hill, G.M., Neville, W.E., Richardson, K.L., Utley, P.R., and Stewart, R.L., Castration Method and Progesterone-Estradiol Implant Effects on Growth Rate of Suckling Calves1, 2. Journal of Dairy Science, 1985. 68(11): p. 3059-3061.
296. Fordyce, G., Hodge, P.B., Beaman, N.J., Laing, A.R., Campero, C., and Shepherd, R.K., An evaluation of calf castration by intra-testicular injection of a lactic acid solution. Aust Vet J, 1989. 66(9): p. 272-6.
297. Giri, S., Yadav, B.P.S., and Panda, S.K., Chemical castration in pigs. The Indian journal of animal sciences, 2002. 72: p. 451-453.
298. Freeman, C. and Coffey, D.S., Sterility in male animals induced by injection of chemical agents into the vas deferens. Fertil Steril, 1973. 24(11): p. 884-90.
299. Kamboj, V.P. and Kar, A.B., Antitesticular effect of metallic and rare earth salts. J Reprod Fertil, 1964. 7: p. 21-8.
300. Kar, A.B., Kamboj, V.P., and Goswami, A., Sterilization of male rhesus monkeys by iron salts. J Reprod Fertil, 1965. 9: p. 115-7.
301. Freund, J., Lipton, M.M., and Thompson, G.E., Aspermatogenesis in the guinea pig induced by testicular tissue and adjuvants. J Exp Med, 1953. 97(5): p. 711-26.
302. Jacobo, P., Guazzone, V.A., Theas, M.S., and Lustig, L., Testicular autoimmunity. Autoimmun Rev, 2011. 10(4): p. 201-4.
303. Eyquem, A. and Kreig, H., Experimental autosensitization of the testis. Ann N Y Acad Sci, 1965. 124(1): p. 270-8.
304. Boughton, B.J. and Spector, W.G., "Auto-immune" testicular lesions induced by injury to the contralateral testis and intradermal injection of adjuvant. The Journal of pathology and bacteriology, 1963. 86: p. 69-74.
305. Katsh, S., ADJUVANTS AND ASPERMATOGENESIS IN THE GUINEA PIG. Int Arch Allergy Appl Immunol, 1964. 24: p. 319-31.
306. Naz, R.K. and Talwar, G.P., Immunological sterilization of male dogs by BCG. Int J Androl, 1981. 4(1): p. 111-28.
307. Talwar, G.P., Naz, R.K., Das, C., and Das, R.P., A practicable immunological approach to block spermatogenesis without loss of androgens. Proc Natl Acad Sci U S A, 1979. 76(11): p. 5882-5.
308. Park, H.K., Paick, S.H., Kim, H.G., Lho, Y.S., and Bae, S.R., Induction of contraception by intraepididymal sclerotherapy. The world journal of men's health, 2014. 32(2): p. 83-86.
309. Dixit, V.P., Agrawal, M., and Jain, H.C., Effects of chemical occlusion of vas deferens on the reproductive organs in gerbil Meriones hurrianae Jerdon. Indian J Exp Biol, 1976. 14(5): p. 617-9.
310. Freeman, C. and Coffey, D.S., Male sterility induced by ethanol injection into the vas deferens. Int J Fertil, 1973. 18(3): p. 129-32.
311. Sharma, J.D., Chinoy, N.J., and Dixit, V.P., Fertility control in vas occluded rats and the biochemical effects of ascorbic acid feeding. Exp Clin Endocrinol, 1983. 82(3): p. 337-41.
312. Raman, G., Purandare, T.V., and Munshi, S.R., Sterility induced in male rats by injection of chemical agents into the vas deferens. Andrologia, 1976. 8(4): p. 321-5.
313. Dixit, V.P., Lohiya, N.K., Arya, M., and Agrawal, M., The effects of chemical occlusion of vas deferens on the testicular function of dog: a preliminary study. Acta Eur Fertil, 1975. 6(4): p. 348-53.
314. Pineda, M.H. and Dooley, M.P., Surgical and chemical vasectomy in the cat. Am J Vet Res, 1984. 45(2): p. 291-300.
315. Pineda, M.H., Reimers, T.J., Faulkner, L.C., Hopwood, M.L., and Seidel, G.E., Jr., Azoospermia in dogs induced by injection of sclerosing agents into the caudae of the epididymides. Am J Vet Res, 1977. 38(6): p. 831-8.
316. Singh, S.K. and Chakravarty, S., Histologic changes in the mouse testis after bilateral vasectomy. Asian J Androl, 2000. 2(2): p. 115-20.
317. Barnett, B.D., Chemical vasectomy of domestic dogs in the Galapagos islands. Theriogenology, 1985. 23(3): p. 499-509.
318. Hoffman, R.A. and Wright, R.G., Fertility control in a non-native population of mountain goats. Northwest Science, 1990. 64(1): p. 1-6.
319. Lewis, R.W. and Garcia, R.R., The results of epididymal ablation by sclerosing agents in the nonhuman primate. Fertil Steril, 1984. 41(3): p. 465-9.

320. Kutzler, M.A., Intratesticular and intraepididymal injections to sterilize male cats: From calcium chloride to zinc gluconate and beyond. J Feline Med Surg, 2015. 17(9): p. 772-6.
321. Plant, J.W., Seaman, J.T., and Jakovljevic, D., Non-surgical sterilisation of rams using a sclerosing agent. Aust Vet J, 1979. 55(6): p. 263-4.
322. Chen, Z.W., Gu, Y.Q., Liang, X.W., Wu, Z.G., Yin, E.J., and Li, H., Safety and efficacy of percutaneous injection of polyurethane elastomer (MPU) plugs for vas occlusion in man. Int J Androl, 1992. 15(6): p. 468-72.
323. SHENG-CAI, Z., SHU-PING, Z., and RUI-CHUAN, Y., Intravasal injection of formed-in-place silicone rubber as a method of vas occlusion. International Journal of Andrology, 1992. 15(6): p. 460-464.
324. SHENG-CAI, Z., YI-HE, L., RUI-CHUAN, Y., and SHU-PING, Z., Recovery of fertility after removal of polyurethane plugs from the human vas deferens occluded for up to 5 years. International Journal of Andrology, 1992. 15(6): p. 465-467.
325. Lohiya, N.K., Manivannan, B., and Mishra, P.K., Repeated vas occlusion and non-invasive reversal with styrene maleic anhydride for male contraception in langur monkeys. Int J Androl, 2000. 23(1): p. 36-42.
326. Lohiya, N.K., Manivannan, B., Mishra, P.K., and Pathak, N., Vas deferens, a site of male contraception: an overview. Asian J Androl, 2001. 3(2): p. 87-95.
327. Lohiya, N.K., Manivannan, B., Mishra, P.K., Sriram, S., Bhande, S.S., and Panneerdoss, S., Preclinical evaluation for noninvasive reversal following long-term vas occlusion with styrene maleic anhydride in langur monkeys. Contraception, 2005. 71(3): p. 214-26.
328. Ansari, A.S., Hussain, M., Khan, S.R., and Lohiya, N.K., Relative suitability of DMSO and NaHCO3 for reversal of RISUG® induced long-term contraception. Andrology, 2016. 4(2): p. 306-13.
