# Supplementary material for: Male animal sterilization: history, current practices, and potential methods for replacing castration
Source: Front Vet Sci. 2024 Jul 3;11:1409386. doi: 10.3389/fvets.2024.1409386 (PMC11255590; doi:10.3389/fvets.2024.1409386)
Supplement: Supplementary file 2 [file Table_2.pdf]

Supplemental Table 2 References. Chemical Castration by Intratesticular Administration and Potential Sterilizing Effects in Male Animals

Target Organ	Admin[1]	Chemical	Trade Name[3]	Species	Dosage	Original Use	Short-term Pathology	Long-term Pathology	Mechanisms of action	T Decreased[3]	Inflammation	Side-Effects	Refs[4]
Testis	Intra-testicular[4]	Zinc compounds	Kastrin, Neutersol™, Zeuterin™, EsterilSol™, Testoblock®, Infertile®	Rat, Dog, Cat, Monkey, Bear, Pig	2.6-58 mg/ml	Dietary supplement; treatment of common cold	Multinucleated giant cells; acute inflammation; edema; testis swelling	Seminiferous tubule necrosis; atrophy;	Sertoli cell barrier damage; massive neutrophilic inflammation; necrosis; tubular fibrosis	Yes	Yes	Scrotal ulceration and dermatitis; necrotizing reactions	1
		4-allyl-2-methoxyphenol	Eugenol Clove oil	Dog	1 ml	Cosmetics; anti-anthelmintic; expectorant	Testis swelling	Seminiferous tubular fibrosis	Testicular necrosis; inflammation	Yes	Yes	Epididymal vacuolation	2
		$CaCl_2$ +/- ethanol & hypertonic NaCl	Calchlorin™, Salts	Rat, Dog, Cat, Bull, Goat, Donkey, Ram, Guinea pig, Buffalo	20-50% in water or ethanol	Inorganic salt; food additive; deicing salt; desiccant	Testis swelling; inflammation	Testicular atrophy	Testicular dehydration; necrosis; coagulative fibrosis	Yes	Yes	Scrotal necrosis if leakage	3
		$CdCl_2$	Metallic salt	Rat, Mouse, Dog, Rabbit, Hamster	150 µ/kg	Inorganic salt pigment	Hemorrhage; edema; inflammation	Testicular atrophy	Decreased blood flow; ischemia; oxidative stress	Yes	Yes	Toxic to numerous organs, if given oral	4
		1,2,3-trlhydroxypropane	Glycerol	Rat, Dog, Cat, Monkey, Rabbit	10-70%	Food additive; soap; laxative	Seminiferous tubule disruption	Testicular atrophy	Hyperosmotic dehydration	Yes	Yes	None	5
		Lactic acid	Chem-Cast®	Rat, Dog, Bull	85-92%	Organic synthesis; food additive	Germ cell degeneration	Testicular atrophy	Sclerosing, caustic chemical; necrosis; fibrosis	Yes	Yes	Scrotal necrosis if leakage	6
		$KMnO_4$	N/A	Pig	0.25 g/17 ml acetic acid	Inorganic oxidizing agent; water treatment	N/A	Seminiferous tubular atrophy; fibrosis efferent ducts	Sclerosing, caustic chemical	N/A	Yes	Leydig cell hypertrophy	7
		Metallic & rare earth salts	N/A	Rat, Mouse, Monkey	0.02-0.08 mM/kg	Salts	Focal necrosis	Testicular total necrosis for some salts	Cytolysis, ischemia, necrosis	N/A	Yes	Some Leydig cell loss; early effects on vas deferens sperm; some salts showed no effect	8
		Ethanol	Alcohol	Bull	100%; 10 ml	Organic solvent	Testis swelling; necrosis	Testicular atrophy	Necrosis; inflammation; edema; fibrosis	Yes	Yes	Only 50% became infertile	9
	Intra-Testicular; s.c or i.m	Inflammatory induction agents[2]	N/A	Guinea pig, Monkey, Rat, Rabbit; Dog; Ram	N/A	Antibody stimulating	Inflammation; seminiferous tubular dilation; testis swelling	Granuloma; necrosis; testicular atrophy	Inflammation;	No	Yes	Injection outside testis required for azoospermia for some agents	10

[1] Administration of the chemical/drug by intra-testicular injection or with subcutaneous or cutaneous (s.c.), intramuscular (i.m.) injection

[2] Testicular antigens +/- killed bacteria; killed mycobacteria; Freund's complete adjuvant injected into foot after intratesticular injection of turpentine; Freund's complete adjuvant plus testis homogenate

[3] N/A, not applicable; T, testosterone

[4] Supplemental References:

1: Zinc compounds [1-41]

2: 4-allyl-2-methoxyphenol [42-47]

3: $CaCl_2$ +/- ethanol and hypertonic $NaCl_2$ [46, 48-84]

4: $CdCl_2$ [85-110]
5: 1,2,3-trlhydroxypropane [111-119]
6: Lactic Acid [120-123]
7: Potassium permanganate [124, 125]
8: Metallic and rare earth salts [126, 127]
9: Ethanol [64]
10: Inflammatory induction agents [35, 128-134]

References:

1. Gordon, E.F., Gordon, R.C., and Passal, D.B., Zinc metabolism: Basic, clinical, and behavioral aspects. The Journal of Pediatrics, 1981. 99(3): p. 341-349.
2. Eby, G.A., Davis, D.R., and Halcomb, W.W., Reduction in duration of common colds by zinc gluconate lozenges in a double-blind study. Antimicrob Agents Chemother, 1984. 25(1): p. 20-4.
3. Hemila, H., Zinc lozenges may shorten the duration of colds: a systematic review. Open Respir Med J, 2011. 5: p. 51-8.
4. Fahim, M.S., Fahim, Z., and Harman, J.M., Chemical sterilization in the male part I: rats. Arch Androl, 1982. 9(3): p. 261-5.
5. Migally, N.B. and Fahim, M.S., Pharmacokinetics of zinc tannate after intratesticular injection. Arch Androl, 1984. 13(2-3): p. 129-36.
6. Tepsumethanon, V., Wilde, H., and Hemachudha, T., Intratesticular injection of a balanced zinc solution for permanent sterilization of dogs. J Med Assoc Thai, 2005. 88(5): p. 686-9.
7. Fahim, M.S., Chemical castration. 1994: USA. p. 14.
8. Fahim, M.S., NEUTERSOL® INJECTABLE SOLUTION for Dogs (Zinc Gluconate Neutralized by Arginine). 2003, FDA: https://animaldrugsatfda.fda.gov/adafda/app/search/public/document/downloadFoi/748. p. 141-217.
9. Fahim, M.S., Wang, M., Sutcu, M.F., Fahim, Z., and Youngquist, R.S., Sterilization of dogs with intra-epididymal injection of zinc arginine. Contraception, 1993. 47(1): p. 107-22.
10. Moldave, K. and Briggs, J.R., Contraception and Fertility Control in Dogs and Cats, Dogs, A.f.C.i.C., Editor. 2013, Alliance for Contraception in Cats & Dogs. p. 1-154.
11. Wikipedia, c., Zeuterin. 2019, Wikipedia, The Free Encyclopedia.
12. ACC&D Zeuterin/ Esterilsol product profile and position paper. [Cited 16 January 2016]. 2015.
13. Vanderstichel, R., Forzan, M., Pérez, G.E., Serpell, J., and Garde, E., Changes in blood testosterone concentrations after surgical and chemical sterilization of male free-roaming dogs in southern Chile. Theriogenology, 2015. 83: p. 1021–1027.
14. Benka, V.A.W., Nonsurgical Fertility Control, in Field Manual for Small Animal Medicine, Polak, J.M. and Kommedal, A.T., Editors. 2018, John Wiley & Sons. p. 179-200.
15. Araujo-Lima, C.F., Nunes, R.J., Carpes, R.M., Aiub, C.A., and Felzenszwalb, I., Pharmacokinetic and Toxicological Evaluation of a Zinc Gluconate-Based Chemical Sterilant Using In Vitro and In Silico Approaches. Biomed Res Int, 2017. 2017: p. 5746768.
16. Fagundes, A.K., Oliveira, E.C., Tenorio, B.M., Melo, C.C., Nery, L.T., Santos, F.A., Alves, L.C., Douglas, R.H., and Silva, V.A., Jr., Injection of a chemical castration agent, zinc gluconate, into the testes of cats results in the impairment of spermatogenesis: a potentially irreversible contraceptive approach for this species? Theriogenology, 2014. 81(2): p. 230-6.
17. Oliveira, E.C., Fagundes, A.K., Melo, C.C., Nery, L.T., Revoredo, R.G., Andrade, T.F., Oliveira-Esquerre, K., Kastelic, J.P., and Silva, V.A., Jr., Intratesticular injection of a zinc-based solution for contraception of domestic cats: a randomized clinical trial of efficacy and safety. Vet J, 2013. 197(2): p. 307-10.
18. Oliveira, E.C., Muller, P.M., Silva, F.L., Nery, L.T., de Sa, M.J., Guerra, M.M., Oliveira-Esquerre, K.P., Kastelic, J.P., and Douglas, R.H., Oral administration of an anti-inflammatory does not compromise the efficacy of intra-testicular injection of zinc gluconate as a contraceptive for dogs. Anim Reprod Sci, 2012. 132(3-4): p. 207-12.
19. Oliveira, E.C., Moura, M.R., de Sa, M.J., Silva, V.A., Jr., Kastelic, J.P., Douglas, R.H., and Marques, A.P., Jr., Permanent contraception of dogs induced with intratesticular injection of a Zinc Gluconate-based solution. Theriogenology, 2012. 77(6): p. 1056-63.
20. Oliveira, E.C., Moura, M.R., Silva, V.A., Jr., Peixoto, C.A., Saraiva, K.L., de Sa, M.J., Douglas, R.H., and de Pinho Marques, A., Jr., Intratesticular injection of a zinc-based solution as a contraceptive for dogs. Theriogenology, 2007. 68(2): p. 137-45.
21. Levy, J.K., Crawford, P.C., Appel, L.D., and Clifford, E.L., Comparison of intratesticular injection of zinc gluconate versus surgical castration to sterilize male dogs. Am J Vet Res, 2008. 69(1): p. 140-3.

22. DiGangi, B.A., Grijalva, J., Jaramillo, E.P.P., Dueñas, I., Glenn, C., Cruz, M.E.C., and Pérez, R.P.M., Post-operative outcomes of surgical and chemical castration with zinc gluconate in dogs presenting to veterinary field clinics. Vet J, 2017. 229: p. 26-30.
23. Woodward, K.N., Keesler, R.I., Reader, J.R., and Christe, K.L., Evaluation of a Zinc Gluconate Neutralized with Arginine Product as a Nonsurgical Method for Sterilization of Rhesus Macaques (Macaca mulatta). J Am Assoc Lab Anim Sci, 2017. 56(5): p. 520-526.
24. Rafatmah, D., Mogheiseh, A., and Eshghi, D., Chemical sterilization with intratesticular administration of zinc gluconate in adult dogs: a preliminary report. Basic Clin Androl, 2019. 29: p. 12.
25. de Macêdo, S.R.B., de Lima, L.A.R., de Torres, S.M., de Oliveira, V.V.G., de Morais, R.N., Peixoto, C.A., Tenorio, B.M., and Junior, V.A.d.S., Effects of intratesticular injection of zinc-based solution in rats in combination with anti-inflammatory and analgesic drugs during chemical sterilization. Veterinary world, 2018. 11(5): p. 649-656.
26. Soto, F.R., Viana, W.G., Mucciolo, G.C., Hosomi, F.Y., Vannucchi, C.I., Mazzei, C.P., Eyherabide, A.R., de Fatima Lucio, C., Dias, R.A., and de Azevedo, S.S., Evaluation of efficacy and safety of zinc gluconate associated with dimethyl sulphoxide for sexually mature canine males chemical neutering. Reprod Domest Anim, 2009. 44(6): p. 927-31.
27. Soto, F., Viana, W., Sousa, A., Pinheiro, S., Mucciolo, G., Hosomi, F., and Azevedo, S., Evaluation of zinc gluconate, either associated or not to dimethyl sulfoxide, as contraceptive method for male dogs. J Anim Reprod, 2007. 4: p. 119-124.
28. Vannucchi, C.I., Angrimani, D.S., Eyherabide, A.R., Mazzei, C.P., Lucio, C.F., Maiorka, P.C., Silva, L.C., and Nichi, M., Effects of intratesticular administration of zinc gluconate and dimethyl sulfoxide on clinical, endocrinological, and reproductive parameters in dogs. Theriogenology, 2015. 84(7): p. 1103-10.
29. Brito, L.F., Sertich, P.L., Rives, W., Knobbe, M., Del Piero, F., and Stull, G.B., Effects of intratesticular zinc gluconate treatment on testicular dimensions, echodensity, histology, sperm production, and testosterone secretion in American black bears (Ursus americanus). Theriogenology, 2011. 75(8): p. 1444-52.
30. Forzan, M.J., Garde, E., Perez, G.E., and Vanderstichel, R.V., Necrosuppurative orchitis and scrotal necrotizing dermatitis following intratesticular administration of zinc gluconate neutralized with arginine (EsterilSol) in 2 mixed-breed dogs. Vet Pathol, 2014. 51(4): p. 820-3.
31. Mann, T. and Lutwak-Mann, C., Male reproductive function and semen: themes and trends in physiology, biochemistry and investigative andrology. 1981, New York: Springer-Verlag.
32. Giles, A.J., Hutchinson, M.-K.N.D., Sonnemann, H.M., Jung, J., Fecci, P.E., Ratnam, N.M., Zhang, W., Song, H., Bailey, R., Davis, D., Reid, C.M., Park, D.M., and Gilbert, M.R., Dexamethasone-induced immunosuppression: mechanisms and implications for immunotherapy. Journal for ImmunoTherapy of Cancer, 2018. 6(1): p. 51.
33. Kaur, G., Thompson, L.A., and Dufour, J.M., Sertoli cells – Immunological sentinels of spermatogenesis,. Seminars in Cell & Developmental Biology, 2014. 30: p. 36-44.
34. Matschurat, C., Rode, K., Hollenbach, J., Wolf, K., Urhausen, C., Beineke, A., Günzel-Apel, A.R., and Brehm, R., Impaired spermatogenesis, tubular wall disruption, altered blood-testis barrier composition and intratubular lymphocytes in an infertile Beagle dog - a putative case of autoimmune orchitis. Histol Histopathol, 2019. 34(5): p. 525-535.
35. Kutzler, M. and Wood, A., Non-surgical methods of contraception and sterilization. Theriogenology, 2006. 66(3): p. 514-25.
36. Uddin, J., Hossain, M., Shamsuddin, M., and Hossain, S., Effects of Esterilsol® on Sterilization of Dogs. commonwealth veterinary journal, 2015. 31: p. 9-14.
37. FDA, Freedom of Information Summary. Neutersol® Injectable Solution for Dogs (zinc gluconate neutralized by arginine). Intratesticular injection for chemical sterilization in 3 to 10 month old male dogs. 2003, US Food and Drug Administration: Washington, DC. p. 141-217.
38. Ball, J.J., Kegley, E.B., Lawrence, T.E., Roberts, S.L., Powell, J.G., and Richeson, J.T., Zinc injection as a novel castration method in beef bulls: effects on performance, behavior, and testosterone and haptoglobin concentration. Journal of animal science, 2018. 96(3): p. 890-901.
39. Dixit, V.P. and Lohiya, N.K., Chemical sterilization: effects of a single high dose of 3-chloro-1,2-propanediol on the testes and epididymides of dog. Acta Eur Fertil, 1975. 6(1): p. 57-62.
40. Dixit, V.P., Lohiya, N.K., and Agawal, M., Effects of alpha-chlorohydrin on the testes and epididymides of dog: a preliminary study. Fertil Steril, 1975. 26(8): p. 781-5.
41. Dixit, V.P., Lohiya, N.K., Arya, M., and Agrawal, M., Reversible changes in the testes and epididymides of dog treated with alpha-chlorohydrin. Acta Biol Med Ger, 1975. 34(11-12): p. 1851-6.
42. Abshenas, J., Molaei, M.M., Derakhshnfar, A., and Ghalekhani, N., Chemical Sterilization by Intratesticular Injection of Eugenia Caryophyllata Essential Oil in Dog: A Histopathological Study. Iranian Journal of Veterinary Surgery, 2013. 08(2): p. 9-16.
43. Asha, M.K., Prashanth, D., Murali, B., Padmaja, R., and Amit, A., Anthelmintic activity of essential oil of Ocimum sanctum and eugenol. Fitoterapia, 2001. 72(6): p. 669-670.
44. Jaganathan, S.K. and Supriyanto, E., Antiproliferative and molecular mechanism of eugenol-induced apoptosis in cancer cells. Molecules, 2012. 17(6): p. 6290-304.
45. Ho, Y.C., Huang, F.M., and Chang, Y.C., Mechanisms of cytotoxicity of eugenol in human osteoblastic cells in vitro. Int Endod J, 2006. 39(5): p. 389-93.
46. Abu-Ahmed, H., Chemical Sterilization of Dogs using Single Bilateral Intra-testicular Injection of Calcium Chloride or Clove Oil. Alexandria Journal of Veterinary Sciences, 2015. 45.

47. Elkomy, A., Aboubakr, M., Saber Ibrahim, S., and Abdelhamid, Y., Protective effects of Syzygium aromaticum oil (Clove) against acrylamide induced hepatic, renal, and testicular toxicity in rats. International Journal of Pharmacology and Toxicology, 2018. 6: p. 12.
48. Koger, L.M., Calcium chloride castration. Modern Vet Prac, 1978. 59: p. 119-121.
49. Jana, K. and Samanta, P.K., Evaluation of single intratesticular injection of calcium chloride for nonsurgical sterilization in adult albino rats. Contraception, 2006. 73(3): p. 289-300.
50. Karmakar, S.N. and Das, S.K., CHEMOSTERILIZATION INDUCED BY INTRATESTICULAR INJECTION OF CALCIUM CHLORIDE (CaCl2) - A TOOL FOR POPULATION CONTROL. International Journal of Pharmaceutical, Chemical & Biological Sciences, 2017. 7(1): p. 25-35.
51. Hami, P.M.M., Jahandideh, A.V., Alireza, and Rafiee, S.M., Ultrasonographic and histopathologic study of chemical castration with calcium chloride solution in rat testis. Journal of Critical Reviews, 2020. 7(1): p. 931-935.
52. Samanta, P.K., Chemosterilization of stray dogs. Indian Journal of Animal Health, 1998. 37(1): p. 61-62.
53. Jana, K. and Samanta, P.K., Sterilization of male stray dogs with a single intratesticular injection of calcium chloride: a dose-dependent study. Contraception, 2007. 75(5): p. 390-400.
54. Leoci, R., Aiudi, G., Silvestre, F., Lissner, E.A., Marino, F., and Lacalandra, G.M., A dose-finding, long-term study on the use of calcium chloride in saline solution as a method of nonsurgical sterilization in dogs: evaluation of the most effective concentration with the lowest risk. Acta Vet Scand, 2014. 56: p. 63.
55. Leoci, R., Aiudi, G., Silvestre, F., Lissner, E.A., and Lacalandra, G.M., Alcohol diluent provides the optimal formulation for calcium chloride non-surgical sterilization in dogs. Acta Veterinaria Scandinavica, 2014. 56(1): p. 62.
56. Anonymous Calcium chloride (“Calchlorin") male animal sterilization: Ingredients and procedure. Parsemus Foundation, 2014.
57. Puri, B., Shah, M.K., Thakur, B., Regmi, B., and Dhakal, I., Intratesticular injection of calcium chloride Is a useful alternative for neutering the male dog. Int. J. Appl. Sci. Biotechnol., 2018. 6(2): p. 158-163.
58. Silva, R.C.A., Paranzini, C.S., Franco, L.G., Miguel, M.P., Honsho, C.S., and Souza, F.F., Calcium chloride combined with dimethyl sulphoxide for the chemical sterilization of dogs. Reproduction in Domestic Animals, 2018. 53(6): p. 1330-1338.
59. Leoci, R., Aiudi, G., Cicirelli, V., Brent, L., Iaria, C., and Lacalandra, G.M., Effects of intratesticular vs intraepididymal calcium chloride sterilant on testicular morphology and fertility in dogs. Theriogenology, 2019. 127: p. 153-160.
60. Thakre, T., Shukla, S.N., Mishra, A., Gupta, N., and Kumar, P., Sterilization of Male Dogs by Intra-testicular Administration of Calcium Chloride Solution. Indian Journal of Animal Research, 2023. 57(8): p. 1091-1095.
61. Jana, K. and Samanta, P.K., Clinical evaluation of non-surgical sterilization of male cats with single intra-testicular injection of calcium chloride. BMC veterinary research, 2011. 7: p. 39-39.
62. Paranzini, C.S., Sousa, A.K., Cardoso, G.S., Perencin, F.M., Trautwein, L.G.C., Bracarense, A., and Martins, M.I.M., Effects of chemical castration using 20% CaCl(2) with 0.5% DMSO in tomcats: Evaluation of inflammatory reaction by infrared thermography and effectiveness of treatment. Theriogenology, 2018. 106: p. 253-258.
63. Ijaz, M., Aleem, R.A., Manzoor, A., Qureshi, A.S., Hassan, F., ud Din, M.T.M., ud Din, Z.M., Hayyat, K., and Tabassum, R., Comparative Evaluation of Single, Bilateral Intra-Testicular Injection of Hypertonic Saline Solution and Calcium Chloride as Chemical Sterilizing Agents in Male Cats. Biomed J Sci & Tech Res, 2019. 21(3): p. 15858-15864.
64. Canpolat, I., Gur, S., Gunay, C., Bulut, S., and Eroksuz, H., An evaluation of the outcome of bull castration by intra-testicular injection of ethanol and calcium chloride. Revue de Médecine Vétérinaire, 2006. 157: p. 8-9.
65. Jana, K., Samanta, P.K., and Ghosh, D., Evaluation of single intratesticular injection of calcium chloride for nonsurgical sterilization of male Black Bengal goats (Capra hircus): a dose-dependent study. Anim Reprod Sci, 2005. 86(1-2): p. 89-108.
66. Martins, L.T., Gonçalves, M.C., Tavares, K., Gaudêncio, S., Dos Santos Neto, P., Dias, A.L.G., Gava, A., Saito, M., Oliveira, C., and Vieira, A., Castration methods do not affect weight gain and have diverse impacts on the welfare of water buffalo males. Livestock Science - LIVEST SCI, 2011. 140: p. 171-176.
67. Sen, C.C., Yumusak, N., Faundez, R., Temamogullari, F., and Taskin, A., Evaluation of intra-testicular injections of calcium chloride and 4-vinylcyclohexene 1,2 monoepoxide for chemical sterilization in guinea pigs. Pol J Vet Sci, 2017. 20(2): p. 251-260.
68. Ibrahim, A., Ali, M.M., Abou-Khalil, N.S., and Ali, M.F., Evaluation of chemical castration with calcium chloride versus surgical castration in donkeys: testosterone as an endpoint marker. BMC Vet Res, 2016. 12: p. 46.

69. Yamada, P.H., Codognoto, V.M., Rydygier de Ruediger, F., Mayara da Silva, K., Aristizábal, V.V., Kastelic, J.P., Rizzoto, G., Ferreira de Souza, F., Gomes de Soutello, R.V., Mungai Chacur, M.G., and Oba, E., A comparison of immunological, chemical and surgical castration of Nelore bulls. Theriogenology, 2021. 169: p. 9-13.
70. Pereira, L.F., Dias, F.C.F., Miguel, M.P., Honsho, C.S., Tavares, D.C., Hellú, J.A.A., and Souza, F.F., Testicular histological evaluation and serum testosterone concentrations of bulls after chemical castration with calcium chloride. Pesquisa Veterinária Brasileira [online], 2018. 38(8): p. 1554-1563.
71. Mitra, B. and Samanta, P.K., Testicular degeneration of scrub bulls by calcium chloride. Indian journal of veterinary surgery, 2000. 21: p. 37-38.
72. Emir, L., Dadali, M., Sunay, M., Erol, D., Caydere, M., and Ustün, H., Chemical castration with intratesticular injection of 20% hypertonic saline: a minimally invasive method. Urol Oncol, 2008. 26(4): p. 392-6.
73. Kwak, B.K. and Lee, S.H., Intratesticular injection of hypertonic saline : non-invasive alternative method for animal castration model. Dev Reprod, 2013. 17(4): p. 435-40.
74. Kwak, B.K. and Lee, S.-H., Evaluation of Newly Developed Chemical Castration Method: Changes in Hormone Gene Expression of Hypothalamic-Pituitary Axis. Development & Reproduction, 2017. 21(3): p. 307-315.
75. Maadi, M.-A., Behfar, M., Rasaei, A., Shalizar-Jalali, A., Najafi, G., and Mohammadi, V., Chemical castration using an intratesticular injection of mannitol: a preliminary study in a rat model. Turkish Journal of Veterinary & Animal Sciences, 2021. 45(3): p. 519-530.
76. Canpolat, I., Karabulut, E., and Eroksuz, Y., Chemical Castration of Adult and non-Adult Male Dogs with Sodium Chloride Solution. IOSR Journal of Agriculture and Veterinary Science, 2016. 9: p. 09-11.
77. Neto, O.A., Gasperin, B.G., Rovani, M.T., Ilha, G.F., Nóbrega, J.E., Jr., Mondadori, R.G., Gonçalves, P.B., and Antoniazzi, A.Q., Intratesticular hypertonic sodium chloride solution treatment as a method of chemical castration in cattle. Theriogenology, 2014. 82(7): p. 1007-1011.e1.
78. Oliveira, F.C., Ferreira, C.E.R., Haas, C.S., Oliveira, L.G., Mondadori, R.G., Schneider, A., Rovani, M.T., Gonçalves, P.B.D., Vieira, A.D., Gasperin, B.G., and Lucia, T., Jr., Chemical castration in cattle with intratesticular injection of sodium chloride: Effects on stress and inflammatory markers. Theriogenology, 2017. 90: p. 114-119.
79. Shen, B., Jensen, R.G., and Bohnert, H.J., Mannitol Protects against Oxidation by Hydroxyl Radicals. Plant physiology, 1997. 115(2): p. 527-532.
80. Leoci, R., CHEMICAL CASTRATION BY INTRATESTICULAR INJECTION OF A CALCIUM CHLORIDE IN ALCOHOL SOLUTION IN DOGS. 2012.
81. Russell, L.D., Saxena, N.K., and Weber, J.E., Intratesticular injection as a method to assess the potential toxicity of various agents and to study mechanisms of normal spermatogenesis. Gamete Res, 1987. 17(1): p. 43-56.
82. Bergh, A., Collin, O., and Lissbrant, E., Effects of acute graded reductions in testicular blood flow on testicular morphology in the adult rat. Biol Reprod, 2001. 64(1): p. 13-20.
83. Kaya, M., Sertoli cells and various types of multinucleates in the rat seminiferous tubules following temporary ligation of the testicular artery. J Anat, 1986. 144: p. 15-29.
84. Abou-Khalil, N.S., Ali, M.F., Ali, M.M., and Ibrahim, A., Surgical castration versus chemical castration in donkeys: response of stress, lipid profile and redox potential biomarkers. BMC Vet Res, 2020. 16(1): p. 310.
85. Cheng, C.Y., Wong, E.W., Lie, P.P., Li, M.W., Su, L., Siu, E.R., Yan, H.H., Mannu, J., Mathur, P.P., Bonanomi, M., Silvestrini, B., and Mruk, D.D., Environmental toxicants and male reproductive function. Spermatogenesis, 2011. 1(1): p. 2-13.
86. Parizek, J. and Zahor, Z., Effect of cadmium salts on testicular tissue. Nature, 1956. 177(4518): p. 1036.
87. Parizek, J., The destructive effect of cadmium ion on testicular tissue and its prevention by zinc. J Endocrinol, 1957. 15(1): p. 56-63.
88. Mason, K.E., Brown, J.A., Young, J.O., and Nesbit, R.R., CADMIUM-INDUCED INJURY OF THE RAT TESTIS. Anat Rec, 1964. 149: p. 135-48.
89. Laskey, J.W., Rehnberg, G.L., Laws, S.C., and Hein, J.F., Reproductive effects of low acute doses of cadmium chloride in adult male rats. Toxicol Appl Pharmacol, 1984. 73(2): p. 250-5.
90. Shojaeepour, S., Dabiri, S., Dabiri, B., Imani, M., Fekri Soofi Abadi, M., and Hashemi, F., Histopathological Findings of Testicular Tissue Following Cadmium Toxicity in Rats. Iran J Pathol, 2021. 16(4): p. 348-353.
91. Mason, K.E. and Young, J.O., Effects of cadmium upon the excurrent duct system of the rat testis. Anat Rec, 1967. 159: p. 311-324.
92. Ali, I., Damdimopoulou, P., Stenius, U., Adamsson, A., Makela, S.I., Akesson, A., Berglund, M., Hakansson, H., and Halldin, K., Cadmium-induced effects on cellular signaling pathways in the liver of transgenic estrogen reporter mice. Toxicol Sci, 2012. 127(1): p. 66-75.
93. Kar, A.B., Chemical sterilization of male Rhesus monkeys. Endocrinology, 1961. 69: p. 1116-9.
94. Setty, B.S. and Kar, A.B., CHEMICAL STERILIZATION OF MALE FROGS (RANA TIGRINA DAUD.). Gen Comp Endocrinol, 1964. 4: p. 353-9.

95. Chatterjee, S.N. and Kar, A.B., Chemical sterilization of stray dogs. Indian Vet J, 1968. 45(8): p. 649-54.
96. Singh, S.K. and Dominic, C.J., Sterilization of a non-scrotal mammal (Suncus murinus L.) by intratesticular injection of cadmium chloride. Acta Eur Fertil, 1978. 9(1): p. 65-70.
97. Kar, A.B. and Das, R.P., Sterilization of males by intratesticular administration of cadmium chloride. Acta Endocrinologica, 1962. 40(3): p. 321-331.
98. Monsefi, M., Alaee, S., Moradshahi, A., and Rohani, L., Cadmium-induced infertility in male mice. Environ Toxicol, 2009.
99. Ren, Y., Shao, W., Zuo, L., Zhao, W., Qin, H., Hua, Y., Lu, D., Mi, C., Zeng, S., and Zu, L., Mechanism of cadmium poisoning on testicular injury in mice. Oncology Letters, 2019. 18(2): p. 1035-1042.
100. Zhu, Q., Li, X., and Ge, R.S., Toxicological Effects of Cadmium on Mammalian Testis. Front Genet, 2020. 11: p. 527.
101. Prozialeck, W.C., Edwards, J.R., Nebert, D.W., Woods, J.M., Barchowsky, A., and Atchison, W.D., The vascular system as a target of metal toxicity. Toxicol Sci, 2008. 102(2): p. 207-18.
102. Angeli, J.K., Cruz Pereira, C.A., de Oliveira Faria, T., Stefanon, I., Padilha, A.S., and Vassallo, D.V., Cadmium exposure induces vascular injury due to endothelial oxidative stress: the role of local angiotensin II and COX-2. Free Radic Biol Med, 2013. 65: p. 838-848.
103. Setchell, B.P. and Waites, G.M., Changes in the permeability of the testicular capillaries and of the 'blood-testis barrier' after injection of cadmium chloride in the rat. J Endocrinol, 1970. 47(1): p. 81-6.
104. Wan, H.T., Mruk, D.D., Wong, C.K., and Cheng, C.Y., The apical ES-BTB-BM functional axis is an emerging target for toxicant-induced infertility. Trends Mol Med, 2013. 19(7): p. 396-405.
105. Wong, C.H. and Cheng, C.Y., The blood-testis barrier: its biology, regulation, and physiological role in spermatogenesis. Curr Top Dev Biol, 2005. 71: p. 263-96.
106. Li, N., Wang, T., and Han, D., Structural, cellular and molecular aspects of immune privilege in the testis. Front Immunol, 2012. 3: p. 152.
107. Franca, L.R., Auharek, S.A., Hess, R.A., Dufour, J.M., and Hinton, B.T., Blood-tissue barriers: morphofunctional and immunological aspects of the blood-testis and blood-epididymal barriers. Adv Exp Med Biol, 2012. 763: p. 237-59.
108. Wang, L., Yan, M., Li, H., Wu, S., Ge, R., Wong, C.K.C., Silvestrini, B., Sun, F., and Cheng, C.Y., The Non-hormonal Male Contraceptive Adjudin Exerts its Effects via MAPs and Signaling Proteins mTORC1/rpS6 and FAK-Y407. Endocrinology, 2021. 162(1).
109. Hew, K.W., Heath, G.L., Jiwa, A.H., and Welsh, M.J., Cadmium in vivo causes disruption of tight junction-associated microfilaments in rat Sertoli cells. Biol Reprod, 1993. 49(4): p. 840-9.
110. Rios, C. and Méndez-Armenta, M., Cadmium Neurotoxicity, in Encyclopedia of Environmental Health, Nriagu, J., Editor. 2019, Elsevier: Oxford. p. 485-491.
111. Heath, E. and Arowolo, R., The early histopathologic effects of intratesticular injection with hyperosmolar glycerol, glucose or NaCl solutions. Andrologia, 1987. 19(6): p. 654-61.
112. Igdoura, S.A. and Wiebe, J.P., Suppression of spermatogenesis by low-level glycerol treatment. J Androl, 1994. 15(3): p. 234-43.
113. Wiebe, J.P., Biological Contraceptive for Males, Patent, U.S., Editor. 1988, The University of Western Ontario London, Canada: USA.
114. Wiebe, J.P., Barr, K.J., Buckingham, K.D., and Gedded, P.D., Prospects of a male contraceptive based on the selective antispermatogenic action of 1,2,3-trihydroxypropane (THP, glycerol), in Male contraception: advances and future prospects, Zatuchni, G.I., Goldsmith, A., Spieler, J.M., Sciearra, J., and Zatuchni, G.I., Editors. 1986, Harper and Row: Philadelphia. p. 252–270.
115. Wiebe, J.P. and Barr, K.J., The control of male fertility by 1,2,3-trihydroxypropane (THP;glycerol): rapid arrest of spermatogenesis without altering libido, accessory organs, gonadal steroidogenesis, and serum testosterone, LH and FSH. Contraception, 1984. 29(3): p. 291-302.
116. Wiebe, J.P. and Barr, K.J., Suppression of spermatogenesis without inhibition of steroidogenesis by a 1,2,3-trihydroxypropane solution. Life Sci, 1984. 34(18): p. 1747-54.
117. Weinbauer, G.F., Galhotra, M.M., and Nieschlag, E., Focal testicular destruction following intratesticular injection of glycerol in rats. Int J Androl, 1985. 8(5): p. 365-75.
118. Madbouly, H., Korany, R., El-Shahat, K., Eissa, H., and Fathi, M., Efficacy of Intratesticular Glycerol Injection as Male Cat Contraception in Comparison with Two Surgical Approaches. Top Companion Anim Med, 2021. 42: p. 100493.
119. Immegart, H.M. and Threlfall, W.R., Evaluation of intratesticular injection of glycerol for nonsurgical sterilization of dogs. Am J Vet Res, 2000. 61: p. 544–549.
120. Stagg, C.M. and Tribble, R.L., Chemical Castration, Patent, U.S., Editor. 1982, Philips, Roxane, Inc., St. Joseph, MO: USA.
121. Nishimura, N., Kawate, N., Sawada, T., and Mori, J., Chemical castration by a single intratesticular injection of lactic acid in rats and dogs. Journal of Reproduction and Development, 1992. 38: p. 263-266.
122. Hill, G.M., Neville, W.E., Richardson, K.L., Utley, P.R., and Stewart, R.L., Castration Method and Progesterone-Estradiol Implant Effects on Growth Rate of Suckling Calves1, 2. Journal of Dairy Science, 1985. 68(11): p. 3059-3061.

123. Fordyce, G., Hodge, P.B., Beaman, N.J., Laing, A.R., Campero, C., and Shepherd, R.K., An evaluation of calf castration by intra-testicular injection of a lactic acid solution. Aust Vet J, 1989. 66(9): p. 272-6.
124. Giri, S., Yadav, B.P.S., and Panda, S.K., Chemical castration in pigs. The Indian journal of animal sciences, 2002. 72: p. 451-453.
125. Freeman, C. and Coffey, D.S., Sterility in male animals induced by injection of chemical agents into the vas deferens. Fertil Steril, 1973. 24(11): p. 884-90.
126. Kamboj, V.P. and Kar, A.B., Antitesticular effect of metallic and rare earth salts. J Reprod Fertil, 1964. 7: p. 21-8.
127. Kar, A.B., Kamboj, V.P., and Goswami, A., Sterilization of male rhesus monkeys by iron salts. J Reprod Fertil, 1965. 9: p. 115-7.
128. Freund, J., Lipton, M.M., and Thompson, G.E., Aspermatogenesis in the guinea pig induced by testicular tissue and adjuvants. J Exp Med, 1953. 97(5): p. 711-26.
129. Jacobo, P., Guazzone, V.A., Theas, M.S., and Lustig, L., Testicular autoimmunity. Autoimmun Rev, 2011. 10(4): p. 201-4.
130. Eyquem, A. and Kreig, H., Experimental autosensitization of the testis. Ann N Y Acad Sci, 1965. 124(1): p. 270-8.
131. Boughton, B.J. and Spector, W.G., "Auto-immune" testicular lesions induced by injury to the contralateral testis and intradermal injection of adjuvant. The Journal of pathology and bacteriology, 1963. 86: p. 69-74.
132. Katsh, S., ADJUVANTS AND ASPERMATOGENESIS IN THE GUINEA PIG. Int Arch Allergy Appl Immunol, 1964. 24: p. 319-31.
133. Naz, R.K. and Talwar, G.P., Immunological sterilization of male dogs by BCG. Int J Androl, 1981. 4(1): p. 111-28.
134. Talwar, G.P., Naz, R.K., Das, C., and Das, R.P., A practicable immunological approach to block spermatogenesis without loss of androgens. Proc Natl Acad Sci U S A, 1979. 76(11): p. 5882-5.
