# Supplementary material for: Male animal sterilization: history, current practices, and potential methods for replacing castration
Source: Front Vet Sci. 2024 Jul 3;11:1409386. doi: 10.3389/fvets.2024.1409386 (PMC11255590; doi:10.3389/fvets.2024.1409386)
Supplement: Supplementary file 3 [file Table_3.pdf]

Supplemental Table 3 References. Chemical Castration by Injection of Non-hormonal Chemicals into the Reproductive Tract and Potential Sterilizing Effects in Male Animals

Target Organ	Admin[1]	Chemical	Trade Name[3]	Species	Dosage	Original Use	Short-term Pathology	Long-term Pathology	Mechanisms of action	T Decreased[3]	Inflammation	Side-Effects	Refs[4]
Epididymis Vas deferens	Intra-lumen	Sclerosing agents[2]	N/A	Ram, Goat, Dog, Cat, Rat, Mouse, Monkey	Wide range	Numerous chemicals	Inflammation; epididymis swollen	Granulomas and cysts; tissue scarring; azoospermia	Spermatic granuloma; fibrosis; blockage	No	Yes	Adhesions between epididymis and tunica vaginalis; sometimes testicular effects	1
	Intra-lumen plugs	Silicone, polyurethane elastomers, hydrogels, co-polymers	RISUG® ADAM™ Vasalgel®	Rat, Rabbit, Monkey, Man	N/A	Elastomers; gels	Occlusion of vas deferens	Azoospermia; testis and epididymal pathology	Prevention of sperm transport; blockage	No	Yes	Testis focal degeneration; hematoma	2

[1] Administration of the chemical/drug by intra-organ lumenal injection (intra-epididymal, intra-vas deferens)

[2] Sclerosing agents consist of a broad range of caustic chemicals that induce tissue damage and elicit fibrosis: Quinacrine dihydrochloride, zinc arginate, sodium tetradecyl sulfate, potassium permanganate, ethanol +/-ascorbic acid, silver nitrate, formaldehyde, styrene maleic anhydride, chlorhexidine digluconate, lactic acid, methylcyanoacrylate, urea and ethanol, formaldehyde and ethanol, $CaCl_2$ in ethanol

[3] N/A, not applicable; T, testosterone

[4] Supplemental References:

1: Sclerosing Agents [1-10, 11l, 12-16]

2: Intra-lumen plugs [17-23]

References:

1. Freeman, C. and Coffey, D.S., Sterility in male animals induced by injection of chemical agents into the vas deferens. Fertil Steril, 1973. 24(11): p. 884-90.
2. Park, H.K., Paick, S.H., Kim, H.G., Lho, Y.S., and Bae, S.R., Induction of contraception by intraepididymal sclerotherapy. The world journal of men's health, 2014. 32(2): p. 83-86.
3. Dixit, V.P., Agrawal, M., and Jain, H.C., Effects of chemical occlusion of vas deferens on the reproductive organs in gerbil Meriones hurrianae Jerdon. Indian J Exp Biol, 1976. 14(5): p. 617-9.
4. Freeman, C. and Coffey, D.S., Male sterility induced by ethanol injection into the vas deferens. Int J Fertil, 1973. 18(3): p. 129-32.
5. Sharma, J.D., Chinoy, N.J., and Dixit, V.P., Fertility control in vas occluded rats and the biochemical effects of ascorbic acid feeding. Exp Clin Endocrinol, 1983. 82(3): p. 337-41.
6. Raman, G., Purandare, T.V., and Munshi, S.R., Sterility induced in male rats by injection of chemical agents into the vas deferens. Andrologia, 1976. 8(4): p. 321-5.
7. Dixit, V.P., Lohiya, N.K., Arya, M., and Agrawal, M., The effects of chemical occlusion of vas deferens on the testicular function of dog: a preliminary study. Acta Eur Fertil, 1975. 6(4): p. 348-53.
8. Pineda, M.H. and Dooley, M.P., Surgical and chemical vasectomy in the cat. Am J Vet Res, 1984. 45(2): p. 291-300.
9. Pineda, M.H., Reimers, T.J., Faulkner, L.C., Hopwood, M.L., and Seidel, G.E., Jr., Azoospermia in dogs induced by injection of sclerosing agents into the caudae of the epididymides. Am J Vet Res, 1977. 38(6): p. 831-8.
10. Singh, S.K. and Chakravarty, S., Histologic changes in the mouse testis after bilateral vasectomy. Asian J Androl, 2000. 2(2): p. 115-20.
11. Barnett, B.D., Chemical vasectomy of domestic dogs in the Galapagos islands. Theriogenology, 1985. 23(3): p. 499-509.
12. Hoffman, R.A. and Wright, R.G., Fertility control in a non-native population of mountain goats. Northwest Science, 1990. 64(1): p. 1-6.
13. Lewis, R.W. and Garcia, R.R., The results of epididymal ablation by sclerosing agents in the nonhuman primate. Fertil Steril, 1984. 41(3): p. 465-9.
14. Leoci, R., Aiudi, G., Cicirelli, V., Brent, L., Iaria, C., and Lacalandra, G.M., Effects of intratesticular vs intraepididymal calcium chloride sterilant on testicular morphology and fertility in dogs. Theriogenology, 2019. 127: p. 153-160.
15. Kutzler, M.A., Intratesticular and intraepididymal injections to sterilize male cats: From calcium chloride to zinc gluconate and beyond. J Feline Med Surg, 2015. 17(9): p. 772-6.
16. Plant, J.W., Seaman, J.T., and Jakovljevic, D., Non-surgical sterilisation of rams using a sclerosing agent. Aust Vet J, 1979. 55(6): p. 263-4.
17. Chen, Z.W., Gu, Y.Q., Liang, X.W., Wu, Z.G., Yin, E.J., and Li, H., Safety and efficacy of percutaneous injection of polyurethane elastomer (MPU) plugs for vas occlusion in man. Int J Androl, 1992. 15(6): p. 468-72.
18. Sheng-Cai, Z., Shu-Ping, Z., and Rui-Chuan, Y., Intravasal injection of formed-in-place silicone rubber as a method of vas occlusion. International Journal of Andrology, 1992. 15(6): p. 460-464.

19. Sheng-Cai, Z., Yi-He, L., Rui-Chuan, Y., and Shu-Ping, Z., Recovery of fertility after removal of polyurethane plugs from the human vas deferens occluded for up to 5 years. International Journal of Andrology, 1992. 15(6): p. 465-467.
20. Lohiya, N.K., Manivannan, B., and Mishra, P.K., Repeated vas occlusion and non-invasive reversal with styrene maleic anhydride for male contraception in langur monkeys. Int J Androl, 2000. 23(1): p. 36-42.
21. Lohiya, N.K., Manivannan, B., Mishra, P.K., and Pathak, N., Vas deferens, a site of male contraception: an overview. Asian J Androl, 2001. 3(2): p. 87-95.
22. Lohiya, N.K., Manivannan, B., Mishra, P.K., Sriram, S., Bhande, S.S., and Panneerdoss, S., Preclinical evaluation for noninvasive reversal following long-term vas occlusion with styrene maleic anhydride in langur monkeys. Contraception, 2005. 71(3): p. 214-26.
23. Ansari, A.S., Hussain, M., Khan, S.R., and Lohiya, N.K., Relative suitability of DMSO and NaHCO3 for reversal of RISUG® induced long-term contraception. Andrology, 2016. 4(2): p. 306-13.
