# Supplementary material for: Male animal sterilization: history, current practices, and potential methods for replacing castration
Source: Front Vet Sci. 2024 Jul 3;11:1409386. doi: 10.3389/fvets.2024.1409386 (PMC11255590; doi:10.3389/fvets.2024.1409386)
Supplement: Supplementary file 4 [file Table_4.pdf]

Supplemental Table 4 References. Examples of Anti-sperm Antibodies (ASA) Used for Active Immunization

ASA	Species	Protein Type	Protein Function	Main Results	Refs[1]
Sperm adhesion molecule 1 (SPAM1) or PH-20	Guinea Pig	Hyaluronidase and Receptor	Penetration through the cumulus cells surrounding the egg. Also binds to the zona pellucida after acrosome reaction.	Temporal infertility (1 year or longer) and absence of normal sperm in the epididymis	[1, 2]
Epididymal Protease Inhibitor (Eppin)	Monkey	WFDC (whey-acidic protein four-disulfide core) protease inhibitor family	Binds to the semen coagulum protein semenogelin-1 (SEMG1), which transiently inhibits sperm motility after ejaculation.	Temporal infertility (approx. 2 years)	[3]
Human fertilization antigen-1 (FA-1)	Mouse	Glycoprotein	Binds to the zona pellucida (ZP3)	Temporal reduction in fertility (for up to 10 months) and blocked binding to zona pellucida in vitro fertilization	[4]
ADAM metallopeptidase domain 2 (ADAM2) or Fertilin or PH-30	Guinea pig	ADAM family member (membrane-anchored protein)	Sperm-egg adhesion and fusion with the egg plasma membrane	Infertility (recovery not determined)	[5]
80 KDa Human sperm antigen (80 kDa HSA)	Marmoset	Glycoprotein	Progressive motility	Temporal infertility (8-10 weeks after booster dose). Loss of sperm progressive motility	[6]

[1] Supplemental References:

1. Primakoff, P., Woolman-Gamer, L., Tung, K.S., and Myles, D.G., Reversible contraceptive effect of PH-20 immunization in male guinea pigs. Biol Reprod, 1997. 56(5): p. 1142-6.
2. Tung, K.S., Primakoff, P., Woolman-Gamer, L., and Myles, D.G., Mechanism of infertility in male guinea pigs immunized with sperm PH-20. Biol Reprod, 1997. 56(5): p. 1133-41.
3. O'Rand M, G., Widgren, E.E., Sivashanmugam, P., Richardson, R.T., Hall, S.H., French, F.S., VandeVoort, C.A., Ramachandra, S.G., Ramesh, V., and Jagannadha Rao, A., Reversible immunocontraception in male monkeys immunized with eppin. Science, 2004. 306(5699): p. 1189-90.
4. Naz, R.K. and Zhu, X., Recombinant fertilization antigen-1 causes a contraceptive effect in actively immunized mice. Biol Reprod, 1998. 59(5): p. 1095-100.
5. Ramarao, C.S., Myles, D.G., White, J.M., and Primakoff, P., Initial evaluation of fertilin as an immunocontraceptive antigen and molecular cloning of the cynomolgus monkey fertilin beta subunit. Mol Reprod Dev, 1996. 43(1): p. 70-5.
6. Khobarekar, B.G., Vernekar, V., Raghavan, V., Kamada, M., Maegawa, M., and Bandivdekar, A.H., Evaluation of the potential of synthetic peptides of 80 kDa human sperm antigen (80 kDaHSA) for the development of contraceptive vaccine for male. Vaccine, 2008. 26(29-30): p. 3711-8.
