# Supplementary material for: Male animal sterilization: history, current practices, and potential methods for replacing castration
Source: Front Vet Sci. 2024 Jul 3;11:1409386. doi: 10.3389/fvets.2024.1409386 (PMC11255590; doi:10.3389/fvets.2024.1409386)
Supplement: Supplementary file 5 [file Table_5.pdf]

Supplemental Table 5 References. Effects of GnRH-immunization in domestic animals in terms of reproduction, behavior, and production performance[1]

**Effect**	**Age**[2]	**Vaccine**[3] **(Doses / Interval)**	**Main Results**[4]	**Refs**[5]
		**Bull (*Bos taurus* & *Bos indicus*)**		
**Reproduction**	**Prepubertal** <6 months	Bopriva® (2 doses / 3 weeks)	Decreased serum T up to 22 weeks old, and scrotum size up to 44 weeks old.	(2)
	**Peripubertal** 6-14 months	GnRH-OA conjugate (3 Doses / 4-5 months)	Decreased scrotum size for more than a year.	(3)
		Bopriva® (2 Doses / 6 weeks)	Decreased serum T and testis weight up to 15 weeks after booster.	(4)
		Improvac® (3 doses / 3-15 weeks)	Decreased scrotum and testis size. Arrested spermatogenesis up to 20 weeks after booster.	(5)
	**Adult** >22 months at booster	GnRH-OA & GnRH-Trx conjugates (3 doses / 20 weeks)	Decreased testosterone and testis weight up to 8 months after first booster.	(6)
		Bopriva® (2-3 doses / 1-3 months)	Decreased scrotum size, sperm motility, and testosterone up to 4 months after first booster.	(7, 8)
**Behavior**	**Prepubertal** <6 months	GnRH-KLH conjugate (2 doses / 1-6 months)	Reduced aggressive behavior (frequency of butts and sparring) compared to intact bull.	(9)
	**Postpubertal** 8-22 months	GnRH-KLH conjugate (2 doses / 8 months)	Reduced aggressive behavior (frequency of butts and sparring) compared to intact bull.	(10)
**Performance**	**Postpubertal** 8-22 months	Bopriva® (2 doses / 4-6 weeks)	*Compared to bulls*: Lower ADG, HCW, and dressing percentage. Higher fat thickness and marbling. *Compared to steers*: Higher ADG. Similar HCW, dressing percentage, fat thickness and meat quality.	(4, 11)
	**Adult** >22 months	GnRH-OA conjugate (3 doses / 2 weeks)	*Compared to bulls*: Similar carcass weight, and dressing percentage. Higher fat depth and marbling. *Compared to steers*: Similar carcass weight, and dressing percentage. Higher marbling.	(3)
		Bopriva® (2 doses / 3 months)	*Compared to steers:* Higher ADG and HCW. Lower dressing percentage. Similar meat quality.	(7)
		**Boar**		
**Reproduction**	**Early-treated** ~10 weeks	GnRH-OA conjugate (2 doses / 8 weeks)	Decreased testis size, serum LH, FSH, and T. Absence of mature sperm in seminiferous tubules up to 8 weeks after booster.	(12, 13)

		GnRH-MBP conjugate (2 doses / 8 weeks)	Decreased scrotum size and plasma T. Absence of mature sperm up to 7 weeks after booster.	(14)
		Improvest®/ Improvac® (2 doses / 5-10 weeks)	Decreased testis weight and serum T. Absence of mature sperm in seminiferous tubules up to 10 weeks after booster.	(15, 16)
	**Late-treated** 15-18 weeks	Improvac® (2 doses / 4 weeks)	Decreased testes weight, bulbo-urethral gland weight, and serum LH, T, and androsterone up to 10 weeks after booster.	(17, 18)
**Behavior**	**Early-treated** ~10 weeks	GnRH-MBP conjugate (2 doses / 8 weeks)	Delayed onset of mounting behavior (4 months later than intact boars).	(14)
		Improvac® (2 doses / 10 weeks)	Decreased activity (time standing), sexual (mounting), and aggressive behavior (biting and fighting), and skin lesions compared to intact boars.	(19, 20)
	**Late-treated** 15-16 weeks	Improvest®/ Improvac® (2 doses / 4-6 weeks)	Decreased aggressive and mounting behaviors, and skin lesions compared to intact boars.	(21) (22)
	**Heavy pork** 37-41 weeks	Improvac® (2-4 doses / 4-10 weeks)	Decreased social, aggressive, and mounting behavior compared to intact boars up to 22 weeks after booster.	(23, 24)
**Performance**	**Early-treated** ~10 weeks	Improvac® (2 doses / 9-10 weeks)	*Compared to barrows*: Similar-higher ADG. Lower FCR. Similar-higher lean meat percentage. *Compared to intact boars*: Similar-higher ADG, and FCR. Lower lean meat percentage.	(20, 25)
	**Late-treated** 15-18 weeks	Improvest®/ Improvac® (2 doses / 4-6 weeks)	*Compared to barrows*: Higher ADG and feed intake. Lower FCR. Lower dressing percentage, marbling and bacon slicing yield. Higher lean meat percentage. *Compared to intact boars*: Higher ADG, feed intake, and FCR. Lower dressing percentage and lean meat percentage. Similar bacon characteristics.	(18, 26-34)
	**Heavy pork** 31-41 weeks	Improvac® (2-4 doses / 4-10 weeks)	*Compared to barrows*: Higher ADG and HCW. Lower FCR, dressing percentage, and backfat thickness.	(23, 26)
**Boar Taint**	**Early-treated** ~10 weeks	Improvac® (2 doses / 9 weeks)	*Compared to barrows*: Similar androstenone and skatole concentration in fat. *Compared to intact boars*: Lower androstenone and skatole concentration in fat.	(25)

	**Late-treated** 15-18 weeks	Improvac® (2 doses / 4-6 weeks)	*Compared to barrows*: Similar androstenone and skatole concentration in fat. *Compared to intact boars*: Lower androstenone and skatole concentration in fat.	(18, 30, 31, 35)
	**Heavy pork** 37-41 weeks	Improvac® (2 doses / 4-6 weeks)	*Compared to intact boars*: Lower androstenone and skatole in fat up to 22 weeks after booster.	(24)
**Small Ruminants (Ram & Buck)**				
**Reproduction**	**Prepubertal Lamb** < 4 months	GnRH-OA & GnRH-Trx conjugates (3 doses / 4-8 weeks)	Decreased scrotum size and absence of mature sperm in seminiferous tubules up to 23 weeks after first booster.	(36)
	**Peripubertal Lamb** 4-8 months	GnRH-OA conjugate (2 doses / 4 weeks)	Decreased serum LH, FSH, and T. Decreased scrotum and accessory glands size. Absence of mature sperm in seminiferous tubules up to 22 weeks after booster	(37, 38)
		Improvac® (2 doses / 2-4 weeks)	Decreased scrotum size up to 4 weeks after booster.	(39)
		Bopriva® (2 doses / 4 weeks)	Decreased plasma T, and sperm concentration in ejaculates (azoospermia in 70-80% of males) up to 1 month after booster.	(40)
	**Adult Buck** >1 year	Vaxstrate® (2 doses / 2-4 weeks)	Decreased plasma LH, FSH, and T, sperm concentration in ejaculates, and scrotum size for more than a year in 90% of animals.	(41)
**Behavior**	**Prepubertal Lamb** < 4 months	GnRH-KHL conjugate (Regime not specified)	Decreased sexual behavior (frequency of mounts and ejaculations).	(42)
		GnRH-OA & GnRH-Trx conjugates (3 doses / 4-8 weeks)	Delayed onset of mounting activity (5 weeks later compared to intact ram).	(36)
	**Adult Buck** >1 year	Vaxstrate® (2 doses / 2-4 weeks)	Decreased agonistic behavior and male odor associated with reproductive season.	(41)
**Performance**	**Prepubertal Lamb** < 4 months	GnRH-KHL conjugate (Regime not specified)	*Compared to physically castrate:* Similar ADG, FCR and dressing percentage. Lower marbling and back fat thickness. *Compared to intact ram:* Lower ADG. Higher FCR, and dressing percentage. Similar marbling and backfat.	(42)
	**Peripubertal Lamb** 4-8 months	GnRH-OA & GnRH-Trx conjugates (2 doses / 8 weeks)	*Compared to physically castrate:* Similar ADG, HCW, dressing percentage and other carcass measurements. *Compared to intact ram*: Similar ADG, HCW, and dressing percentage. Higher subcutaneous fat.	(43)

		Improvac®<br>(2 doses / 2-4 weeks)	*Compared to intact ram:* Similar ADG, HCW, and dressing percentage.	(39)
**Dog**				
**Reproduction & Behavior**	**Prepubertal**<br><6 months	GnRH-CDV Th cell epitope p35 conjugate<br>(2 doses / 4 weeks)	Decreased testes size and absence of mature sperm in seminiferous tubules up to 14 weeks after booster (3/8 dogs).	(44)
	**Adult**<br>>1 year	GnRH-TT conjugate<br>(3 doses / 2-6 weeks)	Decreased serum T and scrotum size up to 28 weeks after booster (5/12 dogs), and absence of mature sperm in seminiferous tubules.	(45)
		Canine GnRH Immuno-therapeutic®<br>(2 doses / 4 weeks)	Decreased serum LH, serum T, and testicular volume up to 8 weeks after booster (4/4 dogs).	(46)
		GnRXG/Q antigen recombinant protein<br>(2 doses / 4 weeks)	Decreased serum T and sperm concentration in ejaculates up to 8 months after booster (5/7 dogs). Decreased sexual, agonistic, and marking behavior.	(47)
**Cat**				
**Reproduction**	**Prepubertal**<br><4 months	GnRH-LKTA conjugate<br>(2 doses / 4 weeks + 2-year dose	Decreased serum T and absence of mature sperm in seminiferous tubules (3/4 cats).	(48)
	**Peripubertal**<br>4-9 months	GnRH-MBP conjugate<br>(2 doses / 6 weeks)	Decreased serum T, testis size and absence of mature sperm in seminiferous tubules up to 6 weeks after booster.	(49)
		GnRH-STF2 conjugate<br>(2 doses / 4 weeks)	Decreased serum T, testis size and number of mature sperm in seminiferous tubules up to 5 months after booster (6/14 cats).	(50)
	**Adult**<br>>9 months	GnRH-KHL conjugate<br>(2 doses / not specified)	Decreased serum T and scrotal size. Absence of sperm in ejaculates at least up to 6 months after booster (6/9 cats).	(51)
		Improvac®<br>(2 doses / 4 weeks)	Decreased serum T, scrotal size, and sperm concentration in ejaculates up to 20 weeks after booster.	(52)
**Stallion**				
**Reproduction & Behavior**	**Young Horse (<4 years)**	Improvac®<br>(2 doses / 4 weeks)	Decreased serum T and testis size up to 10 weeks after booster	(53)
	**Adult Horse (>4 years)**	Equity™<br>(3 doses / 4-8 weeks)	Decreased plasma T, scrotal circumference, libido, and sperm concentration in ejaculates up to 6-8 months after booster.	(54)
	**Adult Pony**	GnRH- OA conjugate<br>(2 doses / 2 weeks)	Decreased serum T, scrotal size, and libido up to 6-14 months after booster. Low or absence of mature sperm in seminiferous tubules.	(55)

[1] Others have reviewed studies before 2000 (1), therefore this table mainly includes studies thereafter.

[2] Age refers to age at booster or second immunization, except for boars. In boar section, *early-treated* and *late-treated* refers to age at first immunization, and *heavy pork* refers to age of slaughter (37-41 weeks, instead of the conventional 24-26 weeks old).

[3] *Vaccines Abbreviations*: OA = Ovoalbumin; Trx = Thioredoxin; MBP = Matose binding protein; KLH = keyhole limpet hemocyanin; TT = Tetanus toxoid; CDV = canine distemper virus; Th = T helper; LKTA = leukotoxin A; STF2 = Salmonella typhimurium flagellin fljB. *Hormone Abbreviations*: FSH = Follicle Stimulating Hormone; LH = Luteinizing Hormone; T = Testosterone.

[4] *Production Performance Abbreviations*: BW = Body Weight; HCW = Hot Carcass Weight (Kg; weight of the carcass after slaughter and the removal of the head, hide, intestinal tract, and internal organs); ADG = Average daily gain (Kg/day; increase in BW per day); FCR = Feed conversation ratio (Kg/Kg; weight of feed intake divided by BW gained); Dressing percentage or carcass yield (Kg/Kg; HCW expressed as the percentage of the BW at slaughter); Lean meat percentage (Kg/Kg; sum of denuded shoulder, back and ham weights as percentage of cold carcass weight); Marbling (intermingling of fat with lean in a muscle); Bacon slicing yield (Kg/Kg) = percentage of green weight (weight of fresh pork bellies after being skinned and trimmed).

[5] Supplemental References:

1. Thompson DL. Immunization against GnRH in male species (comparative aspects). Anim Reprod Sci. 2000;60-61:459-69.
2. Janett F, Gerig T, Tschuor AC, Amatayakul-Chantler S, Walker J, Howard R, et al. Effect of vaccination against gonadotropin-releasing factor (GnRF) with Bopriva(R) in the prepubertal bull calf. Anim Reprod Sci. 2012;131(1-2):72-80.
3. D'Occhio MJ, Aspden WJ, Trigg TE. Sustained testicular atrophy in bulls actively immunized against GnRH: potential to control carcase characteristics. Anim Reprod Sci. 2001;66(1-2):47-58.
4. Amatayakul-Chantler S, Jackson JA, Stegner J, King V, Rubio LM, Howard R, et al. Immunocastration of Bos indicus x Brown Swiss bulls in feedlot with gonadotropin-releasing hormone vaccine Bopriva provides improved performance and meat quality. J Anim Sci. 2012;90(11):3718-28.
5. Monleon E, Noya A, Carmen Garza M, Ripoll G, Sanz A. Effects of an anti-gonadotrophin releasing hormone vaccine on the morphology, structure and function of bull testes. Theriogenology. 2020;141:211-8.
6. Hernandez JA, Zanella EL, Bogden R, de Avila DM, Gaskins CT, Reeves JJ. Reproductive characteristics of grass-fed, luteinizing hormone-releasing hormone-immunocastrated Bos indicus bulls. J Anim Sci. 2005;83(12):2901-7.
7. Amatayakul-Chantler S, Hoe F, Jackson JA, Roca RO, Stegner JE, King V, et al. Effects on performance and carcass and meat quality attributes following immunocastration with the gonadotropin releasing factor vaccine Bopriva or surgical castration of Bos indicus bulls raised on pasture in Brazil. Meat Sci. 2013;95(1):78-84.
8. Yamada PH, Codognoto VM, Rydygier de Ruediger F, Mayara da Silva K, Aristizábal VV, Kastelic JP, et al. A comparison of immunological, chemical and surgical castration of Nelore bulls. Theriogenology. 2021;169:9-13.
9. Huxsoll CC, Price EO, Adams TE. Testis function, carcass traits, and aggressive behavior of beef bulls actively immunized against gonadotropin-releasing hormone. J Anim Sci. 1998;76(7):1760-6.
10. Price EO, Adams TE, Huxsoll CC, Borgwardt RE. Aggressive behavior is reduced in bulls actively immunized against gonadotropin-releasing hormone. J Anim Sci. 2003;81(2):411-5.
11. Marti S, Jackson JA, Slootmans N, Lopez E, Hodge A, Pérez-Juan M, et al. Effects on performance and meat quality of Holstein bulls fed high concentrate diets without implants following immunological castration. Meat Sci. 2017;126:36-42.

12. Oonk HB, Turkstra JA, Schaaper WM, Erkens JH, Schuitemaker-de Weerd MH, van Nes A, et al. New GnRH-like peptide construct to optimize efficient immunocastration of male pigs by immunoneutralization of GnRH. Vaccine. 1998;16(11-12):1074-82.
13. Han X, Zhou Y, Zeng Y, Sui F, Liu Y, Tan Y, et al. Effects of active immunization against GnRH versus surgical castration on hypothalamic-pituitary function in boars. Theriogenology. 2017;97:89-97.
14. Fang F, Li H, Liu Y, Zhang Y, Tao Y, Li Y, et al. Active immunization with recombinant GnRH fusion protein in boars reduces both testicular development and mRNA expression levels of GnRH receptor in pituitary. Anim Reprod Sci. 2010;119(3-4):275-81.
15. Lugar DW, Rhoads ML, Clark-Deener SG, Callahan SR, Revercomb AK, Prusa KJ, Estienne MJ. Immunological castration temporarily reduces testis size and function without long-term effects on libido and sperm quality in boars. Animal. 2017;11(4):643-9.
16. Stojanovic S, Uscebrka G, Zikic D, Stukelj M. Histological and Morphometric Examination of the Testes of Boars and Male Pigs Immunocastrated with Improvac®. ACTA Scientiae Veterinariae. 2017;45(1488).
17. Claus R, Lacorn M, Danowski K, Pearce MC, Bauer A. Short-term endocrine and metabolic reactions before and after second immunization against GnRH in boars. Vaccine. 2007;25(24):4689-96.
18. Dunshea FR, Colantoni C, Howard K, McCauley I, Jackson P, Long KA, et al. Vaccination of boars with a GnRH vaccine (Improvac) eliminates boar taint and increases growth performance. J Anim Sci. 2001;79(10):2524-35.
19. Baumgartner JL, S.; Koller, M.; Pfützner, A.; Grodzycki, M.; Andrews, S.; Schmoll F. The behaviour of male fattening pigs following either surgical castration or vaccination with a GnRF vaccine. Applied animal behaviour science. 2010;124:28-34.
20. Font IFM, Gispert M, Soler J, Diaz M, Garcia-Regueiro JA, Diaz I, Pearce MC. Effect of vaccination against gonadotrophin-releasing factor on growth performance, carcass, meat and fat quality of male Duroc pigs for dry-cured ham production. Meat Sci. 2012;91(2):148-54.
21. Rydhmer L, Lundstrom K, Andersson K. Immunocastration reduces aggressive and sexual behaviour in male pigs. Animal. 2010;4(6):965-72.
22. Puls CL, Rojo A, Matzat PD, Schroeder AL, Ellis M. Behavior of immunologically castrated barrows in comparison to gilts, physically castrated barrows, and intact male pigs. J Anim Sci. 2017;95(6):2345-53.
23. Pesenti Rossi G, Dalla Costa E, Filipe JFS, Mazzola SM, Motta A, Borciani M, et al. Does Immunocastration Affect Behaviour and Body Lesions in Heavy Pigs? Vet Sci. 2022;9(8).
24. Zamaratskaia G, Rydhmer L, Andersson HK, Chen G, Lowagie S, Andersson K, Lundstrom K. Long-term effect of vaccination against gonadotropin-releasing hormone, using Improvac, on hormonal profile and behaviour of male pigs. Anim Reprod Sci. 2008;108(1-2):37-48.
25. Pauly C, Spring P, O'Doherty JV, Ampuero Kragten S, Bee G. Growth performance, carcass characteristics and meat quality of group-penned surgically castrated, immunocastrated (Improvac(R)) and entire male pigs and individually penned entire male pigs. Animal. 2009;3(7):1057-66.
26. Palma-Granados P, Lara L, Seiquer I, Lachica M, Fernández-Fígares I, Haro A, Nieto R. Protein retention, growth performance and carcass traits of individually housed immunocastrated male- and female- and surgically castrated male Iberian pigs fed diets of increasing amino acid concentration. Animal. 2021;15(4):100187.
27. Puls CL, Rojo A, Ellis M, Boler DD, McKeith FK, Killefer J, et al. Growth performance of immunologically castrated (with Improvest) barrows (with or without ractopamine) compared to gilt, physically castrated barrow, and intact male pigs. J Anim Sci. 2014;92(5):2289-95.
28. Oliver WT, McCauley I, Harrell RJ, Suster D, Kerton DJ, Dunshea FR. A gonadotropin-releasing factor vaccine (Improvac) and porcine somatotropin have synergistic and additive effects on growth performance in group-housed boars and gilts. J Anim Sci. 2003;81(8):1959-66.

29. Boler DD, Puls CL, Clark DL, Ellis M, Schroeder AL, Matzat PD, et al. Effects of immunological castration (Improvest) on changes in dressing percentage and carcass characteristics of finishing pigs. J Anim Sci. 2014;92(1):359-68.
30. Weiler U, Gotz M, Schmidt A, Otto M, Muller S. Influence of sex and immunocastration on feed intake behavior, skatole and indole concentrations in adipose tissue of pigs. Animal. 2013;7(2):300-8.
31. Zamaratskaia G, Andersson HK, Chen G, Andersson K, Madej A, Lundstrom K. Effect of a gonadotropin-releasing hormone vaccine (Improvac) on steroid hormones, boar taint compounds and performance in entire male pigs. Reprod Domest Anim. 2008;43(3):351-9.
32. Kyle JM, Bohrer BM, Schroeder AL, Matulis RJ, Boler DD. Effects of immunological castration (Improvest) on further processed belly characteristics and commercial bacon slicing yields of finishing pigs. J Anim Sci. 2014;92(9):4223-33.
33. Little KL, Kyle JM, Bohrer BM, Schroeder AL, Fedler CA, Prusa KJ, Boler DD. A comparison of slice characteristics and sensory characteristics of bacon from immunologically castrated barrows with bacon from physically castrated barrows, boars, and gilts. J Anim Sci. 2014;92(12):5769-77.
34. Tavárez MA, Bohrer BM, Asmus MD, Schroeder AL, Matulis RJ, Boler DD, Dilger AC. Effects of immunological castration and distiller's dried grains with solubles on carcass cutability and commercial bacon slicing yields of barrows slaughtered at two time points. J Anim Sci. 2014;92(7):3149-60.
35. Moore KL, Mullan BP, Dunshea FR. Boar taint, meat quality and fail rate in entire male pigs and male pigs immunized against gonadotrophin releasing factor as related to body weight and feeding regime. Meat Sci. 2017;125:95-101.
36. Ulker H, Kanter M, Gokdal O, Aygun T, Karakus F, Sakarya ME, et al. Testicular development, ultrasonographic and histological appearance of the testis in ram lambs immunized against recombinant LHRH fusion proteins. Anim Reprod Sci. 2005;86(3-4):205-19.
37. Ulker H, Yilmaz A, Karakus F, Yoruk M, Budag C, deAvila DM, Reeves JJ. LHRH fusion protein immunization alters testicular development, ultrasonographic and histological appearance of ram testis. Reprod Domest Anim. 2009;44(4):593-9.
38. Han X, Ren X, Zeng Y, Zhou Y, Song T, Cao X, et al. Physiological interactions between the hypothalamic-pituitary-gonadal axis and spleen in rams actively immunized against GnRH. Int Immunopharmacol. 2016;38:275-83.
39. Needham TL, H.; Hoffman, L.C. The influence of vaccination interval on growth, carcass traits and testicle parameters of immunocastrated ram lambs. Small Ruminant Research. 2016;145:53-7.
40. Rocha LF, Souza RS, Santana ALA, Macedo DS, Santana AMS, Silva RCd, et al. Reproductive parameters of lambs immunocastrated with anti-GnRH vaccine. Animal Reproduction. 2021;18.
41. Godfrey SIW-B, S.W.; Martin, G.B.; Speijers, E.J. Immunisation of goat bucks against GnRH to prevent seasonal reproductive and agonistic behaviour Animal reproduction science. 1996;44:41-54.
42. Kiyma Z, Adams TE, Hess BW, Riley ML, Murdoch WJ, Moss GE. Gonadal function, sexual behavior, feedlot performance, and carcass traits of ram lambs actively immunized against GnRH. J Anim Sci. 2000;78(9):2237-43.
43. Ülker HG, Ö.; Temu,r C.; Budağ, C.; Oto, M.; deAvila, D.M.; Reeves, J.J. The effect of immunization against LHRH on body growth and carcass characteristics in Karakaş ram lambs. Small Ruminant Research. 2002;45:273-8.
44. Jung MJ, Moon YC, Cho IH, Yeh JY, Kim SE, Chang WS, et al. Induction of castration by immunization of male dogs with recombinant gonadotropin-releasing hormone (GnRH)-canine distemper virus (CDV) T helper cell epitope p35. J Vet Sci. 2005;6(1):21-4.
45. Ladd A, Tsong YY, Walfield AM, Thau R. Development of an antifertility vaccine for pets based on active immunization against luteinizing hormone-releasing hormone. Biol Reprod. 1994;51(6):1076-83.

46. Donovan CE, Greer M, Kutzler MA. Physiologic responses following gonadotropin-releasing hormone immunization in intact male dogs. Reprod Domest Anim. 2012;47 Suppl 6:403-5.
47. Siel D, Ubilla M, Vidal S, Loaiza A, Quiroga J, Cifuentes F, et al. Reproductive and Behavioral Evaluation of a New Immunocastration Dog Vaccine. Animals. 2020;10:226.
48. Robbins SC, Jelinski MD, Stotish RL. Assessment of the immunological and biological efficacy of two different doses of a recombinant GnRH vaccine in domestic male and female cats (Felis catus). J Reprod Immunol. 2004;64(1-2):107-19.
49. Jiang S, Hong M, Su S, Song M, Tian Y, Cui P, et al. Effect of active immunization against GnRH-I on the reproductive function in cat. Anim Sci J. 2015;86(8):747-54.
50. Lee YJ, Jo EJ, Lee HW, Hwang BR, Kim YH, Park BJ, et al. Evaluation of infertility efficacy of the E. coli expressed STF2-GnRH vaccine in male cats. J Vet Sci. 2019;20(3):e30.
51. Levy JK, Miller LA, Cynda Crawford P, Ritchey JW, Ross MK, Fagerstone KA. GnRH immunocontraception of male cats. Theriogenology. 2004;62(6):1116-30.
52. Ochoa JS, Favre RN, Garcia MF, Stornelli MC, Sangache WC, Rearte R, et al. Immunocontraception of male domestic cats using GnRH vaccine Improvac. Theriogenology. 2023;198:211-6.
53. Birrell JR, Schulman ML, Botha AE, Ganswindt A, Fosgate GT, Bertschinger HJ. Vaccination against GnRH as a prelude to surgical castration of horses. Equine Vet J. 2021;53(6):1141-9.
54. Janett F, Stump R, Burger D, Thun R. Suppression of testicular function and sexual behavior by vaccination against GnRH (Equity) in the adult stallion. Anim Reprod Sci. 2009;115(1-4):88-102.
55. Turkstra JA, van der Meer FJ, Knaap J, Rottier PJ, Teerds KJ, Colenbrander B, Meloen RH. Effects of GnRH immunization in sexually mature pony stallions. Anim Reprod Sci. 2005;86(3-4):247-59.
